# Proton Pump Inhibitor Use and Incident Epilepsy in the UK Biobank: Evidence From Pharmacovigilance and Multi‐Omics Analyses

**DOI:** 10.1002/cns.71223

**Published:** 2026-11-04

**Authors:** Minghao Li, Yiqian Li, Yuxi Zhang, Balkissou Seebo Saibou, Yifei Chen, Jiayi Xing, Chao Jin, Yuchen Xu, Jiahe Lin, Xinshi Wang

**Affiliations:** ^1^ Department of Neurology The First Affiliated Hospital of Wenzhou Medical University Wenzhou Zhejiang Province People's Republic of China; ^2^ Key Laboratory of Alzheimer's Disease of Zhejiang Province, Institute of Aging Wenzhou Medical University Wenzhou Zhejiang Province People's Republic of China; ^3^ Key Laboratory of Novel Nuclide Technologies on Precision Diagnosis and Treatment & Clinical Transformation of Wenzhou City Wenzhou Zhejiang Province People's Republic of China

**Keywords:** epilepsy, FAERS, inflammation, metabolomics, pharmacovigilance, proteomics, proton pump inhibitors, UK biobank

## Abstract

**Aims:**

Proton pump inhibitors (PPIs) are widely used, but their association with incident epilepsy remains uncertain. We examined this association in the UK Biobank and integrated evidence from pharmacovigilance and multi‐omics.

**Methods:**

We included 492,858 UK Biobank participants without epilepsy at baseline. Cox models, age‐ and sex‐stratified analyses, active‐comparator analyses against H2RA users, propensity score matching, competing‐risk models, and sensitivity analyses were performed. FAERS disproportionality, Olink proteomics, and NMR metabolomics provided supportive evidence.

**Results:**

Baseline PPI use was associated with incident epilepsy after full adjustment (HR 1.43, 95% CI 1.29–1.59) and in the PPI‐H2RA propensity score‐matched analysis (HR 1.41, 95% CI 1.03–1.94). In the GP‐linked subset, requiring an ASM prescription within 365 days after diagnosis yielded HR 1.47 (95% CI 1.07–2.02). FAERS showed a PPI‐epilepsy reporting signal (ROR 1.70, 95% CI 1.61–1.79). Proteomic and metabolomic analyses identified immune‐inflammatory and metabolic associations.

**Conclusion:**

Baseline PPI use was associated with incident epilepsy in the UK Biobank. Active‐comparator, outcome‐definition, and prescription‐supported analyses supported the primary association. Pharmacovigilance and molecular findings provided complementary evidence but did not establish causality.

## Graphical abstract

Baseline PPI use was associated with a higher risk of incident epilepsy in 492,858 UK Biobank participants, with consistent evidence from active‐comparator and sensitivity analyses. FAERS, proteomic, and metabolomic findings provided complementary pharmacovigilance and molecular context, without establishing causality.

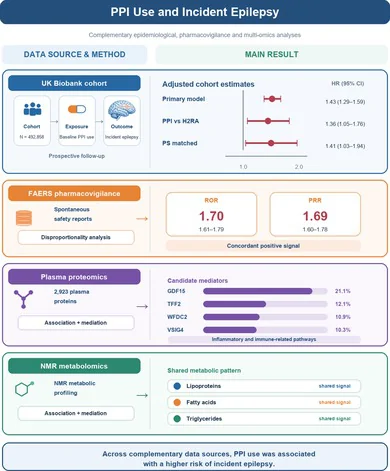

## 1 Introduction

Epilepsy affects approximately 50 million people worldwide and remains a major contributor to neurological morbidity [1, 2]. Although structural, genetic, infectious, and metabolic causes are recognized, many adult‐onset cases lack a clearly modifiable upstream factor [3, 4, 5]. Identifying medication exposures associated with seizure susceptibility is therefore relevant to prevention and prescribing.

Proton pump inhibitors (PPIs) are widely prescribed for gastroesophageal reflux disease, peptic ulcer disease, and *Helicobacter pylori* eradication [6, 7]. Their benefits are established when appropriately indicated, but long‐term safety and the risk of overuse remain concerns [7, 8, 9, 10, 11]. PPI‐related hypomagnesemia and hyponatremia are clinically relevant because electrolyte disturbances can precipitate acute symptomatic seizures [12, 13, 14].

Other mechanisms may also be relevant. PPIs may influence vacuolar‐type H+‐ATPase, lysosomal acidification, autophagy, and inflammasome signaling; alter the gut microbiome and gut‐brain communication; and interact with hERG potassium channels involved in membrane‐potential regulation [15, 16, 17, 18]. Together, these observations support population‐level investigation.

Evidence linking acid‐suppressive therapy to epilepsy remains limited. Observational associations have been reported among adults receiving long‐term PPI therapy [19, 20] and among children exposed to gastric acid inhibitors prenatally or in early childhood [21]. Whether PPI use is associated with incident epilepsy later in adult life remains uncertain, particularly when differences in treatment indication are considered.

We therefore examined baseline PPI use and incident epilepsy in the UK Biobank, evaluated robustness using active‐comparator, propensity score‐matched, competing‐risk, subgroup, and sensitivity analyses, and used FAERS, plasma proteomics, and NMR metabolomics as supportive evidence.

## 2 Methods

### 2.1 Study Population and Design

The primary analysis was conducted among UK Biobank participants with baseline medication information and linked follow‐up data available [22, 23]. We excluded participants with a prior diagnosis of epilepsy or seizure disorder at baseline (ICD‐10 G40 or G41), those missing body mass index, and those missing Townsend deprivation index, yielding a final analytical cohort of 492,858 participants. Definitions of exposure, outcome, covariates, and medication codes are provided in Table S1.

Baseline PPI use was ascertained from self‐reported regular medication use at the baseline assessment. Participants reporting regular use of omeprazole, lansoprazole, esomeprazole, rabeprazole, or pantoprazole were classified as PPI users. Incident epilepsy was defined as a first post‐baseline record of ICD‐10 G40 or G41 in linked Hospital Episode Statistics, Scottish Morbidity Record, or death registration data. Participants were followed from baseline assessment until incident epilepsy, death, or administrative censoring on 31 May 2022.

### 2.2 Cox Regression, Active‐Comparator, and Competing‐Risk Analyses

Cox proportional hazards regression was used to estimate hazard ratios (HRs) and 95% confidence intervals (CIs). Model 1 adjusted for age and sex. Model 2 additionally adjusted for body mass index, smoking status, alcohol intake, ethnicity, educational attainment, and Townsend deprivation index. Model 3 further adjusted for baseline gastrointestinal disease, hypertension, diabetes, and stroke. The proportional hazards assumption was assessed using Schoenfeld residuals. Because age violated this assumption, robustness was examined using a Cox model stratified by 5‐year baseline‐age bands and a model allowing the age effect to vary across 0–5, 5–10, and > 10 years of follow‐up.

To address confounding by indication, we compared PPI users with H2RA users [24, 25]. The active‐comparator propensity score included age, sex, body mass index, Townsend deprivation index, smoking, alcohol intake, ethnicity, education, and baseline acid‐related gastrointestinal disease (ICD‐10 K20, K21, K25–K27, and K29). We used 1:1 nearest‐neighbor matching with a caliper of 0.2 standard deviations of the logit of the propensity score [26]. Absolute standardized mean differences < 0.10 indicated adequate balance. Covariate balance and matched Cox estimates are reported in Tables S3A–C.

Competing risks were evaluated using Fine‐Gray subdistribution hazard models with all‐cause death as the competing event [27, 28]. Sensitivity analyses applied one‐ and two‐year landmark periods, excluded baseline antiseizure medication users, and restricted the outcome to G40. Age‐ and sex‐stratified analyses used Model 2 covariates, with the stratifying variable omitted from its corresponding adjustment set. Interaction *p* values were obtained from likelihood‐ratio tests comparing models with and without the PPI‐by‐subgroup interaction term. Additional subgroup and agent‐specific analyses were performed when event counts permitted stable estimation. We also summarized age at the first post‐baseline G40/G41 record by sex and baseline PPI use.

Neurological‐condition sensitivity analyses considered stroke, brain or central nervous system tumor, traumatic brain injury, central nervous system infection, and dementia. For each condition, participants with a diagnosis at or before baseline were excluded. During follow‐up, participants were censored at the first incident diagnosis if it occurred strictly before epilepsy. The five‐condition joint analysis applied the same rule to the earliest occurrence of any of the five conditions. Diagnostic definitions and complete estimates are provided in Table S14E and Figure S1B.

For the GP prescription‐supported outcome analysis, we restricted the sample to 175,614 participants with baseline‐contiguous GP registration and available prescription records. The primary definition required the first post‐baseline G40/G41 diagnosis to be followed strictly later by an eligible maintenance ASM prescription within 365 days. Secondary definitions required confirmation within 90 days or at any subsequent time during observed GP follow‐up. For confirmed cases, the event date was the date of the first qualifying prescription. Models used Model 3 covariates and were stratified by GP provider and baseline smoking‐data availability. Eligible ASM ingredients and code mappings are provided in Table S1E, and complete estimates are reported in Table S14F and Figure S1C.

### 2.3 Independent Pharmacovigilance Signal Analysis Using FAERS

We analyzed 18,747,025 unique spontaneous adverse event reports submitted to the FDA Adverse Event Reporting System from Q1 2004 to Q4 2025 [29]. After excluding 1,636,826 reports involving antiseizure medications or antipsychotics, 17,110,199 reports remained. Drugs were restricted to the primary‐suspect (PS) role, yielding 161,317 PPI reports (1326 epilepsy‐related events) and 1,135,014 chronic non‐gastrointestinal control medication reports. FAERS drug definitions and exclusion rules are described in Table S5.

Disproportionality was quantified using the reporting odds ratio (ROR) and proportional reporting ratio (PRR) with 95% CIs [30, 31, 32]. FAERS was used as an independent external pharmacovigilance dataset at the reporting‐signal level. Complete FAERS disproportionality results are provided in Table S6.

### 2.4 Proteomic Analyses

Plasma proteomic data from the Olink Explore 3072 platform were available for 52,029 participants with 422 incident epilepsy events [33]. Proteomic measurements were derived from plasma samples collected at the UK Biobank baseline assessment between 2006 and 2010. After quality control, 2911 proteins were analyzed. PPI‐protein associations were estimated using multivariable linear regression, and protein‐epilepsy associations were estimated using Cox regression adjusted for the Model 2 covariates. Proteins associated with both PPI use and incident epilepsy at FDR < 0.05 were defined as bridging proteins.

Exploratory observational mediation analysis used the product‐of‐coefficients approach [34]. Candidate mediator proteins had an estimated proportion mediated > 5% and FDR < 0.05 for the indirect effect. Each protein was analyzed separately among participants with complete Model 2 covariates and an available measurement for that protein; missing protein measurements were not imputed. The sign of the protein coefficient in the epilepsy model was used only to describe whether protein levels were positively or inversely associated with epilepsy. STRING v12.0 was used for the descriptive protein–protein interaction analysis, and pathway enrichment was performed using clusterProfiler [35, 36]. For the network analysis, functional associations with a combined interaction score ≥ 0.70 were retained. The largest connected component was selected, after which proteins with degree < 2 were iteratively removed; network communities were identified by greedy modularity optimization. The network was used only to summarize protein connectivity and was not used to infer causal centrality. The same set of 85 candidate mediator proteins was used for GO, Reactome, and KEGG enrichment. Complete results are provided in Tables S7–S13 and Figure S2.

### 2.5 Metabolomic Analyses

NMR metabolomic measurements generated by the Nightingale Health platform from plasma samples collected at the UK Biobank baseline assessment between 2006 and 2010 were available for up to 480,315 participants across 251 metabolites [37, 38]. Before modeling, each metabolite was standardized to a z score in its available analytic sample. PPI‐metabolite associations were estimated using multivariable linear regression and are expressed as SD differences, whereas metabolite‐epilepsy associations were estimated using Cox regression and are expressed as HRs per 1‐SD higher metabolite level. Both models were adjusted for the Model 2 covariates. Shared FDR‐significant metabolites met FDR < 0.05 in both association analyses.

Concordant and discordant directions were defined by positive and negative products, respectively, of the standardized PPI‐metabolite and metabolite‐epilepsy coefficients. Twelve module‐representative metabolites were evaluated using exploratory product‐of‐coefficients mediation analyses. To reduce computational burden, a fixed simple random sample of 100,000 participants was drawn without replacement from the 480,315 participants with baseline NMR data using a prespecified random seed. Metabolite‐specific complete‐case samples included 99,733–100,000 participants and 741–744 events; missing values were not imputed. The mediator and outcome models used Model 2 covariates. Indirect, direct, and total effects were estimated on the log‐hazard scale and exponentiated to HRs. Percentile 95% CIs were obtained from 200 nonparametric bootstrap samples. Because this finite bootstrap distribution provides limited *p*‐value resolution, inference was based on percentile 95% CIs rather than bootstrap *p* values. Figure 5D summarizes ten prespecified biochemical modules according to the mean coefficient product among shared metabolites. Complete results are provided in Tables S15–S19 and Figures S3 and S4.

### 2.6 Statistical Software and Ethics

All statistical analyses were conducted in R version 4.5.1 using survival, MatchIt, mediation, and clusterProfiler [34, 39]. Table 1 summarizes baseline characteristics. Figure 1 reports cohort construction. Figure 2 summarizes cumulative incidence, sequential Cox models, the revised active‐comparator analysis, and FAERS disproportionality. Figure 3 reports subgroup and agent‐specific UK Biobank and FAERS analyses. Figures 4 and 5 summarize the proteomic and metabolomic analyses, respectively.

**TABLE 1:** Baseline characteristics of participants by PPI use status.

Characteristic	Overall (*N* = 492,858)	PPI users (*N* = 49,451)	Non‐PPI users (*N* = 443,407)	SMD
Age, mean (SD), years	56.5 (8.1)	59.7 (7.2)	56.2 (8.1)	0.452
Body mass index, mean (SD), kg/m^2^	27.4 (4.8)	29.2 (5.2)	27.2 (4.7)	0.407
Townsend deprivation index, mean (SD)	−1.3 (3.1)	−0.9 (3.3)	−1.4 (3.1)	0.148
Sex, *n* (%)				0.011
Female	268,549 (54.5)	27,178 (55.0)	241,371 (54.4)	
Male	224,309 (45.5)	22,273 (45.0)	202,036 (45.6)	
Smoking status, *n* (%)				0.196
Never	268,662 (54.5)	22,783 (46.1)	245,879 (55.5)	
Former	170,092 (34.5)	20,828 (42.1)	149,264 (33.7)	
Current	51,617 (10.5)	5465 (11.1)	46,152 (10.4)	
Missing	2487 (0.5)	375 (0.8)	2112 (0.5)	
Alcohol intake, *n* (%)				0.222
Never	39,038 (7.9)	6058 (12.3)	32,980 (7.4)	
Low	111,488 (22.6)	13,379 (27.1)	98,109 (22.1)	
Moderate	241,010 (48.9)	21,128 (42.7)	219,882 (49.6)	
High	100,265 (20.3)	8735 (17.7)	91,530 (20.6)	
Missing	1057 (0.2)	151 (0.3)	906 (0.2)	
Ethnicity, *n* (%)				0.035
White	463,770 (94.1)	46,801 (94.6)	416,969 (94.0)	
Asian	11,056 (2.2)	1037 (2.1)	10,019 (2.3)	
Black	7835 (1.6)	629 (1.3)	7206 (1.6)	
Mixed	2904 (0.6)	246 (0.5)	2658 (0.6)	
Other/Unknown	7293 (1.5)	738 (1.5)	6555 (1.5)	
Education, *n* (%)				0.363
Degree	158,533 (32.2)	10,417 (21.1)	148,116 (33.4)	
Below degree	241,688 (49.0)	24,072 (48.7)	217,616 (49.1)	
None	83,183 (16.9)	13,917 (28.1)	69,266 (15.6)	
Missing	9454 (1.9)	1045 (2.1)	8409 (1.9)	
Gastrointestinal disease at baseline, *n* (%)				1.413
No	428,172 (86.9)	18,444 (37.3)	409,728 (92.4)	
Yes	64,686 (13.1)	31,007 (62.7)	33,679 (7.6)	
Hypertension at baseline, *n* (%)				0.409
No	360,368 (73.1)	27,690 (56.0)	332,678 (75.0)	
Yes	132,490 (26.9)	21,761 (44.0)	110,729 (25.0)	
Diabetes at baseline, *n* (%)				0.230
No	467,154 (94.8)	44,177 (89.3)	422,977 (95.4)	
Yes	25,704 (5.2)	5274 (10.7)	20,430 (4.6)	
Stroke at baseline, *n* (%)				0.148
No	485,310 (98.5)	47,680 (96.4)	437,630 (98.7)	
Yes	7548 (1.5)	1771 (3.6)	5777 (1.3)	
Incident epilepsy, *n* (%)	3488 (0.71)	639 (1.29)	2849 (0.64)	—
Follow‐up, median (IQR), years	13.2 (12.5–13.9)	13.2 (12.5–13.9)	13.2 (12.5–13.9)	—

*Note:* SMDs are shown to describe baseline differences between PPI users and non‐users; values < 0.10 are commonly considered to indicate good balance.

Abbreviations: BMI, body mass index; IQR, interquartile range; PPI, proton pump inhibitor; SD, standard deviation; SMD, standardized mean difference.

**FIGURE 1:**
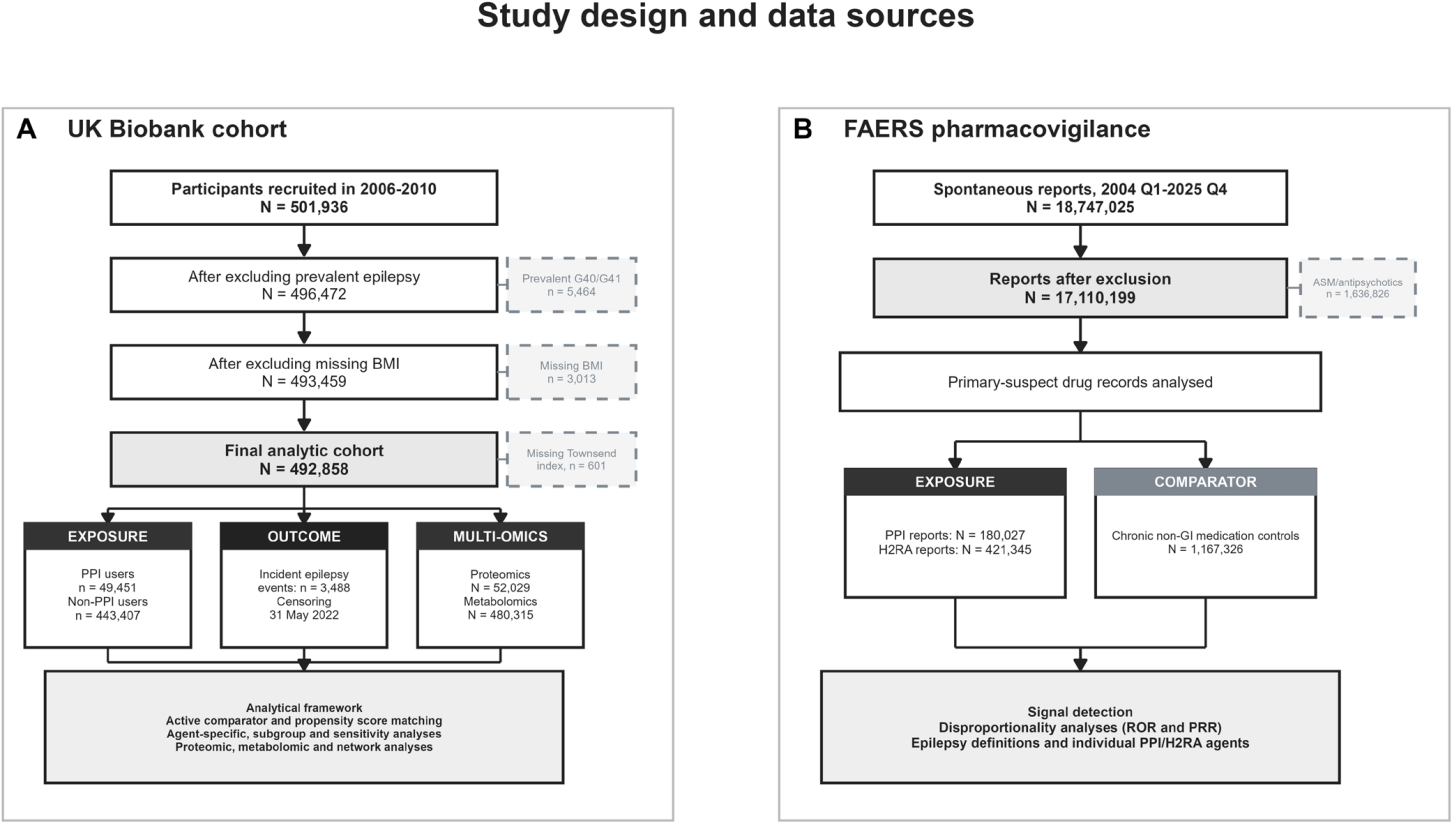
Study flow diagram. Left panel: UK Biobank cohort inclusion and exclusion cascade. Right panel: FAERS dataset construction. The diagram summarizes the analytical cohorts for the primary epidemiological analysis, active‐comparator analysis, proteomic analysis, metabolomic analysis, and FAERS pharmacovigilance signal analysis.

**FIGURE 2:**
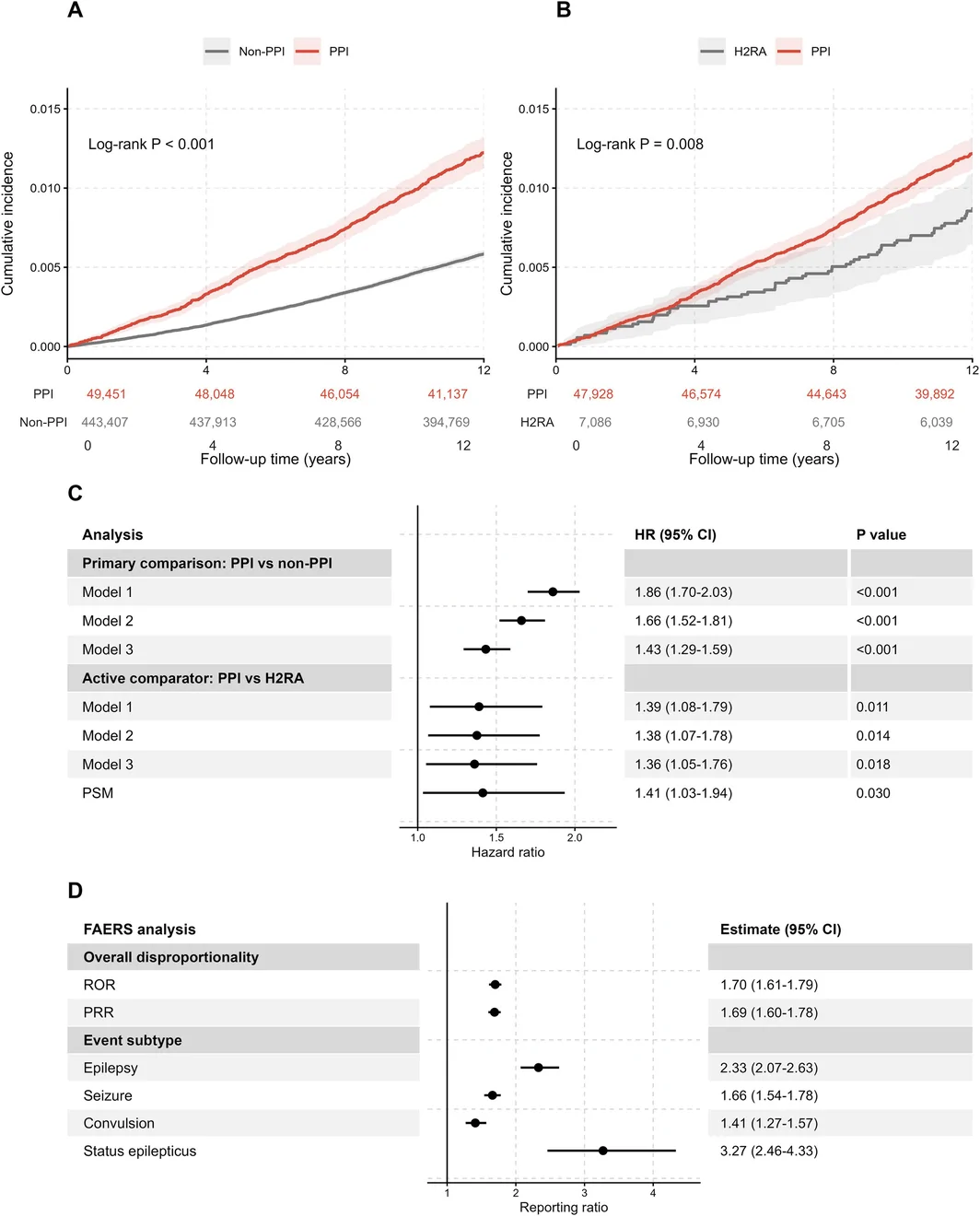
Epidemiological and pharmacovigilance evidence. (A) Cumulative incidence of incident epilepsy by baseline PPI use. (B) Active‐comparator cumulative incidence comparing mutually exclusive PPI and H2RA users. (C) Forest plot of sequential primary and active‐comparator Cox models and the revised PSM estimate incorporating baseline acid‐related gastrointestinal disease. (D) Overall and event‐subtype FAERS disproportionality. FAERS estimates are reporting signals rather than incidence or causal effects.

**FIGURE 3:**
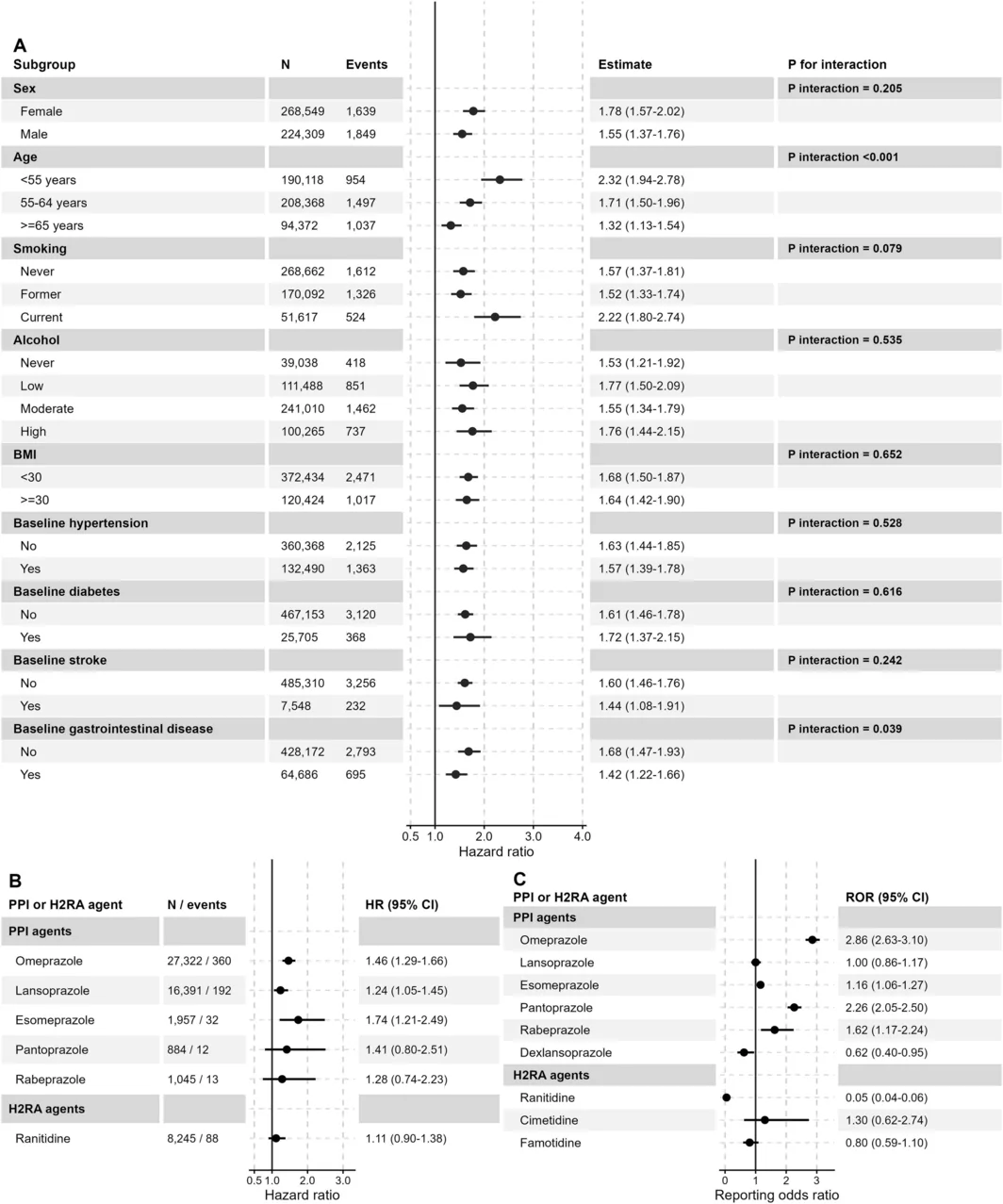
Subgroup and agent‐specific analyses. (A) UK Biobank age‐ and sex‐stratified Cox models adjusted for Model 2 covariates, with the stratifying variable omitted from the corresponding adjustment set. Interaction *p* values were obtained by likelihood‐ratio tests. (B) UK Biobank analyses by individual PPI and H2RA agent; estimates were omitted when event counts were insufficient. (C) FAERS disproportionality by individual PPI and H2RA agent. HRs and RORs are distinct measures and were not compared as equivalent effect estimates.

**FIGURE 4:**
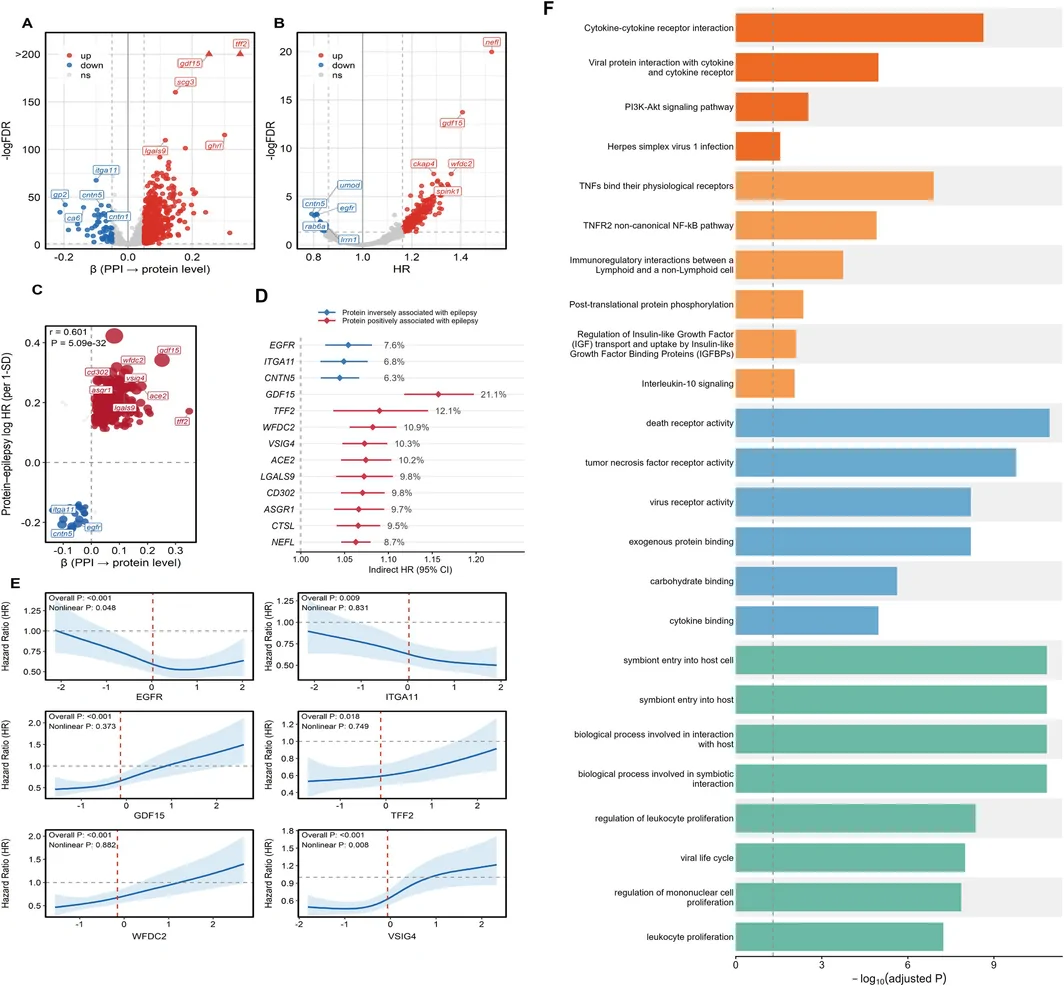
Plasma proteomic signatures linking PPI use and incident epilepsy. (A) Volcano plot of PPI–protein associations. (B) Volcano plot of protein–epilepsy associations. (C) Relationship between the two association coefficients, with bridging proteins highlighted. (D) Candidate mediator proteins ranked by estimated proportion mediated. Colors in panels C and D denote whether protein levels were positively or inversely associated with epilepsy; they do not indicate harmful or protective causal pathways. (E) Restricted cubic spline analyses for representative proteins. Evidence of nonlinearity was present for EGFR and VSIG4, but not for the other displayed proteins. (F) Enrichment analysis of the 85 candidate mediator proteins. Protein–epilepsy and mediation models used Model 2 covariates: Age, sex, body mass index, Townsend deprivation index, smoking status, alcohol intake, ethnicity, and education. Mediation analyses used protein‐specific complete cases; missing protein measurements were not imputed. Mediation estimates are observational and exploratory.

**FIGURE 5:**
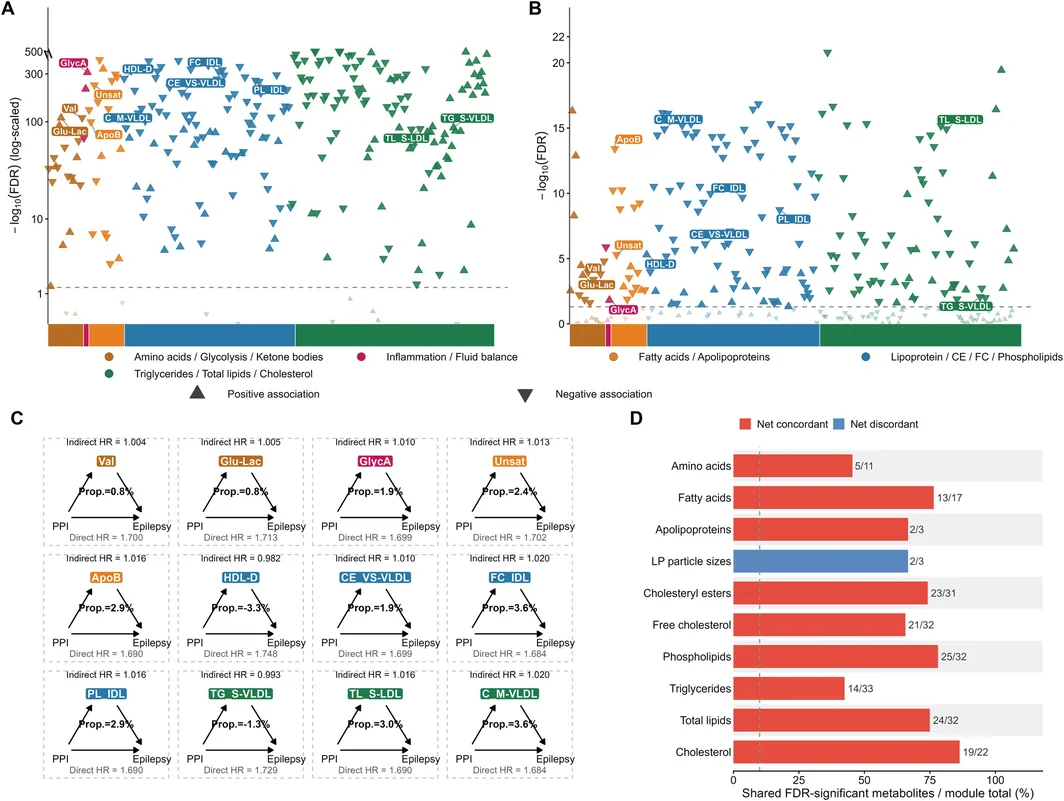
Metabolomic signatures linking PPI use and incident epilepsy. (A) Manhattan plot of PPI‐metabolite associations. The *y*‐axis uses a log10 transformation of −log10(FDR); values above the displayed limit are truncated at the upper boundary and marked by the *y*‐axis break. (B) Manhattan plot of metabolite‐epilepsy associations. Upward and downward triangles in panels A and B indicate positive and negative standardized coefficients, respectively; all 251 metabolites are displayed, and the dashed line marks FDR = 0.05. (C) Mediation diagrams for 12 module‐representative metabolites. Indirect and direct effects are shown as exponentiated HRs. Estimates used metabolite‐specific complete cases from a fixed simple random sample of 100,000 participants drawn without replacement using a prespecified random seed, with percentile 95% CIs from 200 nonparametric bootstrap samples. (D) The 176 shared FDR‐significant metabolites grouped into ten prespecified biochemical modules. Bar length indicates the proportion of metabolites in each module that were significant in both analyses, and the number beside each bar gives the shared count and module total. Red indicates that the PPI‐related metabolite differences were, on average, aligned with the corresponding metabolite associations with epilepsy; blue indicates an overall opposite direction. Metabolite models used Model 2 covariates. FDR, false discovery rate; HR, hazard ratio; NMR, nuclear magnetic resonance; PPI, proton pump inhibitor.

## 3 Results

### 3.1 Study Population

The final analytical cohort comprised 492,858 participants, including 49,451 PPI users (10.0%) and 443,407 non‐users. The study flow is summarized in Figure 1. Compared with non‐users, PPI users were older, had higher BMI, were less likely to have a university degree, and had a substantially greater burden of baseline gastrointestinal disease and cardiometabolic comorbidity (Table 1). Over a median follow‐up of 13.2 years, 3488 incident epilepsy events were recorded.

Among PPI users, omeprazole and lansoprazole were the most commonly reported agents, accounting for the majority of PPI exposure in the cohort.

### 3.2 PPI Use and Incident Epilepsy: Primary and Sensitivity Analyses

In the fully adjusted primary model (Model 3), baseline PPI use was associated with a 43% higher risk of incident epilepsy than non‐use (HR 1.43, 95% CI 1.29–1.59; *p* < 0.001; Figure 2C). Sequential adjustment attenuated the estimate from Model 1 [HR 1.86 (95% CI 1.70–2.03)] to Model 2 [1.66 (1.52–1.81)] and Model 3, indicating partial confounding by demographic, lifestyle, and comorbidity factors. The Schoenfeld results are reported in Table S2. Cumulative incidence remained higher among PPI users during follow‐up (Figure 2A).

The active‐comparator analysis included 55,014 mutually exclusive PPI or H2RA users and remained positive after full adjustment (HR 1.36, 95% CI 1.05–1.76; *p* = 0.018; Figure 2C). Propensity score matching that included baseline acid‐related gastrointestinal disease retained 7086 pairs. All displayed covariates had absolute standardized mean differences < 0.10 after matching (Table S3A,B). During follow‐up, 92 events occurred among matched PPI users and 66 among matched H2RA users. The matched HR was 1.41 (95% CI 1.03–1.94; *p* = 0.030; Table S3C). Fine–Gray analyses accounting for death were consistent with the primary findings (Table S4).

Sensitivity analyses were consistent with the primary result. Estimates were similar after one‐year landmarking [HR 1.43 (95% CI 1.29–1.59)], 2‐year landmarking [1.43 (1.28–1.60)], exclusion of baseline ASM users [1.38 (1.23–1.54)], and restriction to G40 [1.45 (1.31–1.61)]. After baseline exclusion and time‐updated censoring, the HRs were 1.48 (1.31–1.67) for stroke, 1.45 (1.30–1.61) for brain/CNS tumor, 1.45 (1.31–1.61) for traumatic brain injury, 1.40 (1.26–1.55) for CNS infection, and 1.44 (1.29–1.60) for dementia. The five‐condition joint analysis retained 483,855 participants and 2381 epilepsy events and yielded HR 1.47 (1.29–1.67; *p* < 0.001). The age‐stratified and time‐varying‐age models both yielded HR 1.43 (1.29–1.59; Figure S1A,B and Table S14A,E).

### 3.3 FAERS Pharmacovigilance Signal and UK Biobank Subgroup Analyses

FAERS provided an independent pharmacovigilance signal for PPI‐epilepsy reporting. Among 17,110,199 reports retained after exclusions, PPI primary‐suspect reports (*n* = 161,317) showed higher reporting of epilepsy‐related events than chronic non‐gastrointestinal control medication reports (*n* = 1,135,014) (overall ROR 1.70, 95% CI 1.61–1.79; PRR 1.69, 95% CI 1.60–1.78; Figure 2D). Event‐subtype analyses showed positive reporting signals for epilepsy, seizure, convulsion, and status epilepticus.

Age‐stratified analyses showed stronger relative associations at younger baseline ages: HR 2.32 (95% CI 1.94–2.78) for participants aged < 55 years, 1.71 (1.50–1.96) for those aged 55–64 years, and 1.32 (1.13–1.54) for those aged ≥ 65 years (*p* for interaction < 0.001; Figure 3A and Table S14B). The association was present in women and men, with no clear evidence of interaction by sex (*p* = 0.205). Among 3488 incident cases, the median age at the first post‐baseline G40/G41 record was 67.86 years (IQR 60.33–73.13); the corresponding medians were 67.45 years in women and 68.19 years in men. Agent‐specific UK Biobank estimates were positive for omeprazole, lansoprazole, and esomeprazole. There was no clear evidence of an association between ranitidine use and incident epilepsy (HR 1.11, 95% CI 0.90–1.38), and other H2RAs had too few events for stable estimates (Figure 3B). FAERS agent‐specific analyses included both PPI and H2RA agents (Figure 3C).

### 3.4 Proteomic Signatures Linking PPI Use and Epilepsy Risk

In the proteomic sub‐cohort (*N* = 52,029; 422 events), PPI use was associated with 1554 proteins at FDR < 0.05, including 1262 positive and 292 negative associations (Figure 4A). Incident epilepsy was associated with 340 proteins at FDR < 0.05 (Figure 4B). The two analyses shared 312 bridging proteins, among which the PPI–protein and protein–epilepsy coefficients were positively correlated (Pearson *r* = 0.601; *p* < 0.001; Figure 4C and Table S7).

Exploratory complete‐case mediation analysis identified 85 candidate mediator proteins with an estimated proportion mediated > 5% and FDR < 0.05 for the indirect effect. The largest estimates were observed for GDF15 (21.1%), TFF2 (12.1%), WFDC2 (10.9%), VSIG4 (10.3%), ACE2 (10.2%), LGALS9 (9.8%), and ASGR1 (9.7%; Figure 4D). Complete results, including protein‐specific sample sizes and event counts, are provided in Tables S12 and S13.

Restricted cubic spline analyses provided evidence of nonlinearity for EGFR and VSIG4, but not for the other displayed proteins (Figure 4E). Enrichment of the 85 candidate mediator proteins highlighted immune and inflammatory processes, including cytokine‐receptor interactions, PI3K‐Akt signaling, cell adhesion, TNF/NF‐kB‐related pathways, leukocyte proliferation, and extracellular‐matrix terms (Figure 4F and Tables S8–S11). STRING analysis identified several connected communities after graph filtering (Figure S2).

### 3.5 Metabolomic Signatures and Shared Metabolites

Among 480,315 participants with baseline NMR metabolomics, 241 of 251 metabolites were associated with baseline PPI use at FDR < 0.05 (Figure 5A; Table S15), and 181 were associated with incident epilepsy at FDR < 0.05 (Figure 5B; Table S16). All association models used standardized metabolite levels. The two sets shared 176 metabolites (Table S17), of which 126 had concordant and 50 had discordant association directions.

Twelve module‐representative metabolites were selected for exploratory mediation analyses (Figure 5C): Val, Glu‐Lac, GlycA, unsaturated fatty acid fraction, ApoB, HDL‐D, CE_VS‐VLDL, FC_IDL, PL_IDL, TG_S‐VLDL, TL_S‐LDL, and C_M‐VLDL. Individual mediated proportions were modest; percentile 95% CIs are reported in Table S18.

The 176 metabolites associated with both PPI use and incident epilepsy were grouped into ten biochemical modules (Figure 5D). Bar length shows the proportion of metabolites in each module that were significant in both analyses. Red bars indicate that the PPI‐related metabolite differences were, on average, aligned with the corresponding metabolite associations with epilepsy, whereas blue bars indicate an overall opposite direction. Nine modules showed an aligned pattern; the lipoprotein‐particle‐size module was the only module showing an opposite pattern. Module counts and denominators are reported in Table S19. Standardized levels and pairwise correlations of the 12 representative metabolites are shown in Figures S3 and S4.

## 4 Discussion

In this prospective UK Biobank analysis, baseline PPI use was associated with a higher rate of incident epilepsy. The estimate remained positive across active‐comparator matching, competing‐risk models, age and sex analyses, alternative outcome definitions, GP prescription confirmation, and neurological‐condition sensitivity analyses. FAERS showed a corresponding reporting signal, while the proteomic and metabolomic analyses identified inflammatory and metabolic correlates.

Age‐stratified analyses showed positive associations in all three age groups, with a stronger relative association at younger baseline ages. Cox models using 5‐year age strata or a time‐varying age effect both returned an HR of 1.43, consistent with the primary estimate. Among incident cases, the median age at the first qualifying post‐baseline G40/G41 record was 67.86 years. This value describes age at the first recorded diagnosis during UK Biobank follow‐up.

H2RAs were selected as an active comparator because they share major acid‐related clinical indications with PPIs [24, 25], which reduces indication differences relative to comparison with untreated participants. The matched analysis remained consistent with the primary estimate.

FAERS provided an external pharmacovigilance layer distinct from the cohort analysis. The positive signals across epilepsy, seizure, convulsion, and status epilepticus supported consistency at the reporting‐signal level [29, 30, 31, 32].

The proteomic findings provide an inflammatory context. The candidate mediators included proteins related to cellular stress responses, gastric mucosal biology, immune signaling, and neuroaxonal injury [36, 39, 40, 41, 42, 43, 44]. The protein‐interaction network provided a descriptive summary of connectivity among the FDR‐significant candidate mediator proteins.

Metabolomics provided complementary evidence from a distinct molecular layer. PPI‐associated NMR metabolite differences overlapped with epilepsy‐associated metabolites across triglyceride‐rich lipoproteins, fatty‐acid composition, amino‐acid and ketone‐body pathways, and GlycA, a marker of systemic low‐grade inflammation [37, 38, 45, 46]. The distributed pattern across modules indicates a broad systemic inflammatory and metabolic milieu rather than a single dominant metabolite.

Clinically, these findings support judicious PPI use rather than indiscriminate discontinuation. PPIs remain appropriate for established indications, but unnecessary long‐term use should be reassessed, particularly in patients with additional neurological or metabolic risk factors [7, 8, 9].

Several limitations should be considered. First, PPI use was measured at baseline; information on dose, duration, adherence, over‐the‐counter use, and treatment changes was unavailable. Second, although H2RAs share major indications with PPIs, the two drug classes differ in acid‐suppressive potency and prescribing patterns. Residual confounding by gastrointestinal disease severity, healthcare utilization, endoscopic findings, treatment escalation, and prescribing preference may therefore remain, and the active‐comparator analysis should be interpreted as supportive rather than causal evidence. Third, incident epilepsy was primarily identified from first‐occurrence G40/G41 records. A subsequent ASM prescription provided an additional recording criterion but did not constitute clinical adjudication, and this analysis was limited to participants with eligible GP coverage. Individual epilepsy etiology was unavailable. Excluding baseline neurological disease and censoring follow‐up when stroke, brain/CNS tumor, traumatic brain injury, CNS infection, or dementia preceded epilepsy yielded similar estimates, both separately and jointly, but could not eliminate all alternative explanations. Fourth, proteomic and metabolomic measurements were obtained once at baseline, and the mediation analyses were observational; neither the molecular associations nor the protein‐interaction network establish causal mechanisms. Finally, FAERS is subject to voluntary and selective reporting, lacks population denominators, and cannot estimate incidence or causality.

## 5 Conclusion

In the UK Biobank, baseline PPI use was associated with a higher rate of incident epilepsy, and the association was consistent across active‐comparator, outcome‐confirmation, competing‐risk, subgroup, and neurological‐condition sensitivity analyses. FAERS and baseline proteomic and metabolomic analyses provided complementary pharmacovigilance and molecular context. Together, these findings support regular review of long‐term PPI indications and further investigation of the observed association.

## Author Contributions

Minghao Li and Yiqian Li: Conceptualization, formal analysis, and writing – original draft. Yuxi Zhang, Balkissou Seebo Saibou, Jiayi Xing, Chao Jin, Yifei Chen, and Yuchen Xu: data curation, methodology, visualization, and writing – review and editing. Jiahe Lin and Xinshi Wang: supervision, funding acquisition, and writing – review and editing. All authors read and approved the final manuscript.

## Funding

This study was supported by the National Natural Science Foundation of China (82671890).

## Ethics Statement

UK Biobank has ethical approval from the North West Multi‐center Research Ethics Committee (REC reference: 16/NW/0274). All participants provided written informed consent. This research was conducted under UK Biobank Application Number 217802. FAERS data are publicly available de‐identified reports and do not require additional ethical approval. The study was conducted in accordance with the Declaration of Helsinki.

## Conflicts of Interest

The authors declare no conflicts of interest.

## Supporting information


**Table S1:** Variable definitions—exposure, outcome, covariates, and medications (S1A‐S1E).
**Table S2:** Proportional hazards assumption—Schoenfeld residuals test.
**Table S3:** Propensity score matched analysis—covariate balance and Cox results (S3A–S3C).
**Table S4:** Fine‐Gray subdistribution hazard model—competing risks analysis.
**Table S5:** FAERS drug and exclusion definitions (S5A–S5C).
**Table S6:** FAERS disproportionality analysis—complete results.
**Table S7:** PPI‐associated proteins—complete list (312 bridge proteins).
**Table S8:** Gene Ontology Biological Process enrichment analysis.
**Table S9:** Gene Ontology Molecular Function enrichment analysis.
**Table S10:** Reactome pathway enrichment analysis.
**Table S11:** KEGG pathway enrichment analysis.
**Table S12:** Candidate mediator proteins—complete list (85 proteins, PM > 5%).
**Table S13:** Proteins associated with incident epilepsy—Cox regression.
**Table S14:** Additional epidemiological analyses introduced during revision (S14A‐S14F).
**Table S15:** Significant associations between PPI use and baseline NMR metabolites.
**Table S16:** Significant associations between baseline NMR metabolites and incident epilepsy.
**Table S17:** Shared metabolites passing FDR < 0.05 in both association analyses.
**Table S18:** Mediation analysis for 12 metabolites.
**Table S19:** Shared metabolite distribution by biochemical module.
**Figure S1:** Robustness analyses and propensity‐score diagnostics.
**Figure S2:** Protein–protein interaction network of candidate mediator proteins.
**Figure S3:** Standardized plasma levels of representative NMR metabolites.
**Figure S4:** Spearman correlation matrix of representative NMR metabolites.

## Data Availability

UK Biobank data are available to approved researchers through the UK Biobank Access Management System. FAERS data are publicly available through the U.S. Food and Drug Administration. Processed analysis code and derived tables will be made available in a public repository upon acceptance for publication.

## References

[1] GBD 2016 Epilepsy Collaborators , “Global, Regional, and National Burden of Epilepsy, 1990–2016: A Systematic Analysis for the Global Burden of Disease Study 2016,” Lancet Neurology 18 (2019): 357–375. 10.1016/S1474-4422(18)30454-X PMC6416168 30773428

[2] J. D. Steinmetz , K. M. Seeher , N. Schiess , et al., “Global, Regional, and National Burden of Disorders Affecting the Nervous System, 1990–2021: A Systematic Analysis for the Global Burden of Disease Study 2021,” Lancet Neurology 23 (2024): 344–381. 10.1016/S1474-4422(24)00038-3 PMC10949203 38493795

[3] M. R. Keezer , S. M. Sisodiya , and J. W. Sander , “Comorbidities of Epilepsy: Current Concepts and Future Perspectives,” Lancet Neurology 15 (2016): 106–115. 10.1016/S1474-4422(15)00225-2 26549780

[4] R. S. Fisher , C. Acevedo , A. Arzimanoglou , et al., “ILAE Official Report: A Practical Clinical Definition of Epilepsy,” Epilepsia 55 (2014): 475–482. 10.1111/epi.12550 24730690

[5] R. S. Fisher , J. H. Cross , C. D'Souza , et al., “Instruction Manual for the ilae 2017 Operational Classification of Seizure Types,” Epilepsia 58 (2017): 531–542. 10.1111/epi.13671 28276064

[6] E. D. Kantor , C. D. Rehm , J. S. Haas , A. T. Chan , and E. L. Giovannucci , “Trends in Prescription Drug Use Among Adults in the United States From 1999–2012,” JAMA 314 (2015): 1818–1831. 10.1001/jama.2015.13766 PMC4752169 26529160

[7] D. E. Freedberg , L. S. Kim , and Y.‐X. Yang , “The Risks and Benefits of Long‐Term Use of Proton Pump Inhibitors: Expert Review and Best Practice Advice From the American Gastroenterological Association,” Gastroenterology 152 (2017): 706–715. 10.1053/j.gastro.2017.01.031 28257716

[8] R. Yadlapati and P. J. Kahrilas , “The “Dangers” of Chronic Proton Pump Inhibitor Use,” Journal of Allergy and Clinical Immunology 141 (2018): 79–81. 10.1016/j.jaci.2017.06.017 28729001

[9] I. Forgacs and A. Loganayagam , “Overprescribing Proton Pump Inhibitors,” BMJ 336 (2008): 2–3. 10.1136/bmj.39406.449456.BE PMC2174763 18174564

[10] B. Lazarus , Y. Chen , F. P. Wilson , et al., “Proton Pump Inhibitor Use and the Risk of Chronic Kidney Disease,” JAMA Internal Medicine 176 (2016): 238–246. 10.1001/jamainternmed.2015.7193 PMC4772730 26752337

[11] Y. Xie , B. Bowe , Y. Yan , H. Xian , T. Li , and Z. al‐Aly , “Estimates of All Cause Mortality and Cause Specific Mortality Associated With Proton Pump Inhibitors Among US Veterans: Cohort Study,” BMJ 365 (2019): l1580, 10.1136/bmj.l1580. PMC6538974 31147311

[12] C. H. Park , E. H. Kim , Y. H. Roh , H. Y. Kim , and S. K. Lee , “The Association Between the Use of Proton Pump Inhibitors and the Risk of Hypomagnesemia: A Systematic Review and Meta‐Analysis,” PLoS One 9 (2014): e112558. 10.1371/journal.pone.0112558 PMC4230950 25394217

[13] H. Falhammar , J. D. Lindh , J. Calissendorff , J. Skov , D. Nathanson , and B. Mannheimer , “Associations of Proton Pump Inhibitors and Hospitalization due to Hyponatremia: A Population–Based Case–Control Study,” European Journal of Internal Medicine 59 (2019): 65–69. 10.1016/j.ejim.2018.08.012 30154038

[14] R. Nardone , F. Brigo , and E. Trinka , “Acute Symptomatic Seizures Caused by Electrolyte Disturbances,” Journal of Clinical Neurology 12 (2016): 21–33. 10.3988/jcn.2016.12.1.21 PMC4712283 26754778

[15] D. J. Colacurcio and R. A. Nixon , “Disorders of Lysosomal Acidification—The Emerging Role of v‐ATPase in Aging and Neurodegenerative Disease,” Ageing Research Reviews 32 (2016): 75–88. 10.1016/j.arr.2016.05.004 PMC5112157 27197071

[16] F. Imhann , M. J. Bonder , A. Vich Vila , et al., “Proton Pump Inhibitors Affect the Gut Microbiome,” Gut 65 (2016): 740–748. 10.1136/gutjnl-2015-310376 PMC4853569 26657899

[17] C. A. Olson , H. E. Vuong , J. M. Yano , Q. Y. Liang , D. J. Nusbaum , and E. Y. Hsiao , “The Gut Microbiota Mediates the Anti‐Seizure Effects of the Ketogenic Diet,” Cell 173 (2018): 1728–1741.e13. 10.1016/j.cell.2018.04.027 PMC6003870 29804833

[18] P. E. Lazzerini , A. Cartocci , Y. S. Qu , et al., “Proton Pump Inhibitors Directly Block hERG‐Potassium Channel and Independently Increase the Risk of QTc Prolongation in a Large Cohort of US Veterans,” Circulation. Arrhythmia and Electrophysiology 14 (2021): e010042. 10.1161/CIRCEP.121.010042 34143643

[19] C.‐S. Liang , Y. M. Bai , J. W. Hsu , et al., “The Risk of Epilepsy After Long‐Term Proton Pump Inhibitor Therapy,” Seizure 87 (2021): 88–93. 10.1016/j.seizure.2021.03.008 33735722

[20] M. E. Sáez , A. González‐Pérez , D. Gaist , S. Johansson , P. Nagy , and L. A. García Rodríguez , “Risk of Seizure Associated With Use of Acid‐Suppressive Drugs: An Observational Cohort Study,” Epilepsy & Behavior 62 (2016): 72–80. 10.1016/j.yebeh.2016.06.039 27450309

[21] U. Gudnadottir , R. Wickstrom , A. Gunnerbeck , S. Prast‐Nielsen , and N. Brusselaers , “Prenatal and Early Childhood Exposure to Antibiotics or Gastric Acid Inhibitors and Increased Risk of Epilepsy: A Nationwide Population‐Based Study,” Clinical Pharmacology and Therapeutics 118 (2025): 206–217. 10.1002/cpt.3679 PMC12166261 40231335

[22] C. Bycroft , C. Freeman , D. Petkova , et al., “The UK Biobank Resource With Deep Phenotyping and Genomic Data,” Nature 562 (2018): 203–209. 10.1038/s41586-018-0579-z PMC6786975 30305743

[23] C. Sudlow , J. Gallacher , N. Allen , et al., “UK Biobank: An Open Access Resource for Identifying the Causes of a Wide Range of Complex Diseases of Middle and Old Age,” PLoS Medicine 12 (2015): e1001779. 10.1371/journal.pmed.1001779 PMC4380465 25826379

[24] J. L. Lund , D. B. Richardson , and T. Stürmer , “The Active Comparator, New User Study Design in Pharmacoepidemiology: Historical Foundations and Contemporary Application,” Current Epidemiology Reports 2 (2015): 221–228. 10.1007/s40471-015-0053-5 PMC4778958 26954351

[25] R. Sendor and T. Stürmer , “Core Concepts in Pharmacoepidemiology: Confounding by Indication and the Role of Active Comparators,” Pharmacoepidemiology and Drug Safety 31 (2022): 261–269. 10.1002/pds.5407 PMC9121653 35019190

[26] P. C. Austin , “An Introduction to Propensity Score Methods for Reducing the Effects of Confounding in Observational Studies,” Multivariate Behavioral Research 46 (2011): 399–424. 10.1080/00273171.2011.568786 PMC3144483 21818162

[27] J. P. Fine and R. J. Gray , “A Proportional Hazards Model for the Subdistribution of a Competing Risk,” Journal of the American Statistical Association 94 (1999): 496–509. 10.1080/01621459.2017.1401540 PMC6502476 31073256

[28] P. C. Austin and J. P. Fine , “Practical Recommendations for Reporting Fine–Gray Model Analyses for Competing Risk Data,” Statistics in Medicine 36 (2017): 4391–4400. 10.1002/sim.7501 PMC5698744 28913837

[29] U.S. Food and Drug Administration , “FDA Adverse Event Reporting System (FAERS) Public Dashboard,” (2025), https://www.fda.gov/drugs/questions‐and‐answers‐fdas‐adverse‐event‐reporting‐system‐faers.

[30] E. P. Van Puijenbroek , A. Bate , H. G. M. Leufkens , et al., “A Comparison of Measures of Disproportionality for Signal Detection in Spontaneous Reporting Systems for Adverse Drug Reactions,” Pharmacoepidemiology and Drug Safety 11 (2002): 3–10. 10.1002/pds.668 11998548

[31] A. Bate and S. J. W. Evans , “Quantitative Signal Detection Using Spontaneous ADR Reporting,” Pharmacoepidemiology and Drug Safety 18 (2009): 427–436. 10.1002/pds.1742 19358225

[32] K. J. Rothman , S. Lanes , and S. T. Sacks , “The Reporting Odds Ratio and Its Advantages Over the Proportional Reporting Ratio,” Pharmacoepidemiology and Drug Safety 13 (2004): 519–523. 10.1002/pds.1001 15317031

[33] B. B. Sun , J. Chiou , M. Traylor , et al., “Plasma Proteomic Associations With Genetics and Health in the UK Biobank,” Nature 622 (2023): 329–338. 10.1038/s41586-023-06592-6 PMC10567551 37794186

[34] D. Tingley , T. Yamamoto , K. Hirose , L. Keele , and K. Imai , “Mediation: R Package for Causal Mediation Analysis,” Journal of Statistical Software 59 (2014): 1–38.

[35] G. Yu , L.‐G. Wang , Y. Han , and Q.‐Y. He , “clusterProfiler: An R Package for Comparing Biological Themes Among Gene Clusters,” OMICS: A Journal of Integrative Biology 16 (2012): 284–287. 10.1089/omi.2011.0118 PMC3339379 22455463

[36] D. Szklarczyk , R. Kirsch , M. Koutrouli , et al., “The STRING Database in 2023: Protein–Protein Association Networks and Functional Enrichment Analyses for Any Sequenced Genome of Interest,” Nucleic Acids Research 51 (2023): D638–D646. 10.1093/nar/gkac1000 PMC9825434 36370105

[37] T. Buergel , J. Steinfeldt , G. Ruyoga , et al., “Metabolomic Profiles Predict Individual Multidisease Outcomes,” Nature Medicine 28 (2022): 2309–2320. 10.1038/s41591-022-01980-3 PMC9671812 36138150

[38] P. Soininen , A. J. Kangas , P. Würtz , T. Suna , and M. Ala‐Korpela , “Quantitative Serum Nuclear Magnetic Resonance Metabolomics in Cardiovascular Epidemiology and Genetics,” Circulation. Cardiovascular Genetics 8 (2015): 192–206. 10.1161/CIRCGENETICS.114.000216 25691689

[39] T. Wu , E. Hu , S. Xu , et al., “clusterProfiler 4.0: A Universal Enrichment Tool for Interpreting Omics Data,” Innovation (Cambridge, Mass.) 2 (2021): 100141. 10.1016/j.xinn.2021.100141 PMC8454663 34557778

[40] A. Vezzani , J. French , T. Bartfai , and T. Z. Baram , “The Role of Inflammation in Epilepsy,” Nature Reviews. Neurology 7 (2011): 31–40. 10.1038/nrneurol.2010.178 PMC3378051 21135885

[41] A. Vezzani , S. Balosso , and T. Ravizza , “Neuroinflammatory Pathways as Treatment Targets and Biomarkers in Epilepsy,” Nature Reviews. Neurology 15 (2019): 459–472. 10.1038/s41582-019-0217-x 31263255

[42] J. Wischhusen , I. Melero , and W. H. Fridman , “Growth/Differentiation Factor‐15 (GDF‐15): From Biomarker to Novel Targetable Immune Checkpoint,” Frontiers in Immunology 11 (2020): 951. 10.3389/fimmu.2020.00951 PMC7248355 32508832

[43] W. Hoffmann , “Trefoil Factor Family (TFF) Peptides,” Encyclopedia 1 (2021): 974–987.

[44] M. Khalil , C. E. Teunissen , M. Otto , et al., “Neurofilaments as Biomarkers in Neurological Disorders,” Nature Reviews. Neurology 14 (2018): 577–589. 10.1038/s41582-018-0058-z 30171200

[45] J. D. Otvos , I. Shalaurova , J. Wolak‐Dinsmore , et al., “GlycA: A Composite Nuclear Magnetic Resonance Biomarker of Systemic Inflammation,” Clinical Chemistry 61 (2015): 714–723. 10.1373/clinchem.2014.232918 25779987

[46] P. Würtz , A. S. Havulinna , P. Soininen , et al., “Metabolite Profiling and Cardiovascular Event Risk: A Prospective Study of 3 Population‐Based Cohorts,” Circulation 131 (2015): 774–785. 10.1161/CIRCULATIONAHA.114.013116 PMC4351161 25573147

