# Supplementary material for: Proton Pump Inhibitor Use and Incident Epilepsy in the UK Biobank: Evidence From Pharmacovigilance and Multi‐Omics Analyses
Source: CNS Neurosci Ther. 2026 Nov 4;32(11):e71223. doi: 10.1002/cns.71223 (PMC13732812; doi:10.1002/cns.71223)
Supplement: Supplementary file 1 [file CNS-32-e71223-s001.docx]

**Supplementary Appendix**

**Proton Pump Inhibitor Use and Incident Epilepsy in the UK Biobank: Evidence from Pharmacovigilance and Multi-Omics Analyses**

**Contents**

- **Table S1. Variable definitions - exposure, outcome, covariates, and medications (S1A-S1E)**
- **Table S2. Proportional hazards assumption - Schoenfeld residuals test**
- **Table S3. Propensity score matched analysis - covariate balance and Cox results (S3A-S3C)**
- **Table S4. Fine-Gray subdistribution hazard model - competing risks analysis**
- **Table S5. FAERS drug and exclusion definitions (S5A-S5C)**
- **Table S6. FAERS disproportionality analysis - complete results**
- **Table S7. PPI-associated proteins - complete list (312 bridge proteins)**
- **Table S8. Gene Ontology Biological Process enrichment analysis**
- **Table S9. Gene Ontology Molecular Function enrichment analysis**
- **Table S10. Reactome pathway enrichment analysis**
- **Table S11. KEGG pathway enrichment analysis**
- **Table S12. Candidate mediator proteins - complete list (85 proteins, PM>5%)**
- **Table S13. Proteins associated with incident epilepsy - Cox regression**
- **Table S14. Additional epidemiological analyses introduced during revision (S14A-S14F)**
- **Table S15. Significant associations between PPI use and baseline NMR metabolites**
- **Table S16. Significant associations between baseline NMR metabolites and incident epilepsy**
- **Table S17. Shared metabolites passing FDR < 0.05 in both association analyses**
- **Table S18. Mediation analysis for 12 metabolites**
- **Table S19. Shared metabolite distribution by biochemical module**

**Supplementary Figure Legends**

- Supplementary Figure S1. Robustness analyses and propensity-score diagnostics
- Supplementary Figure S2. Protein-protein interaction network of candidate mediator proteins
- Supplementary Figure S3. Standardised plasma levels of representative NMR metabolites
- Supplementary Figure S4. Spearman correlation matrix of representative NMR metabolites

**Supplementary Table S1. Variable definitions — exposure, outcome, covariates, and medications**

S1A: core analytic definitions and covariate recoding. S1B: PPI medication codes (UKB Field 20003). S1C: H2RA active comparator codes (UKB Field 20003). S1D: ASM codes for baseline exclusion. S1E: maintenance ASM definitions used for GP prescription-supported outcome confirmation.

**Table S1A. Core analytic definitions and covariate recoding**

| **Domain** | **Variable** | **Source / Field** | **Definition / Recoding** |
| --- | --- | --- | --- |
| Exposure | Baseline PPI use | UKB Field 20003 | PPI user if any PPI medication code was recorded at baseline assessment |
| Outcome | Incident epilepsy | ICD-10 G40/G41 | First post-baseline record of ICD-10 G40 or G41 in HES, SMR, or death records |
| Time origin | Baseline assessment date | UKB Field 53 | Date of attending assessment centre at baseline; used as time zero; participants with prevalent epilepsy excluded |
| Censoring | Administrative censoring | Study-defined censoring date | Earliest of incident epilepsy, death, or 2022-05-31 |
| Competing risk | All-cause death | UKB death registry linkage | Used as the competing event in Fine-Gray competing-risk models |
| Covariate | Age | UKB Field 21003 | Age at recruitment; included as a continuous variable (years) |
| Covariate | Sex | UKB Field 31 | Female / Male |
| Covariate | Body mass index | UKB Field 21001 | Included as a continuous variable (kg/m²) |
| Covariate | Townsend deprivation index | UKB Field 22189 | Included as a continuous variable; higher values indicate greater deprivation |
| Covariate | Smoking status | UKB Field 20116 | 4-category variable (smoking4): Never (UKB raw value: “Never”) / Former (raw: “Previous”) / Current (raw: “Current”) / Missing (raw: “Prefer not to answer”, empty string, or NA) |
| Covariate | Alcohol intake | UKB Field 1558 | 5-category variable (alcohol5): Never (raw: “Never”) / Low (raw: “Special occasions only” or “One to three times a month”) / Moderate (raw: “Once or twice a week” or “Three or four times a week”) / High (raw: “Daily or almost daily”) / Missing (raw: “Prefer not to answer”, empty string, or NA) |
| Covariate | Ethnicity | UKB Field 21000 | 5-category variable (ethnicity5): White (raw: British, Irish, Any other white background, White) / Asian (raw: Indian, Pakistani, Chinese, Bangladeshi, Any other Asian background, Asian or Asian British) / Black (raw: Caribbean, African, Any other Black background, Black or Black British) / Mixed (raw: White and Asian, White and Black Caribbean, White and Black African, Any other mixed background, Mixed) / Other/Unknown (raw: Other ethnic group, Prefer not to answer, Do not know, empty string, or NA) |
| Covariate | Education | UKB Field 6138 | 4-category variable (education4): Degree (raw string contains “College or University degree”) / None (raw: “None of the above” exactly) / Missing (raw: “Prefer not to answer”, empty string, or NA) / Below degree (all remaining valid combinations, e.g. A levels/AS levels, O levels/GCSEs, CSEs, NVQ/HND/HNC, other professional qualifications) |
| Baseline disease | Baseline gastrointestinal disease | ICD-10 K20, K21, K25-K27, K29 | Diagnosed before baseline assessment |
| Baseline disease | Hypertension | ICD-10 I10–I13 | Diagnosed before baseline assessment |
| Baseline disease | Diabetes | ICD-10 E10–E14 | Diagnosed before baseline assessment |
| Baseline disease | Stroke | ICD-10 I60–I64 | Diagnosed before baseline assessment |

**Table S1B. PPI medication codes (UKB Field 20003)**

| **Mapped ingredient** | **UKB medication text** | **UKB code** |
| --- | --- | --- |
| Esomeprazole | esomeprazole; nexium 20mg tablet | 1141177526; 1141177532 |
| Lansoprazole | lansoprazole; zoton 15mg capsule | 1140864752; 1140923688 |
| Omeprazole | omeprazole; losec 10mg capsule | 1140865634; 1140909578 |
| Pantoprazole | pantoprazole; protium 20mg e/c tablet | 1140929012; 1141164616 |
| Rabeprazole | rabeprazole sodium; pariet 10mg e/c tablet | 1141168584; 1141168590 |

**Table S1C. H2RA active comparator codes (UKB Field 20003)**

| **Mapped ingredient** | **UKB medication text** | **UKB code** |
| --- | --- | --- |
| Cimetidine | cimetidine; tagamet 100 tablet | 1140865426; 1140909500 |
| Famotidine | famotidine; pepcid ac indigestion tablet | 1140865608; 1140909496 |
| Nizatidine | nizatidine; axid 150mg capsule | 1140865618; 1140865758 |
| Ranitidine | ranitidine; zantac 75 tablet | 1140879406; 1140916980 |

**Table S1D. Antiseizure medication (ASM) codes for baseline exclusion (UKB Field 20003)**

| **ASM ingredient** | **Representative UKB text** | **UKB codes** |
| --- | --- | --- |
| Carbamazepine | carbamazepine / tegretol 100mg | 2038459704; 1140872064; 1140872072 |
| Clobazam | clobazam / frisium 10mg | 1140863268; 1140863272 |
| Clonazepam | clonazepam / rivotril 500mcg | 1140872150; 1140872152 |
| Diazepam | diazepam / valium | 1140863152; 1140863244; 1140855856 |
| Ethosuximide | ethosuximide / emeside / zarontin | 1140872160; 1140872162; 1140872164 |
| Gabapentin | gabapentin / neurontin | 1140872228; 1140872236 |
| Lamotrigine | lamotrigine / lamictal | 1140872290; 1140872302 |
| Levetiracetam | levetiracetam / keppra | 1141171932; 1141171940 |
| Oxcarbazepine | oxcarbazepine / trileptal | 1141175204; 1141175212 |
| Phenobarbital | phenobarbitone | 2038460068; 1140872186 |
| Phenytoin | phenytoin / epanutin | 2038460076; 1140872098; 1140872112 |
| Pregabalin | pregabalin / lyrica | 1141200004; 1141200072 |
| Primidone | primidone / mysoline | 1140872132; 1140872134 |
| Sodium valproate | sodium valproate / epilim / valproic acid | 1140872198; 1140872200; 1140872214; 1140872216; 1141172838 |
| Topiramate | topiramate / topamax | 1140923484; 1140927692 |
| Vigabatrin | vigabatrin / sabril | 2018602634; 1140872280; 1140872284 |

**Table S1E. Maintenance ASM definitions for GP prescription-supported epilepsy confirmation**

Drug names were matched case-insensitively using whole-word generic and brand terms. If the product name was missing, an unambiguous code-to-ingredient mapping derived from named prescriptions was used. Read v2 codes were compared using their first five characters. Non-ASM product names were not overridden by code matches. Injectable or infusion products, code dn78., and diazepam, midazolam, lorazepam and nitrazepam were excluded. BNF classifications were not used alone to confirm an ASM prescription. No dm+d-only matches contributed to the corrected case-prescription set. The table lists the ingredients observed, the recognition terms and the exact Read v2 codes used for missing-name records.

| **ASM ingredient** | **Generic / brand recognition terms** | **Read v2 codes used for name-missing records** | **First confirmation within 365 days, n** | **First confirmation at any subsequent time, n** |
| --- | --- | --- | --- | --- |
| Brivaracetam | brivaracetam; briviact | None used | 0 | 0 |
| Carbamazepine | carbamazepine; tegretol; carbagen; teril; timonil; epimaz | dn31.; dn32.; dn34.; dn35.; dn38.; dn3a. | 39 | 40 |
| Clobazam | clobazam; frisium; tapclob; perizam | d263. | 2 | 2 |
| Clonazepam | clonazepam; rivotril | dn4y.; dn4z. | 3 | 3 |
| Eslicarbazepine | eslicarbazepine; zebinix; aptiom | None used | 0 | 0 |
| Ethosuximide | ethosuximide; zarontin; emapax | None used | 0 | 0 |
| Gabapentin | gabapentin; neurontin | dnj1.; dnj2.; dnj3.; dnjy. | 22 | 32 |
| Lacosamide | lacosamide; vimpat | None used | 0 | 0 |
| Lamotrigine | lamotrigine; lamictal | dnf1.; dnf2.; dnf5.; dnfB. | 84 | 88 |
| Levetiracetam | levetiracetam; keppra; desitrend | dno1.; dno2.; dno4.; dnow.; dnox.; dnoy.; dnoz. | 124 | 132 |
| Oxcarbazepine | oxcarbazepine; trileptal | None used | 0 | 0 |
| Phenobarbital | phenobarbital; phenobarbitone; phenobarbit; gardenal; luminal | None used | 1 | 1 |
| Phenytoin | phenytoin; epanutin; fosphenytoin | dn95.; dn96.; dn9w.; dn9z. | 12 | 12 |
| Pregabalin | pregabalin; lyrica; alzain; axalid | dnp7.; dnps.; dnpt.; dnpu.; dnpv.; dnpw.; dnpx.; dnpy.; dnpz. | 13 | 16 |
| Primidone | primidone; mysoline | dna..; dnay. | 1 | 1 |
| Topiramate | topiramate; topamax | dnk1.; dnk2.; dnk7. | 1 | 2 |
| Valproate | valproate; valproic; divalproex; epilim; depakote; convulex; episenta; epival; depakene; orlept; valporate | dnb2.; dnb3.; dnb7.; dnb8.; dnbK.; dnbc.; dnbd.; dnbe.; dnbs.; dnbt.; dnbv.; dnby.; dnbz.; dnh7.; dnh8.; dnhy.; dnhz. | 61 | 62 |
| Vigabatrin | vigabatrin; sabril | None used | 0 | 0 |
| Zonisamide | zonisamide; zonegran | dnq1.; dnq2.; dnq3. | 4 | 4 |

GP confirmation analysis. The analytic subset comprised 175,614 participants with baseline-contiguous GP registration and available prescription records. A first post-baseline G40/G41 diagnosis had to be followed strictly later by an eligible maintenance ASM prescription before the end of observed GP follow-up. The event date was the first eligible prescription date. The primary window was 365 days; secondary definitions used 90 days or any subsequent prescription during observed follow-up. Counts in Table S1E refer to the first confirming ingredient per person within each specified window; zero does not imply absence of that drug from GP records. Models used the Model 3 covariates and were stratified by GP provider and baseline smoking-data availability. Additional screened ingredients with no accepted records were beclamide, cannabidiol, cenobamate, felbamate, methsuximide, perampanel, rufinamide, stiripentol, sulthiame, tiagabine, and trimethadione.

Abbreviations: ASM, antiseizure medication; H2RA, histamine-2 receptor antagonist; HES, Hospital Episode Statistics; ICD-10, International Classification of Diseases, Tenth Revision; PPI, proton pump inhibitor; SMR, Scottish Morbidity Record; UKB, UK Biobank.

**Supplementary Table S2. Proportional hazards assumption — Schoenfeld residuals test**

Schoenfeld residuals test for primary Cox Model 3. The global test was driven by age. Robustness analyses therefore used a Cox model stratified by 5-year age bands and a model allowing a piecewise time-varying age effect; both yielded HR 1.43 (95% CI 1.29-1.59).

| **Variable** | **Chi-squared** | **df** | **P value** | **PH assumption met** |
| --- | --- | --- | --- | --- |
| PPI use (primary exposure) | 3.513 | 1 | 0.061 | Yes |
| Age at recruitment | 28.099 | 1 | <0.001 | No |
| Sex | 3.486 | 1 | 0.062 | Yes |
| Body mass index | 0.924 | 1 | 0.337 | Yes |
| Smoking status | 0.956 | 3 | 0.812 | Yes |
| Alcohol intake | 4.656 | 4 | 0.324 | Yes |
| Ethnicity | 1.033 | 4 | 0.905 | Yes |
| Education | 3.180 | 3 | 0.365 | Yes |
| Townsend deprivation index | 3.641 | 1 | 0.056 | Yes |
| Global test (all variables) | 49.542 | 19 | <0.001 | No (driven by age) |

Abbreviations: df, degrees of freedom; PH, proportional hazards; PPI, proton pump inhibitor.
Note: The global test evaluates whether any covariate violates the PH assumption jointly. The significant global test result was driven by the violation of the PH assumption for age; all other covariates, including PPI use, satisfied the assumption individually.

**Supplementary Table S3. Propensity score-matched analyses – covariate balance and Cox results**

Table S3A reports covariate balance for PPI users versus non-users; Table S3B reports covariate balance for the PPI–H2RA active-comparator analysis; and Table S3C reports the corresponding matched Cox estimates. The active-comparator propensity score included age, sex, body mass index, Townsend deprivation index, smoking, alcohol intake, ethnicity, education, and baseline acid-related gastrointestinal disease (ICD-10 K20, K21, K25–K27, and K29).

Table S3A. Covariate balance after propensity score matching: PPI users versus non-users

| **Covariate** | **Mean (PPI users)** | **Mean (matched controls)** | **SMD** |
| --- | --- | --- | --- |
| Age (years) | 59.55 | 59.46 | 0.013 |
| Sex (proportion male) | 0.452 | 0.459 | 0.013 |
| BMI (kg/m²) | 29.13 | 28.84 | 0.057 |
| Smoking status (coded 0–3) | 1.662 | 1.672 | 0.015 |
| Alcohol intake (coded 0–4) | 2.678 | 2.703 | 0.027 |
| Townsend deprivation index | −0.915 | −0.965 | 0.015 |
| Ethnicity (proportion White) | 0.9479 | 0.9473 | 0.003 |
| Education (proportion Degree or below degree) | 0.5328 | 0.5345 | 0.003 |
| Baseline hypertension (proportion) | 0.428 | 0.397 | 0.063 |
| Baseline diabetes (proportion) | 0.103 | 0.092 | 0.036 |
| Baseline stroke (proportion) | 0.035 | 0.029 | 0.029 |

All SMD values <0.10, indicating adequate covariate balance after matching. Baseline comorbidities (hypertension, diabetes, stroke) were not matched variables but are shown to confirm post-matching balance.

Table S3B. Covariate balance after propensity score matching: PPI versus H2RA active comparator

| **Covariate** | **Before matching \|SMD\|** | **After matching \|SMD\|** | **Balance criterion** |
| --- | --- | --- | --- |
| Age | 0.252 | 0.007 | <0.10 |
| Sex | 0.008 | 0.006 | <0.10 |
| Body mass index | 0.077 | 0.014 | <0.10 |
| Townsend deprivation index | 0.047 | <0.001 | <0.10 |
| Smoking status | 0.092 | 0.024 | <0.10 |
| Alcohol intake | 0.068 | 0.016 | <0.10 |
| Ethnicity | 0.016 | 0.014 | <0.10 |
| Education | 0.120 | 0.009 | <0.10 |
| Baseline acid-related GI disease | 0.360 | 0.000 | <0.10 |

All SMD values were <0.10, indicating adequate covariate balance after matching. Baseline comorbidities were not matched variables and are not shown for the active-comparator analysis. Abbreviations: BMI, body mass index; H2RA, histamine-2 receptor antagonist; PPI, proton pump inhibitor; SMD, standardised mean difference.

Table S3C. Cox regression results after propensity score matching

| **Analysis** | **Matched pairs** | **Total N** | **Events** | **HR (95% CI)** | **P value** |
| --- | --- | --- | --- | --- | --- |
| PPI vs Non-PPI (PSM Cox, cluster-robust SE) | 49,451 | 98,902 | 1,027 | 1.71 (1.51–1.94) | <0.001 |
| PPI vs H2RA (PSM includes baseline acid-related GI disease) | 7,086 | 14,172 | 158 | 1.41 (1.03–1.94) | 0.030 |

Note: The revised PPI-H2RA propensity score incorporated baseline acid-related gastrointestinal disease (K20, K21, K25-K27, and K29). Categorical covariates are summarized by the maximum absolute SMD across encoded levels. All displayed post-matching absolute SMDs were <0.10.

**Supplementary Table S4. Fine–Gray subdistribution hazard model — competing risks analysis**

Fine–Gray subdistribution hazard model (Fine & Gray 1999) treating all-cause death as the competing risk. Covariates identical to primary Cox Model 3. Implemented using finegray() in the R survival package.

| **Analysis** | **N** | **Events (epilepsy)** | **Competing events (death)** | **SHR (95% CI)** | **P value** |
| --- | --- | --- | --- | --- | --- |
| PPI vs Non-PPI (Fine-Gray Model 3) | 492,858 | 3,488 | 39,502 | 1.63 (1.50–1.79) | <0.001 |
| PPI vs H2RA (Fine-Gray Model 3) | 55,011 | 683 | 7,625 | 1.38 (1.07–1.77) | 0.013 |

Note: Fine-Gray estimates were consistent with the corresponding Cox model results, indicating that the associations were not materially explained by competing mortality. Abbreviations: CI, confidence interval; H2RA, histamine-2 receptor antagonist; PPI, proton pump inhibitor; SHR, subdistribution hazard ratio.

**Supplementary Table S5. FAERS drug and exclusion definitions (S5A–S5C)**

Drug matching criteria applied to the FAERS drug name field. All matching is case-insensitive partial string matching. S5A: PPI exposure group. S5B: chronic non-GI control group. S5C: exclusion drugs (ASM and antipsychotics).

**Table S5A. PPI agent matching patterns**

| **PPI agent** | **FAERS matching pattern** | **Notes** |
| --- | --- | --- |
| Omeprazole | omepra* | Case-insensitive partial match |
| Esomeprazole | esomepra* | Searched before lansopra* to avoid misclassification |
| Lansoprazole | lansopra* | Case-insensitive partial match |
| Pantoprazole | pantopra* | Case-insensitive partial match |
| Rabeprazole | rabepra* | Case-insensitive partial match |
| Dexlansoprazole | dexlansopra* | Searched before lansopra* to avoid misclassification |

**Table S5B. Control group — chronic non-GI medication classes and included agents**

| **Drug class** | **Included agents** |
| --- | --- |
| Calcium-channel blocker | Amlodipine; Felodipine; Nifedipine |
| Calcium-channel blocker (non-DHP) | Diltiazem; Verapamil |
| ACE inhibitor | Lisinopril; Ramipril; Enalapril; Perindopril |
| Angiotensin receptor blocker | Losartan; Valsartan; Olmesartan; Telmisartan; Candesartan |
| Beta-blocker | Metoprolol; Atenolol; Bisoprolol; Carvedilol |
| Diuretic | Hydrochlorothiazide; Indapamide; Spironolactone |
| Statin | Atorvastatin; Rosuvastatin; Simvastatin; Pravastatin |
| Other lipid-lowering agent | Ezetimibe; Fenofibrate |
| Antidiabetic (biguanide) | Metformin |
| Antidiabetic (sulfonylurea) | Glipizide; Glyburide; Glimepiride |
| Antidiabetic — thiazolidinedione | Pioglitazone |
| Antidiabetic — DPP-4i | Sitagliptin; Linagliptin |
| Antidiabetic — SGLT-2i | Empagliflozin; Dapagliflozin; Canagliflozin |
| Antidiabetic — GLP-1 | Liraglutide; Semaglutide |
| Antidiabetic — insulin | Insulin (all formulations) |

**Table S5C. Exclusion drug matching patterns — ASM and antipsychotics**

| **Drug class** | **FAERS matching strings** | **Notes** |
| --- | --- | --- |
| Antiseizure medication (ASM) | levetiracetam; valproic\|valproate; lamotrigine; carbamazepine; oxcarbazepine; phenytoin; phenobarbital; topiramate; gabapentin; pregabalin; zonisamide; lacosamide; perampanel; clobazam; clonazepam; ethosuximide; vigabatrin | Case-insensitive partial matching in FAERS drugname field |
| Antipsychotic | olanzapine; quetiapine; risperidone; aripiprazole; clozapine; haloperidol; chlorpromazine | Case-insensitive partial matching in FAERS drugname field |

Abbreviations: ACEi, ACE inhibitor; ARB, angiotensin receptor blocker; ASM, antiseizure medication; CCB, calcium channel blocker; DHP, dihydropyridine; FAERS, FDA Adverse Event Reporting System; GI, gastrointestinal; GLP-1, glucagon-like peptide-1; PPI, proton pump inhibitor; SGLT-2i, sodium-glucose cotransporter-2 inhibitor.

**Supplementary Table S6. FAERS disproportionality analysis — complete results**

Reporting odds ratio (ROR) and proportional reporting ratio (PRR) with 95% CIs. Positive disproportionality signal defined as lower bound of 95% CI >1.0. Analyses stratified by PPI agent, epilepsy event subtype, case definition, calendar period, and age group.

| **Analysis** | **Category** | **ROR** | **95% CI** | **Positive signal** | **PRR** | **PRR 95% CI** |
| --- | --- | --- | --- | --- | --- | --- |
| Overall (any PPI) | All epilepsy-related events | 1.70 | 1.61–1.79 | Yes | 1.69 | 1.60–1.78 |
| Omeprazole | By PPI agent | 2.86 | 2.63–3.10 | Yes | — | — |
| Esomeprazole | By PPI agent | 1.16 | 1.06–1.27 | Yes | — | — |
| Lansoprazole | By PPI agent | 1.00 | 0.86–1.17 | No | — | — |
| Pantoprazole | By PPI agent | 2.26 | 2.05–2.50 | Yes | — | — |
| Rabeprazole | By PPI agent | 1.62 | 1.17–2.24 | Yes | — | — |
| Dexlansoprazole | By PPI agent | 0.62 | 0.40–0.96 | No | — | — |
| Epilepsy PT | By event subtype | 2.33 | 2.07–2.63 | Yes | — | — |
| Seizure PT | By event subtype | 1.66 | 1.54–1.78 | Yes | — | — |
| Convulsion PT | By event subtype | 1.41 | 1.27–1.57 | Yes | — | — |
| Status epilepticus PT | By event subtype | 3.27 | 2.46–4.33 | Yes | — | — |
| Strict (epilepsy/SE only) | By case definition | 2.33 | 2.07–2.63 | Yes | — | — |
| Broad (all four terms) | By case definition | 1.70 | 1.61–1.79 | Yes | 1.69 | 1.60–1.78 |
| 2004–2010 | By calendar period | 1.25 | 1.07–1.46 | Yes | — | — |
| 2011–2015 | By calendar period | 1.72 | 1.54–1.91 | Yes | — | — |
| 2016–2020 | By calendar period | 1.28 | 1.16–1.41 | Yes | — | — |
| 2021–2025 | By calendar period | 3.11 | 2.84–3.41 | Yes | — | — |
| <60 years | By age group | 1.31 | 1.20–1.43 | Yes | — | — |
| ≥60 years | By age group | 2.70 | 2.49–2.92 | Yes | — | — |

Abbreviations: CI, confidence interval; FAERS, FDA Adverse Event Reporting System; PRR, proportional reporting ratio; PT, preferred term; ROR, reporting odds ratio; SE, status epilepticus.
Note: PRR reported for overall analysis only; agent-specific and subgroup PRR values not shown (—) as disproportionality was primarily assessed using ROR. Dexlansoprazole inverse signal likely reflects sparse reporting given limited market penetration.

**Supplementary Table S7. Bridge proteins linking PPI use and incident epilepsy — complete list (N=312)**

All 312 proteins significantly associated with both PPI use (linear regression, FDR<0.05) and incident epilepsy (Cox regression, FDR<0.05) with concordant directional effects. Sorted by absolute product of effect sizes (|β_lm × β_cox|). Abbreviations: FDR, false discovery rate; HR, hazard ratio; PPI, proton pump inhibitor; prot, protein; SE, standard error.

| **Protein** | **N (linear)** | **β (PPI→prot)** | **SE** | **FDR (PPI→prot)** | **N (Cox)** | **Events** | **β (prot→epi)** | **HR** | **HR lower 95% CI** | **HR upper 95% CI** | **FDR (prot→epi)** | **Direction** |
| --- | --- | --- | --- | --- | --- | --- | --- | --- | --- | --- | --- | --- |
| gdf15 | 51,179 | 0.2518 | 0.0069 | <0.001 | 51,179 | 413 | 0.3414019 | 1.407 | 1.301 | 1.522 | <0.001 | Risk |
| tff2 | 50,690 | 0.3486 | 0.0092 | <0.001 | 50,690 | 413 | 0.1710865 | 1.187 | 1.076 | 1.309 | 0.010 | Risk |
| ace2 | 49,637 | 0.1781 | 0.0082 | <0.001 | 49,637 | 403 | 0.2539893 | 1.289 | 1.176 | 1.413 | <0.001 | Risk |
| wfdc2 | 47,989 | 0.1273 | 0.0063 | <0.001 | 47,989 | 384 | 0.3080024 | 1.361 | 1.241 | 1.492 | <0.001 | Risk |
| chi3l1 | 49,446 | 0.1607 | 0.0126 | <0.001 | 49,446 | 409 | 0.2418725 | 1.274 | 1.161 | 1.397 | <0.001 | Risk |
| havcr1 | 50,913 | 0.1471 | 0.0102 | <0.001 | 50,913 | 416 | 0.2551668 | 1.291 | 1.170 | 1.424 | <0.001 | Risk |
| ren | 50,557 | 0.2007 | 0.0122 | <0.001 | 50,557 | 410 | 0.1808333 | 1.198 | 1.094 | 1.312 | 0.002 | Risk |
| ggt1 | 50,487 | 0.1422 | 0.0079 | <0.001 | 50,487 | 402 | 0.2551748 | 1.291 | 1.174 | 1.419 | <0.001 | Risk |
| rbp5 | 50,329 | 0.1439 | 0.0091 | <0.001 | 50,329 | 411 | 0.2505181 | 1.285 | 1.166 | 1.416 | <0.001 | Risk |
| vsig4 | 50,499 | 0.1183 | 0.0066 | <0.001 | 50,499 | 401 | 0.2972149 | 1.346 | 1.223 | 1.482 | <0.001 | Risk |
| ntprobnp | 50,557 | 0.1762 | 0.0168 | <0.001 | 50,557 | 410 | 0.1990882 | 1.220 | 1.110 | 1.342 | 0.001 | Risk |
| nefl | 49,217 | 0.0819 | 0.0072 | <0.001 | 49,217 | 398 | 0.4225620 | 1.526 | 1.406 | 1.656 | <0.001 | Risk |
| agr2 | 48,971 | 0.2040 | 0.0165 | <0.001 | 48,971 | 397 | 0.1693853 | 1.185 | 1.075 | 1.305 | 0.009 | Risk |
| krt18 | 50,329 | 0.2101 | 0.0131 | <0.001 | 50,329 | 411 | 0.1620477 | 1.176 | 1.064 | 1.300 | 0.020 | Risk |
| igfbp4 | 49,917 | 0.1239 | 0.0069 | <0.001 | 49,917 | 397 | 0.2505993 | 1.285 | 1.173 | 1.407 | <0.001 | Risk |
| gpr37 | 49,637 | 0.1211 | 0.0107 | <0.001 | 49,637 | 403 | 0.2499410 | 1.284 | 1.161 | 1.420 | <0.001 | Risk |
| baiap2 | 49,375 | 0.1426 | 0.0104 | <0.001 | 49,375 | 399 | 0.2071442 | 1.230 | 1.121 | 1.350 | <0.001 | Risk |
| eda2r | 50,987 | 0.0905 | 0.0061 | <0.001 | 50,987 | 416 | 0.3183505 | 1.375 | 1.234 | 1.531 | <0.001 | Risk |
| bst2 | 50,996 | 0.1423 | 0.0085 | <0.001 | 50,996 | 413 | 0.1995102 | 1.221 | 1.127 | 1.322 | <0.001 | Risk |
| lgals9 | 50,524 | 0.1161 | 0.0051 | <0.001 | 50,524 | 411 | 0.2430481 | 1.275 | 1.150 | 1.414 | <0.001 | Risk |
| tff1 | 50,321 | 0.1554 | 0.0127 | <0.001 | 50,321 | 399 | 0.1804307 | 1.198 | 1.091 | 1.315 | 0.003 | Risk |
| nppb | 49,637 | 0.1556 | 0.0219 | <0.001 | 49,637 | 403 | 0.1779925 | 1.195 | 1.078 | 1.325 | 0.011 | Risk |
| cxcl9 | 50,774 | 0.1584 | 0.0108 | <0.001 | 50,774 | 413 | 0.1732070 | 1.189 | 1.083 | 1.306 | 0.005 | Risk |
| mme | 50,329 | 0.1714 | 0.0130 | <0.001 | 50,329 | 411 | 0.1592300 | 1.173 | 1.065 | 1.291 | 0.016 | Risk |
| lilrb4 | 49,616 | 0.0995 | 0.0071 | <0.001 | 49,616 | 400 | 0.2739937 | 1.315 | 1.192 | 1.451 | <0.001 | Risk |
| prss8 | 50,359 | 0.0995 | 0.0066 | <0.001 | 50,359 | 409 | 0.2729301 | 1.314 | 1.180 | 1.463 | <0.001 | Risk |
| fuom | 42,991 | 0.1619 | 0.0103 | <0.001 | 42,991 | 341 | 0.1609323 | 1.175 | 1.054 | 1.309 | 0.037 | Risk |
| areg | 50,329 | 0.0970 | 0.0075 | <0.001 | 50,329 | 411 | 0.2652881 | 1.304 | 1.199 | 1.418 | <0.001 | Risk |
| lgals4 | 50,526 | 0.1186 | 0.0088 | <0.001 | 50,526 | 413 | 0.2166905 | 1.242 | 1.129 | 1.366 | <0.001 | Risk |
| echdc3 | 43,947 | 0.1391 | 0.0121 | <0.001 | 43,947 | 351 | 0.1804794 | 1.198 | 1.089 | 1.317 | 0.004 | Risk |
| cd302 | 50,329 | 0.0903 | 0.0049 | <0.001 | 50,329 | 411 | 0.2765186 | 1.319 | 1.205 | 1.443 | <0.001 | Risk |
| lamp3 | 51,028 | 0.1136 | 0.0083 | <0.001 | 51,028 | 414 | 0.2193800 | 1.245 | 1.118 | 1.387 | 0.002 | Risk |
| ocln | 43,873 | 0.1018 | 0.0070 | <0.001 | 43,873 | 349 | 0.2441729 | 1.277 | 1.156 | 1.409 | <0.001 | Risk |
| mmp12 | 50,590 | 0.1178 | 0.0086 | <0.001 | 50,590 | 415 | 0.2068709 | 1.230 | 1.110 | 1.362 | 0.002 | Risk |
| palm2 | 43,853 | 0.1034 | 0.0061 | <0.001 | 43,853 | 348 | 0.2349670 | 1.265 | 1.147 | 1.395 | <0.001 | Risk |
| havcr2 | 50,569 | 0.1045 | 0.0056 | <0.001 | 50,569 | 402 | 0.2286282 | 1.257 | 1.141 | 1.385 | <0.001 | Risk |
| gprc5c | 43,748 | 0.1401 | 0.0091 | <0.001 | 43,748 | 350 | 0.1680150 | 1.183 | 1.089 | 1.285 | 0.002 | Risk |
| asgr1 | 50,160 | 0.0989 | 0.0048 | <0.001 | 50,160 | 407 | 0.2369403 | 1.267 | 1.150 | 1.396 | <0.001 | Risk |
| epo | 50,488 | 0.1728 | 0.0124 | <0.001 | 50,488 | 408 | 0.1346317 | 1.144 | 1.049 | 1.248 | 0.028 | Risk |
| reg3a | 51,179 | 0.1289 | 0.0100 | <0.001 | 51,179 | 413 | 0.1796892 | 1.197 | 1.091 | 1.312 | 0.003 | Risk |
| siglec1 | 50,774 | 0.0988 | 0.0069 | <0.001 | 50,774 | 413 | 0.2340819 | 1.264 | 1.147 | 1.393 | <0.001 | Risk |
| grpel1 | 50,329 | 0.1338 | 0.0098 | <0.001 | 50,329 | 411 | 0.1718354 | 1.187 | 1.087 | 1.298 | 0.003 | Risk |
| reg1b | 51,179 | 0.1380 | 0.0092 | <0.001 | 51,179 | 413 | 0.1643807 | 1.179 | 1.080 | 1.287 | 0.004 | Risk |
| cdcp1 | 50,160 | 0.1320 | 0.0084 | <0.001 | 50,160 | 407 | 0.1711990 | 1.187 | 1.071 | 1.315 | 0.015 | Risk |
| ca6 | 50,410 | -0.1388 | 0.0103 | <0.001 | 50,410 | 401 | -0.1601247 | 0.852 | 0.766 | 0.948 | 0.034 | Protective |
| ifi30 | 42,713 | 0.1248 | 0.0063 | <0.001 | 42,713 | 339 | 0.1763457 | 1.193 | 1.070 | 1.330 | 0.020 | Risk |
| inhbb | 42,991 | 0.1137 | 0.0083 | <0.001 | 42,991 | 341 | 0.1907386 | 1.210 | 1.088 | 1.347 | 0.008 | Risk |
| umod | 51,019 | -0.1045 | 0.0087 | <0.001 | 51,019 | 411 | -0.2072037 | 0.813 | 0.739 | 0.894 | <0.001 | Protective |
| pigr | 49,914 | 0.0722 | 0.0059 | <0.001 | 49,914 | 394 | 0.2999867 | 1.350 | 1.222 | 1.491 | <0.001 | Risk |
| spink1 | 50,569 | 0.0805 | 0.0068 | <0.001 | 50,569 | 402 | 0.2668902 | 1.306 | 1.201 | 1.420 | <0.001 | Risk |
| plaur | 50,194 | 0.0771 | 0.0044 | <0.001 | 50,194 | 409 | 0.2774611 | 1.320 | 1.194 | 1.459 | <0.001 | Risk |
| tnfrsf6b | 50,160 | 0.1052 | 0.0091 | <0.001 | 50,160 | 407 | 0.2002243 | 1.222 | 1.117 | 1.337 | <0.001 | Risk |
| tnfrsf4 | 51,028 | 0.1099 | 0.0060 | <0.001 | 51,028 | 414 | 0.1913852 | 1.211 | 1.104 | 1.328 | 0.001 | Risk |
| col6a3 | 51,179 | 0.0843 | 0.0055 | <0.001 | 51,179 | 413 | 0.2401743 | 1.271 | 1.156 | 1.399 | <0.001 | Risk |
| mmp7 | 49,561 | 0.0963 | 0.0080 | <0.001 | 49,561 | 402 | 0.2101483 | 1.234 | 1.109 | 1.373 | 0.002 | Risk |
| epha2 | 48,733 | 0.0797 | 0.0050 | <0.001 | 48,733 | 400 | 0.2499239 | 1.284 | 1.184 | 1.392 | <0.001 | Risk |
| cd27 | 50,712 | 0.0821 | 0.0066 | <0.001 | 50,712 | 411 | 0.2411030 | 1.273 | 1.163 | 1.393 | <0.001 | Risk |
| tnfrsf10a | 50,160 | 0.0894 | 0.0053 | <0.001 | 50,160 | 407 | 0.2202452 | 1.246 | 1.147 | 1.355 | <0.001 | Risk |
| cntn5 | 50,672 | -0.0838 | 0.0060 | <0.001 | 50,672 | 412 | -0.2335869 | 0.792 | 0.711 | 0.881 | <0.001 | Protective |
| pts | 49,140 | 0.1392 | 0.0091 | <0.001 | 49,140 | 398 | 0.1403424 | 1.151 | 1.046 | 1.266 | 0.038 | Risk |
| ccl7 | 50,488 | 0.1192 | 0.0095 | <0.001 | 50,488 | 408 | 0.1631187 | 1.177 | 1.080 | 1.283 | 0.004 | Risk |
| fstl3 | 50,446 | 0.0862 | 0.0051 | <0.001 | 50,446 | 411 | 0.2235314 | 1.250 | 1.131 | 1.383 | <0.001 | Risk |
| il2ra | 50,196 | 0.0907 | 0.0066 | <0.001 | 50,196 | 406 | 0.2090984 | 1.233 | 1.125 | 1.351 | <0.001 | Risk |
| itga11 | 50,488 | -0.0997 | 0.0056 | <0.001 | 50,488 | 408 | -0.1898120 | 0.827 | 0.749 | 0.914 | 0.004 | Protective |
| trem2 | 50,526 | 0.1281 | 0.0085 | <0.001 | 50,526 | 413 | 0.1463319 | 1.158 | 1.045 | 1.283 | 0.046 | Risk |
| adgrg1 | 50,329 | 0.1049 | 0.0112 | <0.001 | 50,329 | 411 | 0.1784848 | 1.195 | 1.098 | 1.302 | 0.001 | Risk |
| cd74 | 50,996 | 0.0825 | 0.0056 | <0.001 | 50,996 | 413 | 0.2226507 | 1.249 | 1.132 | 1.378 | <0.001 | Risk |
| fabp1 | 50,526 | 0.1093 | 0.0155 | <0.001 | 50,526 | 413 | 0.1672053 | 1.182 | 1.070 | 1.306 | 0.014 | Risk |
| npc2 | 43,213 | 0.0907 | 0.0051 | <0.001 | 43,213 | 345 | 0.2000693 | 1.221 | 1.113 | 1.341 | <0.001 | Risk |
| adm | 50,987 | 0.0694 | 0.0043 | <0.001 | 50,987 | 416 | 0.2589812 | 1.296 | 1.148 | 1.462 | <0.001 | Risk |
| ctsl | 50,557 | 0.0681 | 0.0039 | <0.001 | 50,557 | 410 | 0.2623485 | 1.300 | 1.186 | 1.425 | <0.001 | Risk |
| msr1 | 50,996 | 0.1013 | 0.0075 | <0.001 | 50,996 | 413 | 0.1763837 | 1.193 | 1.072 | 1.327 | 0.016 | Risk |
| siglec5 | 50,569 | 0.1054 | 0.0193 | <0.001 | 50,569 | 402 | 0.1693196 | 1.184 | 1.056 | 1.329 | 0.038 | Risk |
| tnfrsf1a | 50,569 | 0.0669 | 0.0047 | <0.001 | 50,569 | 402 | 0.2650452 | 1.303 | 1.188 | 1.430 | <0.001 | Risk |
| cxadr | 50,488 | 0.0789 | 0.0083 | <0.001 | 50,488 | 408 | 0.2243085 | 1.251 | 1.148 | 1.365 | <0.001 | Risk |
| il4r | 50,488 | 0.0770 | 0.0059 | <0.001 | 50,488 | 408 | 0.2275260 | 1.255 | 1.151 | 1.369 | <0.001 | Risk |
| tnfrsf1b | 50,569 | 0.0977 | 0.0058 | <0.001 | 50,569 | 402 | 0.1786083 | 1.196 | 1.121 | 1.275 | <0.001 | Risk |
| spon2 | 50,315 | 0.0745 | 0.0056 | <0.001 | 50,315 | 408 | 0.2274731 | 1.255 | 1.140 | 1.383 | <0.001 | Risk |
| il6 | 50,329 | 0.1359 | 0.0120 | <0.001 | 50,329 | 411 | 0.1246275 | 1.133 | 1.037 | 1.238 | 0.050 | Risk |
| tnfrsf9 | 50,160 | 0.0977 | 0.0066 | <0.001 | 50,160 | 407 | 0.1727152 | 1.189 | 1.091 | 1.295 | 0.002 | Risk |
| agxt | 50,557 | 0.1208 | 0.0125 | <0.001 | 50,557 | 410 | 0.1395674 | 1.150 | 1.046 | 1.264 | 0.038 | Risk |
| scarb2 | 49,204 | 0.0725 | 0.0050 | <0.001 | 49,204 | 403 | 0.2316650 | 1.261 | 1.151 | 1.381 | <0.001 | Risk |
| pilra | 50,569 | 0.0728 | 0.0068 | <0.001 | 50,569 | 402 | 0.2291078 | 1.257 | 1.134 | 1.395 | <0.001 | Risk |
| tnfrsf11a | 51,111 | 0.0799 | 0.0061 | <0.001 | 51,111 | 414 | 0.2066754 | 1.230 | 1.118 | 1.353 | <0.001 | Risk |
| slamf8 | 49,375 | 0.0821 | 0.0088 | <0.001 | 49,375 | 399 | 0.2002622 | 1.222 | 1.104 | 1.353 | 0.002 | Risk |
| gfra1 | 50,910 | 0.0729 | 0.0045 | <0.001 | 50,910 | 416 | 0.2243683 | 1.252 | 1.134 | 1.381 | <0.001 | Risk |
| ccl11 | 51,111 | 0.0914 | 0.0062 | <0.001 | 51,111 | 414 | 0.1778380 | 1.195 | 1.077 | 1.325 | 0.012 | Risk |
| ulbp2 | 50,160 | 0.0814 | 0.0061 | <0.001 | 50,160 | 407 | 0.1985495 | 1.220 | 1.108 | 1.343 | 0.001 | Risk |
| ctsd | 47,266 | 0.0859 | 0.0066 | <0.001 | 47,266 | 394 | 0.1874262 | 1.206 | 1.106 | 1.316 | <0.001 | Risk |
| cstb | 49,858 | 0.0919 | 0.0071 | <0.001 | 49,858 | 405 | 0.1750616 | 1.191 | 1.079 | 1.315 | 0.008 | Risk |
| ckap4 | 50,863 | 0.0631 | 0.0044 | <0.001 | 50,863 | 412 | 0.2547605 | 1.290 | 1.195 | 1.393 | <0.001 | Risk |
| spp1 | 51,099 | 0.0950 | 0.0075 | <0.001 | 51,099 | 413 | 0.1688956 | 1.184 | 1.074 | 1.306 | 0.011 | Risk |
| il18bp | 50,760 | 0.0724 | 0.0049 | <0.001 | 50,760 | 415 | 0.2210674 | 1.247 | 1.137 | 1.369 | <0.001 | Risk |
| klk4 | 50,987 | 0.0901 | 0.0103 | <0.001 | 50,987 | 416 | 0.1772841 | 1.194 | 1.088 | 1.310 | 0.004 | Risk |
| ada2 | 50,264 | 0.0794 | 0.0069 | <0.001 | 50,264 | 411 | 0.1994712 | 1.221 | 1.109 | 1.344 | 0.001 | Risk |
| fgf23 | 50,903 | 0.1200 | 0.0092 | <0.001 | 50,903 | 416 | 0.1312222 | 1.140 | 1.048 | 1.241 | 0.026 | Risk |
| il10 | 49,616 | 0.1250 | 0.0134 | <0.001 | 49,616 | 400 | 0.1254756 | 1.134 | 1.042 | 1.233 | 0.035 | Risk |
| il1rl1 | 51,011 | 0.0790 | 0.0076 | <0.001 | 51,011 | 411 | 0.1974963 | 1.218 | 1.095 | 1.355 | 0.005 | Risk |
| efna4 | 50,076 | 0.0676 | 0.0048 | <0.001 | 50,076 | 407 | 0.2300398 | 1.259 | 1.153 | 1.374 | <0.001 | Risk |
| rbfox3 | 43,873 | 0.0854 | 0.0062 | <0.001 | 43,873 | 349 | 0.1804914 | 1.198 | 1.093 | 1.313 | 0.003 | Risk |
| rab6a | 50,488 | -0.0695 | 0.0076 | <0.001 | 50,488 | 408 | -0.2197278 | 0.803 | 0.723 | 0.891 | <0.001 | Protective |
| tnfrsf8 | 50,996 | 0.0938 | 0.0073 | <0.001 | 50,996 | 413 | 0.1613771 | 1.175 | 1.074 | 1.285 | 0.007 | Risk |
| pilrb | 50,557 | 0.0743 | 0.0093 | <0.001 | 50,557 | 410 | 0.2013767 | 1.223 | 1.100 | 1.359 | 0.004 | Risk |
| rnase6 | 43,672 | 0.0895 | 0.0048 | <0.001 | 43,672 | 346 | 0.1668259 | 1.182 | 1.100 | 1.269 | <0.001 | Risk |
| ncr3lg1 | 43,769 | 0.0645 | 0.0055 | <0.001 | 43,769 | 349 | 0.2310424 | 1.260 | 1.136 | 1.397 | <0.001 | Risk |
| sncg | 50,160 | 0.0935 | 0.0108 | <0.001 | 50,160 | 407 | 0.1591897 | 1.173 | 1.052 | 1.308 | 0.039 | Risk |
| sirpb1 | 50,444 | 0.0718 | 0.0075 | <0.001 | 50,444 | 413 | 0.2063830 | 1.229 | 1.117 | 1.353 | <0.001 | Risk |
| lrrn1 | 49,616 | -0.0689 | 0.0064 | <0.001 | 49,616 | 400 | -0.2130253 | 0.808 | 0.732 | 0.892 | <0.001 | Protective |
| cst3 | 50,676 | 0.0688 | 0.0045 | <0.001 | 50,676 | 414 | 0.2129817 | 1.237 | 1.120 | 1.367 | <0.001 | Risk |
| siglec8 | 42,783 | 0.0689 | 0.0074 | <0.001 | 42,783 | 343 | 0.2107485 | 1.235 | 1.108 | 1.376 | 0.003 | Risk |
| tnfrsf10b | 50,160 | 0.1190 | 0.0064 | <0.001 | 50,160 | 407 | 0.1219487 | 1.130 | 1.067 | 1.196 | <0.001 | Risk |
| layn | 50,076 | 0.0648 | 0.0055 | <0.001 | 50,076 | 407 | 0.2240270 | 1.251 | 1.141 | 1.371 | <0.001 | Risk |
| reg1a | 50,262 | 0.0862 | 0.0080 | <0.001 | 50,262 | 411 | 0.1680267 | 1.183 | 1.084 | 1.291 | 0.003 | Risk |
| lair1 | 50,526 | 0.0885 | 0.0063 | <0.001 | 50,526 | 413 | 0.1633331 | 1.177 | 1.079 | 1.285 | 0.004 | Risk |
| nectin2 | 50,483 | 0.0634 | 0.0047 | <0.001 | 50,483 | 409 | 0.2243807 | 1.252 | 1.143 | 1.370 | <0.001 | Risk |
| rida | 42,991 | 0.0764 | 0.0081 | <0.001 | 42,991 | 341 | 0.1846473 | 1.203 | 1.084 | 1.334 | 0.008 | Risk |
| tnfrsf14 | 50,609 | 0.0658 | 0.0049 | <0.001 | 50,609 | 413 | 0.2134358 | 1.238 | 1.129 | 1.357 | <0.001 | Risk |
| clec7a | 51,111 | 0.0514 | 0.0096 | <0.001 | 51,111 | 414 | 0.2713406 | 1.312 | 1.160 | 1.484 | <0.001 | Risk |
| hnmt | 49,921 | 0.0763 | 0.0077 | <0.001 | 49,921 | 407 | 0.1824965 | 1.200 | 1.084 | 1.329 | 0.008 | Risk |
| shisa5 | 43,951 | 0.0646 | 0.0041 | <0.001 | 43,951 | 351 | 0.2152542 | 1.240 | 1.155 | 1.331 | <0.001 | Risk |
| cd300e | 50,823 | 0.0765 | 0.0065 | <0.001 | 50,823 | 416 | 0.1808462 | 1.198 | 1.086 | 1.322 | 0.006 | Risk |
| prg2 | 43,951 | 0.0603 | 0.0084 | <0.001 | 43,951 | 351 | 0.2271641 | 1.255 | 1.134 | 1.389 | <0.001 | Risk |
| pcbd1 | 42,912 | 0.0875 | 0.0076 | <0.001 | 42,912 | 341 | 0.1562061 | 1.169 | 1.054 | 1.297 | 0.034 | Risk |
| cd300a | 42,991 | 0.0594 | 0.0048 | <0.001 | 42,991 | 341 | 0.2290227 | 1.257 | 1.133 | 1.396 | <0.001 | Risk |
| cd80 | 43,947 | 0.0713 | 0.0055 | <0.001 | 43,947 | 351 | 0.1908916 | 1.210 | 1.095 | 1.338 | 0.004 | Risk |
| creld1 | 42,991 | 0.0656 | 0.0061 | <0.001 | 42,991 | 341 | 0.2049142 | 1.227 | 1.098 | 1.372 | 0.005 | Risk |
| npdc1 | 50,557 | 0.0742 | 0.0051 | <0.001 | 50,557 | 410 | 0.1800964 | 1.197 | 1.096 | 1.308 | 0.002 | Risk |
| csf1 | 50,774 | 0.0618 | 0.0043 | <0.001 | 50,774 | 413 | 0.2160783 | 1.241 | 1.127 | 1.367 | <0.001 | Risk |
| cxcl13 | 50,996 | 0.1074 | 0.0099 | <0.001 | 50,996 | 413 | 0.1240394 | 1.132 | 1.045 | 1.226 | 0.026 | Risk |
| enpp7 | 48,992 | 0.0796 | 0.0141 | <0.001 | 48,992 | 395 | 0.1671919 | 1.182 | 1.068 | 1.308 | 0.016 | Risk |
| rnf149 | 42,783 | 0.0611 | 0.0050 | <0.001 | 42,783 | 343 | 0.2152406 | 1.240 | 1.138 | 1.352 | <0.001 | Risk |
| cd83 | 50,488 | 0.0790 | 0.0054 | <0.001 | 50,488 | 408 | 0.1637750 | 1.178 | 1.068 | 1.300 | 0.015 | Risk |
| ptn | 49,637 | 0.0888 | 0.0082 | <0.001 | 49,637 | 403 | 0.1449715 | 1.156 | 1.046 | 1.278 | 0.041 | Risk |
| tgfa | 50,488 | 0.0776 | 0.0075 | <0.001 | 50,488 | 408 | 0.1646596 | 1.179 | 1.074 | 1.294 | 0.008 | Risk |
| hsd17b14 | 43,853 | 0.0753 | 0.0101 | <0.001 | 43,853 | 348 | 0.1687655 | 1.184 | 1.068 | 1.313 | 0.018 | Risk |
| cd300lf | 50,987 | 0.0781 | 0.0099 | <0.001 | 50,987 | 416 | 0.1614791 | 1.175 | 1.056 | 1.308 | 0.033 | Risk |
| hgf | 51,111 | 0.0756 | 0.0056 | <0.001 | 51,111 | 414 | 0.1653312 | 1.180 | 1.063 | 1.310 | 0.023 | Risk |
| cd274 | 50,160 | 0.0630 | 0.0055 | <0.001 | 50,160 | 407 | 0.1977849 | 1.219 | 1.118 | 1.328 | <0.001 | Risk |
| agrn | 50,774 | 0.0856 | 0.0054 | <0.001 | 50,774 | 413 | 0.1450088 | 1.156 | 1.051 | 1.272 | 0.031 | Risk |
| sema3g | 42,991 | 0.0800 | 0.0072 | <0.001 | 42,991 | 341 | 0.1551938 | 1.168 | 1.057 | 1.291 | 0.026 | Risk |
| cdh2 | 50,557 | 0.0561 | 0.0058 | <0.001 | 50,557 | 410 | 0.2196425 | 1.246 | 1.118 | 1.388 | 0.002 | Risk |
| chchd10 | 43,853 | 0.0691 | 0.0065 | <0.001 | 43,853 | 348 | 0.1784216 | 1.195 | 1.080 | 1.323 | 0.009 | Risk |
| tgfbr2 | 50,712 | 0.0595 | 0.0052 | <0.001 | 50,712 | 411 | 0.2057807 | 1.228 | 1.123 | 1.344 | <0.001 | Risk |
| micb_mica | 50,488 | 0.0560 | 0.0245 | 0.043 | 50,488 | 408 | 0.2171131 | 1.242 | 1.101 | 1.402 | 0.007 | Risk |
| colec12 | 50,526 | 0.0511 | 0.0042 | <0.001 | 50,526 | 413 | 0.2373683 | 1.268 | 1.145 | 1.404 | <0.001 | Risk |
| itgbl1 | 43,951 | 0.0574 | 0.0059 | <0.001 | 43,951 | 351 | 0.2112847 | 1.235 | 1.123 | 1.358 | <0.001 | Risk |
| tafa5 | 50,329 | 0.0791 | 0.0058 | <0.001 | 50,329 | 411 | 0.1520167 | 1.164 | 1.059 | 1.280 | 0.021 | Risk |
| ltbp2 | 51,179 | 0.0532 | 0.0051 | <0.001 | 51,179 | 413 | 0.2255348 | 1.253 | 1.122 | 1.399 | 0.001 | Risk |
| pik3ip1 | 50,569 | 0.0499 | 0.0044 | <0.001 | 50,569 | 402 | 0.2397207 | 1.271 | 1.162 | 1.391 | <0.001 | Risk |
| tff3 | 50,514 | 0.0928 | 0.0075 | <0.001 | 50,514 | 413 | 0.1278389 | 1.136 | 1.046 | 1.235 | 0.028 | Risk |
| gchfr | 41,935 | 0.0726 | 0.0083 | <0.001 | 41,935 | 327 | 0.1611306 | 1.175 | 1.065 | 1.296 | 0.017 | Risk |
| vmo1 | 50,329 | 0.0715 | 0.0111 | <0.001 | 50,329 | 411 | 0.1634407 | 1.178 | 1.060 | 1.308 | 0.026 | Risk |
| pla2g15 | 50,712 | 0.0791 | 0.0043 | <0.001 | 50,712 | 411 | 0.1471036 | 1.158 | 1.051 | 1.277 | 0.032 | Risk |
| nbl1 | 50,712 | 0.0521 | 0.0043 | <0.001 | 50,712 | 411 | 0.2222265 | 1.249 | 1.137 | 1.372 | <0.001 | Risk |
| angpt2 | 50,987 | 0.0409 | 0.0062 | <0.001 | 50,987 | 416 | 0.2805815 | 1.324 | 1.206 | 1.453 | <0.001 | Risk |
| spink4 | 50,488 | 0.0723 | 0.0106 | <0.001 | 50,488 | 408 | 0.1583269 | 1.172 | 1.067 | 1.286 | 0.013 | Risk |
| il18r1 | 51,111 | 0.0792 | 0.0053 | <0.001 | 51,111 | 414 | 0.1437854 | 1.155 | 1.043 | 1.279 | 0.050 | Risk |
| crh | 49,294 | -0.0766 | 0.0143 | <0.001 | 49,294 | 399 | -0.1472410 | 0.863 | 0.779 | 0.956 | 0.044 | Protective |
| nt5c1a | 43,853 | 0.0675 | 0.0052 | <0.001 | 43,853 | 348 | 0.1666146 | 1.181 | 1.084 | 1.287 | 0.003 | Risk |
| sdc1 | 50,557 | 0.0633 | 0.0064 | <0.001 | 50,557 | 410 | 0.1770104 | 1.194 | 1.091 | 1.305 | 0.002 | Risk |
| fgfr2 | 50,712 | 0.0603 | 0.0036 | <0.001 | 50,712 | 411 | 0.1851764 | 1.203 | 1.090 | 1.329 | 0.005 | Risk |
| dsc2 | 49,999 | 0.0500 | 0.0054 | <0.001 | 49,999 | 396 | 0.2230584 | 1.250 | 1.134 | 1.377 | <0.001 | Risk |
| fgl1 | 43,984 | 0.0593 | 0.0104 | <0.001 | 43,984 | 348 | 0.1862786 | 1.205 | 1.078 | 1.347 | 0.015 | Risk |
| rnase1 | 43,984 | 0.0536 | 0.0040 | <0.001 | 43,984 | 348 | 0.2050345 | 1.228 | 1.130 | 1.333 | <0.001 | Risk |
| nhlrc3 | 41,955 | 0.0618 | 0.0052 | <0.001 | 41,955 | 334 | 0.1750227 | 1.191 | 1.060 | 1.339 | 0.035 | Risk |
| mad1l1 | 49,994 | 0.0906 | 0.0081 | <0.001 | 49,994 | 407 | 0.1187458 | 1.126 | 1.036 | 1.224 | 0.046 | Risk |
| pgf | 51,111 | 0.0520 | 0.0041 | <0.001 | 51,111 | 414 | 0.2065199 | 1.229 | 1.129 | 1.339 | <0.001 | Risk |
| zbtb17 | 49,637 | 0.0754 | 0.0070 | <0.001 | 49,637 | 403 | 0.1395794 | 1.150 | 1.063 | 1.244 | 0.008 | Risk |
| ltbr | 50,278 | 0.0449 | 0.0046 | <0.001 | 50,278 | 411 | 0.2332107 | 1.263 | 1.156 | 1.379 | <0.001 | Risk |
| mdk | 50,558 | 0.0613 | 0.0084 | <0.001 | 50,558 | 409 | 0.1696140 | 1.185 | 1.088 | 1.290 | 0.002 | Risk |
| adamtsl2 | 43,951 | 0.0556 | 0.0053 | <0.001 | 43,951 | 351 | 0.1869377 | 1.206 | 1.089 | 1.335 | 0.006 | Risk |
| dcbld2 | 50,712 | 0.0642 | 0.0057 | <0.001 | 50,712 | 411 | 0.1614809 | 1.175 | 1.063 | 1.299 | 0.020 | Risk |
| acvrl1 | 50,076 | 0.0459 | 0.0040 | <0.001 | 50,076 | 407 | 0.2247490 | 1.252 | 1.137 | 1.379 | <0.001 | Risk |
| tnfrsf12a | 50,712 | 0.0504 | 0.0057 | <0.001 | 50,712 | 411 | 0.2007948 | 1.222 | 1.108 | 1.348 | 0.001 | Risk |
| ptgds | 50,182 | 0.0615 | 0.0048 | <0.001 | 50,182 | 410 | 0.1643064 | 1.179 | 1.076 | 1.291 | 0.007 | Risk |
| ambp | 50,913 | 0.0419 | 0.0032 | <0.001 | 50,913 | 416 | 0.2397153 | 1.271 | 1.149 | 1.405 | <0.001 | Risk |
| lrp11 | 50,557 | 0.0575 | 0.0058 | <0.001 | 50,557 | 410 | 0.1741253 | 1.190 | 1.081 | 1.311 | 0.007 | Risk |
| il10rb | 50,774 | 0.0598 | 0.0047 | <0.001 | 50,774 | 413 | 0.1675079 | 1.182 | 1.080 | 1.295 | 0.005 | Risk |
| sorcs2 | 49,375 | 0.0683 | 0.0057 | <0.001 | 49,375 | 399 | 0.1461928 | 1.157 | 1.047 | 1.279 | 0.040 | Risk |
| tpp1 | 50,194 | 0.0754 | 0.0074 | <0.001 | 50,194 | 408 | 0.1324053 | 1.142 | 1.043 | 1.249 | 0.038 | Risk |
| timp1 | 50,013 | 0.0495 | 0.0046 | <0.001 | 50,013 | 406 | 0.2010639 | 1.223 | 1.118 | 1.337 | <0.001 | Risk |
| egfr | 51,179 | -0.0486 | 0.0027 | <0.001 | 51,179 | 413 | -0.2046061 | 0.815 | 0.741 | 0.897 | <0.001 | Protective |
| mrc1 | 43,984 | 0.0530 | 0.0046 | <0.001 | 43,984 | 348 | 0.1872082 | 1.206 | 1.081 | 1.345 | 0.011 | Risk |
| ctsv | 50,820 | -0.0579 | 0.0068 | <0.001 | 50,820 | 416 | -0.1704889 | 0.843 | 0.759 | 0.937 | 0.019 | Protective |
| ncs1 | 50,987 | 0.0480 | 0.0046 | <0.001 | 50,987 | 416 | 0.2032320 | 1.225 | 1.103 | 1.361 | 0.003 | Risk |
| ccl15 | 51,179 | 0.0557 | 0.0071 | <0.001 | 51,179 | 413 | 0.1746050 | 1.191 | 1.086 | 1.306 | 0.004 | Risk |
| il15 | 50,488 | 0.0617 | 0.0054 | <0.001 | 50,488 | 408 | 0.1567426 | 1.170 | 1.067 | 1.282 | 0.012 | Risk |
| rnaset2 | 50,937 | 0.0481 | 0.0043 | <0.001 | 50,937 | 413 | 0.1997817 | 1.221 | 1.116 | 1.336 | <0.001 | Risk |
| dll1 | 50,712 | 0.0445 | 0.0048 | <0.001 | 50,712 | 411 | 0.2147130 | 1.240 | 1.126 | 1.364 | <0.001 | Risk |
| vwc2 | 49,992 | 0.0560 | 0.0063 | <0.001 | 49,992 | 407 | 0.1700495 | 1.185 | 1.070 | 1.313 | 0.016 | Risk |
| ctso | 51,111 | 0.0593 | 0.0049 | <0.001 | 51,111 | 414 | 0.1604320 | 1.174 | 1.067 | 1.292 | 0.015 | Risk |
| galnt10 | 50,329 | 0.0440 | 0.0046 | <0.001 | 50,329 | 411 | 0.2150460 | 1.240 | 1.126 | 1.366 | <0.001 | Risk |
| tnfsf13b | 51,096 | 0.0560 | 0.0045 | <0.001 | 51,096 | 413 | 0.1685532 | 1.184 | 1.081 | 1.296 | 0.005 | Risk |
| psap | 43,538 | 0.0700 | 0.0046 | <0.001 | 43,538 | 347 | 0.1342326 | 1.144 | 1.040 | 1.258 | 0.050 | Risk |
| cd79b | 51,111 | 0.0573 | 0.0071 | <0.001 | 51,111 | 414 | 0.1583225 | 1.172 | 1.068 | 1.285 | 0.012 | Risk |
| ogn | 50,569 | 0.0541 | 0.0067 | <0.001 | 50,569 | 402 | 0.1626802 | 1.177 | 1.063 | 1.303 | 0.021 | Risk |
| icam1 | 50,346 | 0.0542 | 0.0047 | <0.001 | 50,346 | 412 | 0.1600994 | 1.174 | 1.065 | 1.293 | 0.016 | Risk |
| fstl1 | 42,783 | 0.0381 | 0.0037 | <0.001 | 42,783 | 343 | 0.2263065 | 1.254 | 1.140 | 1.380 | <0.001 | Risk |
| csf1r | 43,984 | 0.0509 | 0.0064 | <0.001 | 43,984 | 348 | 0.1681270 | 1.183 | 1.067 | 1.311 | 0.018 | Risk |
| sgsh | 43,208 | 0.0585 | 0.0091 | <0.001 | 43,208 | 344 | 0.1460319 | 1.157 | 1.050 | 1.275 | 0.034 | Risk |
| fbp1 | 50,557 | 0.0582 | 0.0120 | <0.001 | 50,557 | 410 | 0.1461865 | 1.157 | 1.051 | 1.274 | 0.032 | Risk |
| btn2a1 | 50,774 | 0.0403 | 0.0042 | <0.001 | 50,774 | 413 | 0.2082377 | 1.232 | 1.124 | 1.349 | <0.001 | Risk |
| ceacam8 | 49,637 | 0.0496 | 0.0082 | <0.001 | 49,637 | 403 | 0.1672374 | 1.182 | 1.075 | 1.300 | 0.009 | Risk |
| col18a1 | 50,767 | 0.0549 | 0.0038 | <0.001 | 50,767 | 410 | 0.1508084 | 1.163 | 1.048 | 1.290 | 0.041 | Risk |
| tgoln2 | 43,984 | 0.0438 | 0.0042 | <0.001 | 43,984 | 348 | 0.1886449 | 1.208 | 1.092 | 1.335 | 0.004 | Risk |
| il15ra | 49,616 | 0.0541 | 0.0057 | <0.001 | 49,616 | 400 | 0.1521658 | 1.164 | 1.054 | 1.287 | 0.031 | Risk |
| folr1 | 50,480 | 0.0457 | 0.0046 | <0.001 | 50,480 | 409 | 0.1787348 | 1.196 | 1.087 | 1.316 | 0.005 | Risk |
| kit | 50,679 | -0.0429 | 0.0048 | <0.001 | 50,679 | 414 | -0.1890389 | 0.828 | 0.749 | 0.915 | 0.004 | Protective |
| defa1_defa1b | 49,529 | 0.0501 | 0.0087 | <0.001 | 49,529 | 403 | 0.1600049 | 1.174 | 1.079 | 1.277 | 0.004 | Risk |
| nectin4 | 50,712 | 0.0498 | 0.0051 | <0.001 | 50,712 | 411 | 0.1603548 | 1.174 | 1.077 | 1.279 | 0.005 | Risk |
| ephb4 | 51,179 | 0.0417 | 0.0040 | <0.001 | 51,179 | 413 | 0.1905310 | 1.210 | 1.107 | 1.322 | <0.001 | Risk |
| btn3a2 | 50,488 | 0.0585 | 0.0062 | <0.001 | 50,488 | 408 | 0.1350737 | 1.145 | 1.043 | 1.256 | 0.040 | Risk |
| calb1 | 50,329 | 0.0497 | 0.0070 | <0.001 | 50,329 | 411 | 0.1584029 | 1.172 | 1.060 | 1.296 | 0.024 | Risk |
| septin8 | 43,771 | 0.0405 | 0.0053 | <0.001 | 43,771 | 346 | 0.1942350 | 1.214 | 1.110 | 1.328 | <0.001 | Risk |
| c1qa | 50,862 | 0.0548 | 0.0037 | <0.001 | 50,862 | 414 | 0.1411676 | 1.152 | 1.045 | 1.269 | 0.041 | Risk |
| hspg2 | 50,182 | 0.0472 | 0.0042 | <0.001 | 50,182 | 409 | 0.1595978 | 1.173 | 1.067 | 1.290 | 0.014 | Risk |
| eln | 43,947 | 0.0380 | 0.0056 | <0.001 | 43,947 | 351 | 0.1968287 | 1.218 | 1.062 | 1.396 | 0.044 | Risk |
| ceacam6 | 43,951 | 0.0395 | 0.0064 | <0.001 | 43,951 | 351 | 0.1886363 | 1.208 | 1.112 | 1.312 | <0.001 | Risk |
| mertk | 51,111 | 0.0367 | 0.0041 | <0.001 | 51,111 | 414 | 0.1976970 | 1.219 | 1.106 | 1.343 | 0.002 | Risk |
| c7 | 42,969 | 0.0299 | 0.0052 | <0.001 | 42,969 | 335 | 0.2416046 | 1.273 | 1.143 | 1.418 | <0.001 | Risk |
| igsf8 | 50,557 | 0.0518 | 0.0050 | <0.001 | 50,557 | 410 | 0.1393598 | 1.150 | 1.046 | 1.263 | 0.037 | Risk |
| tnfrsf19 | 50,987 | 0.0458 | 0.0051 | <0.001 | 50,987 | 416 | 0.1564599 | 1.169 | 1.067 | 1.282 | 0.012 | Risk |
| col5a1 | 42,991 | 0.0471 | 0.0065 | <0.001 | 42,991 | 341 | 0.1513923 | 1.163 | 1.056 | 1.281 | 0.025 | Risk |
| omg | 50,329 | -0.0490 | 0.0110 | <0.001 | 50,329 | 411 | -0.1446104 | 0.865 | 0.784 | 0.955 | 0.038 | Protective |
| ccl14 | 50,429 | 0.0478 | 0.0065 | <0.001 | 50,429 | 412 | 0.1480391 | 1.160 | 1.061 | 1.268 | 0.016 | Risk |
| igfbp2 | 51,099 | 0.0259 | 0.0101 | 0.021 | 51,099 | 413 | 0.2727972 | 1.314 | 1.172 | 1.473 | <0.001 | Risk |
| tnfsf13 | 50,774 | 0.0439 | 0.0044 | <0.001 | 50,774 | 413 | 0.1596743 | 1.173 | 1.061 | 1.297 | 0.022 | Risk |
| yap1 | 43,853 | 0.0431 | 0.0044 | <0.001 | 43,853 | 348 | 0.1610682 | 1.175 | 1.054 | 1.309 | 0.036 | Risk |
| phospho1 | 50,160 | 0.0452 | 0.0048 | <0.001 | 50,160 | 407 | 0.1532698 | 1.166 | 1.054 | 1.289 | 0.030 | Risk |
| fgfbp1 | 50,712 | -0.0473 | 0.0054 | <0.001 | 50,712 | 411 | -0.1448991 | 0.865 | 0.785 | 0.954 | 0.036 | Protective |
| lamb1 | 42,783 | 0.0398 | 0.0049 | <0.001 | 42,783 | 343 | 0.1707484 | 1.186 | 1.064 | 1.323 | 0.025 | Risk |
| ccn3 | 51,098 | 0.0478 | 0.0054 | <0.001 | 51,098 | 411 | 0.1419023 | 1.152 | 1.044 | 1.272 | 0.044 | Risk |
| palm | 43,531 | 0.0458 | 0.0049 | <0.001 | 43,531 | 345 | 0.1481099 | 1.160 | 1.045 | 1.287 | 0.047 | Risk |
| pdcd1lg2 | 50,329 | 0.0352 | 0.0051 | <0.001 | 50,329 | 411 | 0.1912492 | 1.211 | 1.105 | 1.326 | 0.001 | Risk |
| b4galt1 | 50,774 | 0.0352 | 0.0045 | <0.001 | 50,774 | 413 | 0.1872726 | 1.206 | 1.097 | 1.326 | 0.002 | Risk |
| il19 | 49,295 | 0.0485 | 0.0115 | <0.001 | 49,295 | 404 | 0.1357834 | 1.145 | 1.044 | 1.257 | 0.039 | Risk |
| bsg | 51,028 | 0.0366 | 0.0033 | <0.001 | 51,028 | 414 | 0.1774312 | 1.194 | 1.082 | 1.318 | 0.007 | Risk |
| efcab14 | 43,769 | 0.0465 | 0.0044 | <0.001 | 43,769 | 349 | 0.1387870 | 1.149 | 1.057 | 1.249 | 0.015 | Risk |
| gfap | 49,295 | 0.0236 | 0.0076 | 0.004 | 49,295 | 399 | 0.2737629 | 1.315 | 1.196 | 1.445 | <0.001 | Risk |
| vcam1 | 50,679 | 0.0427 | 0.0044 | <0.001 | 50,679 | 414 | 0.1491173 | 1.161 | 1.056 | 1.276 | 0.023 | Risk |
| itgav | 50,987 | -0.0420 | 0.0027 | <0.001 | 50,987 | 416 | -0.1512619 | 0.860 | 0.778 | 0.950 | 0.032 | Protective |
| ephb6 | 50,076 | 0.0407 | 0.0051 | <0.001 | 50,076 | 407 | 0.1557331 | 1.169 | 1.063 | 1.285 | 0.017 | Risk |
| lrg1 | 44,019 | 0.0260 | 0.0042 | <0.001 | 44,019 | 347 | 0.2431622 | 1.275 | 1.151 | 1.413 | <0.001 | Risk |
| fam3c | 50,483 | 0.0276 | 0.0048 | <0.001 | 50,483 | 409 | 0.2281961 | 1.256 | 1.142 | 1.383 | <0.001 | Risk |
| pvr | 50,996 | 0.0437 | 0.0060 | <0.001 | 50,996 | 413 | 0.1422504 | 1.153 | 1.052 | 1.264 | 0.027 | Risk |
| igfbp7 | 51,179 | 0.0289 | 0.0049 | <0.001 | 51,179 | 413 | 0.2147663 | 1.240 | 1.150 | 1.336 | <0.001 | Risk |
| sdc4 | 50,557 | -0.0445 | 0.0076 | <0.001 | 50,557 | 410 | -0.1387987 | 0.870 | 0.792 | 0.957 | 0.038 | Protective |
| eif4ebp1 | 50,557 | 0.0457 | 0.0108 | <0.001 | 50,557 | 410 | 0.1346200 | 1.144 | 1.041 | 1.257 | 0.046 | Risk |
| thbs2 | 50,569 | 0.0350 | 0.0061 | <0.001 | 50,569 | 402 | 0.1718342 | 1.187 | 1.087 | 1.297 | 0.003 | Risk |
| b2m | 44,019 | 0.0536 | 0.0052 | <0.001 | 44,019 | 347 | 0.1121600 | 1.119 | 1.043 | 1.200 | 0.021 | Risk |
| chrdl1 | 50,690 | 0.0325 | 0.0051 | <0.001 | 50,690 | 413 | 0.1820875 | 1.200 | 1.077 | 1.337 | 0.014 | Risk |
| klrb1 | 51,111 | 0.0257 | 0.0061 | <0.001 | 51,111 | 414 | 0.2302252 | 1.259 | 1.145 | 1.384 | <0.001 | Risk |
| ceacam1 | 50,632 | 0.0328 | 0.0038 | <0.001 | 50,632 | 411 | 0.1803260 | 1.198 | 1.077 | 1.331 | 0.012 | Risk |
| tnfsf8 | 43,951 | 0.0397 | 0.0058 | <0.001 | 43,951 | 351 | 0.1485530 | 1.160 | 1.049 | 1.283 | 0.037 | Risk |
| polr2f | 50,329 | 0.0468 | 0.0057 | <0.001 | 50,329 | 411 | 0.1248495 | 1.133 | 1.045 | 1.228 | 0.026 | Risk |
| il1r1 | 50,487 | 0.0294 | 0.0037 | <0.001 | 50,487 | 402 | 0.1966051 | 1.217 | 1.109 | 1.336 | <0.001 | Risk |
| sel1l | 42,783 | 0.0448 | 0.0051 | <0.001 | 42,783 | 343 | 0.1286415 | 1.137 | 1.059 | 1.222 | 0.007 | Risk |
| efna1 | 50,487 | 0.0393 | 0.0045 | <0.001 | 50,487 | 402 | 0.1453190 | 1.156 | 1.052 | 1.271 | 0.029 | Risk |
| ca12 | 50,329 | 0.0276 | 0.0053 | <0.001 | 50,329 | 411 | 0.2049160 | 1.227 | 1.127 | 1.336 | <0.001 | Risk |
| cd4 | 50,407 | 0.0436 | 0.0048 | <0.001 | 50,407 | 408 | 0.1271099 | 1.136 | 1.046 | 1.233 | 0.027 | Risk |
| crip2 | 50,160 | 0.0393 | 0.0060 | <0.001 | 50,160 | 407 | 0.1395380 | 1.150 | 1.045 | 1.265 | 0.039 | Risk |
| igfbpl1 | 50,557 | 0.0244 | 0.0055 | <0.001 | 50,557 | 410 | 0.2239276 | 1.251 | 1.137 | 1.377 | <0.001 | Risk |
| bcan | 50,160 | -0.0357 | 0.0056 | <0.001 | 50,160 | 407 | -0.1506458 | 0.860 | 0.775 | 0.954 | 0.041 | Protective |
| cd276 | 50,526 | 0.0349 | 0.0058 | <0.001 | 50,526 | 413 | 0.1532662 | 1.166 | 1.054 | 1.289 | 0.031 | Risk |
| cd59 | 48,730 | 0.0359 | 0.0036 | <0.001 | 48,730 | 391 | 0.1451270 | 1.156 | 1.049 | 1.274 | 0.035 | Risk |
| wars | 50,569 | 0.0324 | 0.0062 | <0.001 | 50,569 | 402 | 0.1575766 | 1.171 | 1.061 | 1.291 | 0.021 | Risk |
| spon1 | 50,609 | 0.0340 | 0.0053 | <0.001 | 50,609 | 413 | 0.1488743 | 1.161 | 1.055 | 1.276 | 0.025 | Risk |
| esam | 51,179 | 0.0293 | 0.0051 | <0.001 | 51,179 | 413 | 0.1714929 | 1.187 | 1.080 | 1.305 | 0.007 | Risk |
| klrd1 | 51,111 | 0.0326 | 0.0076 | <0.001 | 51,111 | 414 | 0.1501391 | 1.162 | 1.052 | 1.283 | 0.032 | Risk |
| ccn4 | 50,987 | 0.0350 | 0.0066 | <0.001 | 50,987 | 416 | 0.1386646 | 1.149 | 1.046 | 1.262 | 0.038 | Risk |
| atraid | 43,769 | 0.0428 | 0.0047 | <0.001 | 43,769 | 349 | 0.1123294 | 1.119 | 1.044 | 1.199 | 0.018 | Risk |
| igfbp3 | 50,760 | -0.0300 | 0.0048 | <0.001 | 50,760 | 415 | -0.1586005 | 0.853 | 0.780 | 0.934 | 0.009 | Protective |
| tpr | 42,515 | 0.0388 | 0.0082 | <0.001 | 42,515 | 334 | 0.1203923 | 1.128 | 1.038 | 1.226 | 0.041 | Risk |
| cd14 | 49,537 | 0.0322 | 0.0067 | <0.001 | 49,537 | 404 | 0.1414427 | 1.152 | 1.045 | 1.270 | 0.040 | Risk |
| clec14a | 50,569 | 0.0314 | 0.0051 | <0.001 | 50,569 | 402 | 0.1445432 | 1.156 | 1.048 | 1.274 | 0.037 | Risk |
| clec5a | 50,391 | 0.0311 | 0.0048 | <0.001 | 50,391 | 408 | 0.1446678 | 1.156 | 1.049 | 1.273 | 0.035 | Risk |
| thbd | 50,557 | 0.0266 | 0.0046 | <0.001 | 50,557 | 410 | 0.1687878 | 1.184 | 1.078 | 1.300 | 0.007 | Risk |
| f11 | 43,381 | -0.0221 | 0.0050 | <0.001 | 43,381 | 342 | -0.1978365 | 0.821 | 0.727 | 0.926 | 0.017 | Protective |
| tnfrsf21 | 50,996 | 0.0243 | 0.0044 | <0.001 | 50,996 | 413 | 0.1703386 | 1.186 | 1.079 | 1.303 | 0.007 | Risk |
| col4a1 | 50,557 | 0.0201 | 0.0065 | 0.004 | 50,557 | 410 | 0.2054393 | 1.228 | 1.117 | 1.350 | <0.001 | Risk |
| apom | 50,679 | -0.0240 | 0.0044 | <0.001 | 50,679 | 414 | -0.1696529 | 0.844 | 0.765 | 0.931 | 0.011 | Protective |
| anpep | 50,678 | 0.0302 | 0.0046 | <0.001 | 50,678 | 414 | 0.1336111 | 1.143 | 1.043 | 1.252 | 0.038 | Risk |
| ly9 | 49,968 | 0.0199 | 0.0048 | <0.001 | 49,968 | 404 | 0.2011043 | 1.223 | 1.115 | 1.341 | <0.001 | Risk |
| apoc1 | 43,934 | -0.0233 | 0.0060 | <0.001 | 43,934 | 347 | -0.1714452 | 0.842 | 0.750 | 0.947 | 0.038 | Protective |
| chchd6 | 43,748 | 0.0308 | 0.0046 | <0.001 | 43,748 | 350 | 0.1296886 | 1.138 | 1.049 | 1.236 | 0.024 | Risk |
| timd4 | 50,760 | 0.0244 | 0.0071 | 0.002 | 50,760 | 415 | 0.1596881 | 1.173 | 1.065 | 1.293 | 0.017 | Risk |
| mansc1 | 50,712 | 0.0225 | 0.0045 | <0.001 | 50,712 | 411 | 0.1701881 | 1.186 | 1.078 | 1.304 | 0.007 | Risk |
| siglec9 | 50,913 | 0.0242 | 0.0049 | <0.001 | 50,913 | 416 | 0.1548675 | 1.168 | 1.058 | 1.289 | 0.025 | Risk |
| myl4 | 42,783 | 0.0272 | 0.0088 | 0.005 | 42,783 | 343 | 0.1357037 | 1.145 | 1.043 | 1.258 | 0.042 | Risk |
| fbn2 | 42,754 | 0.0257 | 0.0097 | 0.017 | 42,754 | 342 | 0.1420848 | 1.153 | 1.042 | 1.275 | 0.050 | Risk |
| spint2 | 50,774 | 0.0246 | 0.0067 | <0.001 | 50,774 | 413 | 0.1475905 | 1.159 | 1.048 | 1.282 | 0.038 | Risk |
| bmper | 42,991 | 0.0170 | 0.0037 | <0.001 | 42,991 | 341 | 0.2115697 | 1.236 | 1.144 | 1.335 | <0.001 | Risk |
| timp4 | 50,569 | 0.0182 | 0.0065 | 0.012 | 50,569 | 402 | 0.1947970 | 1.215 | 1.105 | 1.336 | 0.001 | Risk |
| ebi3_il27 | 50,910 | 0.0207 | 0.0050 | <0.001 | 50,910 | 416 | 0.1697800 | 1.185 | 1.076 | 1.305 | 0.009 | Risk |
| ggt5 | 50,160 | 0.0185 | 0.0038 | <0.001 | 50,160 | 407 | 0.1875883 | 1.206 | 1.104 | 1.318 | <0.001 | Risk |
| ly96 | 50,160 | 0.0235 | 0.0058 | <0.001 | 50,160 | 407 | 0.1469087 | 1.158 | 1.057 | 1.269 | 0.020 | Risk |
| cfd | 44,019 | 0.0243 | 0.0032 | <0.001 | 44,019 | 347 | 0.1298995 | 1.139 | 1.040 | 1.247 | 0.045 | Risk |
| tnr | 50,160 | -0.0210 | 0.0069 | 0.006 | 50,160 | 407 | -0.1482787 | 0.862 | 0.778 | 0.956 | 0.044 | Protective |
| ncr1 | 50,416 | 0.0217 | 0.0060 | <0.001 | 50,416 | 408 | 0.1423743 | 1.153 | 1.046 | 1.271 | 0.040 | Risk |
| tmod4 | 42,386 | 0.0191 | 0.0066 | 0.009 | 42,386 | 329 | 0.1528171 | 1.165 | 1.062 | 1.278 | 0.016 | Risk |
| clec1a | 49,637 | 0.0158 | 0.0054 | 0.008 | 49,637 | 403 | 0.1762768 | 1.193 | 1.086 | 1.310 | 0.004 | Risk |
| siglec7 | 50,557 | 0.0183 | 0.0046 | <0.001 | 50,557 | 410 | 0.1520904 | 1.164 | 1.053 | 1.288 | 0.032 | Risk |
| hspa2 | 43,748 | 0.0215 | 0.0056 | <0.001 | 43,748 | 350 | 0.1285277 | 1.137 | 1.044 | 1.239 | 0.034 | Risk |
| stab2 | 43,951 | 0.0158 | 0.0046 | 0.002 | 43,951 | 351 | 0.1578960 | 1.171 | 1.049 | 1.307 | 0.044 | Risk |
| draxin | 50,160 | 0.0165 | 0.0070 | 0.035 | 50,160 | 407 | 0.1382151 | 1.148 | 1.041 | 1.266 | 0.049 | Risk |
| fnta | 43,748 | 0.0139 | 0.0058 | 0.032 | 43,748 | 350 | 0.1405541 | 1.151 | 1.054 | 1.257 | 0.022 | Risk |
| nfasc | 51,111 | 0.0125 | 0.0046 | 0.015 | 51,111 | 414 | 0.1551095 | 1.168 | 1.058 | 1.289 | 0.025 | Risk |
| gfra2 | 50,329 | 0.0113 | 0.0034 | 0.002 | 50,329 | 411 | 0.1529400 | 1.165 | 1.061 | 1.280 | 0.018 | Risk |
| ifngr1 | 50,774 | 0.0126 | 0.0033 | <0.001 | 50,774 | 413 | 0.1366180 | 1.146 | 1.046 | 1.256 | 0.035 | Risk |
| pcdh1 | 50,950 | 0.0130 | 0.0034 | <0.001 | 50,950 | 413 | 0.1319264 | 1.141 | 1.050 | 1.240 | 0.022 | Risk |
| serpina1 | 43,203 | 0.0118 | 0.0013 | <0.001 | 43,203 | 340 | 0.1193095 | 1.127 | 1.036 | 1.225 | 0.047 | Risk |
| ddr1 | 50,996 | 0.0100 | 0.0038 | 0.017 | 50,996 | 413 | 0.1383692 | 1.148 | 1.046 | 1.261 | 0.037 | Risk |

**Supplementary Table S8. Gene Ontology Biological Process enrichment analysis**

GO Biological Process enrichment of the 85 candidate mediator proteins. In total, 431 terms met FDR < 0.05. Gene-ratio denominators reflect proteins eligible for the respective ontology analysis.

| **ID** | **Description** | **Gene ratio** | **Background ratio** | **Rich factor** | **Fold enrichment** | **z score** | **P value** | **Adjusted P** | **q value** | **Gene IDs** | **Count** |
| --- | --- | --- | --- | --- | --- | --- | --- | --- | --- | --- | --- |
| GO:0046718 | symbiont entry into host cell | 13/81 | 131/18805 | 0.099236641221374 | 23.038827631703 | 16.6484487412597 | 1.245 × 10⁻¹⁴ | 1.426 × 10⁻¹¹ | 1.016 × 10⁻¹¹ | ACE2/CD74/CD80/CTSL/EGFR/EPHA2/HAVCR1/LGALS9/NECTIN2/SCARB2/SIGLEC1/TNFRSF14/TNFRSF4 | 13 |
| GO:0044409 | symbiont entry into host | 13/81 | 136/18805 | 0.0955882352941176 | 22.1918119099492 | 16.3134317929188 | 2.039 × 10⁻¹⁴ | 1.426 × 10⁻¹¹ | 1.016 × 10⁻¹¹ | ACE2/CD74/CD80/CTSL/EGFR/EPHA2/HAVCR1/LGALS9/NECTIN2/SCARB2/SIGLEC1/TNFRSF14/TNFRSF4 | 13 |
| GO:0051701 | biological process involved in interaction with host | 14/81 | 176/18805 | 0.0795454545454545 | 18.4673120089787 | 15.3128474112279 | 2.481 × 10⁻¹⁴ | 1.426 × 10⁻¹¹ | 1.016 × 10⁻¹¹ | ACE2/CD74/CD80/CTSL/EGFR/EPHA2/HAVCR1/INHBB/LGALS9/NECTIN2/SCARB2/SIGLEC1/TNFRSF14/TNFRSF4 | 14 |
| GO:0044403 | biological process involved in symbiotic interaction | 15/81 | 220/18805 | 0.0681818181818182 | 15.8291245791246 | 14.5517051328645 | 2.684 × 10⁻¹⁴ | 1.426 × 10⁻¹¹ | 1.016 × 10⁻¹¹ | ACE2/BST2/CD74/CD80/CTSL/EGFR/EPHA2/HAVCR1/INHBB/LGALS9/NECTIN2/SCARB2/SIGLEC1/TNFRSF14/TNFRSF4 | 15 |
| GO:0070663 | regulation of leukocyte proliferation | 14/81 | 273/18805 | 0.0512820512820513 | 11.9056663501108 | 11.9382508148889 | 1.013 × 10⁻¹¹ | 4.305 × 10⁻⁹ | 3.069 × 10⁻⁹ | BST2/CD300A/CD74/CD80/CSF1/HAVCR2/IL2RA/LGALS9/LILRB4/TNFRSF14/TNFRSF1B/TNFRSF4/TNFRSF9/VSIG4 | 14 |
| GO:0019058 | viral life cycle | 14/81 | 295/18805 | 0.0474576271186441 | 11.0177861477296 | 11.4063808841209 | 2.856 × 10⁻¹¹ | 1.012 × 10⁻⁸ | 7.211 × 10⁻⁹ | ACE2/BST2/CD74/CD80/CTSL/EGFR/EPHA2/HAVCR1/LGALS9/NECTIN2/SCARB2/SIGLEC1/TNFRSF14/TNFRSF4 | 14 |
| GO:0032944 | regulation of mononuclear cell proliferation | 13/81 | 248/18805 | 0.0524193548387097 | 12.169703305456 | 11.6461373675952 | 4.517 × 10⁻¹¹ | 1.371 × 10⁻⁸ | 9.775 × 10⁻⁹ | CD300A/CD74/CD80/CSF1/HAVCR2/IL2RA/LGALS9/LILRB4/TNFRSF14/TNFRSF1B/TNFRSF4/TNFRSF9/VSIG4 | 13 |
| GO:0070661 | leukocyte proliferation | 14/81 | 344/18805 | 0.0406976744186047 | 9.44839219064025 | 10.4014525219537 | 2.178 × 10⁻¹⁰ | 5.786 × 10⁻⁸ | 4.124 × 10⁻⁸ | BST2/CD300A/CD74/CD80/CSF1/HAVCR2/IL2RA/LGALS9/LILRB4/TNFRSF14/TNFRSF1B/TNFRSF4/TNFRSF9/VSIG4 | 14 |
| GO:0050868 | negative regulation of T cell activation | 10/81 | 136/18805 | 0.0735294117647059 | 17.0706245461147 | 12.3711478054204 | 3.403 × 10⁻¹⁰ | 8.034 × 10⁻⁸ | 5.727 × 10⁻⁸ | CD300A/CD74/CD80/HAVCR2/IL2RA/IL4R/LGALS9/LILRB4/TNFRSF14/VSIG4 | 10 |
| GO:0050670 | regulation of lymphocyte proliferation | 12/81 | 243/18805 | 0.0493827160493827 | 11.4647157445511 | 10.7990736502967 | 5.315 × 10⁻¹⁰ | 1.129 × 10⁻⁷ | 8.051 × 10⁻⁸ | CD300A/CD74/CD80/HAVCR2/IL2RA/LGALS9/LILRB4/TNFRSF14/TNFRSF1B/TNFRSF4/TNFRSF9/VSIG4 | 12 |
| GO:0032943 | mononuclear cell proliferation | 13/81 | 308/18805 | 0.0422077922077922 | 9.79898188231522 | 10.2406010625898 | 6.564 × 10⁻¹⁰ | 1.268 × 10⁻⁷ | 9.039 × 10⁻⁸ | CD300A/CD74/CD80/CSF1/HAVCR2/IL2RA/LGALS9/LILRB4/TNFRSF14/TNFRSF1B/TNFRSF4/TNFRSF9/VSIG4 | 13 |
| GO:1903038 | negative regulation of leukocyte cell-cell adhesion | 10/81 | 155/18805 | 0.0645161290322581 | 14.9780963759458 | 11.4932479480284 | 1.23 × 10⁻⁹ | 2.177 × 10⁻⁷ | 1.552 × 10⁻⁷ | CD300A/CD74/CD80/HAVCR2/IL2RA/IL4R/LGALS9/LILRB4/TNFRSF14/VSIG4 | 10 |
| GO:0050777 | negative regulation of immune response | 11/81 | 214/18805 | 0.0514018691588785 | 11.9334833275643 | 10.5799197306522 | 1.935 × 10⁻⁹ | 3.163 × 10⁻⁷ | 2.255 × 10⁻⁷ | AMBP/CD300A/CD80/HAVCR2/IL4R/LGALS9/LILRB4/MMP12/NECTIN2/TNFRSF14/VSIG4 | 11 |
| GO:0002697 | regulation of immune effector process | 14/81 | 414/18805 | 0.0338164251207729 | 7.85083795550784 | 9.27063554376049 | 2.408 × 10⁻⁹ | 3.654 × 10⁻⁷ | 2.605 × 10⁻⁷ | BST2/CD300A/CD74/CD80/HAVCR2/IL4R/LGALS9/LILRB4/NECTIN2/SPON2/TNFRSF14/TNFRSF1B/TNFRSF4/VSIG4 | 14 |
| GO:0016032 | viral process | 14/81 | 421/18805 | 0.0332541567695962 | 7.72030145743527 | 9.17229706081234 | 2.985 × 10⁻⁹ | 4.228 × 10⁻⁷ | 3.014 × 10⁻⁷ | ACE2/BST2/CD74/CD80/CTSL/EGFR/EPHA2/HAVCR1/LGALS9/NECTIN2/SCARB2/SIGLEC1/TNFRSF14/TNFRSF4 | 14 |
| GO:0051250 | negative regulation of lymphocyte activation | 10/81 | 173/18805 | 0.0578034682080925 | 13.4196817241133 | 10.7937435329411 | 3.577 × 10⁻⁹ | 4.751 × 10⁻⁷ | 3.387 × 10⁻⁷ | CD300A/CD74/CD80/HAVCR2/IL2RA/IL4R/LGALS9/LILRB4/TNFRSF14/VSIG4 | 10 |
| GO:0050672 | negative regulation of lymphocyte proliferation | 8/81 | 91/18805 | 0.0879120879120879 | 20.4097137430471 | 12.2074464671318 | 5.297 × 10⁻⁹ | 6.621 × 10⁻⁷ | 4.72 × 10⁻⁷ | CD300A/CD80/HAVCR2/IL2RA/LGALS9/LILRB4/TNFRSF14/VSIG4 | 8 |
| GO:0046651 | lymphocyte proliferation | 12/81 | 300/18805 | 0.04 | 9.28641975308642 | 9.51593191474871 | 5.783 × 10⁻⁹ | 6.698 × 10⁻⁷ | 4.774 × 10⁻⁷ | CD300A/CD74/CD80/HAVCR2/IL2RA/LGALS9/LILRB4/TNFRSF14/TNFRSF1B/TNFRSF4/TNFRSF9/VSIG4 | 12 |
| GO:0002698 | negative regulation of immune effector process | 9/81 | 134/18805 | 0.0671641791044776 | 15.5928689883914 | 11.150070353689 | 6.108 × 10⁻⁹ | 6.698 × 10⁻⁷ | 4.774 × 10⁻⁷ | BST2/CD300A/CD80/HAVCR2/IL4R/LGALS9/LILRB4/NECTIN2/VSIG4 | 9 |
| GO:0032945 | negative regulation of mononuclear cell proliferation | 8/81 | 93/18805 | 0.0860215053763441 | 19.9707951679278 | 12.0624414749993 | 6.304 × 10⁻⁹ | 6.698 × 10⁻⁷ | 4.774 × 10⁻⁷ | CD300A/CD80/HAVCR2/IL2RA/LGALS9/LILRB4/TNFRSF14/VSIG4 | 8 |
| GO:0070664 | negative regulation of leukocyte proliferation | 8/81 | 100/18805 | 0.08 | 18.5728395061728 | 11.5886114231917 | 1.124 × 10⁻⁸ | 1.138 × 10⁻⁶ | 8.11 × 10⁻⁷ | CD300A/CD80/HAVCR2/IL2RA/LGALS9/LILRB4/TNFRSF14/VSIG4 | 8 |
| GO:0050863 | regulation of T cell activation | 13/81 | 395/18805 | 0.0329113924050633 | 7.64072511329895 | 8.77314831518021 | 1.312 × 10⁻⁸ | 1.268 × 10⁻⁶ | 9.036 × 10⁻⁷ | CD27/CD300A/CD74/CD80/HAVCR2/IL2RA/IL4R/LGALS9/LILRB4/TNFRSF14/TNFRSF1B/TNFRSF9/VSIG4 | 13 |
| GO:0002695 | negative regulation of leukocyte activation | 10/81 | 204/18805 | 0.0490196078431373 | 11.3804163640765 | 9.8046032752036 | 1.741 × 10⁻⁸ | 1.608 × 10⁻⁶ | 1.146 × 10⁻⁶ | CD300A/CD74/CD80/HAVCR2/IL2RA/IL4R/LGALS9/LILRB4/TNFRSF14/VSIG4 | 10 |
| GO:0022408 | negative regulation of cell-cell adhesion | 10/81 | 209/18805 | 0.0478468899521531 | 11.1081575993857 | 9.66504083538156 | 2.191 × 10⁻⁸ | 1.94 × 10⁻⁶ | 1.383 × 10⁻⁶ | CD300A/CD74/CD80/HAVCR2/IL2RA/IL4R/LGALS9/LILRB4/TNFRSF14/VSIG4 | 10 |
| GO:0042742 | defense response to bacterium | 12/81 | 343/18805 | 0.0349854227405248 | 8.12223302019221 | 8.75572634889682 | 2.562 × 10⁻⁸ | 2.178 × 10⁻⁶ | 1.552 × 10⁻⁶ | ADM/COLEC12/EPHA2/HAVCR2/LGALS4/MMP7/RNASE1/RNASE6/SPON2/TNFRSF14/TNFRSF1A/WFDC2 | 12 |
| GO:0042098 | T cell proliferation | 10/81 | 217/18805 | 0.0460829493087558 | 10.6986402685327 | 9.45132507306393 | 3.129 × 10⁻⁸ | 2.557 × 10⁻⁶ | 1.823 × 10⁻⁶ | CD80/HAVCR2/IL2RA/LGALS9/LILRB4/TNFRSF14/TNFRSF1B/TNFRSF4/TNFRSF9/VSIG4 | 10 |
| GO:0042130 | negative regulation of T cell proliferation | 7/81 | 75/18805 | 0.0933333333333333 | 21.6683127572016 | 11.7960086469601 | 3.312 × 10⁻⁸ | 2.607 × 10⁻⁶ | 1.858 × 10⁻⁶ | CD80/HAVCR2/IL2RA/LGALS9/LILRB4/TNFRSF14/VSIG4 | 7 |
| GO:0002704 | negative regulation of leukocyte mediated immunity | 7/81 | 76/18805 | 0.0921052631578947 | 21.3832033788174 | 11.7108994888188 | 3.635 × 10⁻⁸ | 2.759 × 10⁻⁶ | 1.967 × 10⁻⁶ | BST2/CD300A/CD80/HAVCR2/LGALS9/LILRB4/NECTIN2 | 7 |
| GO:0050866 | negative regulation of cell activation | 10/81 | 227/18805 | 0.0440528634361234 | 10.2273345298309 | 9.19936879480035 | 4.788 × 10⁻⁸ | 3.509 × 10⁻⁶ | 2.501 × 10⁻⁶ | CD300A/CD74/CD80/HAVCR2/IL2RA/IL4R/LGALS9/LILRB4/TNFRSF14/VSIG4 | 10 |
| GO:0010720 | positive regulation of cell development | 13/81 | 447/18805 | 0.029082774049217 | 6.75187118512995 | 8.09503152893119 | 5.617 × 10⁻⁸ | 3.869 × 10⁻⁶ | 2.758 × 10⁻⁶ | BAIAP2/CD27/CD74/CD80/CSF1/EGFR/IL2RA/IL4R/LGALS9/LILRB4/NEFL/TNFRSF11A/TNFRSF1B | 13 |
| GO:0002440 | production of molecular mediator of immune response | 10/81 | 231/18805 | 0.0432900432900433 | 10.0502378280156 | 9.10293781282484 | 5.644 × 10⁻⁸ | 3.869 × 10⁻⁶ | 2.758 × 10⁻⁶ | BST2/CD74/IL4R/LGALS4/LILRB4/MMP7/SPON2/TNFRSF14/TNFRSF1B/TNFRSF4 | 10 |
| GO:0002696 | positive regulation of leukocyte activation | 12/81 | 372/18805 | 0.032258064516129 | 7.48904818797292 | 8.31424412315371 | 6.238 × 10⁻⁸ | 4.142 × 10⁻⁶ | 2.953 × 10⁻⁶ | CD27/CD74/CD80/HAVCR1/HAVCR2/IL2RA/IL4R/LGALS9/LILRB4/NECTIN2/TNFRSF14/TNFRSF4 | 12 |
| GO:0007162 | negative regulation of cell adhesion | 11/81 | 312/18805 | 0.0352564102564103 | 8.18514561570117 | 8.41739098951993 | 9.495 × 10⁻⁸ | 6.012 × 10⁻⁶ | 4.285 × 10⁻⁶ | CD300A/CD74/CD80/HAVCR2/IL2RA/IL4R/LGALS9/LILRB4/MMP12/TNFRSF14/VSIG4 | 11 |
| GO:0042129 | regulation of T cell proliferation | 9/81 | 184/18805 | 0.0489130434782609 | 11.3556763285024 | 9.28440750190529 | 9.619 × 10⁻⁸ | 6.012 × 10⁻⁶ | 4.285 × 10⁻⁶ | CD80/HAVCR2/IL2RA/LGALS9/LILRB4/TNFRSF14/TNFRSF1B/TNFRSF9/VSIG4 | 9 |
| GO:0019730 | antimicrobial humoral response | 9/81 | 185/18805 | 0.0486486486486487 | 11.2942942942943 | 9.25466963047305 | 1.008 × 10⁻⁷ | 6.118 × 10⁻⁶ | 4.361 × 10⁻⁶ | ADM/CCL11/CXCL9/LGALS4/MMP7/REG1B/RNASE6/SPON2/WFDC2 | 9 |
| GO:0050867 | positive regulation of cell activation | 12/81 | 392/18805 | 0.0306122448979592 | 7.10695389266818 | 8.03662515684603 | 1.102 × 10⁻⁷ | 6.505 × 10⁻⁶ | 4.637 × 10⁻⁶ | CD27/CD74/CD80/HAVCR1/HAVCR2/IL2RA/IL4R/LGALS9/LILRB4/NECTIN2/TNFRSF14/TNFRSF4 | 12 |
| GO:0002274 | myeloid leukocyte activation | 10/81 | 251/18805 | 0.0398406374501992 | 9.24942206482711 | 8.65386115633255 | 1.228 × 10⁻⁷ | 7.051 × 10⁻⁶ | 5.026 × 10⁻⁶ | CD300A/CD74/CSF1/HAVCR1/HAVCR2/IL4R/LGALS9/LILRB4/NECTIN2/VSIG4 | 10 |
| GO:1902107 | positive regulation of leukocyte differentiation | 9/81 | 195/18805 | 0.0461538461538462 | 10.7150997150997 | 8.96932366420367 | 1.579 × 10⁻⁷ | 8.606 × 10⁻⁶ | 6.134 × 10⁻⁶ | CD27/CD74/CD80/CSF1/IL2RA/IL4R/LGALS9/LILRB4/TNFRSF11A | 9 |
| GO:1903708 | positive regulation of hemopoiesis | 9/81 | 195/18805 | 0.0461538461538462 | 10.7150997150997 | 8.96932366420367 | 1.579 × 10⁻⁷ | 8.606 × 10⁻⁶ | 6.134 × 10⁻⁶ | CD27/CD74/CD80/CSF1/IL2RA/IL4R/LGALS9/LILRB4/TNFRSF11A | 9 |
| GO:0046631 | alpha-beta T cell activation | 9/81 | 201/18805 | 0.0447761194029851 | 10.3952459922609 | 8.80787886859116 | 2.044 × 10⁻⁷ | 1.068 × 10⁻⁵ | 7.615 × 10⁻⁶ | CD300A/CD80/CTSL/IL2RA/IL4R/LGALS9/LILRB4/NCR3LG1/TNFRSF14 | 9 |
| GO:0022409 | positive regulation of cell-cell adhesion | 11/81 | 337/18805 | 0.0326409495548961 | 7.57793896765212 | 8.01425527254832 | 2.061 × 10⁻⁷ | 1.068 × 10⁻⁵ | 7.615 × 10⁻⁶ | CD27/CD74/CD80/FSTL3/HAVCR2/IL2RA/IL4R/LGALS9/LILRB4/PLAUR/TNFRSF14 | 11 |
| GO:0060603 | mammary gland duct morphogenesis | 5/81 | 32/18805 | 0.15625 | 36.2750771604938 | 13.1354342822464 | 2.405 × 10⁻⁷ | 1.217 × 10⁻⁵ | 8.674 × 10⁻⁶ | AREG/CCL11/CSF1/EPHA2/FGFR2 | 5 |
| GO:0032496 | response to lipopolysaccharide | 11/81 | 357/18805 | 0.030812324929972 | 7.15340457170523 | 7.72046124777823 | 3.662 × 10⁻⁷ | 1.81 × 10⁻⁵ | 1.29 × 10⁻⁵ | ADM/CD80/CXCL9/FGFR2/HAVCR2/IL18BP/LGALS9/REN/SPON2/TNFRSF11A/TNFRSF1B | 11 |
| GO:0071356 | cellular response to tumor necrosis factor | 9/81 | 219/18805 | 0.0410958904109589 | 9.54084221207509 | 8.36176781333098 | 4.218 × 10⁻⁷ | 2.037 × 10⁻⁵ | 1.452 × 10⁻⁵ | CHI3L1/EDA2R/IL18BP/KRT18/TNFRSF11A/TNFRSF14/TNFRSF1A/TNFRSF1B/TNFRSF4 | 9 |
| GO:0070665 | positive regulation of leukocyte proliferation | 8/81 | 168/18805 | 0.0476190476190476 | 11.0552616108172 | 8.6104991257389 | 6.346 × 10⁻⁷ | 2.997 × 10⁻⁵ | 2.136 × 10⁻⁵ | BST2/CD74/CD80/CSF1/HAVCR2/IL2RA/LGALS9/TNFRSF4 | 8 |
| GO:0002237 | response to molecule of bacterial origin | 11/81 | 379/18805 | 0.029023746701847 | 6.73816736701521 | 7.4224212296025 | 6.613 × 10⁻⁷ | 3.023 × 10⁻⁵ | 2.155 × 10⁻⁵ | ADM/CD80/CXCL9/FGFR2/HAVCR2/IL18BP/LGALS9/REN/SPON2/TNFRSF11A/TNFRSF1B | 11 |
| GO:0043410 | positive regulation of MAPK cascade | 12/81 | 464/18805 | 0.0258620689655172 | 6.00415070242657 | 7.17870620030324 | 6.686 × 10⁻⁷ | 3.023 × 10⁻⁵ | 2.155 × 10⁻⁵ | CD27/CD74/CHI3L1/EDA2R/EGFR/FGFR2/GDF15/GPR37/HAVCR2/IGFBP4/LGALS9/TNFRSF11A | 12 |
| GO:0033209 | tumor necrosis factor-mediated signaling pathway | 7/81 | 118/18805 | 0.0593220338983051 | 13.7722326846621 | 9.15390386764029 | 7.585 × 10⁻⁷ | 3.358 × 10⁻⁵ | 2.394 × 10⁻⁵ | EDA2R/KRT18/TNFRSF11A/TNFRSF14/TNFRSF1A/TNFRSF1B/TNFRSF4 | 7 |
| GO:0006959 | humoral immune response | 10/81 | 309/18805 | 0.0323624595469256 | 7.51328458987574 | 7.59291783625809 | 8.315 × 10⁻⁷ | 3.606 × 10⁻⁵ | 2.57 × 10⁻⁵ | ADM/CCL11/CXCL9/LGALS4/MMP7/REG1B/RNASE6/SPON2/VSIG4/WFDC2 | 10 |
| GO:0034612 | response to tumor necrosis factor | 9/81 | 241/18805 | 0.037344398340249 | 8.66989396035039 | 7.88189083526216 | 9.395 × 10⁻⁷ | 3.961 × 10⁻⁵ | 2.823 × 10⁻⁵ | CHI3L1/EDA2R/IL18BP/KRT18/TNFRSF11A/TNFRSF14/TNFRSF1A/TNFRSF1B/TNFRSF4 | 9 |
| GO:0045582 | positive regulation of T cell differentiation | 7/81 | 122/18805 | 0.0573770491803279 | 13.3206840720502 | 8.97965692487688 | 9.505 × 10⁻⁷ | 3.961 × 10⁻⁵ | 2.823 × 10⁻⁵ | CD27/CD74/CD80/IL2RA/IL4R/LGALS9/LILRB4 | 7 |
| GO:1903037 | regulation of leukocyte cell-cell adhesion | 11/81 | 395/18805 | 0.0278481012658228 | 6.46522894202219 | 7.22018503502806 | 9.921 × 10⁻⁷ | 4.012 × 10⁻⁵ | 2.86 × 10⁻⁵ | CD27/CD300A/CD74/CD80/HAVCR2/IL2RA/IL4R/LGALS9/LILRB4/TNFRSF14/VSIG4 | 11 |
| GO:0046634 | regulation of alpha-beta T cell activation | 7/81 | 123/18805 | 0.0569105691056911 | 13.212385827562 | 8.93736928246878 | 1.004 × 10⁻⁶ | 4.012 × 10⁻⁵ | 2.86 × 10⁻⁵ | CD300A/CD80/IL2RA/IL4R/LGALS9/LILRB4/TNFRSF14 | 7 |
| GO:0002443 | leukocyte mediated immunity | 12/81 | 483/18805 | 0.0248447204968944 | 5.76796257955678 | 6.98213559552029 | 1.02 × 10⁻⁶ | 4.012 × 10⁻⁵ | 2.86 × 10⁻⁵ | BST2/CD27/CD300A/CD74/CD80/HAVCR2/IL4R/LGALS9/LILRB4/NECTIN2/SPON2/TNFRSF1B | 12 |
| GO:0070371 | ERK1 and ERK2 cascade | 10/81 | 319/18805 | 0.0313479623824451 | 7.27775842718371 | 7.43783913707394 | 1.11 × 10⁻⁶ | 4.287 × 10⁻⁵ | 3.056 × 10⁻⁵ | ACE2/CCL11/CD74/CHI3L1/EGFR/EPHA2/FGFR2/HAVCR2/LGALS9/TNFRSF11A | 10 |
| GO:0019731 | antibacterial humoral response | 6/81 | 79/18805 | 0.0759493670886076 | 17.6324425691514 | 9.74350883111112 | 1.145 × 10⁻⁶ | 4.345 × 10⁻⁵ | 3.097 × 10⁻⁵ | ADM/LGALS4/MMP7/RNASE6/SPON2/WFDC2 | 6 |
| GO:0043434 | response to peptide hormone | 12/81 | 494/18805 | 0.0242914979757085 | 5.63952616584196 | 6.87304814329915 | 1.29 × 10⁻⁶ | 4.81 × 10⁻⁵ | 3.429 × 10⁻⁵ | ADM/AREG/BAIAP2/EGFR/EPHA2/FGFR2/GDF15/INHBB/NEFL/REG1B/SPINK1/TNFRSF11A | 12 |
| GO:0045785 | positive regulation of cell adhesion | 12/81 | 499/18805 | 0.0240480961923848 | 5.58301788762711 | 6.8245404239929 | 1.433 × 10⁻⁶ | 5.251 × 10⁻⁵ | 3.743 × 10⁻⁵ | CD27/CD74/CD80/CSF1/FSTL3/HAVCR2/IL2RA/IL4R/LGALS9/LILRB4/PLAUR/TNFRSF14 | 12 |
| GO:0050830 | defense response to Gram-positive bacterium | 7/81 | 131/18805 | 0.0534351145038168 | 12.405522570917 | 8.61589650150185 | 1.535 × 10⁻⁶ | 5.53 × 10⁻⁵ | 3.942 × 10⁻⁵ | ADM/EPHA2/HAVCR2/MMP7/RNASE1/RNASE6/TNFRSF14 | 7 |
| GO:0060443 | mammary gland morphogenesis | 5/81 | 46/18805 | 0.108695652173913 | 25.2348362855609 | 10.8238677904285 | 1.562 × 10⁻⁶ | 5.531 × 10⁻⁵ | 3.943 × 10⁻⁵ | AREG/CCL11/CSF1/EPHA2/FGFR2 | 5 |
| GO:0002703 | regulation of leukocyte mediated immunity | 9/81 | 260/18805 | 0.0346153846153846 | 8.03632478632479 | 7.51428126969624 | 1.761 × 10⁻⁶ | 6.135 × 10⁻⁵ | 4.373 × 10⁻⁵ | BST2/CD300A/CD80/HAVCR2/IL4R/LGALS9/LILRB4/NECTIN2/TNFRSF1B | 9 |
| GO:0050870 | positive regulation of T cell activation | 9/81 | 261/18805 | 0.0344827586206897 | 8.00553426990209 | 7.49597484951988 | 1.818 × 10⁻⁶ | 6.136 × 10⁻⁵ | 4.374 × 10⁻⁵ | CD27/CD74/CD80/HAVCR2/IL2RA/IL4R/LGALS9/LILRB4/TNFRSF14 | 9 |
| GO:0051251 | positive regulation of lymphocyte activation | 10/81 | 337/18805 | 0.029673590504451 | 6.88903542513829 | 7.17492719805277 | 1.819 × 10⁻⁶ | 6.136 × 10⁻⁵ | 4.374 × 10⁻⁵ | CD27/CD74/CD80/HAVCR2/IL2RA/IL4R/LGALS9/LILRB4/TNFRSF14/TNFRSF4 | 10 |
| GO:1902105 | regulation of leukocyte differentiation | 10/81 | 340/18805 | 0.0294117647058824 | 6.8282498184459 | 7.13298442645777 | 1.97 × 10⁻⁶ | 6.452 × 10⁻⁵ | 4.599 × 10⁻⁵ | CD27/CD74/CD80/CSF1/FSTL3/IL2RA/IL4R/LGALS9/LILRB4/TNFRSF11A | 10 |
| GO:0061844 | antimicrobial humoral immune response mediated by antimicrobial peptide | 7/81 | 136/18805 | 0.0514705882352941 | 11.9494371822803 | 8.42886381792199 | 1.973 × 10⁻⁶ | 6.452 × 10⁻⁵ | 4.599 × 10⁻⁵ | ADM/CCL11/CXCL9/LGALS4/MMP7/REG1B/RNASE6 | 7 |
| GO:0045621 | positive regulation of lymphocyte differentiation | 7/81 | 137/18805 | 0.0510948905109489 | 11.8622150130666 | 8.39263038774531 | 2.073 × 10⁻⁶ | 6.673 × 10⁻⁵ | 4.757 × 10⁻⁵ | CD27/CD74/CD80/IL2RA/IL4R/LGALS9/LILRB4 | 7 |
| GO:0002700 | regulation of production of molecular mediator of immune response | 8/81 | 197/18805 | 0.0406091370558376 | 9.42783731277809 | 7.82109790499906 | 2.104 × 10⁻⁶ | 6.674 × 10⁻⁵ | 4.758 × 10⁻⁵ | BST2/CD74/IL4R/LILRB4/SPON2/TNFRSF14/TNFRSF1B/TNFRSF4 | 8 |
| GO:0002699 | positive regulation of immune effector process | 9/81 | 270/18805 | 0.0333333333333333 | 7.73868312757202 | 7.33548674092201 | 2.402 × 10⁻⁶ | 7.453 × 10⁻⁵ | 5.312 × 10⁻⁵ | CD300A/CD74/CD80/IL4R/LGALS9/NECTIN2/SPON2/TNFRSF14/TNFRSF4 | 9 |
| GO:0007159 | leukocyte cell-cell adhesion | 11/81 | 433/18805 | 0.0254041570438799 | 5.8978416445699 | 6.78170327466023 | 2.42 × 10⁻⁶ | 7.453 × 10⁻⁵ | 5.312 × 10⁻⁵ | CD27/CD300A/CD74/CD80/HAVCR2/IL2RA/IL4R/LGALS9/LILRB4/TNFRSF14/VSIG4 | 11 |
| GO:0060444 | branching involved in mammary gland duct morphogenesis | 4/81 | 23/18805 | 0.173913043478261 | 40.3757380568975 | 12.427678842533 | 2.657 × 10⁻⁶ | 8.067 × 10⁻⁵ | 5.751 × 10⁻⁵ | AREG/CCL11/CSF1/EPHA2 | 4 |
| GO:1903039 | positive regulation of leukocyte cell-cell adhesion | 9/81 | 285/18805 | 0.0315789473684211 | 7.33138401559454 | 7.08384269743692 | 3.736 × 10⁻⁶ | 1.118 × 10⁻⁴ | 7.971 × 10⁻⁵ | CD27/CD74/CD80/HAVCR2/IL2RA/IL4R/LGALS9/LILRB4/TNFRSF14 | 9 |
| GO:0032946 | positive regulation of mononuclear cell proliferation | 7/81 | 150/18805 | 0.0466666666666667 | 10.8341563786008 | 7.95341103070797 | 3.79 × 10⁻⁶ | 1.118 × 10⁻⁴ | 7.973 × 10⁻⁵ | CD74/CD80/CSF1/HAVCR2/IL2RA/LGALS9/TNFRSF4 | 7 |
| GO:0070372 | regulation of ERK1 and ERK2 cascade | 9/81 | 296/18805 | 0.0304054054054054 | 7.05893393393393 | 6.91065017911809 | 5.081 × 10⁻⁶ | 1.479 × 10⁻⁴ | 1.054 × 10⁻⁴ | ACE2/CD74/CHI3L1/EGFR/EPHA2/FGFR2/HAVCR2/LGALS9/TNFRSF11A | 9 |
| GO:0032602 | chemokine production | 6/81 | 105/18805 | 0.0571428571428571 | 13.2663139329806 | 8.29003255769811 | 6.066 × 10⁻⁶ | 1.719 × 10⁻⁴ | 1.225 × 10⁻⁴ | CD74/EPHA2/HAVCR2/IL4R/LGALS9/LILRB4 | 6 |
| GO:0032642 | regulation of chemokine production | 6/81 | 105/18805 | 0.0571428571428571 | 13.2663139329806 | 8.29003255769811 | 6.066 × 10⁻⁶ | 1.719 × 10⁻⁴ | 1.225 × 10⁻⁴ | CD74/EPHA2/HAVCR2/IL4R/LGALS9/LILRB4 | 6 |
| GO:0050900 | leukocyte migration | 10/81 | 395/18805 | 0.0253164556962025 | 5.87748085638381 | 6.44370339495198 | 7.431 × 10⁻⁶ | 2.078 × 10⁻⁴ | 1.481 × 10⁻⁴ | CCL11/CD300A/CD74/CSF1/CXCL9/GDF15/LGALS9/NBL1/TNFRSF11A/TNFRSF14 | 10 |
| GO:0002507 | tolerance induction | 4/81 | 30/18805 | 0.133333333333333 | 30.9547325102881 | 10.7995190474049 | 8.038 × 10⁻⁶ | 2.218 × 10⁻⁴ | 1.581 × 10⁻⁴ | HAVCR2/IL2RA/LGALS9/LILRB4 | 4 |
| GO:0002707 | negative regulation of lymphocyte mediated immunity | 5/81 | 65/18805 | 0.0769230769230769 | 17.8584995251662 | 8.95486653760561 | 8.827 × 10⁻⁶ | 2.405 × 10⁻⁴ | 1.714 × 10⁻⁴ | CD80/HAVCR2/LGALS9/LILRB4/NECTIN2 | 5 |
| GO:0044650 | adhesion of symbiont to host cell | 3/81 | 10/18805 | 0.3 | 69.6481481481482 | 14.2815707488821 | 9.04 × 10⁻⁶ | 2.432 × 10⁻⁴ | 1.733 × 10⁻⁴ | ACE2/INHBB/NECTIN2 | 3 |
| GO:0002820 | negative regulation of adaptive immune response | 5/81 | 67/18805 | 0.0746268656716418 | 17.3254099871015 | 8.8045709040776 | 1.025 × 10⁻⁵ | 2.723 × 10⁻⁴ | 1.941 × 10⁻⁴ | CD80/HAVCR2/IL4R/LILRB4/TNFRSF14 | 5 |
| GO:0051048 | negative regulation of secretion | 7/81 | 175/18805 | 0.04 | 9.28641975308642 | 7.24348958996204 | 1.044 × 10⁻⁵ | 2.725 × 10⁻⁴ | 1.942 × 10⁻⁴ | CD300A/CD74/INHBB/LGALS9/TFF2/TNFRSF1A/TNFRSF1B | 7 |
| GO:0030217 | T cell differentiation | 9/81 | 324/18805 | 0.0277777777777778 | 6.44890260631001 | 6.50709476342271 | 1.051 × 10⁻⁵ | 2.725 × 10⁻⁴ | 1.942 × 10⁻⁴ | CD27/CD74/CD80/CTSL/IL2RA/IL4R/LGALS9/LILRB4/TNFRSF9 | 9 |
| GO:0045576 | mast cell activation | 5/81 | 69/18805 | 0.072463768115942 | 16.823224190374 | 8.66062835151269 | 1.185 × 10⁻⁵ | 2.998 × 10⁻⁴ | 2.137 × 10⁻⁴ | CD300A/HAVCR1/IL4R/LGALS9/NECTIN2 | 5 |
| GO:0061180 | mammary gland epithelium development | 5/81 | 69/18805 | 0.072463768115942 | 16.823224190374 | 8.66062835151269 | 1.185 × 10⁻⁵ | 2.998 × 10⁻⁴ | 2.137 × 10⁻⁴ | AREG/CCL11/CSF1/EPHA2/FGFR2 | 5 |
| GO:0019065 | receptor-mediated endocytosis of virus by host cell | 3/81 | 11/18805 | 0.272727272727273 | 63.3164983164983 | 13.597469310955 | 1.239 × 10⁻⁵ | 3.098 × 10⁻⁴ | 2.208 × 10⁻⁴ | ACE2/CTSL/SIGLEC1 | 3 |
| GO:0002718 | regulation of cytokine production involved in immune response | 6/81 | 126/18805 | 0.0476190476190476 | 11.0552616108172 | 7.44852277848478 | 1.728 × 10⁻⁵ | 4.269 × 10⁻⁴ | 3.043 × 10⁻⁴ | BST2/CD74/LILRB4/SPON2/TNFRSF14/TNFRSF1B | 6 |
| GO:1903706 | regulation of hemopoiesis | 10/81 | 436/18805 | 0.0229357798165138 | 5.32478196851286 | 6.00942638766717 | 1.75 × 10⁻⁵ | 4.274 × 10⁻⁴ | 3.047 × 10⁻⁴ | CD27/CD74/CD80/CSF1/FSTL3/IL2RA/IL4R/LGALS9/LILRB4/TNFRSF11A | 10 |
| GO:0002367 | cytokine production involved in immune response | 6/81 | 127/18805 | 0.047244094488189 | 10.968212306795 | 7.41348253211557 | 1.807 × 10⁻⁵ | 4.315 × 10⁻⁴ | 3.076 × 10⁻⁴ | BST2/CD74/LILRB4/SPON2/TNFRSF14/TNFRSF1B | 6 |
| GO:0022612 | gland morphogenesis | 6/81 | 127/18805 | 0.047244094488189 | 10.968212306795 | 7.41348253211557 | 1.807 × 10⁻⁵ | 4.315 × 10⁻⁴ | 3.076 × 10⁻⁴ | AREG/CCL11/CSF1/EGFR/EPHA2/FGFR2 | 6 |
| GO:0045580 | regulation of T cell differentiation | 7/81 | 192/18805 | 0.0364583333333333 | 8.46418467078189 | 6.83743350621096 | 1.905 × 10⁻⁵ | 4.498 × 10⁻⁴ | 3.206 × 10⁻⁴ | CD27/CD74/CD80/IL2RA/IL4R/LGALS9/LILRB4 | 7 |
| GO:0044406 | adhesion of symbiont to host | 3/81 | 13/18805 | 0.230769230769231 | 53.5754985754986 | 12.4720225827815 | 2.134 × 10⁻⁵ | 4.93 × 10⁻⁴ | 3.514 × 10⁻⁴ | ACE2/INHBB/NECTIN2 | 3 |
| GO:0075509 | endocytosis involved in viral entry into host cell | 3/81 | 13/18805 | 0.230769230769231 | 53.5754985754986 | 12.4720225827815 | 2.134 × 10⁻⁵ | 4.93 × 10⁻⁴ | 3.514 × 10⁻⁴ | ACE2/CTSL/SIGLEC1 | 3 |
| GO:0032102 | negative regulation of response to external stimulus | 10/81 | 448/18805 | 0.0223214285714286 | 5.18215388007055 | 5.89259341248123 | 2.208 × 10⁻⁵ | 5.046 × 10⁻⁴ | 3.597 × 10⁻⁴ | HAVCR2/IL2RA/LGALS9/MMP12/NBL1/NECTIN2/PLAUR/TNFRSF1A/TNFRSF1B/VSIG4 | 10 |
| GO:0070374 | positive regulation of ERK1 and ERK2 cascade | 7/81 | 198/18805 | 0.0353535353535354 | 8.20769422621275 | 6.70593106916071 | 2.322 × 10⁻⁵ | 5.25 × 10⁻⁴ | 3.742 × 10⁻⁴ | CD74/CHI3L1/EGFR/FGFR2/HAVCR2/LGALS9/TNFRSF11A | 7 |
| GO:0030879 | mammary gland development | 6/81 | 137/18805 | 0.0437956204379562 | 10.1676128683428 | 7.08330540772615 | 2.777 × 10⁻⁵ | 6.212 × 10⁻⁴ | 4.428 × 10⁻⁴ | AREG/CCL11/CSF1/EPHA2/FGFR2/TNFRSF11A | 6 |
| GO:0043903 | regulation of biological process involved in symbiotic interaction | 4/81 | 42/18805 | 0.0952380952380952 | 22.1105232216343 | 9.00825803550605 | 3.157 × 10⁻⁵ | 6.987 × 10⁻⁴ | 4.981 × 10⁻⁴ | BST2/CD74/LGALS9/NECTIN2 | 4 |
| GO:1901741 | positive regulation of myoblast fusion | 3/81 | 15/18805 | 0.2 | 46.4320987654321 | 11.5774617209509 | 3.375 × 10⁻⁵ | 7.393 × 10⁻⁴ | 5.27 × 10⁻⁴ | CXCL9/GDF15/IL4R | 3 |
| GO:0002449 | lymphocyte mediated immunity | 9/81 | 384/18805 | 0.0234375 | 5.44126157407407 | 5.78340595268102 | 4.011 × 10⁻⁵ | 8.697 × 10⁻⁴ | 6.2 × 10⁻⁴ | CD27/CD74/CD80/HAVCR2/IL4R/LGALS9/LILRB4/NECTIN2/TNFRSF1B | 9 |
| GO:0050671 | positive regulation of lymphocyte proliferation | 6/81 | 147/18805 | 0.0408163265306122 | 9.47593852355757 | 6.78550594861923 | 4.127 × 10⁻⁵ | 8.842 × 10⁻⁴ | 6.303 × 10⁻⁴ | CD74/CD80/HAVCR2/IL2RA/LGALS9/TNFRSF4 | 6 |
| GO:0030574 | collagen catabolic process | 4/81 | 45/18805 | 0.0888888888888889 | 20.636488340192 | 8.67405114130606 | 4.161 × 10⁻⁵ | 8.842 × 10⁻⁴ | 6.303 × 10⁻⁴ | CST3/CTSL/MMP12/MMP7 | 4 |
| GO:0002819 | regulation of adaptive immune response | 7/81 | 219/18805 | 0.0319634703196347 | 7.42065505383618 | 6.28603426179257 | 4.421 × 10⁻⁵ | 9.302 × 10⁻⁴ | 6.63 × 10⁻⁴ | CD80/HAVCR2/IL4R/LILRB4/NECTIN2/TNFRSF14/TNFRSF1B | 7 |
| GO:0002460 | adaptive immune response based on somatic recombination of immune receptors built from immunoglobulin superfamily domains | 9/81 | 396/18805 | 0.0227272727272727 | 5.27637485970819 | 5.65687492248984 | 5.09 × 10⁻⁵ | 0.001 | 7.559 × 10⁻⁴ | CD27/CD74/CD80/HAVCR2/IL18BP/IL4R/LILRB4/NECTIN2/TNFRSF1B | 9 |
| GO:0045619 | regulation of lymphocyte differentiation | 7/81 | 225/18805 | 0.0311111111111111 | 7.22277091906722 | 6.17618835436114 | 5.246 × 10⁻⁵ | 0.001 | 7.715 × 10⁻⁴ | CD27/CD74/CD80/IL2RA/IL4R/LGALS9/LILRB4 | 7 |
| GO:0031348 | negative regulation of defense response | 8/81 | 308/18805 | 0.025974025974026 | 6.03014269680936 | 5.85427783974143 | 5.393 × 10⁻⁵ | 0.001 | 7.854 × 10⁻⁴ | HAVCR2/IL2RA/LGALS9/MMP12/NECTIN2/TNFRSF1A/TNFRSF1B/VSIG4 | 8 |
| GO:0042088 | T-helper 1 type immune response | 4/81 | 49/18805 | 0.0816326530612245 | 18.9518770471151 | 8.27572608219616 | 5.84 × 10⁻⁵ | 0.001 | 8.425 × 10⁻⁴ | CD80/HAVCR2/IL18BP/IL4R | 4 |
| GO:0007173 | epidermal growth factor receptor signaling pathway | 6/81 | 158/18805 | 0.0379746835443038 | 8.81622128457572 | 6.48917100065622 | 6.174 × 10⁻⁵ | 0.001 | 8.822 × 10⁻⁴ | AREG/EGFR/EPHA2/FGFR2/PIGR/PLAUR | 6 |
| GO:0046636 | negative regulation of alpha-beta T cell activation | 4/81 | 50/18805 | 0.08 | 18.5728395061728 | 8.18345549729266 | 6.327 × 10⁻⁵ | 0.001 | 8.957 × 10⁻⁴ | CD300A/IL4R/LGALS9/TNFRSF14 | 4 |
| GO:0030595 | leukocyte chemotaxis | 7/81 | 234/18805 | 0.0299145299145299 | 6.94497203756463 | 6.01877924112335 | 6.718 × 10⁻⁵ | 0.001 | 9.422 × 10⁻⁴ | CCL11/CD74/CSF1/CXCL9/LGALS9/NBL1/TNFRSF11A | 7 |
| GO:0002685 | regulation of leukocyte migration | 7/81 | 236/18805 | 0.0296610169491525 | 6.88611634233103 | 5.98492758168766 | 7.087 × 10⁻⁵ | 0.001 | 9.603 × 10⁻⁴ | CD300A/CD74/CSF1/GDF15/LGALS9/NBL1/TNFRSF14 | 7 |
| GO:0002643 | regulation of tolerance induction | 3/81 | 19/18805 | 0.157894736842105 | 36.6569200779727 | 10.2275540554463 | 7.098 × 10⁻⁵ | 0.001 | 9.603 × 10⁻⁴ | HAVCR2/IL2RA/LILRB4 | 3 |
| GO:0033003 | regulation of mast cell activation | 3/81 | 19/18805 | 0.157894736842105 | 36.6569200779727 | 10.2275540554463 | 7.098 × 10⁻⁵ | 0.001 | 9.603 × 10⁻⁴ | CD300A/HAVCR1/NECTIN2 | 3 |
| GO:0045824 | negative regulation of innate immune response | 5/81 | 100/18805 | 0.05 | 11.608024691358 | 6.99558407009797 | 7.153 × 10⁻⁵ | 0.001 | 9.603 × 10⁻⁴ | HAVCR2/LGALS9/MMP12/NECTIN2/VSIG4 | 5 |
| GO:0001934 | positive regulation of protein phosphorylation | 9/81 | 414/18805 | 0.0217391304347826 | 5.04696725711218 | 5.47640430876867 | 7.164 × 10⁻⁵ | 0.001 | 9.603 × 10⁻⁴ | AREG/CD74/CD80/CHI3L1/EGFR/GFRA1/PLAUR/TNFRSF10A/TNFRSF11A | 9 |
| GO:0097529 | myeloid leukocyte migration | 7/81 | 237/18805 | 0.029535864978903 | 6.85706099911445 | 5.9681491880046 | 7.277 × 10⁻⁵ | 0.001 | 9.67 × 10⁻⁴ | CCL11/CD300A/CD74/CSF1/CXCL9/NBL1/TNFRSF11A | 7 |
| GO:0060326 | cell chemotaxis | 8/81 | 325/18805 | 0.0246153846153846 | 5.71471984805318 | 5.63916631400087 | 7.853 × 10⁻⁵ | 0.001 | 0.001 | CCL11/CD74/CSF1/CXCL9/EPHA2/LGALS9/NBL1/TNFRSF11A | 8 |
| GO:1901739 | regulation of myoblast fusion | 3/81 | 20/18805 | 0.15 | 34.8240740740741 | 9.95413735824335 | 8.325 × 10⁻⁵ | 0.002 | 0.001 | CXCL9/GDF15/IL4R | 3 |
| GO:0038145 | macrophage colony-stimulating factor signaling pathway | 4/81 | 54/18805 | 0.0740740740740741 | 17.1970736168267 | 7.83952110389448 | 8.576 × 10⁻⁵ | 0.002 | 0.001 | CSF1/EGFR/EPHA2/FGFR2 | 4 |
| GO:0008630 | intrinsic apoptotic signaling pathway in response to DNA damage | 5/81 | 106/18805 | 0.0471698113207547 | 10.9509666899604 | 6.75736423351558 | 9.437 × 10⁻⁵ | 0.002 | 0.001 | CD74/EPHA2/SHISA5/TNFRSF1A/TNFRSF1B | 5 |
| GO:0046596 | regulation of viral entry into host cell | 3/81 | 21/18805 | 0.142857142857143 | 33.1657848324515 | 9.70014161135863 | 9.682 × 10⁻⁵ | 0.002 | 0.001 | CD74/LGALS9/NECTIN2 | 3 |
| GO:0002448 | mast cell mediated immunity | 4/81 | 56/18805 | 0.0714285714285714 | 16.5828924162257 | 7.68106332817169 | 9.895 × 10⁻⁵ | 0.002 | 0.001 | CD300A/IL4R/LGALS9/SPON2 | 4 |
| GO:0046638 | positive regulation of alpha-beta T cell differentiation | 4/81 | 56/18805 | 0.0714285714285714 | 16.5828924162257 | 7.68106332817169 | 9.895 × 10⁻⁵ | 0.002 | 0.001 | CD80/IL4R/LGALS9/LILRB4 | 4 |
| GO:0030098 | lymphocyte differentiation | 9/81 | 432/18805 | 0.0208333333333333 | 4.83667695473251 | 5.3060994293678 | 9.908 × 10⁻⁵ | 0.002 | 0.001 | CD27/CD74/CD80/CTSL/IL2RA/IL4R/LGALS9/LILRB4/TNFRSF9 | 9 |
| GO:0042327 | positive regulation of phosphorylation | 9/81 | 437/18805 | 0.0205949656750572 | 4.7813374014747 | 5.26045764204411 | 1.081 × 10⁻⁴ | 0.002 | 0.001 | AREG/CD74/CD80/CHI3L1/EGFR/GFRA1/PLAUR/TNFRSF10A/TNFRSF11A | 9 |
| GO:1903054 | negative regulation of extracellular matrix organization | 3/81 | 22/18805 | 0.136363636363636 | 31.6582491582492 | 9.46334177072304 | 1.118 × 10⁻⁴ | 0.002 | 0.001 | CST3/TNFRSF1A/TNFRSF1B | 3 |
| GO:0038127 | ERBB signaling pathway | 6/81 | 180/18805 | 0.0333333333333333 | 7.73868312757202 | 5.97491128607388 | 1.267 × 10⁻⁴ | 0.002 | 0.002 | AREG/EGFR/EPHA2/FGFR2/PIGR/PLAUR | 6 |
| GO:0030316 | osteoclast differentiation | 5/81 | 113/18805 | 0.0442477876106195 | 10.2725882224407 | 6.50250394089282 | 1.277 × 10⁻⁴ | 0.002 | 0.002 | CSF1/EPHA2/FSTL3/LILRB4/TNFRSF11A | 5 |
| GO:0060143 | positive regulation of syncytium formation by plasma membrane fusion | 3/81 | 23/18805 | 0.130434782608696 | 30.2818035426731 | 9.24185471349511 | 1.281 × 10⁻⁴ | 0.002 | 0.002 | CXCL9/GDF15/IL4R | 3 |
| GO:0036006 | cellular response to macrophage colony-stimulating factor stimulus | 4/81 | 60/18805 | 0.0666666666666667 | 15.477366255144 | 7.38738577498155 | 1.297 × 10⁻⁴ | 0.002 | 0.002 | CSF1/EGFR/EPHA2/FGFR2 | 4 |
| GO:0002823 | negative regulation of adaptive immune response based on somatic recombination of immune receptors built from immunoglobulin superfamily domains | 4/81 | 62/18805 | 0.0645161290322581 | 14.9780963759458 | 7.25091204600409 | 1.474 × 10⁻⁴ | 0.002 | 0.002 | CD80/HAVCR2/IL4R/LILRB4 | 4 |
| GO:0038065 | collagen-activated signaling pathway | 4/81 | 62/18805 | 0.0645161290322581 | 14.9780963759458 | 7.25091204600409 | 1.474 × 10⁻⁴ | 0.002 | 0.002 | EGFR/EPHA2/FGFR2/ITGA11 | 4 |
| GO:0043030 | regulation of macrophage activation | 4/81 | 63/18805 | 0.0634920634920635 | 14.7403488144229 | 7.18502660091587 | 1.569 × 10⁻⁴ | 0.003 | 0.002 | CD74/HAVCR2/IL4R/VSIG4 | 4 |
| GO:0036005 | response to macrophage colony-stimulating factor | 4/81 | 64/18805 | 0.0625 | 14.5100308641975 | 7.12062741988144 | 1.668 × 10⁻⁴ | 0.003 | 0.002 | CSF1/EGFR/EPHA2/FGFR2 | 4 |
| GO:0061138 | morphogenesis of a branching epithelium | 6/81 | 191/18805 | 0.031413612565445 | 7.29299980608881 | 5.74940446177291 | 1.751 × 10⁻⁴ | 0.003 | 0.002 | ADM/AREG/CCL11/CSF1/EPHA2/FGFR2 | 6 |
| GO:0006935 | chemotaxis | 9/81 | 466/18805 | 0.01931330472103 | 4.48378636146877 | 5.00869933251591 | 1.752 × 10⁻⁴ | 0.003 | 0.002 | CCL11/CD74/CSF1/CXCL9/EPHA2/LGALS9/NBL1/PLAUR/TNFRSF11A | 9 |
| GO:0042330 | taxis | 9/81 | 468/18805 | 0.0192307692307692 | 4.46462488129155 | 4.99210048362768 | 1.809 × 10⁻⁴ | 0.003 | 0.002 | CCL11/CD74/CSF1/CXCL9/EPHA2/LGALS9/NBL1/PLAUR/TNFRSF11A | 9 |
| GO:0045953 | negative regulation of natural killer cell mediated cytotoxicity | 3/81 | 26/18805 | 0.115384615384615 | 26.7877492877493 | 8.65430499961478 | 1.864 × 10⁻⁴ | 0.003 | 0.002 | HAVCR2/LGALS9/NECTIN2 | 3 |
| GO:0071887 | leukocyte apoptotic process | 5/81 | 124/18805 | 0.0403225806451613 | 9.36131023496615 | 6.14403151187024 | 1.974 × 10⁻⁴ | 0.003 | 0.002 | CD27/CD74/CTSL/IL2RA/LGALS9 | 5 |
| GO:0032623 | interleukin-2 production | 4/81 | 67/18805 | 0.0597014925373134 | 13.8603279896812 | 6.93579332612799 | 1.992 × 10⁻⁴ | 0.003 | 0.002 | CD80/HAVCR2/LILRB4/VSIG4 | 4 |
| GO:0032663 | regulation of interleukin-2 production | 4/81 | 67/18805 | 0.0597014925373134 | 13.8603279896812 | 6.93579332612799 | 1.992 × 10⁻⁴ | 0.003 | 0.002 | CD80/HAVCR2/LILRB4/VSIG4 | 4 |
| GO:0006898 | receptor-mediated endocytosis | 7/81 | 280/18805 | 0.025 | 5.80401234567901 | 5.32687292515936 | 2.045 × 10⁻⁴ | 0.003 | 0.002 | ACE2/ADM/ASGR1/CTSL/LILRB4/SCARB2/SIGLEC1 | 7 |
| GO:0002716 | negative regulation of natural killer cell mediated immunity | 3/81 | 27/18805 | 0.111111111111111 | 25.7956104252401 | 8.48008750141512 | 2.09 × 10⁻⁴ | 0.003 | 0.002 | HAVCR2/LGALS9/NECTIN2 | 3 |
| GO:0043043 | peptide biosynthetic process | 3/81 | 27/18805 | 0.111111111111111 | 25.7956104252401 | 8.48008750141512 | 2.09 × 10⁻⁴ | 0.003 | 0.002 | GGT1/LGALS4/MMP7 | 3 |
| GO:0018108 | peptidyl-tyrosine phosphorylation | 6/81 | 198/18805 | 0.0303030303030303 | 7.03516647961093 | 5.61502876412194 | 2.128 × 10⁻⁴ | 0.003 | 0.002 | AREG/CD80/EGFR/FGFR2/GFRA1/LILRB4 | 6 |
| GO:0018212 | peptidyl-tyrosine modification | 6/81 | 199/18805 | 0.0301507537688442 | 6.999813884236 | 5.59636617530502 | 2.186 × 10⁻⁴ | 0.003 | 0.002 | AREG/CD80/EGFR/FGFR2/GFRA1/LILRB4 | 6 |
| GO:0002706 | regulation of lymphocyte mediated immunity | 6/81 | 200/18805 | 0.03 | 6.96481481481481 | 5.57783213623873 | 2.246 × 10⁻⁴ | 0.003 | 0.002 | CD80/HAVCR2/LGALS9/LILRB4/NECTIN2/TNFRSF1B | 6 |
| GO:0043304 | regulation of mast cell degranulation | 3/81 | 28/18805 | 0.107142857142857 | 24.8743386243386 | 8.31506362021477 | 2.334 × 10⁻⁴ | 0.003 | 0.002 | CD300A/IL4R/LGALS9 | 3 |
| GO:0052372 | modulation by symbiont of entry into host | 3/81 | 28/18805 | 0.107142857142857 | 24.8743386243386 | 8.31506362021477 | 2.334 × 10⁻⁴ | 0.003 | 0.002 | CD74/LGALS9/NECTIN2 | 3 |
| GO:0002761 | regulation of myeloid leukocyte differentiation | 5/81 | 130/18805 | 0.0384615384615385 | 8.9292497625831 | 5.9668041517391 | 2.459 × 10⁻⁴ | 0.004 | 0.003 | CD74/CSF1/FSTL3/LILRB4/TNFRSF11A | 5 |
| GO:0002822 | regulation of adaptive immune response based on somatic recombination of immune receptors built from immunoglobulin superfamily domains | 6/81 | 204/18805 | 0.0294117647058824 | 6.8282498184459 | 5.50495040414723 | 2.499 × 10⁻⁴ | 0.004 | 0.003 | CD80/HAVCR2/IL4R/LILRB4/NECTIN2/TNFRSF1B | 6 |
| GO:0060142 | regulation of syncytium formation by plasma membrane fusion | 3/81 | 29/18805 | 0.103448275862069 | 24.0166028097063 | 8.15843764222228 | 2.595 × 10⁻⁴ | 0.004 | 0.003 | CXCL9/GDF15/IL4R | 3 |
| GO:0010562 | positive regulation of phosphorus metabolic process | 9/81 | 493/18805 | 0.0182555780933063 | 4.23822402524228 | 4.79215409763952 | 2.661 × 10⁻⁴ | 0.004 | 0.003 | AREG/CD74/CD80/CHI3L1/EGFR/GFRA1/PLAUR/TNFRSF10A/TNFRSF11A | 9 |
| GO:0045937 | positive regulation of phosphate metabolic process | 9/81 | 493/18805 | 0.0182555780933063 | 4.23822402524228 | 4.79215409763952 | 2.661 × 10⁻⁴ | 0.004 | 0.003 | AREG/CD74/CD80/CHI3L1/EGFR/GFRA1/PLAUR/TNFRSF10A/TNFRSF11A | 9 |
| GO:0001818 | negative regulation of cytokine production | 8/81 | 389/18805 | 0.0205655526992288 | 4.77450887048145 | 4.94772909303043 | 2.69 × 10⁻⁴ | 0.004 | 0.003 | BST2/EPHA2/HAVCR2/INHBB/LGALS9/LILRB4/SIGLEC1/VSIG4 | 8 |
| GO:0038061 | non-canonical NF-kappaB signal transduction | 5/81 | 133/18805 | 0.037593984962406 | 8.72783811380303 | 5.88242950519258 | 2.734 × 10⁻⁴ | 0.004 | 0.003 | CD27/CHI3L1/HAVCR2/LGALS9/TNFRSF10A | 5 |
| GO:0032722 | positive regulation of chemokine production | 4/81 | 73/18805 | 0.0547945205479452 | 12.7211229494335 | 6.59943706344805 | 2.774 × 10⁻⁴ | 0.004 | 0.003 | CD74/HAVCR2/IL4R/LGALS9 | 4 |
| GO:0045670 | regulation of osteoclast differentiation | 4/81 | 73/18805 | 0.0547945205479452 | 12.7211229494335 | 6.59943706344805 | 2.774 × 10⁻⁴ | 0.004 | 0.003 | CSF1/FSTL3/LILRB4/TNFRSF11A | 4 |
| GO:0001763 | morphogenesis of a branching structure | 6/81 | 208/18805 | 0.0288461538461538 | 6.69693732193732 | 5.4340043002282 | 2.774 × 10⁻⁴ | 0.004 | 0.003 | ADM/AREG/CCL11/CSF1/EPHA2/FGFR2 | 6 |
| GO:0035710 | CD4-positive, alpha-beta T cell activation | 5/81 | 134/18805 | 0.0373134328358209 | 8.66270499355076 | 5.85489391055471 | 2.83 × 10⁻⁴ | 0.004 | 0.003 | CD80/CTSL/IL2RA/IL4R/LGALS9 | 5 |
| GO:0032703 | negative regulation of interleukin-2 production | 3/81 | 30/18805 | 0.1 | 23.2160493827161 | 8.00950729185536 | 2.874 × 10⁻⁴ | 0.004 | 0.003 | HAVCR2/LILRB4/VSIG4 | 3 |
| GO:0002832 | negative regulation of response to biotic stimulus | 5/81 | 135/18805 | 0.037037037037037 | 8.59853680841335 | 5.82764396882531 | 2.929 × 10⁻⁴ | 0.004 | 0.003 | HAVCR2/LGALS9/MMP12/NECTIN2/VSIG4 | 5 |
| GO:0046635 | positive regulation of alpha-beta T cell activation | 4/81 | 75/18805 | 0.0533333333333333 | 12.3818930041152 | 6.4959781609912 | 3.078 × 10⁻⁴ | 0.004 | 0.003 | CD80/IL4R/LGALS9/LILRB4 | 4 |
| GO:0043122 | regulation of canonical NF-kappaB signal transduction | 7/81 | 300/18805 | 0.0233333333333333 | 5.41707818930041 | 5.07246996458856 | 3.11 × 10⁻⁴ | 0.004 | 0.003 | BST2/CD74/EDA2R/LGALS9/LILRB4/SHISA5/TNFRSF1A | 7 |
| GO:0002825 | regulation of T-helper 1 type immune response | 3/81 | 31/18805 | 0.0967741935483871 | 22.4671445639188 | 7.86765007025416 | 3.172 × 10⁻⁴ | 0.004 | 0.003 | CD80/HAVCR2/IL4R | 3 |
| GO:0019886 | antigen processing and presentation of exogenous peptide antigen via MHC class II | 3/81 | 31/18805 | 0.0967741935483871 | 22.4671445639188 | 7.86765007025416 | 3.172 × 10⁻⁴ | 0.004 | 0.003 | CD74/CTSL/IFI30 | 3 |
| GO:0043123 | positive regulation of canonical NF-kappaB signal transduction | 6/81 | 214/18805 | 0.0280373831775701 | 6.509172724126 | 5.33101880318466 | 3.23 × 10⁻⁴ | 0.004 | 0.003 | BST2/CD74/EDA2R/LGALS9/SHISA5/TNFRSF1A | 6 |
| GO:0046632 | alpha-beta T cell differentiation | 5/81 | 138/18805 | 0.036231884057971 | 8.41161209518698 | 5.74755684568109 | 3.242 × 10⁻⁴ | 0.004 | 0.003 | CD80/CTSL/IL4R/LGALS9/LILRB4 | 5 |
| GO:0002286 | T cell activation involved in immune response | 5/81 | 140/18805 | 0.0357142857142857 | 8.29144620811287 | 5.69550203485035 | 3.464 × 10⁻⁴ | 0.004 | 0.003 | CD74/CD80/HAVCR2/IL4R/LGALS9 | 5 |
| GO:0001911 | negative regulation of leukocyte mediated cytotoxicity | 3/81 | 32/18805 | 0.09375 | 21.7650462962963 | 7.73231195928549 | 3.49 × 10⁻⁴ | 0.004 | 0.003 | HAVCR2/LGALS9/NECTIN2 | 3 |
| GO:0032682 | negative regulation of chemokine production | 3/81 | 32/18805 | 0.09375 | 21.7650462962963 | 7.73231195928549 | 3.49 × 10⁻⁴ | 0.004 | 0.003 | EPHA2/LGALS9/LILRB4 | 3 |
| GO:0050679 | positive regulation of epithelial cell proliferation | 6/81 | 218/18805 | 0.0275229357798165 | 6.38973836221543 | 5.26452988340698 | 3.566 × 10⁻⁴ | 0.004 | 0.003 | AREG/CCL11/EGFR/FGFR2/MMP12/SPINK1 | 6 |
| GO:0002702 | positive regulation of production of molecular mediator of immune response | 5/81 | 141/18805 | 0.0354609929078014 | 8.23264162507661 | 5.66986155295205 | 3.58 × 10⁻⁴ | 0.004 | 0.003 | CD74/IL4R/SPON2/TNFRSF14/TNFRSF4 | 5 |
| GO:0046637 | regulation of alpha-beta T cell differentiation | 4/81 | 81/18805 | 0.0493827160493827 | 11.4647157445511 | 6.20781754014688 | 4.131 × 10⁻⁴ | 0.005 | 0.004 | CD80/IL4R/LGALS9/LILRB4 | 4 |
| GO:0007249 | canonical NF-kappaB signal transduction | 7/81 | 318/18805 | 0.0220125786163522 | 5.11045112198152 | 4.86225959660832 | 4.413 × 10⁻⁴ | 0.005 | 0.004 | BST2/CD74/EDA2R/LGALS9/LILRB4/SHISA5/TNFRSF1A | 7 |
| GO:0002495 | antigen processing and presentation of peptide antigen via MHC class II | 3/81 | 35/18805 | 0.0857142857142857 | 19.8994708994709 | 7.36071235255702 | 4.562 × 10⁻⁴ | 0.006 | 0.004 | CD74/CTSL/IFI30 | 3 |
| GO:0016486 | peptide hormone processing | 3/81 | 35/18805 | 0.0857142857142857 | 19.8994708994709 | 7.36071235255702 | 4.562 × 10⁻⁴ | 0.006 | 0.004 | ACE2/CTSL/REN | 3 |
| GO:0031342 | negative regulation of cell killing | 3/81 | 35/18805 | 0.0857142857142857 | 19.8994708994709 | 7.36071235255702 | 4.562 × 10⁻⁴ | 0.006 | 0.004 | HAVCR2/LGALS9/NECTIN2 | 3 |
| GO:0097530 | granulocyte migration | 5/81 | 149/18805 | 0.0335570469798658 | 7.79062059822686 | 5.47345621919533 | 4.611 × 10⁻⁴ | 0.006 | 0.004 | CCL11/CD300A/CD74/CSF1/CXCL9 | 5 |
| GO:0009615 | response to virus | 8/81 | 425/18805 | 0.0188235294117647 | 4.37007988380537 | 4.62200298271641 | 4.852 × 10⁻⁴ | 0.006 | 0.004 | BST2/CCL11/CXCL9/LGALS9/MMP12/NPC2/RNASE6/SPON2 | 8 |
| GO:0032868 | response to insulin | 7/81 | 324/18805 | 0.0216049382716049 | 5.01581313824112 | 4.79569530431174 | 4.934 × 10⁻⁴ | 0.006 | 0.004 | ADM/BAIAP2/EGFR/EPHA2/FGFR2/INHBB/TNFRSF11A | 7 |
| GO:0048013 | ephrin receptor signaling pathway | 4/81 | 85/18805 | 0.0470588235294118 | 10.9251997095134 | 6.03203856244657 | 4.963 × 10⁻⁴ | 0.006 | 0.004 | EFNA4/EGFR/EPHA2/FGFR2 | 4 |
| GO:0070227 | lymphocyte apoptotic process | 4/81 | 85/18805 | 0.0470588235294118 | 10.9251997095134 | 6.03203856244657 | 4.963 × 10⁻⁴ | 0.006 | 0.004 | CD27/CD74/IL2RA/LGALS9 | 4 |
| GO:2000514 | regulation of CD4-positive, alpha-beta T cell activation | 4/81 | 85/18805 | 0.0470588235294118 | 10.9251997095134 | 6.03203856244657 | 4.963 × 10⁻⁴ | 0.006 | 0.004 | CD80/IL2RA/IL4R/LGALS9 | 4 |
| GO:1903531 | negative regulation of secretion by cell | 5/81 | 154/18805 | 0.0324675324675325 | 7.5376783710117 | 5.35798094761559 | 5.36 × 10⁻⁴ | 0.006 | 0.004 | CD300A/INHBB/LGALS9/TNFRSF1A/TNFRSF1B | 5 |
| GO:0002504 | antigen processing and presentation of peptide or polysaccharide antigen via MHC class II | 3/81 | 37/18805 | 0.0810810810810811 | 18.8238238238238 | 7.13774551692603 | 5.383 × 10⁻⁴ | 0.006 | 0.004 | CD74/CTSL/IFI30 | 3 |
| GO:0097193 | intrinsic apoptotic signaling pathway | 7/81 | 330/18805 | 0.0212121212121212 | 4.92461653572765 | 4.73075318273672 | 5.502 × 10⁻⁴ | 0.006 | 0.005 | CD74/EDA2R/EPHA2/PLAUR/SHISA5/TNFRSF1A/TNFRSF1B | 7 |
| GO:0050730 | regulation of peptidyl-tyrosine phosphorylation | 5/81 | 156/18805 | 0.0320512820512821 | 7.44104146881925 | 5.31323389398338 | 5.683 × 10⁻⁴ | 0.006 | 0.005 | AREG/CD80/EGFR/GFRA1/LILRB4 | 5 |
| GO:0043372 | positive regulation of CD4-positive, alpha-beta T cell differentiation | 3/81 | 38/18805 | 0.0789473684210526 | 18.3284600389864 | 7.03270912026183 | 5.826 × 10⁻⁴ | 0.007 | 0.005 | CD80/IL4R/LGALS9 | 3 |
| GO:0048145 | regulation of fibroblast proliferation | 4/81 | 90/18805 | 0.0444444444444444 | 10.318244170096 | 5.82812341529 | 6.162 × 10⁻⁴ | 0.007 | 0.005 | CD300A/CD74/EGFR/IFI30 | 4 |
| GO:0048754 | branching morphogenesis of an epithelial tube | 5/81 | 159/18805 | 0.0314465408805031 | 7.3006444599736 | 5.24757929854692 | 6.197 × 10⁻⁴ | 0.007 | 0.005 | AREG/CCL11/CSF1/EPHA2/FGFR2 | 5 |
| GO:0006516 | glycoprotein catabolic process | 3/81 | 39/18805 | 0.0769230769230769 | 17.8584995251662 | 6.93160298428025 | 6.292 × 10⁻⁴ | 0.007 | 0.005 | CST3/CTSL/MMP12 | 3 |
| GO:0140448 | signaling receptor ligand precursor processing | 3/81 | 39/18805 | 0.0769230769230769 | 17.8584995251662 | 6.93160298428025 | 6.292 × 10⁻⁴ | 0.007 | 0.005 | ACE2/CTSL/REN | 3 |
| GO:0048732 | gland development | 8/81 | 451/18805 | 0.0177383592017738 | 4.11814623196737 | 4.40846807611699 | 7.16 × 10⁻⁴ | 0.008 | 0.006 | AREG/CCL11/CSF1/EGFR/EPHA2/FGFR2/INHBB/TNFRSF11A | 8 |
| GO:0002478 | antigen processing and presentation of exogenous peptide antigen | 3/81 | 41/18805 | 0.0731707317073171 | 16.9873532068654 | 6.7402204421842 | 7.294 × 10⁻⁴ | 0.008 | 0.006 | CD74/CTSL/IFI30 | 3 |
| GO:0002573 | myeloid leukocyte differentiation | 6/81 | 250/18805 | 0.024 | 5.57185185185185 | 4.78630079493748 | 7.34 × 10⁻⁴ | 0.008 | 0.006 | CD74/CSF1/EPHA2/FSTL3/LILRB4/TNFRSF11A | 6 |
| GO:0001503 | ossification | 8/81 | 458/18805 | 0.0174672489082969 | 4.05520513235215 | 4.35370399055337 | 7.913 × 10⁻⁴ | 0.009 | 0.006 | AREG/CSF1/EGFR/EPHA2/FGFR2/FSTL3/ITGA11/TNFRSF11A | 8 |
| GO:0002002 | regulation of angiotensin levels in blood | 2/81 | 10/18805 | 0.2 | 46.4320987654321 | 9.45170045065842 | 8.064 × 10⁻⁴ | 0.009 | 0.006 | ACE2/REN | 2 |
| GO:0002420 | natural killer cell mediated cytotoxicity directed against tumor cell target | 2/81 | 10/18805 | 0.2 | 46.4320987654321 | 9.45170045065842 | 8.064 × 10⁻⁴ | 0.009 | 0.006 | HAVCR2/NECTIN2 | 2 |
| GO:0002778 | antibacterial peptide production | 2/81 | 10/18805 | 0.2 | 46.4320987654321 | 9.45170045065842 | 8.064 × 10⁻⁴ | 0.009 | 0.006 | LGALS4/MMP7 | 2 |
| GO:0002855 | regulation of natural killer cell mediated immune response to tumor cell | 2/81 | 10/18805 | 0.2 | 46.4320987654321 | 9.45170045065842 | 8.064 × 10⁻⁴ | 0.009 | 0.006 | HAVCR2/NECTIN2 | 2 |
| GO:0007250 | activation of NF-kappaB-inducing kinase activity | 2/81 | 10/18805 | 0.2 | 46.4320987654321 | 9.45170045065842 | 8.064 × 10⁻⁴ | 0.009 | 0.006 | CHI3L1/TNFRSF10A | 2 |
| GO:0045657 | positive regulation of monocyte differentiation | 2/81 | 10/18805 | 0.2 | 46.4320987654321 | 9.45170045065842 | 8.064 × 10⁻⁴ | 0.009 | 0.006 | CD74/CSF1 | 2 |
| GO:0046640 | regulation of alpha-beta T cell proliferation | 3/81 | 43/18805 | 0.0697674418604651 | 16.1972437553833 | 6.56187277514385 | 8.392 × 10⁻⁴ | 0.009 | 0.006 | IL2RA/LGALS9/TNFRSF14 | 3 |
| GO:0002275 | myeloid cell activation involved in immune response | 4/81 | 98/18805 | 0.0408163265306122 | 9.47593852355757 | 5.53308162796159 | 8.487 × 10⁻⁴ | 0.009 | 0.006 | CD300A/HAVCR2/IL4R/LGALS9 | 4 |
| GO:0043409 | negative regulation of MAPK cascade | 5/81 | 171/18805 | 0.0292397660818713 | 6.78831853295791 | 5.00110239452367 | 8.603 × 10⁻⁴ | 0.009 | 0.006 | ACE2/AMBP/CD300A/LILRB4/RNF149 | 5 |
| GO:0051051 | negative regulation of transport | 8/81 | 467/18805 | 0.0171306209850107 | 3.977053427446 | 4.2848675239497 | 8.973 × 10⁻⁴ | 0.009 | 0.007 | CD300A/CD74/INHBB/LGALS9/SPINK1/TFF2/TNFRSF1A/TNFRSF1B | 8 |
| GO:0046006 | regulation of activated T cell proliferation | 3/81 | 44/18805 | 0.0681818181818182 | 15.8291245791246 | 6.47712345240363 | 8.978 × 10⁻⁴ | 0.009 | 0.007 | IL2RA/LGALS9/LILRB4 | 3 |
| GO:0032689 | negative regulation of type II interferon production | 3/81 | 45/18805 | 0.0666666666666667 | 15.477366255144 | 6.39510553582565 | 9.589 × 10⁻⁴ | 0.010 | 0.007 | HAVCR2/LGALS9/LILRB4 | 3 |
| GO:0002423 | natural killer cell mediated immune response to tumor cell | 2/81 | 11/18805 | 0.181818181818182 | 42.2109988776656 | 8.99224615713845 | 9.828 × 10⁻⁴ | 0.010 | 0.007 | HAVCR2/NECTIN2 | 2 |
| GO:0002887 | negative regulation of myeloid leukocyte mediated immunity | 2/81 | 11/18805 | 0.181818181818182 | 42.2109988776656 | 8.99224615713845 | 9.828 × 10⁻⁴ | 0.010 | 0.007 | CD300A/LGALS9 | 2 |
| GO:0042756 | drinking behavior | 2/81 | 11/18805 | 0.181818181818182 | 42.2109988776656 | 8.99224615713845 | 9.828 × 10⁻⁴ | 0.010 | 0.007 | ACE2/REN | 2 |
| GO:0045625 | regulation of T-helper 1 cell differentiation | 2/81 | 11/18805 | 0.181818181818182 | 42.2109988776656 | 8.99224615713845 | 9.828 × 10⁻⁴ | 0.010 | 0.007 | CD80/IL4R | 2 |
| GO:0048009 | insulin-like growth factor receptor signaling pathway | 4/81 | 102/18805 | 0.0392156862745098 | 9.1043330912612 | 5.39796477025899 | 9.855 × 10⁻⁴ | 0.010 | 0.007 | EGFR/EPHA2/FGFR2/IGFBP4 | 4 |
| GO:0032635 | interleukin-6 production | 5/81 | 177/18805 | 0.0282485875706215 | 6.55820604031527 | 4.88659644685783 | 0.001 | 0.010 | 0.007 | CD74/HAVCR2/LGALS9/LILRB4/SPON2 | 5 |
| GO:0032675 | regulation of interleukin-6 production | 5/81 | 177/18805 | 0.0282485875706215 | 6.55820604031527 | 4.88659644685783 | 0.001 | 0.010 | 0.007 | CD74/HAVCR2/LGALS9/LILRB4/SPON2 | 5 |
| GO:0031638 | zymogen activation | 3/81 | 46/18805 | 0.0652173913043478 | 15.1409017713366 | 6.31567093777215 | 0.001 | 0.010 | 0.007 | CTSL/GGT1/PLAUR | 3 |
| GO:0043300 | regulation of leukocyte degranulation | 3/81 | 46/18805 | 0.0652173913043478 | 15.1409017713366 | 6.31567093777215 | 0.001 | 0.010 | 0.007 | CD300A/IL4R/LGALS9 | 3 |
| GO:0046633 | alpha-beta T cell proliferation | 3/81 | 46/18805 | 0.0652173913043478 | 15.1409017713366 | 6.31567093777215 | 0.001 | 0.010 | 0.007 | IL2RA/LGALS9/TNFRSF14 | 3 |
| GO:0071375 | cellular response to peptide hormone stimulus | 7/81 | 369/18805 | 0.018970189701897 | 4.40412860918733 | 4.34364283556748 | 0.001 | 0.010 | 0.007 | BAIAP2/EGFR/EPHA2/FGFR2/GDF15/INHBB/SPINK1 | 7 |
| GO:0007520 | myoblast fusion | 3/81 | 47/18805 | 0.0638297872340426 | 14.8187549251379 | 6.23868262030735 | 0.001 | 0.011 | 0.007 | CXCL9/GDF15/IL4R | 3 |
| GO:0050798 | activated T cell proliferation | 3/81 | 47/18805 | 0.0638297872340426 | 14.8187549251379 | 6.23868262030735 | 0.001 | 0.011 | 0.007 | IL2RA/LGALS9/LILRB4 | 3 |
| GO:0042102 | positive regulation of T cell proliferation | 4/81 | 105/18805 | 0.0380952380952381 | 8.84420928865373 | 5.30140926994325 | 0.001 | 0.011 | 0.008 | CD80/HAVCR2/IL2RA/LGALS9 | 4 |
| GO:0002709 | regulation of T cell mediated immunity | 4/81 | 106/18805 | 0.0377358490566038 | 8.76077335196832 | 5.2700781649607 | 0.001 | 0.011 | 0.008 | CD80/LILRB4/NECTIN2/TNFRSF1B | 4 |
| GO:0031295 | T cell costimulation | 3/81 | 48/18805 | 0.0625 | 14.5100308641975 | 6.16401355574965 | 0.001 | 0.011 | 0.008 | CD80/LILRB4/TNFRSF14 | 3 |
| GO:2000516 | positive regulation of CD4-positive, alpha-beta T cell activation | 3/81 | 48/18805 | 0.0625 | 14.5100308641975 | 6.16401355574965 | 0.001 | 0.011 | 0.008 | CD80/IL4R/LGALS9 | 3 |
| GO:0002775 | antimicrobial peptide production | 2/81 | 12/18805 | 0.166666666666667 | 38.6934156378601 | 8.59065689599698 | 0.001 | 0.011 | 0.008 | LGALS4/MMP7 | 2 |
| GO:0042117 | monocyte activation | 2/81 | 12/18805 | 0.166666666666667 | 38.6934156378601 | 8.59065689599698 | 0.001 | 0.011 | 0.008 | CSF1/LILRB4 | 2 |
| GO:0097067 | cellular response to thyroid hormone stimulus | 2/81 | 12/18805 | 0.166666666666667 | 38.6934156378601 | 8.59065689599698 | 0.001 | 0.011 | 0.008 | CTSL/INHBB | 2 |
| GO:0043367 | CD4-positive, alpha-beta T cell differentiation | 4/81 | 107/18805 | 0.0373831775700935 | 8.67889696550133 | 5.23915763832003 | 0.001 | 0.011 | 0.008 | CD80/CTSL/IL4R/LGALS9 | 4 |
| GO:0050673 | epithelial cell proliferation | 8/81 | 488/18805 | 0.0163934426229508 | 3.80590973487148 | 4.13071040466029 | 0.001 | 0.011 | 0.008 | AREG/CCL11/EGFR/EPHA2/FGFR2/IGFBP4/MMP12/SPINK1 | 8 |
| GO:0050829 | defense response to Gram-negative bacterium | 4/81 | 108/18805 | 0.037037037037037 | 8.59853680841335 | 5.20863829680044 | 0.001 | 0.011 | 0.008 | ADM/MMP7/RNASE6/TNFRSF14 | 4 |
| GO:0097305 | response to alcohol | 6/81 | 276/18805 | 0.0217391304347826 | 5.04696725711218 | 4.45478301870483 | 0.001 | 0.011 | 0.008 | CD27/FGFR2/INHBB/NEFL/SPINK1/TNFRSF11A | 6 |
| GO:0031294 | lymphocyte costimulation | 3/81 | 49/18805 | 0.0612244897959184 | 14.2139077853364 | 6.09154580436569 | 0.001 | 0.011 | 0.008 | CD80/LILRB4/TNFRSF14 | 3 |
| GO:0036323 | vascular endothelial growth factor receptor-1 signaling pathway | 3/81 | 49/18805 | 0.0612244897959184 | 14.2139077853364 | 6.09154580436569 | 0.001 | 0.011 | 0.008 | EGFR/EPHA2/FGFR2 | 3 |
| GO:0019884 | antigen processing and presentation of exogenous antigen | 3/81 | 50/18805 | 0.06 | 13.9296296296296 | 6.02116969391218 | 0.001 | 0.012 | 0.008 | CD74/CTSL/IFI30 | 3 |
| GO:0048144 | fibroblast proliferation | 4/81 | 110/18805 | 0.0363636363636364 | 8.44219977553311 | 5.14876708625179 | 0.001 | 0.012 | 0.008 | CD300A/CD74/EGFR/IFI30 | 4 |
| GO:0007565 | female pregnancy | 5/81 | 190/18805 | 0.0263157894736842 | 6.10948667966212 | 4.65576301464357 | 0.001 | 0.012 | 0.009 | ACE2/ADM/AMBP/HAVCR2/LGALS9 | 5 |
| GO:0002664 | regulation of T cell tolerance induction | 2/81 | 13/18805 | 0.153846153846154 | 35.7169990503324 | 8.23560799029559 | 0.001 | 0.012 | 0.009 | IL2RA/LILRB4 | 2 |
| GO:0043301 | negative regulation of leukocyte degranulation | 2/81 | 13/18805 | 0.153846153846154 | 35.7169990503324 | 8.23560799029559 | 0.001 | 0.012 | 0.009 | CD300A/LGALS9 | 2 |
| GO:0060670 | branching involved in labyrinthine layer morphogenesis | 2/81 | 13/18805 | 0.153846153846154 | 35.7169990503324 | 8.23560799029559 | 0.001 | 0.012 | 0.009 | ADM/FGFR2 | 2 |
| GO:0070278 | extracellular matrix constituent secretion | 2/81 | 13/18805 | 0.153846153846154 | 35.7169990503324 | 8.23560799029559 | 0.001 | 0.012 | 0.009 | TNFRSF1A/TNFRSF1B | 2 |
| GO:1902337 | regulation of apoptotic process involved in morphogenesis | 2/81 | 13/18805 | 0.153846153846154 | 35.7169990503324 | 8.23560799029559 | 0.001 | 0.012 | 0.009 | TNFRSF1A/TNFRSF1B | 2 |
| GO:0032963 | collagen metabolic process | 4/81 | 112/18805 | 0.0357142857142857 | 8.29144620811287 | 5.09039517993174 | 0.001 | 0.012 | 0.009 | CST3/CTSL/MMP12/MMP7 | 4 |
| GO:0042116 | macrophage activation | 4/81 | 113/18805 | 0.0353982300884956 | 8.21807057795258 | 5.06175096454621 | 0.001 | 0.013 | 0.009 | CD74/HAVCR2/IL4R/VSIG4 | 4 |
| GO:0036215 | response to stem cell factor | 3/81 | 52/18805 | 0.0576923076923077 | 13.3938746438746 | 5.88629073206795 | 0.001 | 0.013 | 0.009 | EGFR/EPHA2/FGFR2 | 3 |
| GO:0036216 | cellular response to stem cell factor stimulus | 3/81 | 52/18805 | 0.0576923076923077 | 13.3938746438746 | 5.88629073206795 | 0.001 | 0.013 | 0.009 | EGFR/EPHA2/FGFR2 | 3 |
| GO:0038109 | Kit signaling pathway | 3/81 | 52/18805 | 0.0576923076923077 | 13.3938746438746 | 5.88629073206795 | 0.001 | 0.013 | 0.009 | EGFR/EPHA2/FGFR2 | 3 |
| GO:0050856 | regulation of T cell receptor signaling pathway | 3/81 | 52/18805 | 0.0576923076923077 | 13.3938746438746 | 5.88629073206795 | 0.001 | 0.013 | 0.009 | CD300A/LILRB4/NECTIN2 | 3 |
| GO:0001776 | leukocyte homeostasis | 4/81 | 114/18805 | 0.0350877192982456 | 8.14598223954949 | 5.03345747542847 | 0.001 | 0.013 | 0.009 | CD74/CSF1/IL2RA/LGALS9 | 4 |
| GO:1903900 | regulation of viral life cycle | 4/81 | 115/18805 | 0.0347826086956522 | 8.0751476113795 | 5.00550718484252 | 0.002 | 0.013 | 0.009 | BST2/CD74/LGALS9/NECTIN2 | 4 |
| GO:0042269 | regulation of natural killer cell mediated cytotoxicity | 3/81 | 53/18805 | 0.0566037735849057 | 13.1411600279525 | 5.8216036676011 | 0.002 | 0.013 | 0.009 | HAVCR2/LGALS9/NECTIN2 | 3 |
| GO:0043303 | mast cell degranulation | 3/81 | 53/18805 | 0.0566037735849057 | 13.1411600279525 | 5.8216036676011 | 0.002 | 0.013 | 0.009 | CD300A/IL4R/LGALS9 | 3 |
| GO:0002444 | myeloid leukocyte mediated immunity | 4/81 | 116/18805 | 0.0344827586206897 | 8.00553426990209 | 4.97789279118689 | 0.002 | 0.013 | 0.009 | CD300A/IL4R/LGALS9/SPON2 | 4 |
| GO:0070555 | response to interleukin-1 | 4/81 | 116/18805 | 0.0344827586206897 | 8.00553426990209 | 4.97789279118689 | 0.002 | 0.013 | 0.009 | CHI3L1/INHBB/LGALS9/TNFRSF11A | 4 |
| GO:0043549 | regulation of kinase activity | 7/81 | 397/18805 | 0.017632241813602 | 4.09350996672575 | 4.09743228060502 | 0.002 | 0.013 | 0.010 | CD300A/CD74/CHI3L1/EGFR/LILRB4/TNFRSF10A/TNFRSF11A | 7 |
| GO:0002291 | T cell activation via T cell receptor contact with antigen bound to MHC molecule on antigen presenting cell | 2/81 | 14/18805 | 0.142857142857143 | 33.1657848324515 | 7.91865712382115 | 0.002 | 0.013 | 0.010 | HAVCR2/LGALS9 | 2 |
| GO:1904748 | regulation of apoptotic process involved in development | 2/81 | 14/18805 | 0.142857142857143 | 33.1657848324515 | 7.91865712382115 | 0.002 | 0.013 | 0.010 | TNFRSF1A/TNFRSF1B | 2 |
| GO:0045581 | negative regulation of T cell differentiation | 3/81 | 54/18805 | 0.0555555555555556 | 12.89780521262 | 5.75863870603524 | 0.002 | 0.013 | 0.010 | CD74/IL4R/LILRB4 | 3 |
| GO:0031547 | brain-derived neurotrophic factor receptor signaling pathway | 3/81 | 55/18805 | 0.0545454545454545 | 12.6632996632997 | 5.69731795557437 | 0.002 | 0.014 | 0.010 | EGFR/EPHA2/FGFR2 | 3 |
| GO:0035790 | platelet-derived growth factor receptor-alpha signaling pathway | 3/81 | 55/18805 | 0.0545454545454545 | 12.6632996632997 | 5.69731795557437 | 0.002 | 0.014 | 0.010 | EGFR/EPHA2/FGFR2 | 3 |
| GO:1902106 | negative regulation of leukocyte differentiation | 4/81 | 120/18805 | 0.0333333333333333 | 7.73868312757202 | 4.87065559338133 | 0.002 | 0.015 | 0.010 | CD74/FSTL3/IL4R/LILRB4 | 4 |
| GO:0002520 | immune system development | 5/81 | 202/18805 | 0.0247524752475248 | 5.74654687690991 | 4.46098004436312 | 0.002 | 0.015 | 0.010 | HAVCR2/IL2RA/LGALS9/LILRB4/TNFRSF11A | 5 |
| GO:0002279 | mast cell activation involved in immune response | 3/81 | 56/18805 | 0.0535714285714286 | 12.4371693121693 | 5.63756839457613 | 0.002 | 0.015 | 0.010 | CD300A/IL4R/LGALS9 | 3 |
| GO:0002517 | T cell tolerance induction | 2/81 | 15/18805 | 0.133333333333333 | 30.9547325102881 | 7.63336448068856 | 0.002 | 0.015 | 0.011 | IL2RA/LILRB4 | 2 |
| GO:0035860 | glial cell-derived neurotrophic factor receptor signaling pathway | 2/81 | 15/18805 | 0.133333333333333 | 30.9547325102881 | 7.63336448068856 | 0.002 | 0.015 | 0.011 | GDF15/GFRA1 | 2 |
| GO:0046007 | negative regulation of activated T cell proliferation | 2/81 | 15/18805 | 0.133333333333333 | 30.9547325102881 | 7.63336448068856 | 0.002 | 0.015 | 0.011 | LGALS9/LILRB4 | 2 |
| GO:0046598 | positive regulation of viral entry into host cell | 2/81 | 15/18805 | 0.133333333333333 | 30.9547325102881 | 7.63336448068856 | 0.002 | 0.015 | 0.011 | CD74/LGALS9 | 2 |
| GO:0075294 | positive regulation by symbiont of entry into host | 2/81 | 15/18805 | 0.133333333333333 | 30.9547325102881 | 7.63336448068856 | 0.002 | 0.015 | 0.011 | CD74/LGALS9 | 2 |
| GO:0002686 | negative regulation of leukocyte migration | 3/81 | 57/18805 | 0.0526315789473684 | 12.2189733593242 | 5.5793214862355 | 0.002 | 0.015 | 0.011 | CD300A/GDF15/NBL1 | 3 |
| GO:0002886 | regulation of myeloid leukocyte mediated immunity | 3/81 | 57/18805 | 0.0526315789473684 | 12.2189733593242 | 5.5793214862355 | 0.002 | 0.015 | 0.011 | CD300A/IL4R/LGALS9 | 3 |
| GO:0071621 | granulocyte chemotaxis | 4/81 | 122/18805 | 0.0327868852459016 | 7.61181946974297 | 4.81887776200673 | 0.002 | 0.015 | 0.011 | CCL11/CD74/CSF1/CXCL9 | 4 |
| GO:0038063 | collagen-activated tyrosine kinase receptor signaling pathway | 3/81 | 58/18805 | 0.0517241379310345 | 12.0083014048531 | 5.52251282991429 | 0.002 | 0.016 | 0.011 | EGFR/EPHA2/FGFR2 | 3 |
| GO:0002715 | regulation of natural killer cell mediated immunity | 3/81 | 59/18805 | 0.0508474576271187 | 11.8047708725675 | 5.46708184506583 | 0.002 | 0.016 | 0.012 | HAVCR2/LGALS9/NECTIN2 | 3 |
| GO:0060135 | maternal process involved in female pregnancy | 3/81 | 59/18805 | 0.0508474576271187 | 11.8047708725675 | 5.46708184506583 | 0.002 | 0.016 | 0.012 | ACE2/HAVCR2/LGALS9 | 3 |
| GO:0046642 | negative regulation of alpha-beta T cell proliferation | 2/81 | 16/18805 | 0.125 | 29.0200617283951 | 7.37472041472707 | 0.002 | 0.016 | 0.012 | LGALS9/TNFRSF14 | 2 |
| GO:0044703 | multi-organism reproductive process | 5/81 | 211/18805 | 0.023696682464455 | 5.50143350301328 | 4.32487906309003 | 0.002 | 0.017 | 0.012 | ACE2/ADM/AMBP/HAVCR2/LGALS9 | 5 |
| GO:0048012 | hepatocyte growth factor receptor signaling pathway | 3/81 | 60/18805 | 0.05 | 11.608024691358 | 5.41297148421225 | 0.002 | 0.017 | 0.012 | EGFR/EPHA2/FGFR2 | 3 |
| GO:0002688 | regulation of leukocyte chemotaxis | 4/81 | 127/18805 | 0.031496062992126 | 7.31214153786332 | 4.69441757367993 | 0.002 | 0.017 | 0.012 | CD74/CSF1/LGALS9/NBL1 | 4 |
| GO:1903707 | negative regulation of hemopoiesis | 4/81 | 127/18805 | 0.031496062992126 | 7.31214153786332 | 4.69441757367993 | 0.002 | 0.017 | 0.012 | CD74/FSTL3/IL4R/LILRB4 | 4 |
| GO:0001961 | positive regulation of cytokine-mediated signaling pathway | 3/81 | 61/18805 | 0.0491803278688525 | 11.4177292046145 | 5.36012797186882 | 0.002 | 0.018 | 0.012 | CD74/CSF1/MMP12 | 3 |
| GO:0014009 | glial cell proliferation | 3/81 | 61/18805 | 0.0491803278688525 | 11.4177292046145 | 5.36012797186882 | 0.002 | 0.018 | 0.012 | AREG/CSF1/EGFR | 3 |
| GO:0070231 | T cell apoptotic process | 3/81 | 61/18805 | 0.0491803278688525 | 11.4177292046145 | 5.36012797186882 | 0.002 | 0.018 | 0.012 | CD27/IL2RA/LGALS9 | 3 |
| GO:0000768 | syncytium formation by plasma membrane fusion | 3/81 | 62/18805 | 0.0483870967741936 | 11.2335722819594 | 5.30850056668692 | 0.002 | 0.018 | 0.013 | CXCL9/GDF15/IL4R | 3 |
| GO:0070228 | regulation of lymphocyte apoptotic process | 3/81 | 62/18805 | 0.0483870967741936 | 11.2335722819594 | 5.30850056668692 | 0.002 | 0.018 | 0.013 | CD27/CD74/LGALS9 | 3 |
| GO:0140253 | cell-cell fusion | 3/81 | 62/18805 | 0.0483870967741936 | 11.2335722819594 | 5.30850056668692 | 0.002 | 0.018 | 0.013 | CXCL9/GDF15/IL4R | 3 |
| GO:0045471 | response to ethanol | 4/81 | 131/18805 | 0.0305343511450382 | 7.08887004052398 | 4.59962038162292 | 0.002 | 0.018 | 0.013 | CD27/FGFR2/SPINK1/TNFRSF11A | 4 |
| GO:0035791 | platelet-derived growth factor receptor-beta signaling pathway | 3/81 | 63/18805 | 0.0476190476190476 | 11.0552616108172 | 5.25804134441284 | 0.003 | 0.019 | 0.013 | EGFR/EPHA2/FGFR2 | 3 |
| GO:0043370 | regulation of CD4-positive, alpha-beta T cell differentiation | 3/81 | 63/18805 | 0.0476190476190476 | 11.0552616108172 | 5.25804134441284 | 0.003 | 0.019 | 0.013 | CD80/IL4R/LGALS9 | 3 |
| GO:0002366 | leukocyte activation involved in immune response | 6/81 | 319/18805 | 0.0188087774294671 | 4.36665505631023 | 3.98878660740146 | 0.003 | 0.019 | 0.013 | CD300A/CD74/CD80/HAVCR2/IL4R/LGALS9 | 6 |
| GO:0044706 | multi-multicellular organism process | 5/81 | 219/18805 | 0.0228310502283105 | 5.30046789559727 | 4.21030071025416 | 0.003 | 0.019 | 0.013 | ACE2/ADM/AMBP/HAVCR2/LGALS9 | 5 |
| GO:0045637 | regulation of myeloid cell differentiation | 5/81 | 220/18805 | 0.0227272727272727 | 5.27637485970819 | 4.19637353938406 | 0.003 | 0.019 | 0.014 | CD74/CSF1/FSTL3/LILRB4/TNFRSF11A | 5 |
| GO:0045834 | positive regulation of lipid metabolic process | 4/81 | 133/18805 | 0.0300751879699248 | 6.98227049104242 | 4.55370362659331 | 0.003 | 0.019 | 0.014 | ADM/CD74/GDF15/TNFRSF1A | 4 |
| GO:0002763 | positive regulation of myeloid leukocyte differentiation | 3/81 | 64/18805 | 0.046875 | 10.8825231481481 | 5.20870499954232 | 0.003 | 0.019 | 0.014 | CD74/CSF1/TNFRSF11A | 3 |
| GO:0045620 | negative regulation of lymphocyte differentiation | 3/81 | 64/18805 | 0.046875 | 10.8825231481481 | 5.20870499954232 | 0.003 | 0.019 | 0.014 | CD74/IL4R/LILRB4 | 3 |
| GO:0001991 | regulation of systemic arterial blood pressure by circulatory renin-angiotensin | 2/81 | 18/18805 | 0.111111111111111 | 25.7956104252401 | 6.92230377394258 | 0.003 | 0.019 | 0.014 | ACE2/REN | 2 |
| GO:0002830 | positive regulation of type 2 immune response | 2/81 | 18/18805 | 0.111111111111111 | 25.7956104252401 | 6.92230377394258 | 0.003 | 0.019 | 0.014 | CD74/IL4R | 2 |
| GO:0050864 | regulation of B cell activation | 4/81 | 134/18805 | 0.0298507462686567 | 6.93016399484061 | 4.53109979977113 | 0.003 | 0.019 | 0.014 | CD27/CD300A/CD74/TNFRSF4 | 4 |
| GO:0002263 | cell activation involved in immune response | 6/81 | 323/18805 | 0.0185758513931889 | 4.31257883270267 | 3.9496745818066 | 0.003 | 0.019 | 0.014 | CD300A/CD74/CD80/HAVCR2/IL4R/LGALS9 | 6 |
| GO:0009612 | response to mechanical stimulus | 5/81 | 223/18805 | 0.0224215246636771 | 5.20539223827714 | 4.15509566214195 | 0.003 | 0.020 | 0.014 | CHI3L1/EGFR/TNFRSF10A/TNFRSF11A/TNFRSF1A | 5 |
| GO:0033674 | positive regulation of kinase activity | 5/81 | 223/18805 | 0.0224215246636771 | 5.20539223827714 | 4.15509566214195 | 0.003 | 0.020 | 0.014 | CD74/CHI3L1/EGFR/TNFRSF10A/TNFRSF11A | 5 |
| GO:0006518 | peptide metabolic process | 3/81 | 65/18805 | 0.0461538461538462 | 10.7150997150997 | 5.16044866379593 | 0.003 | 0.020 | 0.014 | GGT1/LGALS4/MMP7 | 3 |
| GO:0022617 | extracellular matrix disassembly | 3/81 | 65/18805 | 0.0461538461538462 | 10.7150997150997 | 5.16044866379593 | 0.003 | 0.020 | 0.014 | CST3/MMP12/MMP7 | 3 |
| GO:0032757 | positive regulation of interleukin-8 production | 3/81 | 65/18805 | 0.0461538461538462 | 10.7150997150997 | 5.16044866379593 | 0.003 | 0.020 | 0.014 | CD74/CHI3L1/LGALS9 | 3 |
| GO:0051384 | response to glucocorticoid | 4/81 | 136/18805 | 0.0294117647058824 | 6.8282498184459 | 4.48657983042359 | 0.003 | 0.020 | 0.014 | ADM/AREG/EGFR/NEFL | 4 |
| GO:0006949 | syncytium formation | 3/81 | 66/18805 | 0.0454545454545455 | 10.5527497194164 | 5.11323173975428 | 0.003 | 0.020 | 0.014 | CXCL9/GDF15/IL4R | 3 |
| GO:1901224 | positive regulation of non-canonical NF-kappaB signal transduction | 3/81 | 66/18805 | 0.0454545454545455 | 10.5527497194164 | 5.11323173975428 | 0.003 | 0.020 | 0.014 | CD27/HAVCR2/LGALS9 | 3 |
| GO:0002456 | T cell mediated immunity | 4/81 | 137/18805 | 0.0291970802919708 | 6.7784085788952 | 4.46465544768781 | 0.003 | 0.020 | 0.014 | CD80/LILRB4/NECTIN2/TNFRSF1B | 4 |
| GO:0016485 | protein processing | 5/81 | 227/18805 | 0.0220264317180617 | 5.11366726491543 | 4.10119977190731 | 0.003 | 0.021 | 0.015 | ACE2/CTSL/GGT1/PLAUR/REN | 5 |
| GO:0045780 | positive regulation of bone resorption | 2/81 | 19/18805 | 0.105263157894737 | 24.4379467186485 | 6.72275862551942 | 0.003 | 0.021 | 0.015 | EGFR/TNFRSF11A | 2 |
| GO:0051132 | NK T cell activation | 2/81 | 19/18805 | 0.105263157894737 | 24.4379467186485 | 6.72275862551942 | 0.003 | 0.021 | 0.015 | CD300A/NCR3LG1 | 2 |
| GO:0030888 | regulation of B cell proliferation | 3/81 | 68/18805 | 0.0441176470588235 | 10.2423747276688 | 5.02176418770374 | 0.003 | 0.022 | 0.015 | CD300A/CD74/TNFRSF4 | 3 |
| GO:0060760 | positive regulation of response to cytokine stimulus | 3/81 | 68/18805 | 0.0441176470588235 | 10.2423747276688 | 5.02176418770374 | 0.003 | 0.022 | 0.015 | CD74/CSF1/MMP12 | 3 |
| GO:0071674 | mononuclear cell migration | 5/81 | 230/18805 | 0.0217391304347826 | 5.04696725711218 | 4.06160338095881 | 0.003 | 0.022 | 0.015 | CSF1/LGALS9/NBL1/TNFRSF11A/TNFRSF14 | 5 |
| GO:0002285 | lymphocyte activation involved in immune response | 5/81 | 231/18805 | 0.0216450216450216 | 5.0251189140078 | 4.04855738155469 | 0.003 | 0.022 | 0.016 | CD74/CD80/HAVCR2/IL4R/LGALS9 | 5 |
| GO:0071222 | cellular response to lipopolysaccharide | 5/81 | 232/18805 | 0.021551724137931 | 5.0034589186888 | 4.03558643844722 | 0.003 | 0.022 | 0.016 | CD80/CXCL9/HAVCR2/SPON2/TNFRSF1B | 5 |
| GO:0019229 | regulation of vasoconstriction | 3/81 | 69/18805 | 0.0434782608695652 | 10.0939345142244 | 4.97744240573956 | 0.003 | 0.022 | 0.016 | ACE2/ADM/EGFR | 3 |
| GO:0050792 | regulation of viral process | 4/81 | 142/18805 | 0.028169014084507 | 6.53973222048339 | 4.35823386449963 | 0.003 | 0.022 | 0.016 | BST2/CD74/LGALS9/NECTIN2 | 4 |
| GO:0045655 | regulation of monocyte differentiation | 2/81 | 20/18805 | 0.1 | 23.2160493827161 | 6.53799440684694 | 0.003 | 0.022 | 0.016 | CD74/CSF1 | 2 |
| GO:0060602 | branch elongation of an epithelium | 2/81 | 20/18805 | 0.1 | 23.2160493827161 | 6.53799440684694 | 0.003 | 0.022 | 0.016 | AREG/FGFR2 | 2 |
| GO:0060749 | mammary gland alveolus development | 2/81 | 20/18805 | 0.1 | 23.2160493827161 | 6.53799440684694 | 0.003 | 0.022 | 0.016 | AREG/TNFRSF11A | 2 |
| GO:0061377 | mammary gland lobule development | 2/81 | 20/18805 | 0.1 | 23.2160493827161 | 6.53799440684694 | 0.003 | 0.022 | 0.016 | AREG/TNFRSF11A | 2 |
| GO:0060562 | epithelial tube morphogenesis | 6/81 | 337/18805 | 0.0178041543026706 | 4.13342125508298 | 3.81761490007059 | 0.003 | 0.022 | 0.016 | ADM/AREG/CCL11/CSF1/EPHA2/FGFR2 | 6 |
| GO:0048002 | antigen processing and presentation of peptide antigen | 3/81 | 71/18805 | 0.0422535211267606 | 9.80959833072509 | 4.89145810644601 | 0.004 | 0.024 | 0.017 | CD74/CTSL/IFI30 | 3 |
| GO:0097191 | extrinsic apoptotic signaling pathway | 5/81 | 237/18805 | 0.0210970464135021 | 4.89790071365318 | 3.97183040822505 | 0.004 | 0.024 | 0.017 | CD27/KRT18/TNFRSF10A/TNFRSF1A/TNFRSF1B | 5 |
| GO:0019216 | regulation of lipid metabolic process | 6/81 | 343/18805 | 0.0174927113702624 | 4.0611165100961 | 3.76318752959984 | 0.004 | 0.024 | 0.017 | ADM/CD74/GDF15/NPC2/SCARB2/TNFRSF1A | 6 |
| GO:0090713 | immunological memory process | 2/81 | 21/18805 | 0.0952380952380952 | 22.1105232216343 | 6.36623871627512 | 0.004 | 0.024 | 0.017 | CD27/TNFRSF14 | 2 |
| GO:0006909 | phagocytosis | 5/81 | 238/18805 | 0.0210084033613445 | 4.87732129888993 | 3.95929366587832 | 0.004 | 0.024 | 0.017 | CD300A/CD302/COLEC12/HAVCR1/SPON2 | 5 |
| GO:0051338 | regulation of transferase activity | 7/81 | 463/18805 | 0.0151187904967603 | 3.50998586779724 | 3.59672092584318 | 0.004 | 0.025 | 0.018 | CD300A/CD74/CHI3L1/EGFR/LILRB4/TNFRSF10A/TNFRSF11A | 7 |
| GO:0071675 | regulation of mononuclear cell migration | 4/81 | 148/18805 | 0.027027027027027 | 6.27460794127461 | 4.23709743187106 | 0.004 | 0.025 | 0.018 | CSF1/LGALS9/NBL1/TNFRSF14 | 4 |
| GO:0050854 | regulation of antigen receptor-mediated signaling pathway | 3/81 | 73/18805 | 0.0410958904109589 | 9.54084221207509 | 4.80881830952506 | 0.004 | 0.025 | 0.018 | CD300A/LILRB4/NECTIN2 | 3 |
| GO:0035739 | CD4-positive, alpha-beta T cell proliferation | 2/81 | 22/18805 | 0.0909090909090909 | 21.1054994388328 | 6.20600386142926 | 0.004 | 0.025 | 0.018 | IL2RA/LGALS9 | 2 |
| GO:0060713 | labyrinthine layer morphogenesis | 2/81 | 22/18805 | 0.0909090909090909 | 21.1054994388328 | 6.20600386142926 | 0.004 | 0.025 | 0.018 | ADM/FGFR2 | 2 |
| GO:1905038 | regulation of membrane lipid metabolic process | 2/81 | 22/18805 | 0.0909090909090909 | 21.1054994388328 | 6.20600386142926 | 0.004 | 0.025 | 0.018 | SCARB2/TNFRSF1A | 2 |
| GO:2000561 | regulation of CD4-positive, alpha-beta T cell proliferation | 2/81 | 22/18805 | 0.0909090909090909 | 21.1054994388328 | 6.20600386142926 | 0.004 | 0.025 | 0.018 | IL2RA/LGALS9 | 2 |
| GO:0071260 | cellular response to mechanical stimulus | 3/81 | 74/18805 | 0.0405405405405405 | 9.41191191191191 | 4.76868250678436 | 0.004 | 0.025 | 0.018 | EGFR/TNFRSF10A/TNFRSF1A | 3 |
| GO:1903053 | regulation of extracellular matrix organization | 3/81 | 74/18805 | 0.0405405405405405 | 9.41191191191191 | 4.76868250678436 | 0.004 | 0.025 | 0.018 | CST3/TNFRSF1A/TNFRSF1B | 3 |
| GO:0071496 | cellular response to external stimulus | 3/81 | 75/18805 | 0.04 | 9.28641975308642 | 4.72930133233491 | 0.004 | 0.026 | 0.019 | EGFR/TNFRSF10A/TNFRSF1A | 3 |
| GO:0071219 | cellular response to molecule of bacterial origin | 5/81 | 246/18805 | 0.0203252032520325 | 4.71870922412928 | 3.8614534447228 | 0.004 | 0.027 | 0.019 | CD80/CXCL9/HAVCR2/SPON2/TNFRSF1B | 5 |
| GO:0002687 | positive regulation of leukocyte migration | 4/81 | 152/18805 | 0.0263157894736842 | 6.10948667966212 | 4.15999716461916 | 0.004 | 0.027 | 0.019 | CD74/CSF1/LGALS9/TNFRSF14 | 4 |
| GO:0038128 | ERBB2 signaling pathway | 2/81 | 23/18805 | 0.0869565217391304 | 20.1878690284487 | 6.05603058445721 | 0.004 | 0.027 | 0.019 | AREG/EGFR | 2 |
| GO:2000272 | negative regulation of signaling receptor activity | 2/81 | 23/18805 | 0.0869565217391304 | 20.1878690284487 | 6.05603058445721 | 0.004 | 0.027 | 0.019 | ACE2/LILRB4 | 2 |
| GO:0043603 | amide metabolic process | 7/81 | 480/18805 | 0.0145833333333333 | 3.38567386831276 | 3.48239487500423 | 0.005 | 0.029 | 0.021 | ACE2/CTSL/GGT1/PLA2G15/REN/SCARB2/TNFRSF1A | 7 |
| GO:0031960 | response to corticosteroid | 4/81 | 157/18805 | 0.0254777070063694 | 5.91491704018243 | 4.06741234207038 | 0.005 | 0.029 | 0.021 | ADM/AREG/EGFR/NEFL | 4 |
| GO:0045088 | regulation of innate immune response | 7/81 | 482/18805 | 0.0145228215767635 | 3.37162542902515 | 3.46928230551451 | 0.005 | 0.029 | 0.021 | CD300A/COLEC12/HAVCR2/LGALS9/MMP12/NECTIN2/VSIG4 | 7 |
| GO:0003081 | regulation of systemic arterial blood pressure by renin-angiotensin | 2/81 | 24/18805 | 0.0833333333333333 | 19.34670781893 | 5.91524481623064 | 0.005 | 0.029 | 0.021 | ACE2/REN | 2 |
| GO:0003177 | pulmonary valve development | 2/81 | 24/18805 | 0.0833333333333333 | 19.34670781893 | 5.91524481623064 | 0.005 | 0.029 | 0.021 | TNFRSF1A/TNFRSF1B | 2 |
| GO:0045063 | T-helper 1 cell differentiation | 2/81 | 24/18805 | 0.0833333333333333 | 19.34670781893 | 5.91524481623064 | 0.005 | 0.029 | 0.021 | CD80/IL4R | 2 |
| GO:0045591 | positive regulation of regulatory T cell differentiation | 2/81 | 24/18805 | 0.0833333333333333 | 19.34670781893 | 5.91524481623064 | 0.005 | 0.029 | 0.021 | LGALS9/LILRB4 | 2 |
| GO:0060571 | morphogenesis of an epithelial fold | 2/81 | 24/18805 | 0.0833333333333333 | 19.34670781893 | 5.91524481623064 | 0.005 | 0.029 | 0.021 | EGFR/FGFR2 | 2 |
| GO:0097066 | response to thyroid hormone | 2/81 | 24/18805 | 0.0833333333333333 | 19.34670781893 | 5.91524481623064 | 0.005 | 0.029 | 0.021 | CTSL/INHBB | 2 |
| GO:0032720 | negative regulation of tumor necrosis factor production | 3/81 | 80/18805 | 0.0375 | 8.70601851851852 | 4.54289232261821 | 0.005 | 0.030 | 0.022 | HAVCR2/LGALS9/LILRB4 | 3 |
| GO:0002790 | peptide secretion | 5/81 | 257/18805 | 0.0194552529182879 | 4.51674112504203 | 3.7335909283497 | 0.005 | 0.031 | 0.022 | CD74/EGFR/INHBB/MMP7/SPINK1 | 5 |
| GO:1901136 | carbohydrate derivative catabolic process | 5/81 | 258/18805 | 0.0193798449612403 | 4.49923437649536 | 3.72232563895186 | 0.005 | 0.031 | 0.022 | CHI3L1/CST3/CTSL/MMP12/SCARB2 | 5 |
| GO:0060907 | positive regulation of macrophage cytokine production | 2/81 | 25/18805 | 0.08 | 18.5728395061728 | 5.78272403759073 | 0.005 | 0.031 | 0.022 | CD74/SPON2 | 2 |
| GO:0072677 | eosinophil migration | 2/81 | 25/18805 | 0.08 | 18.5728395061728 | 5.78272403759073 | 0.005 | 0.031 | 0.022 | CCL11/CD300A | 2 |
| GO:1903306 | negative regulation of regulated secretory pathway | 2/81 | 25/18805 | 0.08 | 18.5728395061728 | 5.78272403759073 | 0.005 | 0.031 | 0.022 | CD300A/LGALS9 | 2 |
| GO:1903902 | positive regulation of viral life cycle | 2/81 | 25/18805 | 0.08 | 18.5728395061728 | 5.78272403759073 | 0.005 | 0.031 | 0.022 | CD74/LGALS9 | 2 |
| GO:0002260 | lymphocyte homeostasis | 3/81 | 81/18805 | 0.037037037037037 | 8.59853680841335 | 4.50755961558546 | 0.005 | 0.031 | 0.022 | CD74/IL2RA/LGALS9 | 3 |
| GO:0043299 | leukocyte degranulation | 3/81 | 81/18805 | 0.037037037037037 | 8.59853680841335 | 4.50755961558546 | 0.005 | 0.031 | 0.022 | CD300A/IL4R/LGALS9 | 3 |
| GO:0045859 | regulation of protein kinase activity | 6/81 | 369/18805 | 0.016260162601626 | 3.77496737930342 | 3.54083777666741 | 0.005 | 0.031 | 0.022 | CD300A/CHI3L1/EGFR/LILRB4/TNFRSF10A/TNFRSF11A | 6 |
| GO:0032869 | cellular response to insulin stimulus | 5/81 | 260/18805 | 0.0192307692307692 | 4.46462488129155 | 3.69996648003095 | 0.005 | 0.031 | 0.022 | BAIAP2/EGFR/EPHA2/FGFR2/INHBB | 5 |
| GO:1903556 | negative regulation of tumor necrosis factor superfamily cytokine production | 3/81 | 82/18805 | 0.0365853658536585 | 8.4936766034327 | 4.47283078940203 | 0.005 | 0.031 | 0.022 | HAVCR2/LGALS9/LILRB4 | 3 |
| GO:0042267 | natural killer cell mediated cytotoxicity | 3/81 | 83/18805 | 0.036144578313253 | 8.39134315037929 | 4.43868786113118 | 0.006 | 0.032 | 0.023 | HAVCR2/LGALS9/NECTIN2 | 3 |
| GO:0070098 | chemokine-mediated signaling pathway | 3/81 | 83/18805 | 0.036144578313253 | 8.39134315037929 | 4.43868786113118 | 0.006 | 0.032 | 0.023 | CCL11/CXCL9/TFF2 | 3 |
| GO:0001906 | cell killing | 5/81 | 263/18805 | 0.0190114068441065 | 4.41369760127682 | 3.66684833413797 | 0.006 | 0.032 | 0.023 | CCL11/CXCL9/HAVCR2/LGALS9/NECTIN2 | 5 |
| GO:0051347 | positive regulation of transferase activity | 5/81 | 263/18805 | 0.0190114068441065 | 4.41369760127682 | 3.66684833413797 | 0.006 | 0.032 | 0.023 | CD74/CHI3L1/EGFR/TNFRSF10A/TNFRSF11A | 5 |
| GO:0033598 | mammary gland epithelial cell proliferation | 2/81 | 26/18805 | 0.0769230769230769 | 17.8584995251662 | 5.65767082263203 | 0.006 | 0.032 | 0.023 | AREG/EPHA2 | 2 |
| GO:0034138 | toll-like receptor 3 signaling pathway | 2/81 | 26/18805 | 0.0769230769230769 | 17.8584995251662 | 5.65767082263203 | 0.006 | 0.032 | 0.023 | COLEC12/HAVCR2 | 2 |
| GO:0045624 | positive regulation of T-helper cell differentiation | 2/81 | 26/18805 | 0.0769230769230769 | 17.8584995251662 | 5.65767082263203 | 0.006 | 0.032 | 0.023 | CD80/IL4R | 2 |
| GO:0060561 | apoptotic process involved in morphogenesis | 2/81 | 26/18805 | 0.0769230769230769 | 17.8584995251662 | 5.65767082263203 | 0.006 | 0.032 | 0.023 | TNFRSF1A/TNFRSF1B | 2 |
| GO:0002720 | positive regulation of cytokine production involved in immune response | 3/81 | 85/18805 | 0.0352941176470588 | 8.19389978213508 | 4.37209146115429 | 0.006 | 0.034 | 0.024 | CD74/SPON2/TNFRSF14 | 3 |
| GO:0032418 | lysosome localization | 3/81 | 85/18805 | 0.0352941176470588 | 8.19389978213508 | 4.37209146115429 | 0.006 | 0.034 | 0.024 | CD300A/IL4R/LGALS9 | 3 |
| GO:0002837 | regulation of immune response to tumor cell | 2/81 | 27/18805 | 0.0740740740740741 | 17.1970736168267 | 5.53939181903496 | 0.006 | 0.034 | 0.024 | HAVCR2/NECTIN2 | 2 |
| GO:0032753 | positive regulation of interleukin-4 production | 2/81 | 27/18805 | 0.0740740740740741 | 17.1970736168267 | 5.53939181903496 | 0.006 | 0.034 | 0.024 | HAVCR2/LGALS9 | 2 |
| GO:0060669 | embryonic placenta morphogenesis | 2/81 | 27/18805 | 0.0740740740740741 | 17.1970736168267 | 5.53939181903496 | 0.006 | 0.034 | 0.024 | ADM/FGFR2 | 2 |
| GO:0050871 | positive regulation of B cell activation | 3/81 | 86/18805 | 0.0348837209302326 | 8.09862187769165 | 4.33960560213082 | 0.006 | 0.034 | 0.024 | CD27/CD74/TNFRSF4 | 3 |
| GO:0072332 | intrinsic apoptotic signaling pathway by p53 class mediator | 3/81 | 86/18805 | 0.0348837209302326 | 8.09862187769165 | 4.33960560213082 | 0.006 | 0.034 | 0.024 | CD74/EDA2R/SHISA5 | 3 |
| GO:1990849 | vacuolar localization | 3/81 | 86/18805 | 0.0348837209302326 | 8.09862187769165 | 4.33960560213082 | 0.006 | 0.034 | 0.024 | CD300A/IL4R/LGALS9 | 3 |
| GO:0001892 | embryonic placenta development | 3/81 | 87/18805 | 0.0344827586206897 | 8.00553426990209 | 4.30764079475137 | 0.006 | 0.035 | 0.025 | ADM/EGFR/FGFR2 | 3 |
| GO:0001894 | tissue homeostasis | 5/81 | 272/18805 | 0.0183823529411765 | 4.26765613652869 | 3.57039488789001 | 0.006 | 0.036 | 0.026 | CSF1/EGFR/OCLN/TFF2/TNFRSF11A | 5 |
| GO:0060249 | anatomical structure homeostasis | 5/81 | 272/18805 | 0.0183823529411765 | 4.26765613652869 | 3.57039488789001 | 0.006 | 0.036 | 0.026 | CSF1/EGFR/OCLN/TFF2/TNFRSF11A | 5 |
| GO:0002834 | regulation of response to tumor cell | 2/81 | 28/18805 | 0.0714285714285714 | 16.5828924162257 | 5.42728089212016 | 0.006 | 0.036 | 0.026 | HAVCR2/NECTIN2 | 2 |
| GO:0002228 | natural killer cell mediated immunity | 3/81 | 88/18805 | 0.0340909090909091 | 7.91456228956229 | 4.27618241937189 | 0.006 | 0.036 | 0.026 | HAVCR2/LGALS9/NECTIN2 | 3 |
| GO:0002294 | CD4-positive, alpha-beta T cell differentiation involved in immune response | 3/81 | 88/18805 | 0.0340909090909091 | 7.91456228956229 | 4.27618241937189 | 0.006 | 0.036 | 0.026 | CD80/IL4R/LGALS9 | 3 |
| GO:0034103 | regulation of tissue remodeling | 3/81 | 88/18805 | 0.0340909090909091 | 7.91456228956229 | 4.27618241937189 | 0.006 | 0.036 | 0.026 | CST3/EGFR/TNFRSF11A | 3 |
| GO:0071216 | cellular response to biotic stimulus | 5/81 | 273/18805 | 0.0183150183150183 | 4.25202369646814 | 3.55993602756831 | 0.006 | 0.036 | 0.026 | CD80/CXCL9/HAVCR2/SPON2/TNFRSF1B | 5 |
| GO:0002287 | alpha-beta T cell activation involved in immune response | 3/81 | 89/18805 | 0.0337078651685393 | 7.82563462338743 | 4.24521642906457 | 0.007 | 0.037 | 0.026 | CD80/IL4R/LGALS9 | 3 |
| GO:0002293 | alpha-beta T cell differentiation involved in immune response | 3/81 | 89/18805 | 0.0337078651685393 | 7.82563462338743 | 4.24521642906457 | 0.007 | 0.037 | 0.026 | CD80/IL4R/LGALS9 | 3 |
| GO:0042310 | vasoconstriction | 3/81 | 89/18805 | 0.0337078651685393 | 7.82563462338743 | 4.24521642906457 | 0.007 | 0.037 | 0.026 | ACE2/ADM/EGFR | 3 |
| GO:0003401 | axis elongation | 2/81 | 29/18805 | 0.0689655172413793 | 16.0110685398042 | 5.32080549148078 | 0.007 | 0.038 | 0.027 | AREG/FGFR2 | 2 |
| GO:0006910 | phagocytosis, recognition | 2/81 | 29/18805 | 0.0689655172413793 | 16.0110685398042 | 5.32080549148078 | 0.007 | 0.038 | 0.027 | COLEC12/SPON2 | 2 |
| GO:0008286 | insulin receptor signaling pathway | 4/81 | 176/18805 | 0.0227272727272727 | 5.27637485970819 | 3.74891544081094 | 0.007 | 0.038 | 0.027 | BAIAP2/EGFR/EPHA2/FGFR2 | 4 |
| GO:0046849 | bone remodeling | 3/81 | 91/18805 | 0.032967032967033 | 7.65364265364265 | 4.18470810961152 | 0.007 | 0.039 | 0.027 | EGFR/EPHA2/TNFRSF11A | 3 |
| GO:0051591 | response to cAMP | 3/81 | 91/18805 | 0.032967032967033 | 7.65364265364265 | 4.18470810961152 | 0.007 | 0.039 | 0.027 | AREG/INHBB/REN | 3 |
| GO:0032273 | positive regulation of protein polymerization | 3/81 | 92/18805 | 0.0326086956521739 | 7.57045088566828 | 4.15514030123631 | 0.007 | 0.039 | 0.028 | BAIAP2/CCL11/OCLN | 3 |
| GO:1990868 | response to chemokine | 3/81 | 92/18805 | 0.0326086956521739 | 7.57045088566828 | 4.15514030123631 | 0.007 | 0.039 | 0.028 | CCL11/CXCL9/TFF2 | 3 |
| GO:1990869 | cellular response to chemokine | 3/81 | 92/18805 | 0.0326086956521739 | 7.57045088566828 | 4.15514030123631 | 0.007 | 0.039 | 0.028 | CCL11/CXCL9/TFF2 | 3 |
| GO:0043032 | positive regulation of macrophage activation | 2/81 | 30/18805 | 0.0666666666666667 | 15.477366255144 | 5.21949553630586 | 0.007 | 0.039 | 0.028 | HAVCR2/IL4R | 2 |
| GO:0045672 | positive regulation of osteoclast differentiation | 2/81 | 30/18805 | 0.0666666666666667 | 15.477366255144 | 5.21949553630586 | 0.007 | 0.039 | 0.028 | CSF1/TNFRSF11A | 2 |
| GO:0015833 | peptide transport | 5/81 | 283/18805 | 0.0176678445229682 | 4.10177550931379 | 3.45802361603834 | 0.008 | 0.040 | 0.029 | CD74/EGFR/INHBB/MMP7/SPINK1 | 5 |
| GO:0042100 | B cell proliferation | 3/81 | 93/18805 | 0.032258064516129 | 7.48904818797292 | 4.12601387042562 | 0.008 | 0.040 | 0.029 | CD300A/CD74/TNFRSF4 | 3 |
| GO:0010721 | negative regulation of cell development | 5/81 | 285/18805 | 0.0175438596491228 | 4.07299111977475 | 3.43820334534876 | 0.008 | 0.041 | 0.029 | CCL11/CD74/FSTL3/IL4R/LILRB4 | 5 |
| GO:0050886 | endocrine process | 3/81 | 94/18805 | 0.0319148936170213 | 7.40937746256895 | 4.09731723751593 | 0.008 | 0.041 | 0.029 | ACE2/INHBB/REN | 3 |
| GO:0032607 | interferon-alpha production | 2/81 | 31/18805 | 0.0645161290322581 | 14.9780963759458 | 5.12293428717444 | 0.008 | 0.041 | 0.029 | HAVCR2/MMP12 | 2 |
| GO:0032647 | regulation of interferon-alpha production | 2/81 | 31/18805 | 0.0645161290322581 | 14.9780963759458 | 5.12293428717444 | 0.008 | 0.041 | 0.029 | HAVCR2/MMP12 | 2 |
| GO:0042113 | B cell activation | 5/81 | 287/18805 | 0.0174216027874564 | 4.04460790639652 | 3.4185630469543 | 0.008 | 0.042 | 0.030 | BST2/CD27/CD300A/CD74/TNFRSF4 | 5 |
| GO:0038084 | vascular endothelial growth factor signaling pathway | 3/81 | 95/18805 | 0.0315789473684211 | 7.33138401559454 | 4.06903924754852 | 0.008 | 0.042 | 0.030 | EGFR/EPHA2/FGFR2 | 3 |
| GO:0071677 | positive regulation of mononuclear cell migration | 3/81 | 95/18805 | 0.0315789473684211 | 7.33138401559454 | 4.06903924754852 | 0.008 | 0.042 | 0.030 | CSF1/LGALS9/TNFRSF14 | 3 |
| GO:2000106 | regulation of leukocyte apoptotic process | 3/81 | 95/18805 | 0.0315789473684211 | 7.33138401559454 | 4.06903924754852 | 0.008 | 0.042 | 0.030 | CD27/CD74/LGALS9 | 3 |
| GO:0002690 | positive regulation of leukocyte chemotaxis | 3/81 | 96/18805 | 0.03125 | 7.25501543209877 | 4.04116915038871 | 0.008 | 0.043 | 0.031 | CD74/CSF1/LGALS9 | 3 |
| GO:0002710 | negative regulation of T cell mediated immunity | 2/81 | 32/18805 | 0.0625 | 14.5100308641975 | 5.03075079780501 | 0.008 | 0.043 | 0.031 | CD80/LILRB4 | 2 |
| GO:0002292 | T cell differentiation involved in immune response | 3/81 | 97/18805 | 0.0309278350515464 | 7.18022145857197 | 4.01369658197243 | 0.008 | 0.044 | 0.031 | CD80/IL4R/LGALS9 | 3 |
| GO:0050678 | regulation of epithelial cell proliferation | 6/81 | 410/18805 | 0.0146341463414634 | 3.39747064137308 | 3.22822386973627 | 0.009 | 0.044 | 0.031 | AREG/CCL11/EGFR/FGFR2/MMP12/SPINK1 | 6 |
| GO:0072659 | protein localization to plasma membrane | 5/81 | 295/18805 | 0.0169491525423729 | 3.93492362418916 | 3.34174200064084 | 0.009 | 0.046 | 0.033 | EGFR/EPHA2/KRT18/SCARB2/TNFRSF1A | 5 |
| GO:0001959 | regulation of cytokine-mediated signaling pathway | 4/81 | 189/18805 | 0.0211640211640212 | 4.91344960480763 | 3.55644238145511 | 0.009 | 0.046 | 0.033 | CD74/CSF1/MMP12/TNFRSF1A | 4 |
| GO:1903532 | positive regulation of secretion by cell | 5/81 | 297/18805 | 0.0168350168350168 | 3.90842582200607 | 3.32295749043306 | 0.009 | 0.047 | 0.033 | EGFR/IL4R/INHBB/SPINK1/TNFRSF11A | 5 |
| GO:0001910 | regulation of leukocyte mediated cytotoxicity | 3/81 | 100/18805 | 0.03 | 6.96481481481481 | 3.93356583470216 | 0.009 | 0.047 | 0.034 | HAVCR2/LGALS9/NECTIN2 | 3 |
| GO:0032640 | tumor necrosis factor production | 4/81 | 191/18805 | 0.0209424083769634 | 4.86199987072587 | 3.5283966267779 | 0.009 | 0.047 | 0.034 | HAVCR2/LGALS9/LILRB4/SPON2 | 4 |
| GO:0032680 | regulation of tumor necrosis factor production | 4/81 | 191/18805 | 0.0209424083769634 | 4.86199987072587 | 3.5283966267779 | 0.009 | 0.047 | 0.034 | HAVCR2/LGALS9/LILRB4/SPON2 | 4 |
| GO:0002418 | immune response to tumor cell | 2/81 | 34/18805 | 0.0588235294117647 | 13.6564996368918 | 4.85822561125316 | 0.009 | 0.047 | 0.034 | HAVCR2/NECTIN2 | 2 |
| GO:0002828 | regulation of type 2 immune response | 2/81 | 34/18805 | 0.0588235294117647 | 13.6564996368918 | 4.85822561125316 | 0.009 | 0.047 | 0.034 | CD74/IL4R | 2 |
| GO:0007435 | salivary gland morphogenesis | 2/81 | 34/18805 | 0.0588235294117647 | 13.6564996368918 | 4.85822561125316 | 0.009 | 0.047 | 0.034 | EGFR/FGFR2 | 2 |
| GO:0045671 | negative regulation of osteoclast differentiation | 2/81 | 34/18805 | 0.0588235294117647 | 13.6564996368918 | 4.85822561125316 | 0.009 | 0.047 | 0.034 | FSTL3/LILRB4 | 2 |
| GO:0051654 | establishment of mitochondrion localization | 2/81 | 34/18805 | 0.0588235294117647 | 13.6564996368918 | 4.85822561125316 | 0.009 | 0.047 | 0.034 | NECTIN2/NEFL | 2 |
| GO:0048771 | tissue remodeling | 4/81 | 192/18805 | 0.0208333333333333 | 4.83667695473251 | 3.5145196320615 | 0.010 | 0.048 | 0.034 | CST3/EGFR/EPHA2/TNFRSF11A | 4 |
| GO:0003073 | regulation of systemic arterial blood pressure | 3/81 | 102/18805 | 0.0294117647058824 | 6.8282498184459 | 3.88195880376178 | 0.010 | 0.049 | 0.035 | ACE2/ADM/REN | 3 |
| GO:0032755 | positive regulation of interleukin-6 production | 3/81 | 102/18805 | 0.0294117647058824 | 6.8282498184459 | 3.88195880376178 | 0.010 | 0.049 | 0.035 | CD74/LGALS9/SPON2 | 3 |
| GO:0002719 | negative regulation of cytokine production involved in immune response | 2/81 | 35/18805 | 0.0571428571428571 | 13.2663139329806 | 4.77731937402334 | 0.010 | 0.049 | 0.035 | BST2/LILRB4 | 2 |
| GO:0032633 | interleukin-4 production | 2/81 | 35/18805 | 0.0571428571428571 | 13.2663139329806 | 4.77731937402334 | 0.010 | 0.049 | 0.035 | HAVCR2/LGALS9 | 2 |
| GO:0032673 | regulation of interleukin-4 production | 2/81 | 35/18805 | 0.0571428571428571 | 13.2663139329806 | 4.77731937402334 | 0.010 | 0.049 | 0.035 | HAVCR2/LGALS9 | 2 |
| GO:0048147 | negative regulation of fibroblast proliferation | 2/81 | 35/18805 | 0.0571428571428571 | 13.2663139329806 | 4.77731937402334 | 0.010 | 0.049 | 0.035 | CD300A/IFI30 | 2 |
| GO:0061081 | positive regulation of myeloid leukocyte cytokine production involved in immune response | 2/81 | 35/18805 | 0.0571428571428571 | 13.2663139329806 | 4.77731937402334 | 0.010 | 0.049 | 0.035 | CD74/SPON2 | 2 |

**Supplementary Table S9. Gene Ontology Molecular Function enrichment analysis**

GO Molecular Function enrichment of the 85 candidate mediator proteins. In total, 60 terms met FDR < 0.05. Gene-ratio denominators reflect proteins eligible for the respective ontology analysis.

| **ID** | **Description** | **Gene ratio** | **Background ratio** | **Rich factor** | **Fold enrichment** | **z score** | **P value** | **Adjusted P** | **q value** | **Gene IDs** | **Count** |
| --- | --- | --- | --- | --- | --- | --- | --- | --- | --- | --- | --- |
| GO:0005035 | death receptor activity | 7/84 | 13/18639 | 0.538461538461538 | 119.480769230769 | 28.7520330771541 | 4.91 × 10⁻¹⁴ | 1.134 × 10⁻¹¹ | 7.494 × 10⁻¹² | EDA2R/TNFRSF10A/TNFRSF11A/TNFRSF14/TNFRSF1A/TNFRSF1B/TNFRSF4 | 7 |
| GO:0005031 | tumor necrosis factor receptor activity | 6/84 | 10/18639 | 0.6 | 133.135714285714 | 28.1212267814124 | 1.446 × 10⁻¹² | 1.67 × 10⁻¹⁰ | 1.104 × 10⁻¹⁰ | EDA2R/TNFRSF11A/TNFRSF14/TNFRSF1A/TNFRSF1B/TNFRSF4 | 6 |
| GO:0001618 | virus receptor activity | 9/84 | 81/18639 | 0.111111111111111 | 24.6547619047619 | 14.3550372134082 | 9.902 × 10⁻¹¹ | 6.4 × 10⁻⁹ | 4.229 × 10⁻⁹ | ACE2/CD80/EGFR/EPHA2/HAVCR1/NECTIN2/SCARB2/TNFRSF14/TNFRSF4 | 9 |
| GO:0140272 | exogenous protein binding | 9/84 | 82/18639 | 0.109756097560976 | 24.3540940766551 | 14.260175980253 | 1.108 × 10⁻¹⁰ | 6.4 × 10⁻⁹ | 4.229 × 10⁻⁹ | ACE2/CD80/EGFR/EPHA2/HAVCR1/NECTIN2/SCARB2/TNFRSF14/TNFRSF4 | 9 |
| GO:0030246 | carbohydrate binding | 11/84 | 280/18639 | 0.0392857142857143 | 8.7172193877551 | 8.75435530278706 | 5.084 × 10⁻⁸ | 2.349 × 10⁻⁶ | 1.552 × 10⁻⁶ | AMBP/ASGR1/CD302/CHI3L1/COLEC12/FUOM/LAYN/LGALS4/LGALS9/REG1B/SIGLEC1 | 11 |
| GO:0019955 | cytokine binding | 8/84 | 144/18639 | 0.0555555555555556 | 12.327380952381 | 9.18105104864255 | 2.774 × 10⁻⁷ | 1.068 × 10⁻⁵ | 7.056 × 10⁻⁶ | CD74/IL18BP/IL2RA/NBL1/TNFRSF11A/TNFRSF14/TNFRSF1A/TNFRSF1B | 8 |
| GO:0140375 | immune receptor activity | 6/84 | 156/18639 | 0.0384615384615385 | 8.53434065934066 | 6.35814074640758 | 7.408 × 10⁻⁵ | 0.002 | 0.001 | CD74/GFRA1/IL2RA/IL4R/LILRB4/PIGR | 6 |
| GO:0005030 | neurotrophin receptor activity | 4/84 | 51/18639 | 0.0784313725490196 | 17.4033613445378 | 7.89242430748643 | 8.162 × 10⁻⁵ | 0.002 | 0.001 | EGFR/EPHA2/FGFR2/GFRA1 | 4 |
| GO:0004866 | endopeptidase inhibitor activity | 6/84 | 161/18639 | 0.0372670807453416 | 8.26929902395741 | 6.23285190524602 | 8.821 × 10⁻⁵ | 0.002 | 0.001 | AMBP/BST2/COL6A3/CST3/SPINK1/WFDC2 | 6 |
| GO:0005003 | ephrin receptor activity | 4/84 | 53/18639 | 0.0754716981132076 | 16.7466307277628 | 7.72398512620497 | 9.499 × 10⁻⁵ | 0.002 | 0.001 | EFNA4/EGFR/EPHA2/FGFR2 | 4 |
| GO:0005005 | transmembrane-ephrin receptor activity | 4/84 | 53/18639 | 0.0754716981132076 | 16.7466307277628 | 7.72398512620497 | 9.499 × 10⁻⁵ | 0.002 | 0.001 | EFNA4/EGFR/EPHA2/FGFR2 | 4 |
| GO:0038064 | collagen receptor activity | 4/84 | 53/18639 | 0.0754716981132076 | 16.7466307277628 | 7.72398512620497 | 9.499 × 10⁻⁵ | 0.002 | 0.001 | EGFR/EPHA2/FGFR2/ITGA11 | 4 |
| GO:0030414 | peptidase inhibitor activity | 6/84 | 169/18639 | 0.0355029585798817 | 7.87785291631446 | 6.04326454690716 | 1.152 × 10⁻⁴ | 0.002 | 0.001 | AMBP/BST2/COL6A3/CST3/SPINK1/WFDC2 | 6 |
| GO:0004714 | transmembrane receptor protein tyrosine kinase activity | 4/84 | 60/18639 | 0.0666666666666667 | 14.7928571428571 | 7.19991738733032 | 1.544 × 10⁻⁴ | 0.003 | 0.002 | EFNA4/EGFR/EPHA2/FGFR2 | 4 |
| GO:0061135 | endopeptidase regulator activity | 6/84 | 184/18639 | 0.0326086956521739 | 7.23563664596273 | 5.71928192116398 | 1.832 × 10⁻⁴ | 0.003 | 0.002 | AMBP/BST2/COL6A3/CST3/SPINK1/WFDC2 | 6 |
| GO:0001968 | fibronectin binding | 3/84 | 30/18639 | 0.1 | 22.1892857142857 | 7.81491473157479 | 3.283 × 10⁻⁴ | 0.005 | 0.003 | CTSL/FSTL3/LILRB4 | 3 |
| GO:0048029 | monosaccharide binding | 4/84 | 74/18639 | 0.0540540540540541 | 11.9942084942085 | 6.37589518756676 | 3.474 × 10⁻⁴ | 0.005 | 0.003 | ASGR1/COLEC12/FUOM/LGALS9 | 4 |
| GO:0019838 | growth factor binding | 5/84 | 136/18639 | 0.0367647058823529 | 8.1578256302521 | 5.63687379744226 | 3.737 × 10⁻⁴ | 0.005 | 0.003 | EGFR/EPHA2/FGFR2/IGFBP4/IL2RA | 5 |
| GO:0098631 | cell adhesion mediator activity | 4/84 | 76/18639 | 0.0526315789473684 | 11.6785714285714 | 6.27631436474473 | 3.847 × 10⁻⁴ | 0.005 | 0.003 | BAIAP2/CNTN5/ITGA11/KRT18 | 4 |
| GO:0019199 | transmembrane receptor protein kinase activity | 4/84 | 78/18639 | 0.0512820512820513 | 11.3791208791209 | 6.1803917381886 | 4.248 × 10⁻⁴ | 0.005 | 0.003 | EFNA4/EGFR/EPHA2/FGFR2 | 4 |
| GO:0061134 | peptidase regulator activity | 6/84 | 226/18639 | 0.0265486725663717 | 5.8909608091024 | 4.97731289560179 | 5.499 × 10⁻⁴ | 0.006 | 0.004 | AMBP/BST2/COL6A3/CST3/SPINK1/WFDC2 | 6 |
| GO:0005125 | cytokine activity | 6/84 | 234/18639 | 0.0256410256410256 | 5.68956043956044 | 4.85714392676476 | 6.599 × 10⁻⁴ | 0.007 | 0.005 | AREG/CCL11/CSF1/CXCL9/GDF15/INHBB | 6 |
| GO:0004867 | serine-type endopeptidase inhibitor activity | 4/84 | 95/18639 | 0.0421052631578947 | 9.34285714285714 | 5.48508949349252 | 8.947 × 10⁻⁴ | 0.009 | 0.006 | AMBP/COL6A3/SPINK1/WFDC2 | 4 |
| GO:0004896 | cytokine receptor activity | 4/84 | 96/18639 | 0.0416666666666667 | 9.24553571428571 | 5.44970898914054 | 9.304 × 10⁻⁴ | 0.009 | 0.006 | CD74/GFRA1/IL2RA/IL4R | 4 |
| GO:0005004 | GPI-linked ephrin receptor activity | 3/84 | 49/18639 | 0.0612244897959184 | 13.58527696793 | 5.93512595752481 | 0.001 | 0.009 | 0.006 | EGFR/EPHA2/FGFR2 | 3 |
| GO:0005008 | hepatocyte growth factor receptor activity | 3/84 | 49/18639 | 0.0612244897959184 | 13.58527696793 | 5.93512595752481 | 0.001 | 0.009 | 0.006 | EGFR/EPHA2/FGFR2 | 3 |
| GO:0005009 | insulin receptor activity | 3/84 | 49/18639 | 0.0612244897959184 | 13.58527696793 | 5.93512595752481 | 0.001 | 0.009 | 0.006 | EGFR/EPHA2/FGFR2 | 3 |
| GO:0005011 | macrophage colony-stimulating factor receptor activity | 3/84 | 49/18639 | 0.0612244897959184 | 13.58527696793 | 5.93512595752481 | 0.001 | 0.009 | 0.006 | EGFR/EPHA2/FGFR2 | 3 |
| GO:0005018 | platelet-derived growth factor alpha-receptor activity | 3/84 | 49/18639 | 0.0612244897959184 | 13.58527696793 | 5.93512595752481 | 0.001 | 0.009 | 0.006 | EGFR/EPHA2/FGFR2 | 3 |
| GO:0005020 | stem cell factor receptor activity | 3/84 | 49/18639 | 0.0612244897959184 | 13.58527696793 | 5.93512595752481 | 0.001 | 0.009 | 0.006 | EGFR/EPHA2/FGFR2 | 3 |
| GO:0008288 | boss receptor activity | 3/84 | 49/18639 | 0.0612244897959184 | 13.58527696793 | 5.93512595752481 | 0.001 | 0.009 | 0.006 | EGFR/EPHA2/FGFR2 | 3 |
| GO:0036332 | placental growth factor receptor activity | 3/84 | 49/18639 | 0.0612244897959184 | 13.58527696793 | 5.93512595752481 | 0.001 | 0.009 | 0.006 | EGFR/EPHA2/FGFR2 | 3 |
| GO:0038062 | protein tyrosine kinase collagen receptor activity | 3/84 | 49/18639 | 0.0612244897959184 | 13.58527696793 | 5.93512595752481 | 0.001 | 0.009 | 0.006 | EGFR/EPHA2/FGFR2 | 3 |
| GO:0060175 | brain-derived neurotrophic factor receptor activity | 3/84 | 49/18639 | 0.0612244897959184 | 13.58527696793 | 5.93512595752481 | 0.001 | 0.009 | 0.006 | EGFR/EPHA2/FGFR2 | 3 |
| GO:0005006 | epidermal growth factor receptor activity | 3/84 | 50/18639 | 0.06 | 13.3135714285714 | 5.86610510114832 | 0.001 | 0.009 | 0.006 | EGFR/EPHA2/FGFR2 | 3 |
| GO:0005007 | fibroblast growth factor receptor activity | 3/84 | 50/18639 | 0.06 | 13.3135714285714 | 5.86610510114832 | 0.001 | 0.009 | 0.006 | EGFR/EPHA2/FGFR2 | 3 |
| GO:0005017 | platelet-derived growth factor receptor activity | 3/84 | 50/18639 | 0.06 | 13.3135714285714 | 5.86610510114832 | 0.001 | 0.009 | 0.006 | EGFR/EPHA2/FGFR2 | 3 |
| GO:0005019 | platelet-derived growth factor beta-receptor activity | 3/84 | 50/18639 | 0.06 | 13.3135714285714 | 5.86610510114832 | 0.001 | 0.009 | 0.006 | EGFR/EPHA2/FGFR2 | 3 |
| GO:0005010 | insulin-like growth factor receptor activity | 3/84 | 51/18639 | 0.0588235294117647 | 13.0525210084034 | 5.79903153842849 | 0.002 | 0.009 | 0.006 | EGFR/EPHA2/FGFR2 | 3 |
| GO:0005021 | vascular endothelial growth factor receptor activity | 3/84 | 51/18639 | 0.0588235294117647 | 13.0525210084034 | 5.79903153842849 | 0.002 | 0.009 | 0.006 | EGFR/EPHA2/FGFR2 | 3 |
| GO:0098632 | cell-cell adhesion mediator activity | 3/84 | 55/18639 | 0.0545454545454545 | 12.1032467532468 | 5.54843567173338 | 0.002 | 0.011 | 0.007 | BAIAP2/CNTN5/KRT18 | 3 |
| GO:0004175 | endopeptidase activity | 7/84 | 405/18639 | 0.017283950617284 | 3.83518518518518 | 3.88129617833021 | 0.002 | 0.013 | 0.008 | ACE2/CTSL/MMP12/MMP7/PIK3IP1/PRSS8/REN | 7 |
| GO:0001786 | phosphatidylserine binding | 3/84 | 59/18639 | 0.0508474576271187 | 11.2826876513317 | 5.32253605670083 | 0.002 | 0.013 | 0.008 | CD300A/HAVCR1/SCARB2 | 3 |
| GO:0045236 | CXCR chemokine receptor binding | 2/84 | 17/18639 | 0.117647058823529 | 26.1050420168067 | 6.96756565091787 | 0.003 | 0.013 | 0.009 | CXCL9/TFF2 | 2 |
| GO:0070492 | oligosaccharide binding | 2/84 | 17/18639 | 0.117647058823529 | 26.1050420168067 | 6.96756565091787 | 0.003 | 0.013 | 0.009 | LGALS9/REG1B | 2 |
| GO:0004857 | enzyme inhibitor activity | 7/84 | 416/18639 | 0.0168269230769231 | 3.73377403846154 | 3.79409464217176 | 0.003 | 0.014 | 0.009 | AMBP/BST2/COL6A3/CST3/LILRB4/SPINK1/WFDC2 | 7 |
| GO:0098641 | cadherin binding involved in cell-cell adhesion | 2/84 | 18/18639 | 0.111111111111111 | 24.6547619047619 | 6.75557237236273 | 0.003 | 0.014 | 0.010 | BAIAP2/KRT18 | 2 |
| GO:0038024 | cargo receptor activity | 3/84 | 65/18639 | 0.0461538461538462 | 10.2412087912088 | 5.0215935485656 | 0.003 | 0.015 | 0.010 | ASGR1/COLEC12/SCARB2 | 3 |
| GO:0016500 | protein-hormone receptor activity | 3/84 | 67/18639 | 0.0447761194029851 | 9.93550106609808 | 4.92987369000525 | 0.003 | 0.016 | 0.011 | EGFR/EPHA2/FGFR2 | 3 |
| GO:0004713 | protein tyrosine kinase activity | 4/84 | 140/18639 | 0.0285714285714286 | 6.33979591836735 | 4.26700712537449 | 0.004 | 0.017 | 0.011 | EFNA4/EGFR/EPHA2/FGFR2 | 4 |
| GO:0005518 | collagen binding | 3/84 | 72/18639 | 0.0416666666666667 | 9.24553571428571 | 4.71653513516857 | 0.004 | 0.019 | 0.012 | CTSL/ITGA11/MMP12 | 3 |
| GO:0042379 | chemokine receptor binding | 3/84 | 72/18639 | 0.0416666666666667 | 9.24553571428571 | 4.71653513516857 | 0.004 | 0.019 | 0.012 | CCL11/CXCL9/TFF2 | 3 |
| GO:0019865 | immunoglobulin binding | 2/84 | 26/18639 | 0.0769230769230769 | 17.0686813186813 | 5.51654976620797 | 0.006 | 0.026 | 0.017 | AMBP/PIGR | 2 |
| GO:0008083 | growth factor activity | 4/84 | 162/18639 | 0.0246913580246914 | 5.47883597883598 | 3.8522614397335 | 0.006 | 0.026 | 0.017 | AREG/CSF1/GDF15/INHBB | 4 |
| GO:0005044 | scavenger receptor activity | 2/84 | 27/18639 | 0.0740740740740741 | 16.4365079365079 | 5.40061524569451 | 0.007 | 0.027 | 0.018 | COLEC12/SCARB2 | 2 |
| GO:0072341 | modified amino acid binding | 3/84 | 91/18639 | 0.032967032967033 | 7.31514913657771 | 4.06316614398236 | 0.008 | 0.033 | 0.022 | CD300A/HAVCR1/SCARB2 | 3 |
| GO:0043178 | alcohol binding | 3/84 | 92/18639 | 0.0326086956521739 | 7.23563664596273 | 4.0341003358175 | 0.008 | 0.034 | 0.022 | NPC2/RBP5/SCARB2 | 3 |
| GO:0019207 | kinase regulator activity | 5/84 | 287/18639 | 0.0174216027874564 | 3.86572921851667 | 3.29187410164749 | 0.010 | 0.038 | 0.025 | AREG/EGFR/GPRC5C/LILRB4/PIK3IP1 | 5 |
| GO:0030296 | protein tyrosine kinase activator activity | 2/84 | 33/18639 | 0.0606060606060606 | 13.448051948052 | 4.81549205938829 | 0.010 | 0.038 | 0.025 | AREG/EGFR | 2 |
| GO:0004869 | cysteine-type endopeptidase inhibitor activity | 2/84 | 36/18639 | 0.0555555555555556 | 12.327380952381 | 4.57718094514525 | 0.011 | 0.044 | 0.029 | CST3/WFDC2 | 2 |

**Supplementary Table S10. Reactome pathway enrichment analysis**

Reactome enrichment of the 85 candidate mediator proteins. In total, 10 pathways met FDR < 0.05.

| **ID** | **Description** | **Gene ratio** | **Background ratio** | **Rich factor** | **Fold enrichment** | **z score** | **P value** | **Adjusted P** | **q value** | **Gene IDs** | **Count** |
| --- | --- | --- | --- | --- | --- | --- | --- | --- | --- | --- | --- |
| R-HSA-5669034 | TNFs bind their physiological receptors | 7/72 | 29/11214 | 0.241379310344828 | 37.5948275862069 | 15.8615743879157 | 4.655 × 10⁻¹⁰ | 1.261 × 10⁻⁷ | 1.102 × 10⁻⁷ | CD27/EDA2R/TNFRSF14/TNFRSF1A/TNFRSF1B/TNFRSF4/TNFRSF9 | 7 |
| R-HSA-5668541 | TNFR2 non-canonical NF-kB pathway | 8/72 | 89/11214 | 0.0898876404494382 | 14 | 9.89769183778069 | 9.045 × 10⁻⁸ | 1.226 × 10⁻⁵ | 1.071 × 10⁻⁵ | CD27/EDA2R/TNFRSF11A/TNFRSF14/TNFRSF1A/TNFRSF1B/TNFRSF4/TNFRSF9 | 8 |
| R-HSA-198933 | Immunoregulatory interactions between a Lymphoid and a non-Lymphoid cell | 8/72 | 133/11214 | 0.0601503759398496 | 9.36842105263158 | 7.80414713319359 | 2.005 × 10⁻⁶ | 1.811 × 10⁻⁴ | 1.583 × 10⁻⁴ | CD300A/COLEC12/LILRB4/NCR3LG1/NECTIN2/NPDC1/PILRA/SIGLEC1 | 8 |
| R-HSA-8957275 | Post-translational protein phosphorylation | 6/72 | 108/11214 | 0.0555555555555556 | 8.65277777777778 | 6.42388455919935 | 6.474 × 10⁻⁵ | 0.004 | 0.004 | CKAP4/CSF1/CST3/FSTL3/IGFBP4/SHISA5 | 6 |
| R-HSA-381426 | Regulation of Insulin-like Growth Factor (IGF) transport and uptake by Insulin-like Growth Factor Binding Proteins (IGFBPs) | 6/72 | 125/11214 | 0.048 | 7.476 | 5.85276702672642 | 1.457 × 10⁻⁴ | 0.008 | 0.007 | CKAP4/CSF1/CST3/FSTL3/IGFBP4/SHISA5 | 6 |
| R-HSA-6783783 | Interleukin-10 signaling | 4/72 | 47/11214 | 0.0851063829787234 | 13.2553191489362 | 6.7678524000716 | 2.262 × 10⁻⁴ | 0.009 | 0.008 | CD80/CSF1/TNFRSF1A/TNFRSF1B | 4 |
| R-HSA-9694614 | Attachment and Entry | 3/72 | 19/11214 | 0.157894736842105 | 24.5921052631579 | 8.27326682200159 | 2.284 × 10⁻⁴ | 0.009 | 0.008 | ACE2/CTSL/HAVCR1 | 3 |
| R-HSA-449147 | Signaling by Interleukins | 10/72 | 460/11214 | 0.0217391304347826 | 3.38586956521739 | 4.20035676181458 | 6.687 × 10⁻⁴ | 0.022 | 0.020 | CCL11/CD80/CSF1/HAVCR2/IL18BP/IL2RA/IL4R/LGALS9/TNFRSF1A/TNFRSF1B | 10 |
| R-HSA-1442490 | Collagen degradation | 4/72 | 64/11214 | 0.0625 | 9.734375 | 5.63287312692871 | 7.422 × 10⁻⁴ | 0.022 | 0.020 | COL6A3/CTSL/MMP12/MMP7 | 4 |
| R-HSA-9772572 | Early SARS-CoV-2 Infection Events | 3/72 | 38/11214 | 0.0789473684210526 | 12.2960526315789 | 5.60687522182295 | 0.002 | 0.049 | 0.043 | ACE2/CTSL/HAVCR1 | 3 |

**Supplementary Table S11. KEGG pathway enrichment analysis**

KEGG enrichment of the 85 candidate mediator proteins. Four pathways met FDR < 0.05.

| **ID** | **Description** | **Gene ratio** | **Background ratio** | **Rich factor** | **Fold enrichment** | **z score** | **P value** | **Adjusted P** | **q value** | **Gene IDs** | **Count** |
| --- | --- | --- | --- | --- | --- | --- | --- | --- | --- | --- | --- |
| hsa04060 | Cytokine-cytokine receptor interaction | 16/58 | 298/9424 | 0.0536912751677852 | 8.72390650312428 | 10.6619446471331 | 1.615 × 10⁻¹¹ | 2.293 × 10⁻⁹ | 2.091 × 10⁻⁹ | 6356/939/1435/4283/60401/9518/3559/3566/3625/8797/8792/8764/7132/7133/7293/3604 | 16 |
| hsa04061 | Viral protein interaction with cytokine and cytokine receptor | 8/58 | 100/9424 | 0.08 | 12.9986206896552 | 9.49208694237611 | 1.499 × 10⁻⁷ | 1.065 × 10⁻⁵ | 9.706 × 10⁻⁶ | 6356/1435/4283/3559/8797/8764/7132/7133 | 8 |
| hsa04151 | PI3K-Akt signaling pathway | 10/58 | 362/9424 | 0.0276243093922652 | 4.48847399504668 | 5.326099332434 | 6.205 × 10⁻⁵ | 0.003 | 0.003 | 374/1293/1435/1945/1956/1969/2263/3559/3566/22801 | 10 |
| hsa05168 | Herpes simplex virus 1 infection | 6/58 | 180/9424 | 0.0333333333333333 | 5.41609195402299 | 4.70734368715565 | 7.934 × 10⁻⁴ | 0.028 | 0.026 | 684/972/5819/29992/8764/7132 | 6 |

**Supplementary Table S12. Candidate mediator proteins – complete list (85 proteins, PM >5%)**

All 85 proteins with an estimated proportion mediated >5% and FDR <0.05 for the indirect effect are shown. Each protein was analysed using participants with complete Model 2 covariates and an available measurement for that protein; missing protein measurements were not imputed. Consequently, sample size and event count vary across proteins. The protein–epilepsy association column describes the direction of the protein coefficient in the epilepsy model; it does not denote a protective or harmful causal pathway. Proportions mediated should not be summed because proteins are correlated and were assessed separately.

| **Protein** | **N** | **Events** | **Protein–epilepsy association** | **β (PPI→protein)** | **HR (protein→epilepsy)** | **Indirect HR (95% CI)** | **PM (%)** | **FDR indirect** |
| --- | --- | --- | --- | --- | --- | --- | --- | --- |
| GDF15 | 51,179 | 413 | Positively associated with epilepsy | 0.4270476 | 1.4069185 | 1.157 (1.118–1.197) | 21.07 | <0.001 |
| NEFL | 49,217 | 398 | Positively associated with epilepsy | 0.1441424 | 1.5258659 | 1.063 (1.046–1.080) | 8.75 | <0.001 |
| WFDC2 | 47,989 | 384 | Positively associated with epilepsy | 0.2562183 | 1.3607042 | 1.082 (1.056–1.109) | 10.93 | <0.001 |
| CKAP4 | 50,863 | 412 | Positively associated with epilepsy | 0.1996844 | 1.2901526 | 1.052 (1.035–1.070) | 7.17 | <0.001 |
| VSIG4 | 50,499 | 401 | Positively associated with epilepsy | 0.2363852 | 1.3461045 | 1.073 (1.047–1.099) | 10.30 | <0.001 |
| CD302 | 50,329 | 411 | Positively associated with epilepsy | 0.2464249 | 1.3185315 | 1.071 (1.046–1.096) | 9.79 | <0.001 |
| EPHA2 | 48,733 | 400 | Positively associated with epilepsy | 0.2207240 | 1.2839277 | 1.057 (1.037–1.077) | 7.92 | <0.001 |
| AREG | 50,329 | 411 | Positively associated with epilepsy | 0.1885279 | 1.3038066 | 1.051 (1.033–1.070) | 7.19 | <0.001 |
| SHISA5 | 43,951 | 351 | Positively associated with epilepsy | 0.2222681 | 1.2401770 | 1.049 (1.031–1.067) | 7.87 | <0.001 |
| SPINK1 | 50,569 | 402 | Positively associated with epilepsy | 0.1658198 | 1.3058971 | 1.045 (1.029–1.062) | 6.52 | <0.001 |
| EDA2R | 50,987 | 416 | Positively associated with epilepsy | 0.1710545 | 1.3748580 | 1.056 (1.035–1.077) | 7.76 | <0.001 |
| PIGR | 49,914 | 394 | Positively associated with epilepsy | 0.1642814 | 1.3498409 | 1.051 (1.032–1.070) | 6.96 | <0.001 |
| CTSL | 50,557 | 410 | Positively associated with epilepsy | 0.2418580 | 1.2999795 | 1.066 (1.041–1.091) | 9.45 | <0.001 |
| ACE2 | 49,637 | 403 | Positively associated with epilepsy | 0.2819593 | 1.2891580 | 1.074 (1.046–1.103) | 10.17 | <0.001 |
| TNFRSF1A | 50,569 | 402 | Positively associated with epilepsy | 0.1844409 | 1.3034898 | 1.050 (1.031–1.070) | 7.21 | <0.001 |
| PLAUR | 50,194 | 409 | Positively associated with epilepsy | 0.2257934 | 1.3197748 | 1.065 (1.040–1.090) | 8.71 | <0.001 |
| TNFRSF1B | 50,569 | 402 | Positively associated with epilepsy | 0.2287175 | 1.1955523 | 1.042 (1.026–1.058) | 6.02 | <0.001 |
| IGFBP4 | 49,917 | 397 | Positively associated with epilepsy | 0.2342569 | 1.2847952 | 1.060 (1.037–1.084) | 8.80 | <0.001 |
| LILRB4 | 49,616 | 400 | Positively associated with epilepsy | 0.1915320 | 1.3152065 | 1.054 (1.033–1.075) | 7.69 | <0.001 |
| GGT1 | 50,487 | 402 | Positively associated with epilepsy | 0.2322567 | 1.2906872 | 1.061 (1.037–1.086) | 8.74 | <0.001 |
| TNFRSF10A | 50,160 | 407 | Positively associated with epilepsy | 0.2260085 | 1.2463823 | 1.051 (1.031–1.072) | 7.58 | <0.001 |
| EFNA4 | 50,076 | 407 | Positively associated with epilepsy | 0.1903965 | 1.2586501 | 1.045 (1.026–1.063) | 6.67 | <0.001 |
| CD27 | 50,712 | 411 | Positively associated with epilepsy | 0.1724063 | 1.2726522 | 1.042 (1.025–1.060) | 5.86 | <0.001 |
| RBP5 | 50,329 | 411 | Positively associated with epilepsy | 0.2057840 | 1.2846909 | 1.053 (1.031–1.075) | 7.41 | <0.001 |
| IL4R | 50,488 | 408 | Positively associated with epilepsy | 0.1864833 | 1.2554901 | 1.043 (1.025–1.062) | 6.04 | <0.001 |
| HAVCR1 | 50,913 | 416 | Positively associated with epilepsy | 0.1857327 | 1.2906769 | 1.049 (1.028–1.069) | 6.77 | <0.001 |
| CHI3L1 | 49,446 | 409 | Positively associated with epilepsy | 0.1734789 | 1.2736318 | 1.043 (1.025–1.061) | 5.81 | <0.001 |
| PIK3IP1 | 50,569 | 402 | Positively associated with epilepsy | 0.1524004 | 1.2708941 | 1.037 (1.022–1.053) | 5.39 | <0.001 |
| PRSS8 | 50,359 | 409 | Positively associated with epilepsy | 0.1873661 | 1.3138084 | 1.052 (1.030–1.075) | 7.10 | <0.001 |
| SCARB2 | 49,204 | 403 | Positively associated with epilepsy | 0.1912817 | 1.2606974 | 1.045 (1.026–1.065) | 6.84 | <0.001 |
| BST2 | 50,996 | 413 | Positively associated with epilepsy | 0.2366357 | 1.2208047 | 1.048 (1.028–1.069) | 6.88 | <0.001 |
| COL6A3 | 51,179 | 413 | Positively associated with epilepsy | 0.1957042 | 1.2714708 | 1.048 (1.028–1.069) | 6.79 | <0.001 |
| ASGR1 | 50,160 | 407 | Positively associated with epilepsy | 0.2703111 | 1.2673654 | 1.066 (1.038–1.095) | 9.75 | <0.001 |
| OCLN | 43,873 | 349 | Positively associated with epilepsy | 0.2086889 | 1.2765651 | 1.052 (1.030–1.075) | 8.03 | <0.001 |
| RNASE1 | 43,984 | 348 | Positively associated with epilepsy | 0.1898089 | 1.2275674 | 1.040 (1.023–1.057) | 6.48 | <0.001 |
| NECTIN2 | 50,483 | 409 | Positively associated with epilepsy | 0.1866559 | 1.2515474 | 1.043 (1.024–1.062) | 6.22 | <0.001 |
| RNF149 | 42,783 | 343 | Positively associated with epilepsy | 0.1840840 | 1.2401602 | 1.040 (1.023–1.058) | 6.16 | <0.001 |
| LGALS9 | 50,524 | 411 | Positively associated with epilepsy | 0.2869874 | 1.2751299 | 1.072 (1.040–1.105) | 9.79 | <0.001 |
| PALM2 | 43,853 | 348 | Positively associated with epilepsy | 0.2349592 | 1.2648671 | 1.057 (1.032–1.082) | 8.73 | <0.001 |
| SIGLEC1 | 50,774 | 413 | Positively associated with epilepsy | 0.1970872 | 1.2637480 | 1.047 (1.026–1.069) | 6.52 | <0.001 |
| HAVCR2 | 50,569 | 402 | Positively associated with epilepsy | 0.2513028 | 1.2568746 | 1.059 (1.033–1.086) | 8.47 | <0.001 |
| GPR37 | 49,637 | 403 | Positively associated with epilepsy | 0.1587857 | 1.2839497 | 1.040 (1.023–1.059) | 5.63 | <0.001 |
| IL18BP | 50,760 | 415 | Positively associated with epilepsy | 0.1995378 | 1.2474075 | 1.045 (1.025–1.066) | 6.17 | <0.001 |
| RNASE6 | 43,672 | 346 | Positively associated with epilepsy | 0.2669042 | 1.1815485 | 1.046 (1.025–1.066) | 7.30 | <0.001 |
| LAYN | 50,076 | 407 | Positively associated with epilepsy | 0.1593346 | 1.2511047 | 1.036 (1.020–1.053) | 5.43 | <0.001 |
| AMBP | 50,913 | 416 | Positively associated with epilepsy | 0.1714008 | 1.2708873 | 1.042 (1.023–1.061) | 5.87 | <0.001 |
| SPON2 | 50,315 | 408 | Positively associated with epilepsy | 0.1848858 | 1.2554237 | 1.043 (1.023–1.063) | 6.22 | <0.001 |
| NBL1 | 50,712 | 411 | Positively associated with epilepsy | 0.1687327 | 1.2488542 | 1.038 (1.021–1.056) | 5.29 | <0.001 |
| TNFRSF14 | 50,609 | 413 | Positively associated with epilepsy | 0.1862696 | 1.2379241 | 1.041 (1.022–1.059) | 5.62 | <0.001 |
| GFRA1 | 50,910 | 416 | Positively associated with epilepsy | 0.2124328 | 1.2515319 | 1.049 (1.026–1.072) | 6.80 | <0.001 |
| COLEC12 | 50,526 | 413 | Positively associated with epilepsy | 0.1531017 | 1.2679081 | 1.037 (1.020–1.054) | 5.13 | <0.001 |
| CD74 | 50,996 | 413 | Positively associated with epilepsy | 0.1974791 | 1.2493841 | 1.045 (1.024–1.066) | 6.41 | <0.001 |
| IL2RA | 50,196 | 406 | Positively associated with epilepsy | 0.1939041 | 1.2325663 | 1.041 (1.022–1.061) | 5.83 | <0.001 |
| LGALS4 | 50,526 | 413 | Positively associated with epilepsy | 0.1838908 | 1.2419596 | 1.041 (1.022–1.060) | 5.63 | <0.001 |
| FSTL3 | 50,446 | 411 | Positively associated with epilepsy | 0.2141641 | 1.2504849 | 1.049 (1.026–1.073) | 6.87 | <0.001 |
| CSF1 | 50,774 | 413 | Positively associated with epilepsy | 0.1943632 | 1.2411995 | 1.043 (1.023–1.064) | 5.94 | <0.001 |
| BAIAP2 | 49,375 | 399 | Positively associated with epilepsy | 0.1936344 | 1.2301600 | 1.041 (1.021–1.061) | 5.50 | <0.001 |
| NCR3LG1 | 43,769 | 349 | Positively associated with epilepsy | 0.1733952 | 1.2599127 | 1.041 (1.021–1.061) | 6.55 | <0.001 |
| NPC2 | 43,213 | 345 | Positively associated with epilepsy | 0.2508746 | 1.2214874 | 1.051 (1.027–1.077) | 8.71 | <0.001 |
| EGFR | 51,179 | 413 | Inversely associated with epilepsy | -0.2578896 | 0.8149682 | 1.054 (1.028–1.081) | 7.62 | <0.001 |
| CNTN5 | 50,672 | 412 | Inversely associated with epilepsy | -0.1862962 | 0.7916888 | 1.044 (1.023–1.067) | 6.32 | <0.001 |
| CD300A | 42,991 | 341 | Positively associated with epilepsy | 0.1857035 | 1.2573706 | 1.043 (1.022–1.065) | 7.53 | <0.001 |
| ADM | 50,987 | 416 | Positively associated with epilepsy | 0.1885449 | 1.2956094 | 1.050 (1.026–1.075) | 6.96 | <0.001 |
| CST3 | 50,676 | 414 | Positively associated with epilepsy | 0.1929272 | 1.2373620 | 1.042 (1.021–1.063) | 5.73 | <0.001 |
| TNFRSF11A | 51,111 | 414 | Positively associated with epilepsy | 0.1729571 | 1.2295834 | 1.036 (1.019–1.055) | 5.07 | <0.001 |
| PILRA | 50,569 | 402 | Positively associated with epilepsy | 0.1483151 | 1.2574776 | 1.035 (1.018–1.052) | 5.01 | <0.001 |
| TNFRSF4 | 51,028 | 414 | Positively associated with epilepsy | 0.2487661 | 1.2109259 | 1.049 (1.024–1.074) | 6.75 | <0.001 |
| GPRC5C | 43,748 | 350 | Positively associated with epilepsy | 0.2397413 | 1.1829544 | 1.041 (1.020–1.063) | 6.42 | <0.001 |
| NPDC1 | 50,557 | 410 | Positively associated with epilepsy | 0.1997335 | 1.1973327 | 1.037 (1.018–1.056) | 5.36 | <0.001 |
| TNFRSF9 | 50,160 | 407 | Positively associated with epilepsy | 0.2037227 | 1.1885275 | 1.036 (1.017–1.055) | 5.35 | <0.001 |
| MMP12 | 50,590 | 415 | Positively associated with epilepsy | 0.1779313 | 1.2298238 | 1.037 (1.018–1.057) | 5.21 | <0.001 |
| REN | 50,557 | 410 | Positively associated with epilepsy | 0.2229728 | 1.1982154 | 1.041 (1.020–1.063) | 6.01 | <0.001 |
| MMP7 | 49,561 | 402 | Positively associated with epilepsy | 0.1685969 | 1.2338610 | 1.036 (1.017–1.056) | 5.13 | <0.001 |
| ITGA11 | 50,488 | 408 | Inversely associated with epilepsy | -0.2523711 | 0.8271146 | 1.049 (1.022–1.076) | 6.82 | <0.001 |
| CD80 | 43,947 | 351 | Positively associated with epilepsy | 0.1938771 | 1.2103282 | 1.038 (1.017–1.059) | 5.90 | <0.001 |
| FGFR2 | 50,712 | 411 | Positively associated with epilepsy | 0.2251276 | 1.2034307 | 1.043 (1.019–1.067) | 5.88 | <0.001 |
| REG1B | 51,179 | 413 | Positively associated with epilepsy | 0.2127377 | 1.1786630 | 1.036 (1.016–1.056) | 5.05 | <0.001 |
| CXCL9 | 50,774 | 413 | Positively associated with epilepsy | 0.1980483 | 1.1891123 | 1.035 (1.015–1.055) | 4.85 | <0.001 |
| TFF2 | 50,690 | 413 | Positively associated with epilepsy | 0.5027680 | 1.1865934 | 1.090 (1.037–1.145) | 12.14 | <0.001 |
| INHBB | 42,991 | 341 | Positively associated with epilepsy | 0.2005708 | 1.2101431 | 1.039 (1.016–1.062) | 6.77 | <0.001 |
| CCL11 | 51,111 | 414 | Positively associated with epilepsy | 0.2014028 | 1.1946318 | 1.036 (1.014–1.059) | 5.08 | 0.001 |
| IFI30 | 42,713 | 339 | Positively associated with epilepsy | 0.2807266 | 1.1928504 | 1.051 (1.019–1.084) | 8.07 | 0.002 |
| KRT18 | 50,329 | 411 | Positively associated with epilepsy | 0.2091084 | 1.1759164 | 1.034 (1.013–1.057) | 4.87 | 0.002 |
| PLA2G15 | 50,712 | 411 | Positively associated with epilepsy | 0.2560900 | 1.1584740 | 1.038 (1.013–1.065) | 5.31 | 0.003 |
| FUOM | 42,991 | 341 | Positively associated with epilepsy | 0.2324077 | 1.1746054 | 1.038 (1.012–1.065) | 6.62 | 0.004 |

**Supplementary Table S13. Proteins associated with incident epilepsy – Cox regression**

**Proteins associated with incident epilepsy at FDR <0.05 in the proteomic sub-cohort are shown. Each Cox model used participants with complete Model 2 covariates and an available measurement for the protein being analysed; missing protein measurements were not imputed. Model 2 included age, sex, body mass index, Townsend deprivation index, smoking status, alcohol intake, ethnicity, and education. Protein-specific sample sizes and event counts therefore vary across rows. The corresponding protein coefficient from these same models was used as the protein–epilepsy (b-path) estimate in Supplementary Table S12. Rows are sorted by FDR.**

| **Protein** | **N** | **Events** | **β** | **HR (95% CI)** | **FDR** |
| --- | --- | --- | --- | --- | --- |
| nefl | 49,217 | 398 | 0.4225620 | 1.526 (1.406–1.656) | 1.09 × 10⁻²⁰ |
| gdf15 | 51,179 | 413 | 0.3414019 | 1.407 (1.301–1.522) | 1.93 × 10⁻¹⁴ |
| ckap4 | 50,863 | 412 | 0.2547605 | 1.29 (1.195–1.393) | 4.62 × 10⁻⁸ |
| wfdc2 | 47,989 | 384 | 0.3080024 | 1.361 (1.241–1.492) | 4.62 × 10⁻⁸ |
| spink1 | 50,569 | 402 | 0.2668902 | 1.306 (1.201–1.42) | 2.28 × 10⁻⁷ |
| areg | 50,329 | 411 | 0.2652881 | 1.304 (1.199–1.418) | 2.88 × 10⁻⁷ |
| epha2 | 48,733 | 400 | 0.2499239 | 1.284 (1.184–1.392) | 5.1 × 10⁻⁷ |
| vsig4 | 50,499 | 401 | 0.2972149 | 1.346 (1.223–1.482) | 5.1 × 10⁻⁷ |
| cd302 | 50,329 | 411 | 0.2765186 | 1.319 (1.205–1.443) | 5.45 × 10⁻⁷ |
| shisa5 | 43,951 | 351 | 0.2152542 | 1.24 (1.155–1.331) | 8.07 × 10⁻⁷ |
| angpt2 | 50,987 | 416 | 0.2805815 | 1.324 (1.206–1.453) | 8.3 × 10⁻⁷ |
| pigr | 49,914 | 394 | 0.2999867 | 1.35 (1.222–1.491) | 8.3 × 10⁻⁷ |
| eda2r | 50,987 | 416 | 0.3183505 | 1.375 (1.234–1.531) | 1.55 × 10⁻⁶ |
| gfap | 49,295 | 399 | 0.2737629 | 1.315 (1.196–1.445) | 2.97 × 10⁻⁶ |
| ctsl | 50,557 | 410 | 0.2623485 | 1.3 (1.186–1.425) | 3.9 × 10⁻⁶ |
| igfbp7 | 51,179 | 413 | 0.2147663 | 1.24 (1.15–1.336) | 3.9 × 10⁻⁶ |
| tnfrsf1a | 50,569 | 402 | 0.2650452 | 1.303 (1.188–1.43) | 3.9 × 10⁻⁶ |
| lilrb4 | 49,616 | 400 | 0.2739937 | 1.315 (1.192–1.451) | 7.65 × 10⁻⁶ |
| plaur | 50,194 | 409 | 0.2774611 | 1.32 (1.194–1.459) | 8.04 × 10⁻⁶ |
| tnfrsf1b | 50,569 | 402 | 0.1786083 | 1.196 (1.121–1.275) | 8.04 × 10⁻⁶ |
| ace2 | 49,637 | 403 | 0.2539893 | 1.289 (1.176–1.413) | 8.9 × 10⁻⁶ |
| igfbp4 | 49,917 | 397 | 0.2505993 | 1.285 (1.173–1.407) | 9.48 × 10⁻⁶ |
| bmper | 42,991 | 341 | 0.2115697 | 1.236 (1.144–1.335) | 9.55 × 10⁻⁶ |
| ggt1 | 50,487 | 402 | 0.2551748 | 1.291 (1.174–1.419) | 1.63 × 10⁻⁵ |
| cd27 | 50,712 | 411 | 0.2411030 | 1.273 (1.163–1.393) | 1.85 × 10⁻⁵ |
| pik3ip1 | 50,569 | 402 | 0.2397207 | 1.271 (1.162–1.391) | 1.99 × 10⁻⁵ |
| ltbr | 50,278 | 411 | 0.2332107 | 1.263 (1.156–1.379) | 2.22 × 10⁻⁵ |
| tnfrsf10a | 50,160 | 407 | 0.2202452 | 1.246 (1.147–1.355) | 2.35 × 10⁻⁵ |
| il4r | 50,488 | 408 | 0.2275260 | 1.255 (1.151–1.369) | 2.63 × 10⁻⁵ |
| efna4 | 50,076 | 407 | 0.2300398 | 1.259 (1.153–1.374) | 2.63 × 10⁻⁵ |
| chi3l1 | 49,446 | 409 | 0.2418725 | 1.274 (1.161–1.397) | 2.69 × 10⁻⁵ |
| cxadr | 50,488 | 408 | 0.2243085 | 1.251 (1.148–1.365) | 3.4 × 10⁻⁵ |
| havcr1 | 50,913 | 416 | 0.2551668 | 1.291 (1.17–1.424) | 3.46 × 10⁻⁵ |
| rbp5 | 50,329 | 411 | 0.2505181 | 1.285 (1.166–1.416) | 3.73 × 10⁻⁵ |
| scarb2 | 49,204 | 403 | 0.2316650 | 1.261 (1.151–1.381) | 4.8 × 10⁻⁵ |
| prss8 | 50,359 | 409 | 0.2729301 | 1.314 (1.18–1.463) | 4.93 × 10⁻⁵ |
| col6a3 | 51,179 | 413 | 0.2401743 | 1.271 (1.156–1.399) | 6.71 × 10⁻⁵ |
| bst2 | 50,996 | 413 | 0.1995102 | 1.221 (1.127–1.322) | 6.9 × 10⁻⁵ |
| rnf149 | 42,783 | 343 | 0.2152406 | 1.24 (1.138–1.352) | 7.84 × 10⁻⁵ |
| rnase1 | 43,984 | 348 | 0.2050345 | 1.228 (1.13–1.333) | 7.87 × 10⁻⁵ |
| gpr37 | 49,637 | 403 | 0.2499410 | 1.284 (1.161–1.42) | 8.27 × 10⁻⁵ |
| nectin2 | 50,483 | 409 | 0.2243807 | 1.252 (1.143–1.37) | 8.6 × 10⁻⁵ |
| ocln | 43,873 | 349 | 0.2441729 | 1.277 (1.156–1.409) | 8.85 × 10⁻⁵ |
| asgr1 | 50,160 | 407 | 0.2369403 | 1.267 (1.15–1.396) | 1.12 × 10⁻⁴ |
| layn | 50,076 | 407 | 0.2240270 | 1.251 (1.141–1.371) | 1.12 × 10⁻⁴ |
| klrb1 | 51,111 | 414 | 0.2302252 | 1.259 (1.145–1.384) | 1.19 × 10⁻⁴ |
| pgf | 51,111 | 414 | 0.2065199 | 1.229 (1.129–1.339) | 1.38 × 10⁻⁴ |
| siglec1 | 50,774 | 413 | 0.2340819 | 1.264 (1.147–1.393) | 1.38 × 10⁻⁴ |
| ca12 | 50,329 | 411 | 0.2049160 | 1.227 (1.127–1.336) | 1.39 × 10⁻⁴ |
| palm2 | 43,853 | 348 | 0.2349670 | 1.265 (1.147–1.395) | 1.58 × 10⁻⁴ |
| igfbp2 | 51,099 | 413 | 0.2727972 | 1.314 (1.172–1.473) | 1.65 × 10⁻⁴ |
| il18bp | 50,760 | 415 | 0.2210674 | 1.247 (1.137–1.369) | 1.65 × 10⁻⁴ |
| ambp | 50,913 | 416 | 0.2397153 | 1.271 (1.149–1.405) | 1.65 × 10⁻⁴ |
| fam3c | 50,483 | 409 | 0.2281961 | 1.256 (1.142–1.383) | 1.65 × 10⁻⁴ |
| lrg1 | 44,019 | 347 | 0.2431622 | 1.275 (1.151–1.413) | 1.75 × 10⁻⁴ |
| fstl1 | 42,783 | 343 | 0.2263065 | 1.254 (1.14–1.38) | 1.84 × 10⁻⁴ |
| nbl1 | 50,712 | 411 | 0.2222265 | 1.249 (1.137–1.372) | 1.9 × 10⁻⁴ |
| havcr2 | 50,569 | 402 | 0.2286282 | 1.257 (1.141–1.385) | 1.92 × 10⁻⁴ |
| ckb | 43,951 | 351 | 0.2791007 | 1.322 (1.174–1.488) | 1.93 × 10⁻⁴ |
| lgals9 | 50,524 | 411 | 0.2430481 | 1.275 (1.15–1.414) | 1.93 × 10⁻⁴ |
| spon2 | 50,315 | 408 | 0.2274731 | 1.255 (1.14–1.383) | 1.97 × 10⁻⁴ |
| igfbpl1 | 50,557 | 410 | 0.2239276 | 1.251 (1.137–1.377) | 2.12 × 10⁻⁴ |
| rnase6 | 43,672 | 346 | 0.1668259 | 1.182 (1.1–1.269) | 2.12 × 10⁻⁴ |
| acvrl1 | 50,076 | 407 | 0.2247490 | 1.252 (1.137–1.379) | 2.29 × 10⁻⁴ |
| colec12 | 50,526 | 413 | 0.2373683 | 1.268 (1.145–1.404) | 2.29 × 10⁻⁴ |
| tnfrsf14 | 50,609 | 413 | 0.2134358 | 1.238 (1.129–1.357) | 2.29 × 10⁻⁴ |
| cd274 | 50,160 | 407 | 0.1977849 | 1.219 (1.118–1.328) | 2.75 × 10⁻⁴ |
| dsc2 | 49,999 | 396 | 0.2230584 | 1.25 (1.134–1.377) | 2.75 × 10⁻⁴ |
| btn2a1 | 50,774 | 413 | 0.2082377 | 1.232 (1.124–1.349) | 3.12 × 10⁻⁴ |
| ceacam6 | 43,951 | 351 | 0.1886363 | 1.208 (1.112–1.312) | 3.12 × 10⁻⁴ |
| tgfbr2 | 50,712 | 411 | 0.2057807 | 1.228 (1.123–1.344) | 3.12 × 10⁻⁴ |
| gfra1 | 50,910 | 416 | 0.2243683 | 1.252 (1.134–1.381) | 3.13 × 10⁻⁴ |
| il2ra | 50,196 | 406 | 0.2090984 | 1.233 (1.125–1.351) | 3.13 × 10⁻⁴ |
| lgals4 | 50,526 | 413 | 0.2166905 | 1.242 (1.129–1.366) | 3.13 × 10⁻⁴ |
| cd74 | 50,996 | 413 | 0.2226507 | 1.249 (1.132–1.378) | 3.48 × 10⁻⁴ |
| c7 | 42,969 | 335 | 0.2416046 | 1.273 (1.143–1.418) | 4.15 × 10⁻⁴ |
| dll1 | 50,712 | 411 | 0.2147130 | 1.24 (1.126–1.364) | 4.15 × 10⁻⁴ |
| prg2 | 43,951 | 351 | 0.2271641 | 1.255 (1.134–1.389) | 4.15 × 10⁻⁴ |
| timp1 | 50,013 | 406 | 0.2010639 | 1.223 (1.118–1.337) | 4.15 × 10⁻⁴ |
| csf1 | 50,774 | 413 | 0.2160783 | 1.241 (1.127–1.367) | 4.2 × 10⁻⁴ |
| ncr3lg1 | 43,769 | 349 | 0.2310424 | 1.26 (1.136–1.397) | 4.27 × 10⁻⁴ |
| baiap2 | 49,375 | 399 | 0.2071442 | 1.23 (1.121–1.35) | 4.41 × 10⁻⁴ |
| galnt10 | 50,329 | 411 | 0.2150460 | 1.24 (1.126–1.366) | 4.41 × 10⁻⁴ |
| itgbl1 | 43,951 | 351 | 0.2112847 | 1.235 (1.123–1.358) | 4.41 × 10⁻⁴ |
| rnaset2 | 50,937 | 413 | 0.1997817 | 1.221 (1.116–1.336) | 4.41 × 10⁻⁴ |
| tnfrsf6b | 50,160 | 407 | 0.2002243 | 1.222 (1.117–1.337) | 4.41 × 10⁻⁴ |
| fstl3 | 50,446 | 411 | 0.2235314 | 1.25 (1.131–1.383) | 4.46 × 10⁻⁴ |
| krt19 | 50,408 | 407 | 0.2013612 | 1.223 (1.117–1.34) | 4.71 × 10⁻⁴ |
| pilra | 50,569 | 402 | 0.2291078 | 1.257 (1.134–1.395) | 4.71 × 10⁻⁴ |
| clec7a | 51,111 | 414 | 0.2713406 | 1.312 (1.16–1.484) | 5.19 × 10⁻⁴ |
| cd300a | 42,991 | 341 | 0.2290227 | 1.257 (1.133–1.396) | 5.36 × 10⁻⁴ |
| ly9 | 49,968 | 404 | 0.2011043 | 1.223 (1.115–1.341) | 6.11 × 10⁻⁴ |
| umod | 51,019 | 411 | -0.2072037 | 0.813 (0.739–0.894) | 6.12 × 10⁻⁴ |
| cntn5 | 50,672 | 412 | -0.2335869 | 0.792 (0.711–0.881) | 6.24 × 10⁻⁴ |
| tnfrsf11a | 51,111 | 414 | 0.2066754 | 1.23 (1.118–1.353) | 6.59 × 10⁻⁴ |
| col4a1 | 50,557 | 410 | 0.2054393 | 1.228 (1.117–1.35) | 6.73 × 10⁻⁴ |
| septin8 | 43,771 | 346 | 0.1942350 | 1.214 (1.11–1.328) | 6.73 × 10⁻⁴ |
| ctsd | 47,266 | 394 | 0.1874262 | 1.206 (1.106–1.316) | 7.21 × 10⁻⁴ |
| lrrn1 | 49,616 | 400 | -0.2130253 | 0.808 (0.732–0.892) | 7.21 × 10⁻⁴ |
| ephb4 | 51,179 | 413 | 0.1905310 | 1.21 (1.107–1.322) | 7.35 × 10⁻⁴ |
| npc2 | 43,213 | 345 | 0.2000693 | 1.221 (1.113–1.341) | 7.35 × 10⁻⁴ |
| sirpb1 | 50,444 | 413 | 0.2063830 | 1.229 (1.117–1.353) | 7.35 × 10⁻⁴ |
| cst3 | 50,676 | 414 | 0.2129817 | 1.237 (1.12–1.367) | 7.59 × 10⁻⁴ |
| adm | 50,987 | 416 | 0.2589812 | 1.296 (1.148–1.462) | 7.68 × 10⁻⁴ |
| egfr | 51,179 | 413 | -0.2046061 | 0.815 (0.741–0.897) | 7.69 × 10⁻⁴ |
| tnfrsf10b | 50,160 | 407 | 0.1219487 | 1.13 (1.067–1.196) | 8.43 × 10⁻⁴ |
| ggt5 | 50,160 | 407 | 0.1875883 | 1.206 (1.104–1.318) | 8.44 × 10⁻⁴ |
| il1r1 | 50,487 | 402 | 0.1966051 | 1.217 (1.109–1.336) | 9.56 × 10⁻⁴ |
| rab6a | 50,488 | 408 | -0.2197278 | 0.803 (0.723–0.891) | 9.56 × 10⁻⁴ |
| pdcd1lg2 | 50,329 | 411 | 0.1912492 | 1.211 (1.105–1.326) | 0.001 |
| adgrg1 | 50,329 | 411 | 0.1784848 | 1.195 (1.098–1.302) | 0.001 |
| ntprobnp | 50,557 | 410 | 0.1990882 | 1.22 (1.11–1.342) | 0.001 |
| ada2 | 50,264 | 411 | 0.1994712 | 1.221 (1.109–1.344) | 0.001 |
| tnfrsf4 | 51,028 | 414 | 0.1913852 | 1.211 (1.104–1.328) | 0.001 |
| ulbp2 | 50,160 | 407 | 0.1985495 | 1.22 (1.108–1.343) | 0.001 |
| ltbp2 | 51,179 | 413 | 0.2255348 | 1.253 (1.122–1.399) | 0.001 |
| tnfrsf12a | 50,712 | 411 | 0.2007948 | 1.222 (1.108–1.348) | 0.001 |
| timp4 | 50,569 | 402 | 0.1947970 | 1.215 (1.105–1.336) | 0.001 |
| gipc2 | 43,853 | 348 | 0.1970580 | 1.218 (1.106–1.341) | 0.002 |
| lamp3 | 51,028 | 414 | 0.2193800 | 1.245 (1.118–1.387) | 0.002 |
| reg4 | 50,609 | 413 | 0.1925029 | 1.212 (1.103–1.332) | 0.002 |
| gprc5c | 43,748 | 350 | 0.1680150 | 1.183 (1.089–1.285) | 0.002 |
| npdc1 | 50,557 | 410 | 0.1800964 | 1.197 (1.096–1.308) | 0.002 |
| mertk | 51,111 | 414 | 0.1976970 | 1.219 (1.106–1.343) | 0.002 |
| cdh2 | 50,557 | 410 | 0.2196425 | 1.246 (1.118–1.388) | 0.002 |
| mmp12 | 50,590 | 415 | 0.2068709 | 1.23 (1.11–1.362) | 0.002 |
| tnfrsf9 | 50,160 | 407 | 0.1727152 | 1.189 (1.091–1.295) | 0.002 |
| mdk | 50,558 | 409 | 0.1696140 | 1.185 (1.088–1.29) | 0.002 |
| ren | 50,557 | 410 | 0.1808333 | 1.198 (1.094–1.312) | 0.002 |
| b4galt1 | 50,774 | 413 | 0.1872726 | 1.206 (1.097–1.326) | 0.002 |
| sdc1 | 50,557 | 410 | 0.1770104 | 1.194 (1.091–1.305) | 0.002 |
| mmp7 | 49,561 | 402 | 0.2101483 | 1.234 (1.109–1.373) | 0.002 |
| slamf8 | 49,375 | 399 | 0.2002622 | 1.222 (1.104–1.353) | 0.002 |
| rbfox3 | 43,873 | 349 | 0.1804914 | 1.198 (1.093–1.313) | 0.003 |
| thbs2 | 50,569 | 402 | 0.1718342 | 1.187 (1.087–1.297) | 0.003 |
| reg3a | 51,179 | 413 | 0.1796892 | 1.197 (1.091–1.312) | 0.003 |
| siglec8 | 42,783 | 343 | 0.2107485 | 1.235 (1.108–1.376) | 0.003 |
| nt5c1a | 43,853 | 348 | 0.1666146 | 1.181 (1.084–1.287) | 0.003 |
| ncs1 | 50,987 | 416 | 0.2032320 | 1.225 (1.103–1.361) | 0.003 |
| grpel1 | 50,329 | 411 | 0.1718354 | 1.187 (1.087–1.298) | 0.003 |
| fam3b | 50,329 | 411 | 0.1969577 | 1.218 (1.1–1.348) | 0.003 |
| tff1 | 50,321 | 399 | 0.1804307 | 1.198 (1.091–1.315) | 0.003 |
| reg1a | 50,262 | 411 | 0.1680267 | 1.183 (1.084–1.291) | 0.003 |
| cd80 | 43,947 | 351 | 0.1908916 | 1.21 (1.095–1.338) | 0.004 |
| itga11 | 50,488 | 408 | -0.1898120 | 0.827 (0.749–0.914) | 0.004 |
| klk4 | 50,987 | 416 | 0.1772841 | 1.194 (1.088–1.31) | 0.004 |
| pilrb | 50,557 | 410 | 0.2013767 | 1.223 (1.1–1.359) | 0.004 |
| echdc3 | 43,947 | 351 | 0.1804794 | 1.198 (1.089–1.317) | 0.004 |
| defa1_defa1b | 49,529 | 403 | 0.1600049 | 1.174 (1.079–1.277) | 0.004 |
| kit | 50,679 | 414 | -0.1890389 | 0.828 (0.749–0.915) | 0.004 |
| ccl15 | 51,179 | 413 | 0.1746050 | 1.191 (1.086–1.306) | 0.004 |
| ccl7 | 50,488 | 408 | 0.1631187 | 1.177 (1.08–1.283) | 0.004 |
| pi3 | 49,888 | 408 | 0.1754733 | 1.192 (1.086–1.308) | 0.004 |
| clec1a | 49,637 | 403 | 0.1762768 | 1.193 (1.086–1.31) | 0.004 |
| lair1 | 50,526 | 413 | 0.1633331 | 1.177 (1.079–1.285) | 0.004 |
| reg1b | 51,179 | 413 | 0.1643807 | 1.179 (1.08–1.287) | 0.004 |
| tgoln2 | 43,984 | 348 | 0.1886449 | 1.208 (1.092–1.335) | 0.004 |
| fgfr2 | 50,712 | 411 | 0.1851764 | 1.203 (1.09–1.329) | 0.005 |
| folr1 | 50,480 | 409 | 0.1787348 | 1.196 (1.087–1.316) | 0.005 |
| nectin4 | 50,712 | 411 | 0.1603548 | 1.174 (1.077–1.279) | 0.005 |
| cxcl9 | 50,774 | 413 | 0.1732070 | 1.189 (1.083–1.306) | 0.005 |
| il1rl1 | 51,011 | 411 | 0.1974963 | 1.218 (1.095–1.355) | 0.005 |
| tnfsf13b | 51,096 | 413 | 0.1685532 | 1.184 (1.081–1.296) | 0.005 |
| il10rb | 50,774 | 413 | 0.1675079 | 1.182 (1.08–1.295) | 0.005 |
| creld1 | 42,991 | 341 | 0.2049142 | 1.227 (1.098–1.372) | 0.005 |
| adamtsl2 | 43,951 | 351 | 0.1869377 | 1.206 (1.089–1.335) | 0.006 |
| cd300e | 50,823 | 416 | 0.1808462 | 1.198 (1.086–1.322) | 0.006 |
| esam | 51,179 | 413 | 0.1714929 | 1.187 (1.08–1.305) | 0.007 |
| lrp11 | 50,557 | 410 | 0.1741253 | 1.19 (1.081–1.311) | 0.007 |
| tnfrsf21 | 50,996 | 413 | 0.1703386 | 1.186 (1.079–1.303) | 0.007 |
| tnfrsf8 | 50,996 | 413 | 0.1613771 | 1.175 (1.074–1.285) | 0.007 |
| bsg | 51,028 | 414 | 0.1774312 | 1.194 (1.082–1.318) | 0.007 |
| sel1l | 42,783 | 343 | 0.1286415 | 1.137 (1.059–1.222) | 0.007 |
| thbd | 50,557 | 410 | 0.1687878 | 1.184 (1.078–1.3) | 0.007 |
| ptgds | 50,182 | 410 | 0.1643064 | 1.179 (1.076–1.291) | 0.007 |
| micb_mica | 50,488 | 408 | 0.2171131 | 1.242 (1.101–1.402) | 0.007 |
| mansc1 | 50,712 | 411 | 0.1701881 | 1.186 (1.078–1.304) | 0.007 |
| hnmt | 49,921 | 407 | 0.1824965 | 1.2 (1.084–1.329) | 0.008 |
| inhbb | 42,991 | 341 | 0.1907386 | 1.21 (1.088–1.347) | 0.008 |
| rida | 42,991 | 341 | 0.1846473 | 1.203 (1.084–1.334) | 0.008 |
| lag3 | 50,329 | 411 | 0.1603906 | 1.174 (1.073–1.285) | 0.008 |
| tgfa | 50,488 | 408 | 0.1646596 | 1.179 (1.074–1.294) | 0.008 |
| cstb | 49,858 | 405 | 0.1750616 | 1.191 (1.079–1.315) | 0.008 |
| zbtb17 | 49,637 | 403 | 0.1395794 | 1.15 (1.063–1.244) | 0.008 |
| ceacam8 | 49,637 | 403 | 0.1672374 | 1.182 (1.075–1.3) | 0.009 |
| chchd10 | 43,853 | 348 | 0.1784216 | 1.195 (1.08–1.323) | 0.009 |
| ebi3_il27 | 50,910 | 416 | 0.1697800 | 1.185 (1.076–1.305) | 0.009 |
| igfbp3 | 50,760 | 415 | -0.1586005 | 0.853 (0.78–0.934) | 0.009 |
| agr2 | 48,971 | 397 | 0.1693853 | 1.185 (1.075–1.305) | 0.009 |
| tff2 | 50,690 | 413 | 0.1710865 | 1.187 (1.076–1.309) | 0.010 |
| flt1 | 50,245 | 411 | 0.1358728 | 1.146 (1.059–1.239) | 0.011 |
| cpvl | 50,903 | 416 | 0.1734152 | 1.189 (1.076–1.315) | 0.011 |
| nppb | 49,637 | 403 | 0.1779925 | 1.195 (1.078–1.325) | 0.011 |
| spp1 | 51,099 | 413 | 0.1688956 | 1.184 (1.074–1.306) | 0.011 |
| apom | 50,679 | 414 | -0.1696529 | 0.844 (0.765–0.931) | 0.011 |
| mrc1 | 43,984 | 348 | 0.1872082 | 1.206 (1.081–1.345) | 0.011 |
| ccl11 | 51,111 | 414 | 0.1778380 | 1.195 (1.077–1.325) | 0.012 |
| cd79b | 51,111 | 414 | 0.1583225 | 1.172 (1.068–1.285) | 0.012 |
| il15 | 50,488 | 408 | 0.1567426 | 1.17 (1.067–1.282) | 0.012 |
| ceacam1 | 50,632 | 411 | 0.1803260 | 1.198 (1.077–1.331) | 0.012 |
| tnfrsf19 | 50,987 | 416 | 0.1564599 | 1.169 (1.067–1.282) | 0.012 |
| spink4 | 50,488 | 408 | 0.1583269 | 1.172 (1.067–1.286) | 0.013 |
| chrdl1 | 50,690 | 413 | 0.1820875 | 1.2 (1.077–1.337) | 0.014 |
| fabp1 | 50,526 | 413 | 0.1672053 | 1.182 (1.07–1.306) | 0.014 |
| hspg2 | 50,182 | 409 | 0.1595978 | 1.173 (1.067–1.29) | 0.014 |
| spink6 | 50,987 | 416 | 0.1760816 | 1.193 (1.074–1.325) | 0.014 |
| ctso | 51,111 | 414 | 0.1604320 | 1.174 (1.067–1.292) | 0.015 |
| fgl1 | 43,984 | 348 | 0.1862786 | 1.205 (1.078–1.347) | 0.015 |
| cdcp1 | 50,160 | 407 | 0.1711990 | 1.187 (1.071–1.315) | 0.015 |
| cd83 | 50,488 | 408 | 0.1637750 | 1.178 (1.068–1.3) | 0.015 |
| efcab14 | 43,769 | 349 | 0.1387870 | 1.149 (1.057–1.249) | 0.015 |
| vwc2 | 49,992 | 407 | 0.1700495 | 1.185 (1.07–1.313) | 0.016 |
| spink2 | 42,991 | 341 | 0.1837856 | 1.202 (1.076–1.343) | 0.016 |
| ccl14 | 50,429 | 412 | 0.1480391 | 1.16 (1.061–1.268) | 0.016 |
| msr1 | 50,996 | 413 | 0.1763837 | 1.193 (1.072–1.327) | 0.016 |
| mme | 50,329 | 411 | 0.1592300 | 1.173 (1.065–1.291) | 0.016 |
| enpp7 | 48,992 | 395 | 0.1671919 | 1.182 (1.068–1.308) | 0.016 |
| icam1 | 50,346 | 412 | 0.1600994 | 1.174 (1.065–1.293) | 0.016 |
| tmod4 | 42,386 | 329 | 0.1528171 | 1.165 (1.062–1.278) | 0.016 |
| gchfr | 41,935 | 327 | 0.1611306 | 1.175 (1.065–1.296) | 0.017 |
| timd4 | 50,760 | 415 | 0.1596881 | 1.173 (1.065–1.293) | 0.017 |
| ephb6 | 50,076 | 407 | 0.1557331 | 1.169 (1.063–1.285) | 0.017 |
| f11 | 43,381 | 342 | -0.1978365 | 0.821 (0.727–0.926) | 0.017 |
| hsd17b14 | 43,853 | 348 | 0.1687655 | 1.184 (1.068–1.313) | 0.018 |
| csf1r | 43,984 | 348 | 0.1681270 | 1.183 (1.067–1.311) | 0.018 |
| atraid | 43,769 | 349 | 0.1123294 | 1.119 (1.044–1.199) | 0.018 |
| gfra2 | 50,329 | 411 | 0.1529400 | 1.165 (1.061–1.28) | 0.018 |
| ctsv | 50,820 | 416 | -0.1704889 | 0.843 (0.759–0.937) | 0.019 |
| ifi30 | 42,713 | 339 | 0.1763457 | 1.193 (1.07–1.33) | 0.020 |
| krt18 | 50,329 | 411 | 0.1620477 | 1.176 (1.064–1.3) | 0.020 |
| dcbld2 | 50,712 | 411 | 0.1614809 | 1.175 (1.063–1.299) | 0.020 |
| hepacam2 | 43,853 | 348 | 0.1687585 | 1.184 (1.066–1.315) | 0.020 |
| ly96 | 50,160 | 407 | 0.1469087 | 1.158 (1.057–1.269) | 0.020 |
| wars | 50,569 | 402 | 0.1575766 | 1.171 (1.061–1.291) | 0.021 |
| b2m | 44,019 | 347 | 0.1121600 | 1.119 (1.043–1.2) | 0.021 |
| guca2a | 50,318 | 401 | 0.1522531 | 1.164 (1.059–1.28) | 0.021 |
| tafa5 | 50,329 | 411 | 0.1520167 | 1.164 (1.059–1.28) | 0.021 |
| ogn | 50,569 | 402 | 0.1626802 | 1.177 (1.063–1.303) | 0.021 |
| fnta | 43,748 | 350 | 0.1405541 | 1.151 (1.054–1.257) | 0.022 |
| neb | 42,515 | 334 | 0.1329384 | 1.142 (1.051–1.241) | 0.022 |
| tnfsf13 | 50,774 | 413 | 0.1596743 | 1.173 (1.061–1.297) | 0.022 |
| pcdh1 | 50,950 | 413 | 0.1319264 | 1.141 (1.05–1.24) | 0.022 |
| shbg | 43,984 | 348 | 0.1983765 | 1.219 (1.076–1.382) | 0.022 |
| cenpf | 43,151 | 347 | 0.1506895 | 1.163 (1.057–1.279) | 0.023 |
| hgf | 51,111 | 414 | 0.1653312 | 1.18 (1.063–1.31) | 0.023 |
| vcam1 | 50,679 | 414 | 0.1491173 | 1.161 (1.056–1.276) | 0.023 |
| chchd6 | 43,748 | 350 | 0.1296886 | 1.138 (1.049–1.236) | 0.024 |
| calb1 | 50,329 | 411 | 0.1584029 | 1.172 (1.06–1.296) | 0.024 |
| col5a1 | 42,991 | 341 | 0.1513923 | 1.163 (1.056–1.281) | 0.025 |
| lamb1 | 42,783 | 343 | 0.1707484 | 1.186 (1.064–1.323) | 0.025 |
| nfasc | 51,111 | 414 | 0.1551095 | 1.168 (1.058–1.289) | 0.025 |
| siglec9 | 50,913 | 416 | 0.1548675 | 1.168 (1.058–1.289) | 0.025 |
| spon1 | 50,609 | 413 | 0.1488743 | 1.161 (1.055–1.276) | 0.025 |
| vmo1 | 50,329 | 411 | 0.1634407 | 1.178 (1.06–1.308) | 0.026 |
| cxcl13 | 50,996 | 413 | 0.1240394 | 1.132 (1.045–1.226) | 0.026 |
| fgf23 | 50,903 | 416 | 0.1312222 | 1.14 (1.048–1.241) | 0.026 |
| ncan | 50,996 | 413 | -0.1658008 | 0.847 (0.761–0.943) | 0.026 |
| polr2f | 50,329 | 411 | 0.1248495 | 1.133 (1.045–1.228) | 0.026 |
| sema3g | 42,991 | 341 | 0.1551938 | 1.168 (1.057–1.291) | 0.026 |
| pvr | 50,996 | 413 | 0.1422504 | 1.153 (1.052–1.264) | 0.027 |
| cd4 | 50,407 | 408 | 0.1271099 | 1.136 (1.046–1.233) | 0.027 |
| epo | 50,488 | 408 | 0.1346317 | 1.144 (1.049–1.248) | 0.028 |
| c1ql2 | 42,675 | 335 | -0.1818266 | 0.834 (0.741–0.938) | 0.028 |
| tff3 | 50,514 | 413 | 0.1278389 | 1.136 (1.046–1.235) | 0.028 |
| cend1 | 42,537 | 336 | 0.1244605 | 1.133 (1.044–1.228) | 0.029 |
| efna1 | 50,487 | 402 | 0.1453190 | 1.156 (1.052–1.271) | 0.029 |
| phospho1 | 50,160 | 407 | 0.1532698 | 1.166 (1.054–1.289) | 0.030 |
| cd276 | 50,526 | 413 | 0.1532662 | 1.166 (1.054–1.289) | 0.031 |
| agrn | 50,774 | 413 | 0.1450088 | 1.156 (1.051–1.272) | 0.031 |
| il15ra | 49,616 | 400 | 0.1521658 | 1.164 (1.054–1.287) | 0.031 |
| fbp1 | 50,557 | 410 | 0.1461865 | 1.157 (1.051–1.274) | 0.032 |
| ror1 | 50,557 | 410 | 0.1495463 | 1.161 (1.052–1.282) | 0.032 |
| klrd1 | 51,111 | 414 | 0.1501391 | 1.162 (1.052–1.283) | 0.032 |
| pla2g15 | 50,712 | 411 | 0.1471036 | 1.158 (1.051–1.277) | 0.032 |
| itgav | 50,987 | 416 | -0.1512619 | 0.86 (0.778–0.95) | 0.032 |
| siglec7 | 50,557 | 410 | 0.1520904 | 1.164 (1.053–1.288) | 0.032 |
| cd300lf | 50,987 | 416 | 0.1614791 | 1.175 (1.056–1.308) | 0.033 |
| icam3 | 51,179 | 413 | 0.1389928 | 1.149 (1.048–1.26) | 0.034 |
| pcbd1 | 42,912 | 341 | 0.1562061 | 1.169 (1.054–1.297) | 0.034 |
| ca6 | 50,410 | 401 | -0.1601247 | 0.852 (0.766–0.948) | 0.034 |
| hspa2 | 43,748 | 350 | 0.1285277 | 1.137 (1.044–1.239) | 0.034 |
| sgsh | 43,208 | 344 | 0.1460319 | 1.157 (1.05–1.275) | 0.034 |
| ngfr | 43,947 | 351 | 0.1225952 | 1.13 (1.042–1.227) | 0.034 |
| nhlrc3 | 41,955 | 334 | 0.1750227 | 1.191 (1.06–1.339) | 0.035 |
| cd59 | 48,730 | 391 | 0.1451270 | 1.156 (1.049–1.274) | 0.035 |
| clec5a | 50,391 | 408 | 0.1446678 | 1.156 (1.049–1.273) | 0.035 |
| kel | 49,373 | 399 | 0.1515589 | 1.164 (1.051–1.288) | 0.035 |
| ifngr1 | 50,774 | 413 | 0.1366180 | 1.146 (1.046–1.256) | 0.035 |
| il10 | 49,616 | 400 | 0.1254756 | 1.134 (1.042–1.233) | 0.035 |
| fgfbp1 | 50,712 | 411 | -0.1448991 | 0.865 (0.785–0.954) | 0.036 |
| yap1 | 43,853 | 348 | 0.1610682 | 1.175 (1.054–1.309) | 0.036 |
| ddr1 | 50,996 | 413 | 0.1383692 | 1.148 (1.046–1.261) | 0.037 |
| fuom | 42,991 | 341 | 0.1609323 | 1.175 (1.054–1.309) | 0.037 |
| tnfsf8 | 43,951 | 351 | 0.1485530 | 1.16 (1.049–1.283) | 0.037 |
| igsf8 | 50,557 | 410 | 0.1393598 | 1.15 (1.046–1.263) | 0.037 |
| clec14a | 50,569 | 402 | 0.1445432 | 1.156 (1.048–1.274) | 0.037 |
| agxt | 50,557 | 410 | 0.1395674 | 1.15 (1.046–1.264) | 0.038 |
| apoc1 | 43,934 | 347 | -0.1714452 | 0.842 (0.75–0.947) | 0.038 |
| ccn4 | 50,987 | 416 | 0.1386646 | 1.149 (1.046–1.262) | 0.038 |
| omg | 50,329 | 411 | -0.1446104 | 0.865 (0.784–0.955) | 0.038 |
| prss27 | 50,391 | 408 | 0.1409469 | 1.151 (1.046–1.267) | 0.038 |
| pts | 49,140 | 398 | 0.1403424 | 1.151 (1.046–1.266) | 0.038 |
| sdc4 | 50,557 | 410 | -0.1387987 | 0.87 (0.792–0.957) | 0.038 |
| siglec5 | 50,569 | 402 | 0.1693196 | 1.184 (1.056–1.329) | 0.038 |
| spint2 | 50,774 | 413 | 0.1475905 | 1.159 (1.048–1.282) | 0.038 |
| tpp1 | 50,194 | 408 | 0.1324053 | 1.142 (1.043–1.249) | 0.038 |
| anpep | 50,678 | 414 | 0.1336111 | 1.143 (1.043–1.252) | 0.038 |
| crip2 | 50,160 | 407 | 0.1395380 | 1.15 (1.045–1.265) | 0.039 |
| il19 | 49,295 | 404 | 0.1357834 | 1.145 (1.044–1.257) | 0.039 |
| sncg | 50,160 | 407 | 0.1591897 | 1.173 (1.052–1.308) | 0.039 |
| sorcs2 | 49,375 | 399 | 0.1461928 | 1.157 (1.047–1.279) | 0.040 |
| btn3a2 | 50,488 | 408 | 0.1350737 | 1.145 (1.043–1.256) | 0.040 |
| ncr1 | 50,416 | 408 | 0.1423743 | 1.153 (1.046–1.271) | 0.040 |
| cd14 | 49,537 | 404 | 0.1414427 | 1.152 (1.045–1.27) | 0.040 |
| col18a1 | 50,767 | 410 | 0.1508084 | 1.163 (1.048–1.29) | 0.041 |
| muc13 | 49,373 | 399 | 0.1398212 | 1.15 (1.044–1.266) | 0.041 |
| bcan | 50,160 | 407 | -0.1506458 | 0.86 (0.775–0.954) | 0.041 |
| c1qa | 50,862 | 414 | 0.1411676 | 1.152 (1.045–1.269) | 0.041 |
| ptn | 49,637 | 403 | 0.1449715 | 1.156 (1.046–1.278) | 0.041 |
| tpr | 42,515 | 334 | 0.1203923 | 1.128 (1.038–1.226) | 0.041 |
| myl4 | 42,783 | 343 | 0.1357037 | 1.145 (1.043–1.258) | 0.042 |
| tnr | 50,160 | 407 | -0.1482787 | 0.862 (0.778–0.956) | 0.044 |
| eln | 43,947 | 351 | 0.1968287 | 1.218 (1.062–1.396) | 0.044 |
| stab2 | 43,951 | 351 | 0.1578960 | 1.171 (1.049–1.307) | 0.044 |
| tlr3 | 51,111 | 414 | 0.1480369 | 1.16 (1.046–1.285) | 0.044 |
| ccn3 | 51,098 | 411 | 0.1419023 | 1.152 (1.044–1.272) | 0.044 |
| crh | 49,294 | 399 | -0.1472410 | 0.863 (0.779–0.956) | 0.044 |
| cfd | 44,019 | 347 | 0.1298995 | 1.139 (1.04–1.247) | 0.045 |
| mad1l1 | 49,994 | 407 | 0.1187458 | 1.126 (1.036–1.224) | 0.046 |
| eif4ebp1 | 50,557 | 410 | 0.1346200 | 1.144 (1.041–1.257) | 0.046 |
| trem2 | 50,526 | 413 | 0.1463319 | 1.158 (1.045–1.283) | 0.046 |
| palm | 43,531 | 345 | 0.1481099 | 1.16 (1.045–1.287) | 0.047 |
| serpina1 | 43,203 | 340 | 0.1193095 | 1.127 (1.036–1.225) | 0.047 |
| draxin | 50,160 | 407 | 0.1382151 | 1.148 (1.041–1.266) | 0.049 |
| bcl2l11 | 50,488 | 408 | 0.1513268 | 1.163 (1.045–1.295) | 0.050 |
| fbn2 | 42,754 | 342 | 0.1420848 | 1.153 (1.042–1.275) | 0.050 |
| il18r1 | 51,111 | 414 | 0.1437854 | 1.155 (1.043–1.279) | 0.050 |
| il6 | 50,329 | 411 | 0.1246275 | 1.133 (1.037–1.238) | 0.050 |
| insl5 | 43,769 | 349 | 0.1497219 | 1.162 (1.044–1.292) | 0.050 |
| psap | 43,538 | 347 | 0.1342326 | 1.144 (1.04–1.258) | 0.050 |

**Supplementary Table S14. Additional epidemiological analyses introduced during revision**

S14A summarizes model, lagged, and outcome-definition analyses; S14B reports age- and sex-stratified estimates; S14C summarizes age at first recorded diagnosis; S14D reports agent-specific estimates; S14E reports neurological-disease censoring analyses; and S14F reports GP prescription-supported outcome analyses.

**Supplementary Table S14A. Model, landmark, and outcome-definition analyses**

| **Analysis** | **N** | **Events** | **HR** | **95% CI** | **P** |
| --- | --- | --- | --- | --- | --- |
| Primary analysis | 492,858 | 3,488 | 1.43 | 1.29–1.59 | <0.001 |
| One-year landmark | 491,784 | 3,328 | 1.43 | 1.29–1.59 | <0.001 |
| Two-year landmark | 490,137 | 3,142 | 1.43 | 1.28–1.60 | <0.001 |
| Exclude baseline ASM users | 487,791 | 3,235 | 1.38 | 1.23–1.54 | <0.001 |
| G40-only outcome | 492,858 | 3,383 | 1.45 | 1.31–1.61 | <0.001 |
| Age-stratified Cox model | 492,858 | 3,488 | 1.43 | 1.29–1.59 | <0.001 |
| Time-varying age effect | 492,858 | 3,488 | 1.43 | 1.29–1.59 | <0.001 |

**Supplementary Table S14B. Age- and sex-stratified analyses of PPI use and incident epilepsy**

Age- and sex-stratified Cox models used the Model 2 covariates. The stratifying variable was omitted from its corresponding stratified adjustment set. Interaction P values were obtained from likelihood-ratio tests comparing models with and without the PPI-by-subgroup interaction term.

| **Subgroup** | **N** | **Events** | **HR** | **95% CI** | **P for interaction** |
| --- | --- | --- | --- | --- | --- |
| Female | 268,549 | 1,639 | 1.78 | 1.57–2.02 | 0.205 |
| Male | 224,309 | 1,849 | 1.55 | 1.37–1.76 | 0.205 |
| Age <55 years | 190,118 | 954 | 2.32 | 1.94–2.78 | <0.001 |
| Age 55-64 years | 208,368 | 1,497 | 1.71 | 1.50–1.96 | <0.001 |
| Age ≥65 years | 94,372 | 1,037 | 1.32 | 1.13–1.54 | <0.001 |

**Supplementary Table S14C. Age at first recorded incident epilepsy diagnosis**

| **Statistic or comparison** | **N** | **Mean or mean difference, years** | **Median (IQR) or 95% CI** | **P** |
| --- | --- | --- | --- | --- |
| Overall age at first recorded diagnosis | 3,488 | 66.32 | 67.86 (60.33–73.13) | - |
| Female versus male | 1,639 / 1,849 | -0.80 | -1.39 to -0.21 | 0.008 |
| PPI user versus non-user | 639 / 2,849 | 0.63 | -0.13 to 1.39 | 0.103 |

**Supplementary Table S14D. Agent-specific UK Biobank estimates**

| **Agent** | **N** | **Events** | **HR** | **95% CI** | **P** |
| --- | --- | --- | --- | --- | --- |
| Omeprazole | 27,322 | 360 | 1.46 | 1.29–1.66 | <0.001 |
| Lansoprazole | 16,391 | 192 | 1.24 | 1.05–1.45 | 0.010 |
| Esomeprazole | 1,957 | 32 | 1.74 | 1.21–2.49 | 0.003 |
| Pantoprazole | 884 | 12 | 1.41 | 0.80–2.51 | 0.235 |
| Rabeprazole | 1,045 | 13 | 1.28 | 0.74–2.23 | 0.373 |
| Ranitidine | 8,245 | 88 | 1.11 | 0.90–1.38 | 0.327 |
| Cimetidine | 370 | 0 | Not estimated | - | Sparse events |
| Famotidine | 31 | 0 | Not estimated | - | Sparse events |
| Nizatidine | 198 | 4 | Not estimated | - | Sparse events |

**Supplementary Table S14E. Neurological exclusion and time-updated censoring analyses**

| **Analysis** | **N** | **Events** | **HR** | **95% CI** | **P** |
| --- | --- | --- | --- | --- | --- |
| Stroke | 485,310 | 2,753 | 1.48 | 1.31–1.67 | <0.001 |
| Brain/CNS tumour | 492,264 | 3,193 | 1.45 | 1.30–1.61 | <0.001 |
| Traumatic brain injury | 492,307 | 3,356 | 1.45 | 1.31–1.61 | <0.001 |
| CNS infection | 492,477 | 3,413 | 1.40 | 1.26–1.55 | <0.001 |
| Dementia | 492,782 | 3,270 | 1.44 | 1.29–1.60 | <0.001 |
| Any of the five conditions | 483,855 | 2,381 | 1.47 | 1.29–1.67 | <0.001 |

**Supplementary Table S14F. GP prescription-supported outcome analyses**

| **Outcome definition** | **N** | **Events** | **HR** | **95% CI** | **P** |
| --- | --- | --- | --- | --- | --- |
| Original G40/G41 outcome in GP-linked subset | 175,614 | 593 | 1.58 | 1.24–2.03 | <0.001 |
| G40/G41 plus ASM prescription within 365 days (primary) | 175,614 | 367 | 1.47 | 1.07–2.02 | 0.018 |
| G40/G41 plus any subsequent ASM prescription (secondary) | 175,614 | 395 | 1.54 | 1.13–2.08 | 0.006 |
| G40/G41 plus ASM prescription within 90 days (secondary) | 175,614 | 332 | 1.49 | 1.06–2.09 | 0.020 |

**Supplementary Table S15. Significant associations between PPI use and baseline NMR metabolites**

Associations with FDR < 0.05 are shown. Metabolite concentrations were standardised before analysis; β therefore represents the adjusted SD difference in baseline metabolite level for PPI users versus non-users. Models used Model 2 covariates. Rows are ordered by FDR.

| **Metabolite** | **Abbreviation** | **N** | **β (SD)** | **SE** | **P value** | **FDR** | **Direction** |
| --- | --- | --- | --- | --- | --- | --- | --- |
| Cholesterol in IDL | C_IDL | 480,310 | -0.1806 | 0.0047 | <0.001 | <0.001 | Negative |
| Cholesterol in Large HDL | C_L-HDL | 480,310 | -0.1599 | 0.0040 | <0.001 | <0.001 | Negative |
| Cholesterol in Very Large HDL | C_VL-HDL | 480,310 | -0.1779 | 0.0041 | <0.001 | <0.001 | Negative |
| Cholesterol to Total Lipids in IDL percentage | C/TL_IDL% | 480,310 | -0.2083 | 0.0046 | <0.001 | <0.001 | Negative |
| Cholesterol to Total Lipids in Large HDL percentage | C/TL_L-HDL% | 480,310 | -0.2021 | 0.0043 | <0.001 | <0.001 | Negative |
| Cholesterol to Total Lipids in Large LDL percentage | C/TL_L-LDL% | 480,310 | -0.1932 | 0.0046 | <0.001 | <0.001 | Negative |
| Cholesterol to Total Lipids in Medium VLDL percentage | C/TL_M-VLDL% | 480,310 | -0.1922 | 0.0044 | <0.001 | <0.001 | Negative |
| Cholesterol to Total Lipids in Very Small VLDL percentage | C/TL_VS-VLDL% | 480,310 | -0.2179 | 0.0045 | <0.001 | <0.001 | Negative |
| Cholesteryl Esters in Large HDL | CE_L-HDL | 480,310 | -0.1607 | 0.0040 | <0.001 | <0.001 | Negative |
| Cholesteryl Esters in Very Large HDL | CE_VL-HDL | 480,310 | -0.1768 | 0.0041 | <0.001 | <0.001 | Negative |
| Cholesteryl Esters to Total Lipids in IDL percentage | CE/TL_IDL% | 480,310 | -0.1932 | 0.0047 | <0.001 | <0.001 | Negative |
| Cholesteryl Esters to Total Lipids in Large HDL percentage | CE/TL_L-HDL% | 480,310 | -0.1899 | 0.0044 | <0.001 | <0.001 | Negative |
| Cholesteryl Esters to Total Lipids in Medium VLDL percentage | CE/TL_M-VLDL% | 480,310 | -0.1928 | 0.0044 | <0.001 | <0.001 | Negative |
| Cholesteryl Esters to Total Lipids in Very Small VLDL percentage | CE/TL_VS-VLDL% | 480,310 | -0.2219 | 0.0044 | <0.001 | <0.001 | Negative |
| Concentration of Large HDL Particles | L-HDL-P | 480,310 | -0.1558 | 0.0040 | <0.001 | <0.001 | Negative |
| Concentration of Very Large HDL Particles | VL-HDL-P | 480,310 | -0.1709 | 0.0042 | <0.001 | <0.001 | Negative |
| Free Cholesterol in Large HDL | FC_L-HDL | 480,310 | -0.1559 | 0.0040 | <0.001 | <0.001 | Negative |
| Free Cholesterol in Very Large HDL | FC_VL-HDL | 480,310 | -0.1750 | 0.0043 | <0.001 | <0.001 | Negative |
| Free Cholesterol to Total Lipids in Large HDL percentage | FC/TL_L-HDL% | 480,310 | -0.1808 | 0.0045 | <0.001 | <0.001 | Negative |
| Free Cholesterol to Total Lipids in Large LDL percentage | FC/TL_L-LDL% | 480,310 | -0.2038 | 0.0045 | <0.001 | <0.001 | Negative |
| Free Cholesterol to Total Lipids in Medium HDL percentage | FC/TL_M-HDL% | 480,310 | -0.1875 | 0.0041 | <0.001 | <0.001 | Negative |
| Free Cholesterol to Total Lipids in Medium VLDL percentage | FC/TL_M-VLDL% | 480,310 | -0.1836 | 0.0045 | <0.001 | <0.001 | Negative |
| Free Cholesterol to Total Lipids in Small VLDL percentage | FC/TL_S-VLDL% | 480,310 | -0.1967 | 0.0045 | <0.001 | <0.001 | Negative |
| Linoleic Acid to Total Fatty Acids percentage | LA% | 479,703 | -0.1936 | 0.0044 | <0.001 | <0.001 | Negative |
| Monounsaturated Fatty Acids to Total Fatty Acids percentage | MUFA% | 479,703 | 0.1681 | 0.0043 | <0.001 | <0.001 | Positive |
| Phospholipids to Total Lipids in Large HDL percentage | PL/TL_L-HDL% | 480,310 | 0.2092 | 0.0044 | <0.001 | <0.001 | Positive |
| Phospholipids to Total Lipids in Medium HDL percentage | PL/TL_M-HDL% | 480,310 | 0.1778 | 0.0043 | <0.001 | <0.001 | Positive |
| Phospholipids to Total Lipids in Medium VLDL percentage | PL/TL_M-VLDL% | 480,310 | -0.1790 | 0.0046 | <0.001 | <0.001 | Negative |
| Phospholipids to Total Lipids in Small VLDL percentage | PL/TL_S-VLDL% | 480,310 | -0.1931 | 0.0046 | <0.001 | <0.001 | Negative |
| Total Lipids in Very Large HDL | TL_VL-HDL | 480,310 | -0.1631 | 0.0041 | <0.001 | <0.001 | Negative |
| Triglycerides to Total Lipids in IDL percentage | TG/TL_IDL% | 480,310 | 0.2180 | 0.0046 | <0.001 | <0.001 | Positive |
| Triglycerides to Total Lipids in Large LDL percentage | TG/TL_L-LDL% | 480,310 | 0.1960 | 0.0047 | <0.001 | <0.001 | Positive |
| Triglycerides to Total Lipids in Medium VLDL percentage | TG/TL_M-VLDL% | 480,310 | 0.1921 | 0.0045 | <0.001 | <0.001 | Positive |
| Triglycerides to Total Lipids in Very Small VLDL percentage | TG/TL_VS-VLDL% | 480,310 | 0.2089 | 0.0045 | <0.001 | <0.001 | Positive |
| Cholesteryl Esters in IDL | CE_IDL | 480,310 | -0.1796 | 0.0047 | <0.001 | <0.001 | Negative |
| Free Cholesterol in IDL | FC_IDL | 480,310 | -0.1790 | 0.0047 | <0.001 | <0.001 | Negative |
| Triglycerides to Total Lipids in Small VLDL percentage | TG/TL_S-VLDL% | 480,310 | 0.1764 | 0.0047 | <0.001 | <0.001 | Positive |
| Glycoprotein Acetyls | GlycA | 480,312 | 0.1703 | 0.0045 | <0.001 | <0.001 | Positive |
| Polyunsaturated Fatty Acids to Monounsaturated Fatty Acids ratio | PUFA/MUFA | 479,702 | -0.1582 | 0.0043 | <0.001 | <0.001 | Negative |
| Total Lipids in Large HDL | TL_L-HDL | 480,310 | -0.1477 | 0.0040 | <0.001 | <0.001 | Negative |
| Triglycerides to Total Lipids in Medium LDL percentage | TG/TL_M-LDL% | 480,310 | 0.1751 | 0.0048 | <0.001 | <0.001 | Positive |
| Phospholipids in Very Large HDL | PL_VL-HDL | 480,310 | -0.1522 | 0.0042 | <0.001 | <0.001 | Negative |
| Free Cholesterol in Large LDL | FC_L-LDL | 480,310 | -0.1748 | 0.0048 | <0.001 | <0.001 | Negative |
| Total Esterified Cholesterol | EC | 480,310 | -0.1682 | 0.0047 | <0.001 | <0.001 | Negative |
| Triglycerides to Total Lipids in Small LDL percentage | TG/TL_S-LDL% | 480,310 | 0.1670 | 0.0046 | <0.001 | <0.001 | Positive |
| Polyunsaturated Fatty Acids to Total Fatty Acids percentage | PUFA% | 479,703 | -0.1567 | 0.0044 | <0.001 | <0.001 | Negative |
| HDL Cholesterol | HDL-C | 480,311 | -0.1444 | 0.0040 | <0.001 | <0.001 | Negative |
| Cholesterol to Total Lipids in Medium HDL percentage | C/TL_M-HDL% | 480,310 | -0.1565 | 0.0044 | <0.001 | <0.001 | Negative |
| Cholesteryl Esters in HDL | CE_HDL | 480,310 | -0.1438 | 0.0040 | <0.001 | <0.001 | Negative |
| Free Cholesterol to Total Lipids in Medium LDL percentage | FC/TL_M-LDL% | 480,310 | -0.1586 | 0.0045 | <0.001 | <0.001 | Negative |
| Average Diameter for HDL Particles | HDL-D | 480,310 | -0.1418 | 0.0040 | <0.001 | <0.001 | Negative |
| Total Lipids in IDL | TL_IDL | 480,310 | -0.1654 | 0.0047 | <0.001 | <0.001 | Negative |
| Cholesterol to Total Lipids in Small VLDL percentage | C/TL_S-VLDL% | 480,310 | -0.1647 | 0.0047 | <0.001 | <0.001 | Negative |
| Free Cholesterol in HDL | FC_HDL | 480,310 | -0.1419 | 0.0041 | <0.001 | <0.001 | Negative |
| Total Cholesterol | TC | 480,311 | -0.1634 | 0.0047 | <0.001 | <0.001 | Negative |
| Phospholipids to Total Lipids in Very Small VLDL percentage | PL/TL_VS-VLDL% | 480,310 | 0.1604 | 0.0046 | <0.001 | <0.001 | Positive |
| Phospholipids in IDL | PL_IDL | 480,310 | -0.1621 | 0.0047 | <0.001 | <0.001 | Negative |
| Cholesterol to Total Lipids in Medium LDL percentage | C/TL_M-LDL% | 480,310 | -0.1661 | 0.0048 | <0.001 | <0.001 | Negative |
| Omega-6 Fatty Acids to Total Fatty Acids percentage | n6% | 479,703 | -0.1530 | 0.0045 | <0.001 | <0.001 | Negative |
| Phospholipids in Large HDL | PL_L-HDL | 480,310 | -0.1363 | 0.0040 | <0.001 | <0.001 | Negative |
| Free Cholesterol in LDL | FC_LDL | 480,310 | -0.1636 | 0.0048 | <0.001 | <0.001 | Negative |
| Triglycerides to Total Lipids in Very Large HDL percentage | TG/TL_VL-HDL% | 480,082 | 0.1525 | 0.0046 | <0.001 | <0.001 | Positive |
| Degree of Unsaturation | Unsat | 479,704 | -0.1444 | 0.0044 | <0.001 | <0.001 | Negative |
| Triglycerides to Total Lipids in Large HDL percentage | TG/TL_L-HDL% | 480,310 | 0.1465 | 0.0045 | <0.001 | <0.001 | Positive |
| Triglycerides to Total Lipids in Small HDL percentage | TG/TL_S-HDL% | 480,310 | 0.1409 | 0.0044 | <0.001 | <0.001 | Positive |
| Cholesteryl Esters in Medium VLDL | CE_M-VLDL | 480,310 | -0.1544 | 0.0048 | <0.001 | <0.001 | Negative |
| Free Cholesterol to Total Lipids in Small HDL percentage | FC/TL_S-HDL% | 480,310 | -0.1427 | 0.0045 | <0.001 | <0.001 | Negative |
| Creatinine | CRE | 470,866 | 0.1353 | 0.0043 | <0.001 | <0.001 | Positive |
| Free Cholesterol to Total Lipids in Very Large HDL percentage | FC/TL_VL-HDL% | 480,082 | 0.1320 | 0.0042 | <0.001 | <0.001 | Positive |
| Total Free Cholesterol | TFC | 480,310 | -0.1477 | 0.0048 | <0.001 | <0.001 | Negative |
| Triglycerides to Phosphoglycerides ratio | TG/PG | 479,704 | 0.1315 | 0.0043 | <0.001 | <0.001 | Positive |
| Sphingomyelins | SM | 479,697 | -0.1381 | 0.0045 | <0.001 | <0.001 | Negative |
| Cholesterol in Large LDL | C_L-LDL | 480,310 | -0.1483 | 0.0048 | <0.001 | <0.001 | Negative |
| Clinical LDL Cholesterol | LDL-C_clin | 480,311 | -0.1459 | 0.0049 | <0.001 | <0.001 | Negative |
| Cholesteryl Esters in Very Small VLDL | CE_VS-VLDL | 480,310 | -0.1411 | 0.0047 | <0.001 | <0.001 | Negative |
| Phospholipids in Large LDL | PL_L-LDL | 480,310 | -0.1445 | 0.0048 | <0.001 | <0.001 | Negative |
| Triglycerides to Total Lipids in Very Large VLDL percentage | TG/TL_VL-VLDL% | 476,091 | 0.1378 | 0.0046 | <0.001 | <0.001 | Positive |
| Free Cholesterol in Small LDL | FC_S-LDL | 480,310 | -0.1441 | 0.0049 | <0.001 | <0.001 | Negative |
| Free Cholesterol to Total Lipids in Small LDL percentage | FC/TL_S-LDL% | 480,310 | -0.1368 | 0.0047 | <0.001 | <0.001 | Negative |
| Cholesterol to Total Lipids in Very Large VLDL percentage | C/TL_VL-VLDL% | 476,091 | -0.1320 | 0.0045 | <0.001 | <0.001 | Negative |
| Triglycerides to Total Lipids in Medium HDL percentage | TG/TL_M-HDL% | 480,310 | 0.1304 | 0.0045 | <0.001 | <0.001 | Positive |
| Concentration of IDL Particles | IDL-P | 480,310 | -0.1397 | 0.0048 | <0.001 | <0.001 | Negative |
| Cholesteryl Esters to Total Lipids in Medium HDL percentage | CE/TL_M-HDL% | 480,310 | -0.1308 | 0.0045 | <0.001 | <0.001 | Negative |
| Cholesterol to Total Lipids in Small LDL percentage | C/TL_S-LDL% | 480,310 | -0.1398 | 0.0049 | <0.001 | <0.001 | Negative |
| Total Lipids in Large LDL | TL_L-LDL | 480,310 | -0.1392 | 0.0048 | <0.001 | <0.001 | Negative |
| Cholesteryl Esters in Large LDL | CE_L-LDL | 480,310 | -0.1370 | 0.0048 | <0.001 | <0.001 | Negative |
| Free Cholesterol in Medium LDL | FC_M-LDL | 480,310 | -0.1377 | 0.0049 | <0.001 | <0.001 | Negative |
| Total Lipids in HDL | TL_HDL | 480,310 | -0.1155 | 0.0041 | <0.001 | <0.001 | Negative |
| Triglycerides in Small HDL | TG_S-HDL | 480,310 | 0.1265 | 0.0045 | <0.001 | <0.001 | Positive |
| Cholesteryl Esters to Total Lipids in Large VLDL percentage | CE/TL_L-VLDL% | 480,256 | -0.1270 | 0.0046 | <0.001 | <0.001 | Negative |
| Free Cholesterol to Total Lipids in IDL percentage | FC/TL_IDL% | 480,310 | -0.1329 | 0.0048 | <0.001 | <0.001 | Negative |
| Cholesteryl Esters to Total Lipids in Very Large VLDL percentage | CE/TL_VL-VLDL% | 476,091 | -0.1256 | 0.0046 | <0.001 | <0.001 | Negative |
| Free Cholesterol to Total Lipids in Very Small VLDL percentage | FC/TL_VS-VLDL% | 480,310 | -0.1325 | 0.0048 | <0.001 | <0.001 | Negative |
| LDL Cholesterol | LDL-C | 480,310 | -0.1314 | 0.0049 | <0.001 | <0.001 | Negative |
| Linoleic Acid | LA | 479,703 | -0.1297 | 0.0048 | <0.001 | <0.001 | Negative |
| Free Cholesterol to Total Lipids in Very Large VLDL percentage | FC/TL_VL-VLDL% | 476,091 | -0.1235 | 0.0047 | <0.001 | <0.001 | Negative |
| Cholesterol to Total Lipids in Small HDL percentage | C/TL_S-HDL% | 480,310 | -0.1232 | 0.0047 | <0.001 | <0.001 | Negative |
| Total Cholesterol Minus HDL-C | non-HDL-C | 480,311 | -0.1256 | 0.0049 | <0.001 | <0.001 | Negative |
| Cholesterol in Very Small VLDL | C_VS-VLDL | 480,310 | -0.1221 | 0.0047 | <0.001 | <0.001 | Negative |
| Phospholipids in LDL | PL_LDL | 480,310 | -0.1255 | 0.0049 | <0.001 | <0.001 | Negative |
| Total Concentration of Lipoprotein Particles | Total-P | 480,310 | -0.1126 | 0.0044 | <0.001 | <0.001 | Negative |
| Phospholipids to Total Lipids in Very Large HDL percentage | PL/TL_VL-HDL% | 480,082 | -0.1135 | 0.0045 | <0.001 | <0.001 | Negative |
| Total Phospholipids in Lipoprotein Particles | PL_LipoP | 480,310 | -0.1162 | 0.0046 | <0.001 | <0.001 | Negative |
| Cholesteryl Esters to Total Lipids in Small VLDL percentage | CE/TL_S-VLDL% | 480,310 | -0.1224 | 0.0048 | <0.001 | <0.001 | Negative |
| Cholesterol in Medium VLDL | C_M-VLDL | 480,310 | -0.1214 | 0.0049 | <0.001 | <0.001 | Negative |
| Total Lipids in LDL | TL_LDL | 480,310 | -0.1213 | 0.0049 | <0.001 | <0.001 | Negative |
| Omega-6 Fatty Acids | n6FA | 479,703 | -0.1191 | 0.0048 | <0.001 | <0.001 | Negative |
| Free Cholesterol in Medium HDL | FC_M-HDL | 480,310 | -0.1037 | 0.0042 | <0.001 | <0.001 | Negative |
| Apolipoprotein A1 | ApoA1 | 480,311 | -0.1033 | 0.0042 | <0.001 | <0.001 | Negative |
| Total Cholines | TCho | 479,704 | -0.1093 | 0.0045 | <0.001 | <0.001 | Negative |
| Cholesteryl Esters in LDL | CE_LDL | 480,310 | -0.1174 | 0.0049 | <0.001 | <0.001 | Negative |
| Remnant Cholesterol (Non-HDL, Non-LDL -Cholesterol) | Remnant-C | 480,310 | -0.1148 | 0.0048 | <0.001 | <0.001 | Negative |
| Cholesterol in Medium HDL | C_M-HDL | 480,310 | -0.0991 | 0.0042 | <0.001 | <0.001 | Negative |
| Concentration of Large LDL Particles | L-LDL-P | 480,310 | -0.1136 | 0.0049 | <0.001 | <0.001 | Negative |
| Cholesteryl Esters in Medium HDL | CE_M-HDL | 480,310 | -0.0974 | 0.0042 | <0.001 | <0.001 | Negative |
| Concentration of HDL Particles | HDL-P | 480,310 | -0.1008 | 0.0044 | <0.001 | <0.001 | Negative |
| Phospholipids in HDL | PL_HDL | 480,310 | -0.0960 | 0.0042 | <0.001 | <0.001 | Negative |
| Polyunsaturated Fatty Acids | PUFA | 479,703 | -0.1080 | 0.0048 | <0.001 | <0.001 | Negative |
| Phospholipids in Small LDL | PL_S-LDL | 480,310 | -0.1099 | 0.0049 | <0.001 | <0.001 | Negative |
| Valine | Val | 479,951 | -0.1031 | 0.0046 | <0.001 | <0.001 | Negative |
| Glucose-lactate | Glu-Lac | 479,065 | 0.1072 | 0.0048 | <0.001 | <0.001 | Positive |
| Average Diameter for VLDL Particles | VLDL-D | 480,310 | 0.0960 | 0.0043 | <0.001 | <0.001 | Positive |
| Triglycerides in Very Large VLDL | TG_VL-VLDL | 480,310 | 0.0997 | 0.0045 | <0.001 | <0.001 | Positive |
| Concentration of LDL Particles | LDL-P | 480,310 | -0.1053 | 0.0049 | <0.001 | <0.001 | Negative |
| Cholesterol in Small LDL | C_S-LDL | 480,310 | -0.1052 | 0.0049 | <0.001 | <0.001 | Negative |
| Apolipoprotein B | ApoB | 480,311 | -0.1039 | 0.0049 | <0.001 | <0.001 | Negative |
| Phosphatidylcholines | PC | 479,704 | -0.0949 | 0.0045 | <0.001 | <0.001 | Negative |
| Cholesterol to Total Lipids in Large VLDL percentage | C/TL_L-VLDL% | 480,256 | -0.0983 | 0.0048 | <0.001 | <0.001 | Negative |
| Concentration of Medium HDL Particles | M-HDL-P | 480,310 | -0.0877 | 0.0042 | <0.001 | <0.001 | Negative |
| Phospholipids in Chylomicrons and Extremely Large VLDL | PL_CM+XL-VLDL | 480,310 | 0.0929 | 0.0045 | <0.001 | <0.001 | Positive |
| Glucose | GLU | 479,389 | 0.0985 | 0.0048 | <0.001 | <0.001 | Positive |
| Triglycerides in Chylomicrons and Extremely Large VLDL | TG_CM+XL-VLDL | 480,310 | 0.0918 | 0.0046 | <0.001 | <0.001 | Positive |
| Triglycerides in Small VLDL | TG_S-VLDL | 480,310 | 0.0921 | 0.0046 | <0.001 | <0.001 | Positive |
| Total Lipids in Chylomicrons and Extremely Large VLDL | TL_CM+XL-VLDL | 480,310 | 0.0903 | 0.0045 | <0.001 | <0.001 | Positive |
| Average Diameter for LDL Particles | LDL-D | 480,310 | -0.0924 | 0.0047 | <0.001 | <0.001 | Negative |
| Total Lipids in Small LDL | TL_S-LDL | 480,310 | -0.0959 | 0.0049 | <0.001 | <0.001 | Negative |
| Total Lipids in Lipoprotein Particles | TL_LipoP | 480,310 | -0.0936 | 0.0048 | <0.001 | <0.001 | Negative |
| Concentration of Chylomicrons and Extremely Large VLDL Particles | CM+XL-VLDL-P | 480,310 | 0.0885 | 0.0046 | <0.001 | <0.001 | Positive |
| Triglycerides in VLDL | TG_VLDL | 480,310 | 0.0873 | 0.0046 | <0.001 | <0.001 | Positive |
| Triglycerides in Very Small VLDL | TG_VS-VLDL | 480,310 | 0.0897 | 0.0047 | <0.001 | <0.001 | Positive |
| Cholesterol in Medium LDL | C_M-LDL | 480,310 | -0.0922 | 0.0049 | <0.001 | <0.001 | Negative |
| Concentration of Very Large VLDL Particles | VL-VLDL-P | 480,310 | 0.0854 | 0.0045 | <0.001 | <0.001 | Positive |
| Total Lipids in Very Large VLDL | TL_VL-VLDL | 480,310 | 0.0852 | 0.0045 | <0.001 | <0.001 | Positive |
| Total Triglycerides | TG | 480,311 | 0.0866 | 0.0046 | <0.001 | <0.001 | Positive |
| Phosphoglycerides | PG | 479,704 | -0.0853 | 0.0045 | <0.001 | <0.001 | Negative |
| Free Cholesterol in Chylomicrons and Extremely Large VLDL | FC_CM+XL-VLDL | 480,310 | 0.0856 | 0.0046 | <0.001 | <0.001 | Positive |
| Cholesteryl Esters to Total Lipids in Very Large HDL percentage | CE/TL_VL-HDL% | 480,082 | -0.0909 | 0.0048 | <0.001 | <0.001 | Negative |
| Triglycerides in Medium HDL | TG_M-HDL | 480,310 | 0.0871 | 0.0047 | <0.001 | <0.001 | Positive |
| Concentration of Small LDL Particles | S-LDL-P | 480,310 | -0.0870 | 0.0049 | <0.001 | <0.001 | Negative |
| Phospholipids in Medium LDL | PL_M-LDL | 480,310 | -0.0870 | 0.0049 | <0.001 | <0.001 | Negative |
| Albumin | ALB | 480,249 | -0.0845 | 0.0048 | <0.001 | <0.001 | Negative |
| Cholesteryl Esters in Small LDL | CE_S-LDL | 480,310 | -0.0859 | 0.0049 | <0.001 | <0.001 | Negative |
| Cholesterol in Chylomicrons and Extremely Large VLDL | C_CM+XL-VLDL | 480,310 | 0.0802 | 0.0045 | <0.001 | <0.001 | Positive |
| Phospholipids in Large VLDL | PL_L-VLDL | 480,310 | 0.0797 | 0.0045 | <0.001 | <0.001 | Positive |
| Concentration of Medium LDL Particles | M-LDL-P | 480,310 | -0.0847 | 0.0049 | <0.001 | <0.001 | Negative |
| Cholesteryl Esters to Total Lipids in Small HDL percentage | CE/TL_S-HDL% | 480,310 | -0.0828 | 0.0048 | <0.001 | <0.001 | Negative |
| Phospholipids to Total Lipids in Large VLDL percentage | PL/TL_L-VLDL% | 480,256 | 0.0789 | 0.0046 | <0.001 | <0.001 | Positive |
| Total Lipids in Medium LDL | TL_M-LDL | 480,310 | -0.0833 | 0.0049 | <0.001 | <0.001 | Negative |
| Total Concentration of Branched-Chain Amino Acids (Leucine + Isoleucine + Valine) | BCAA | 479,929 | -0.0791 | 0.0046 | <0.001 | <0.001 | Negative |
| Triglycerides in Large VLDL | TG_L-VLDL | 480,310 | 0.0776 | 0.0046 | <0.001 | <0.001 | Positive |
| Phospholipids in Very Large VLDL | PL_VL-VLDL | 480,310 | 0.0767 | 0.0045 | <0.001 | <0.001 | Positive |
| Triglycerides in HDL | TG_HDL | 480,310 | 0.0793 | 0.0048 | <0.001 | <0.001 | Positive |
| Cholesteryl Esters in Chylomicrons and Extremely Large VLDL | CE_CM+XL-VLDL | 480,310 | 0.0749 | 0.0046 | <0.001 | <0.001 | Positive |
| Concentration of Large VLDL Particles | L-VLDL-P | 480,310 | 0.0737 | 0.0046 | <0.001 | <0.001 | Positive |
| Total Lipids in Medium HDL | TL_M-HDL | 480,310 | -0.0688 | 0.0043 | <0.001 | <0.001 | Negative |
| Triglycerides in Small LDL | TG_S-LDL | 480,310 | 0.0743 | 0.0046 | <0.001 | <0.001 | Positive |
| Glycine | Gly | 479,709 | -0.0716 | 0.0045 | <0.001 | <0.001 | Negative |
| Total Lipids in Large VLDL | TL_L-VLDL | 480,310 | 0.0707 | 0.0046 | <0.001 | <0.001 | Positive |
| Phospholipids to Total Lipids in IDL percentage | PL/TL_IDL% | 480,310 | 0.0751 | 0.0049 | <0.001 | <0.001 | Positive |
| Saturated Fatty Acids to Total Fatty Acids percentage | SFA% | 479,703 | 0.0724 | 0.0047 | <0.001 | <0.001 | Positive |
| Cholesteryl Esters in Medium LDL | CE_M-LDL | 480,310 | -0.0727 | 0.0049 | <0.001 | <0.001 | Negative |
| Free Cholesterol in Very Small VLDL | FC_VS-VLDL | 480,310 | -0.0719 | 0.0048 | <0.001 | <0.001 | Negative |
| Free Cholesterol in Large VLDL | FC_L-VLDL | 480,310 | 0.0678 | 0.0045 | <0.001 | <0.001 | Positive |
| Free Cholesterol in Very Large VLDL | FC_VL-VLDL | 480,310 | 0.0668 | 0.0045 | <0.001 | <0.001 | Positive |
| Free Cholesterol in Medium VLDL | FC_M-VLDL | 480,310 | -0.0695 | 0.0049 | <0.001 | <0.001 | Negative |
| Free Cholesterol in Small VLDL | FC_S-VLDL | 480,310 | -0.0695 | 0.0049 | <0.001 | <0.001 | Negative |
| Monounsaturated Fatty Acids | MUFA | 479,703 | 0.0664 | 0.0047 | <0.001 | <0.001 | Positive |
| Cholesteryl Esters to Total Lipids in Large LDL percentage | CE/TL_L-LDL% | 480,310 | -0.0688 | 0.0049 | <0.001 | <0.001 | Negative |
| Triglycerides in Medium LDL | TG_M-LDL | 480,310 | 0.0659 | 0.0047 | <0.001 | <0.001 | Positive |
| Spectrometer-corrected alanine | Ala_sc | 480,117 | 0.0673 | 0.0048 | <0.001 | <0.001 | Positive |
| Phospholipids to Total Lipids in Chylomicrons and Extremely Large VLDL percentage | PL/TL_CM+XL-VLDL% | 460,111 | 0.0685 | 0.0049 | <0.001 | <0.001 | Positive |
| Acetone | Acetn | 480,308 | -0.0680 | 0.0049 | <0.001 | <0.001 | Negative |
| Triglycerides in Medium VLDL | TG_M-VLDL | 480,310 | 0.0633 | 0.0046 | <0.001 | <0.001 | Positive |
| Concentration of Very Small VLDL Particles | VS-VLDL-P | 480,310 | -0.0630 | 0.0048 | <0.001 | <0.001 | Negative |
| Cholesteryl Esters in VLDL | CE_VLDL | 480,310 | -0.0633 | 0.0048 | <0.001 | <0.001 | Negative |
| Docosahexaenoic Acid | DHA | 479,703 | -0.0595 | 0.0046 | <0.001 | <0.001 | Negative |
| Triglycerides in LDL | TG_LDL | 480,310 | 0.0598 | 0.0047 | <0.001 | <0.001 | Positive |
| Alanine | Ala | 480,116 | 0.0607 | 0.0048 | <0.001 | <0.001 | Positive |
| Triglycerides in IDL | TG_IDL | 480,310 | 0.0594 | 0.0047 | <0.001 | <0.001 | Positive |
| Citrate | CIT | 480,277 | -0.0592 | 0.0048 | <0.001 | <0.001 | Negative |
| 3-Hydroxybutyrate | 3OHB | 470,798 | -0.0606 | 0.0049 | <0.001 | <0.001 | Negative |
| Total Lipids in Very Small VLDL | TL_VS-VLDL | 480,310 | -0.0559 | 0.0048 | <0.001 | <0.001 | Negative |
| Phospholipids in Medium HDL | PL_M-HDL | 480,310 | -0.0492 | 0.0044 | <0.001 | <0.001 | Negative |
| Isoleucine | Ile | 480,250 | -0.0529 | 0.0047 | <0.001 | <0.001 | Negative |
| Triglycerides in Large LDL | TG_L-LDL | 480,310 | 0.0526 | 0.0047 | <0.001 | <0.001 | Positive |
| Leucine | Leu | 480,271 | -0.0512 | 0.0046 | <0.001 | <0.001 | Negative |
| Docosahexaenoic Acid to Total Fatty Acids percentage | DHA% | 479,703 | -0.0480 | 0.0045 | <0.001 | <0.001 | Negative |
| Phenylalanine | Phe | 480,013 | 0.0510 | 0.0049 | <0.001 | <0.001 | Positive |
| Acetoacetate | AcAc | 480,297 | -0.0509 | 0.0049 | <0.001 | <0.001 | Negative |
| Tyrosine | Tyr | 479,865 | -0.0480 | 0.0048 | <0.001 | <0.001 | Negative |
| Cholesterol in Large VLDL | C_L-VLDL | 480,310 | 0.0451 | 0.0046 | <0.001 | <0.001 | Positive |
| Total Lipids in VLDL | TL_VLDL | 480,310 | 0.0446 | 0.0046 | <0.001 | <0.001 | Positive |
| Cholesterol in Very Large VLDL | C_VL-VLDL | 480,310 | 0.0440 | 0.0046 | <0.001 | <0.001 | Positive |
| Phospholipids in Medium VLDL | PL_M-VLDL | 480,310 | -0.0463 | 0.0048 | <0.001 | <0.001 | Negative |
| Free Cholesterol in Small HDL | FC_S-HDL | 480,310 | -0.0414 | 0.0047 | <0.001 | <0.001 | Negative |
| Cholesterol in Small VLDL | C_S-VLDL | 480,310 | -0.0410 | 0.0048 | <0.001 | <0.001 | Negative |
| Cholesterol to Total Lipids in Chylomicrons and Extremely Large VLDL percentage | C/TL_CM+XL-VLDL% | 460,111 | -0.0388 | 0.0049 | <0.001 | <0.001 | Negative |
| Concentration of Medium VLDL Particles | M-VLDL-P | 480,310 | -0.0376 | 0.0048 | <0.001 | <0.001 | Negative |
| Phospholipids to Total Lipids in Small HDL percentage | PL/TL_S-HDL% | 480,310 | 0.0358 | 0.0047 | <0.001 | <0.001 | Positive |
| VLDL Cholesterol | VLDL-C | 480,310 | -0.0366 | 0.0048 | <0.001 | <0.001 | Negative |
| Phospholipids in Very Small VLDL | PL_VS-VLDL | 480,310 | -0.0360 | 0.0048 | <0.001 | <0.001 | Negative |
| Free Cholesterol to Total Lipids in Chylomicrons and Extremely Large VLDL percentage | FC/TL_CM+XL-VLDL% | 460,111 | -0.0362 | 0.0048 | <0.001 | <0.001 | Negative |
| Cholesteryl Esters to Total Lipids in Chylomicrons and Extremely Large VLDL percentage | CE/TL_CM+XL-VLDL% | 460,111 | -0.0346 | 0.0049 | <0.001 | <0.001 | Negative |
| Cholesterol to Total Lipids in Very Large HDL percentage | C/TL_VL-HDL% | 480,082 | 0.0324 | 0.0047 | <0.001 | <0.001 | Positive |
| Concentration of Small VLDL Particles | S-VLDL-P | 480,310 | 0.0327 | 0.0047 | <0.001 | <0.001 | Positive |
| Cholesterol in Small HDL | C_S-HDL | 480,310 | -0.0319 | 0.0048 | <0.001 | <0.001 | Negative |
| Phospholipids in Small VLDL | PL_S-VLDL | 480,310 | -0.0298 | 0.0048 | <0.001 | <0.001 | Negative |
| Triglycerides to Total Lipids in Large VLDL percentage | TG/TL_L-VLDL% | 480,256 | 0.0292 | 0.0049 | <0.001 | <0.001 | Positive |
| Cholesteryl Esters in Small HDL | CE_S-HDL | 480,310 | -0.0270 | 0.0048 | <0.001 | <0.001 | Negative |
| Lactate | LAC | 479,480 | 0.0263 | 0.0049 | <0.001 | <0.001 | Positive |
| Omega-3 Fatty Acids | n3FA | 479,703 | -0.0251 | 0.0047 | <0.001 | <0.001 | Negative |
| Apolipoprotein B to Apolipoprotein A1 ratio | ApoB/ApoA1 | 480,311 | -0.0247 | 0.0047 | <0.001 | <0.001 | Negative |
| Cholesteryl Esters in Small VLDL | CE_S-VLDL | 480,310 | -0.0243 | 0.0048 | <0.001 | <0.001 | Negative |
| Omega-3 Fatty Acids to Total Fatty Acids percentage | n3% | 479,703 | -0.0236 | 0.0047 | <0.001 | <0.001 | Negative |
| Phospholipids in VLDL | PL_VLDL | 480,310 | 0.0229 | 0.0047 | <0.001 | <0.001 | Positive |
| Concentration of Small HDL Particles | S-HDL-P | 480,310 | -0.0220 | 0.0048 | <0.001 | <0.001 | Negative |
| Total Lipids in Small VLDL | TL_S-VLDL | 480,310 | 0.0216 | 0.0047 | <0.001 | <0.001 | Positive |
| Glutamine | Gln | 478,405 | 0.0215 | 0.0048 | <0.001 | <0.001 | Positive |
| Cholesteryl Esters in Very Large VLDL | CE_VL-VLDL | 480,310 | 0.0202 | 0.0046 | <0.001 | <0.001 | Positive |
| Phospholipids to Total Lipids in Very Large VLDL percentage | PL/TL_VL-VLDL% | 476,091 | -0.0206 | 0.0049 | <0.001 | <0.001 | Negative |
| Phospholipids in Small HDL | PL_S-HDL | 480,310 | 0.0197 | 0.0047 | <0.001 | <0.001 | Positive |
| Cholesteryl Esters in Large VLDL | CE_L-VLDL | 480,310 | 0.0193 | 0.0047 | <0.001 | <0.001 | Positive |
| Cholesteryl Esters to Total Lipids in Medium LDL percentage | CE/TL_M-LDL% | 480,310 | 0.0171 | 0.0047 | <0.001 | <0.001 | Positive |
| Phospholipids to Total Lipids in Small LDL percentage | PL/TL_S-LDL% | 480,310 | -0.0172 | 0.0048 | <0.001 | <0.001 | Negative |
| Saturated Fatty Acids | SFA | 479,703 | 0.0170 | 0.0047 | <0.001 | <0.001 | Positive |
| Omega-6 Fatty Acids to Omega-3 Fatty Acids ratio | n6/n3 | 479,674 | -0.0155 | 0.0047 | 0.001 | 0.001 | Negative |
| Total Lipids in Small HDL | TL_S-HDL | 480,310 | 0.0136 | 0.0047 | 0.004 | 0.004 | Positive |
| Triglycerides to Total Lipids in Chylomicrons and Extremely Large VLDL percentage | TG/TL_CM+XL-VLDL% | 460,111 | 0.0143 | 0.0049 | 0.004 | 0.004 | Positive |
| Triglycerides in Large HDL | TG_L-HDL | 480,310 | -0.0126 | 0.0047 | 0.008 | 0.008 | Negative |
| Total Lipids in Medium VLDL | TL_M-VLDL | 480,310 | -0.0102 | 0.0048 | 0.032 | 0.033 | Negative |
| Acetate | Acet | 480,097 | 0.0099 | 0.0049 | 0.042 | 0.044 | Positive |

**Supplementary Table S16. Significant associations between baseline NMR metabolites and incident epilepsy**

All 181 metabolite-epilepsy associations with FDR < 0.05 are shown. Metabolites were standardised before analysis; HRs therefore correspond to a 1-SD higher baseline metabolite level. Models used Model 2 covariates. Rows are ordered by FDR.

| **Metabolite** | **Abbreviation** | **N** | **Events** | **β per SD** | **SE** | **HR per SD (95% CI)** | **P value** | **FDR** | **Direction** |
| --- | --- | --- | --- | --- | --- | --- | --- | --- | --- |
| Cholesterol to Total Lipids in Medium LDL percentage | C/TL_M-LDL% | 480,310 | 3,377 | -0.0882 | 0.0087 | 0.916 (0.900-0.931) | <0.001 | <0.001 | HR < 1 |
| Triglycerides to Total Lipids in Medium LDL percentage | TG/TL_M-LDL% | 480,310 | 3,377 | 0.0979 | 0.0101 | 1.103 (1.081-1.125) | <0.001 | <0.001 | HR > 1 |
| Free Cholesterol in Small LDL | FC_S-LDL | 480,310 | 3,377 | -0.1561 | 0.0173 | 0.855 (0.827-0.885) | <0.001 | <0.001 | HR < 1 |
| Cholesterol to Total Lipids in Small LDL percentage | C/TL_S-LDL% | 480,310 | 3,377 | -0.1092 | 0.0122 | 0.897 (0.875-0.918) | <0.001 | <0.001 | HR < 1 |
| Free Cholesterol in Medium LDL | FC_M-LDL | 480,310 | 3,377 | -0.1556 | 0.0175 | 0.856 (0.827-0.886) | <0.001 | <0.001 | HR < 1 |
| Triglycerides to Total Lipids in Large LDL percentage | TG/TL_L-LDL% | 480,310 | 3,377 | 0.0967 | 0.0109 | 1.102 (1.078-1.125) | <0.001 | <0.001 | HR > 1 |
| Acetate | Acet | 480,097 | 3,377 | 0.0655 | 0.0074 | 1.068 (1.052-1.083) | <0.001 | <0.001 | HR > 1 |
| Cholesterol in Medium LDL | C_M-LDL | 480,310 | 3,377 | -0.1544 | 0.0176 | 0.857 (0.828-0.887) | <0.001 | <0.001 | HR < 1 |
| Cholesterol in Small LDL | C_S-LDL | 480,310 | 3,377 | -0.1533 | 0.0176 | 0.858 (0.829-0.888) | <0.001 | <0.001 | HR < 1 |
| Cholesterol to Total Lipids in Large LDL percentage | C/TL_L-LDL% | 480,310 | 3,377 | -0.0789 | 0.0091 | 0.924 (0.908-0.941) | <0.001 | <0.001 | HR < 1 |
| Cholesteryl Esters in Medium LDL | CE_M-LDL | 480,310 | 3,377 | -0.1505 | 0.0178 | 0.860 (0.831-0.891) | <0.001 | <0.001 | HR < 1 |
| LDL Cholesterol | LDL-C | 480,310 | 3,377 | -0.1496 | 0.0177 | 0.861 (0.832-0.891) | <0.001 | <0.001 | HR < 1 |
| Clinical LDL Cholesterol | LDL-C_clin | 480,311 | 3,377 | -0.1493 | 0.0176 | 0.861 (0.832-0.892) | <0.001 | <0.001 | HR < 1 |
| Cholesteryl Esters to Total Lipids in Large LDL percentage | CE/TL_L-LDL% | 480,310 | 3,377 | -0.0755 | 0.0089 | 0.927 (0.911-0.944) | <0.001 | <0.001 | HR < 1 |
| Cholesteryl Esters in LDL | CE_LDL | 480,310 | 3,377 | -0.1488 | 0.0177 | 0.862 (0.832-0.892) | <0.001 | <0.001 | HR < 1 |
| Phospholipids in Medium LDL | PL_M-LDL | 480,310 | 3,377 | -0.1485 | 0.0177 | 0.862 (0.833-0.892) | <0.001 | <0.001 | HR < 1 |
| Total Lipids in Medium LDL | TL_M-LDL | 480,310 | 3,377 | -0.1485 | 0.0177 | 0.862 (0.833-0.892) | <0.001 | <0.001 | HR < 1 |
| Phospholipids in LDL | PL_LDL | 480,310 | 3,377 | -0.1465 | 0.0176 | 0.864 (0.834-0.894) | <0.001 | <0.001 | HR < 1 |
| Total Lipids in Small LDL | TL_S-LDL | 480,310 | 3,377 | -0.1465 | 0.0176 | 0.864 (0.834-0.894) | <0.001 | <0.001 | HR < 1 |
| Cholesterol in Medium VLDL | C_M-VLDL | 480,310 | 3,377 | -0.1475 | 0.0177 | 0.863 (0.833-0.893) | <0.001 | <0.001 | HR < 1 |
| Free Cholesterol in LDL | FC_LDL | 480,310 | 3,377 | -0.1467 | 0.0177 | 0.864 (0.834-0.894) | <0.001 | <0.001 | HR < 1 |
| Cholesteryl Esters in Small LDL | CE_S-LDL | 480,310 | 3,377 | -0.1459 | 0.0177 | 0.864 (0.835-0.895) | <0.001 | <0.001 | HR < 1 |
| Cholesteryl Esters in Large LDL | CE_L-LDL | 480,310 | 3,377 | -0.1459 | 0.0178 | 0.864 (0.835-0.895) | <0.001 | <0.001 | HR < 1 |
| Cholesterol in Large LDL | C_L-LDL | 480,310 | 3,377 | -0.1452 | 0.0178 | 0.865 (0.835-0.896) | <0.001 | <0.001 | HR < 1 |
| Total Lipids in LDL | TL_LDL | 480,310 | 3,377 | -0.1439 | 0.0176 | 0.866 (0.837-0.896) | <0.001 | <0.001 | HR < 1 |
| Cholesteryl Esters in Medium VLDL | CE_M-VLDL | 480,310 | 3,377 | -0.1439 | 0.0177 | 0.866 (0.836-0.897) | <0.001 | <0.001 | HR < 1 |
| Phospholipids in Large LDL | PL_L-LDL | 480,310 | 3,377 | -0.1433 | 0.0177 | 0.866 (0.837-0.897) | <0.001 | <0.001 | HR < 1 |
| Concentration of Medium LDL Particles | M-LDL-P | 480,310 | 3,377 | -0.1440 | 0.0178 | 0.866 (0.836-0.897) | <0.001 | <0.001 | HR < 1 |
| Concentration of LDL Particles | LDL-P | 480,310 | 3,377 | -0.1433 | 0.0177 | 0.867 (0.837-0.897) | <0.001 | <0.001 | HR < 1 |
| Total Cholesterol Minus HDL-C | non-HDL-C | 480,311 | 3,377 | -0.1424 | 0.0177 | 0.867 (0.838-0.898) | <0.001 | <0.001 | HR < 1 |
| Phospholipids in Small LDL | PL_S-LDL | 480,310 | 3,377 | -0.1403 | 0.0175 | 0.869 (0.840-0.899) | <0.001 | <0.001 | HR < 1 |
| Free Cholesterol in Medium VLDL | FC_M-VLDL | 480,310 | 3,377 | -0.1426 | 0.0179 | 0.867 (0.837-0.898) | <0.001 | <0.001 | HR < 1 |
| Concentration of Small LDL Particles | S-LDL-P | 480,310 | 3,377 | -0.1403 | 0.0177 | 0.869 (0.839-0.900) | <0.001 | <0.001 | HR < 1 |
| Total Lipids in Large LDL | TL_L-LDL | 480,310 | 3,377 | -0.1397 | 0.0178 | 0.870 (0.840-0.900) | <0.001 | <0.001 | HR < 1 |
| Concentration of Large LDL Particles | L-LDL-P | 480,310 | 3,377 | -0.1385 | 0.0177 | 0.871 (0.841-0.901) | <0.001 | <0.001 | HR < 1 |
| Free Cholesterol in Large LDL | FC_L-LDL | 480,310 | 3,377 | -0.1395 | 0.0178 | 0.870 (0.840-0.901) | <0.001 | <0.001 | HR < 1 |
| Apolipoprotein B | ApoB | 480,311 | 3,377 | -0.1379 | 0.0177 | 0.871 (0.842-0.902) | <0.001 | <0.001 | HR < 1 |
| Acetone | Acetn | 480,308 | 3,377 | 0.0711 | 0.0093 | 1.074 (1.054-1.093) | <0.001 | <0.001 | HR > 1 |
| Free Cholesterol in Small VLDL | FC_S-VLDL | 480,310 | 3,377 | -0.1362 | 0.0178 | 0.873 (0.843-0.904) | <0.001 | <0.001 | HR < 1 |
| Concentration of Medium VLDL Particles | M-VLDL-P | 480,310 | 3,377 | -0.1372 | 0.0181 | 0.872 (0.841-0.903) | <0.001 | <0.001 | HR < 1 |
| Phospholipids in Medium VLDL | PL_M-VLDL | 480,310 | 3,377 | -0.1361 | 0.0179 | 0.873 (0.843-0.904) | <0.001 | <0.001 | HR < 1 |
| Remnant Cholesterol (Non-HDL, Non-LDL -Cholesterol) | Remnant-C | 480,310 | 3,377 | -0.1294 | 0.0177 | 0.879 (0.849-0.910) | <0.001 | <0.001 | HR < 1 |
| Total Lipids in Medium VLDL | TL_M-VLDL | 480,310 | 3,377 | -0.1320 | 0.0182 | 0.876 (0.846-0.908) | <0.001 | <0.001 | HR < 1 |
| Total Free Cholesterol | TFC | 480,310 | 3,377 | -0.1294 | 0.0179 | 0.879 (0.848-0.910) | <0.001 | <0.001 | HR < 1 |
| Triglycerides to Total Lipids in IDL percentage | TG/TL_IDL% | 480,310 | 3,377 | 0.1129 | 0.0158 | 1.120 (1.085-1.155) | <0.001 | <0.001 | HR > 1 |
| Cholesterol to Total Lipids in IDL percentage | C/TL_IDL% | 480,310 | 3,377 | -0.1154 | 0.0162 | 0.891 (0.863-0.920) | <0.001 | <0.001 | HR < 1 |
| Cholesteryl Esters to Total Lipids in IDL percentage | CE/TL_IDL% | 480,310 | 3,377 | -0.1178 | 0.0166 | 0.889 (0.860-0.918) | <0.001 | <0.001 | HR < 1 |
| Total Cholesterol | TC | 480,311 | 3,377 | -0.1282 | 0.0181 | 0.880 (0.849-0.911) | <0.001 | <0.001 | HR < 1 |
| Total Esterified Cholesterol | EC | 480,310 | 3,377 | -0.1269 | 0.0182 | 0.881 (0.850-0.913) | <0.001 | <0.001 | HR < 1 |
| Cholesteryl Esters in IDL | CE_IDL | 480,310 | 3,377 | -0.1253 | 0.0181 | 0.882 (0.851-0.914) | <0.001 | <0.001 | HR < 1 |
| Phospholipids in Small VLDL | PL_S-VLDL | 480,310 | 3,377 | -0.1240 | 0.0180 | 0.883 (0.853-0.915) | <0.001 | <0.001 | HR < 1 |
| Cholesteryl Esters in VLDL | CE_VLDL | 480,310 | 3,377 | -0.1225 | 0.0178 | 0.885 (0.854-0.916) | <0.001 | <0.001 | HR < 1 |
| Cholesterol in IDL | C_IDL | 480,310 | 3,377 | -0.1243 | 0.0181 | 0.883 (0.852-0.915) | <0.001 | <0.001 | HR < 1 |
| Linoleic Acid | LA | 479,703 | 3,372 | -0.1213 | 0.0179 | 0.886 (0.855-0.917) | <0.001 | <0.001 | HR < 1 |
| Apolipoprotein B to Apolipoprotein A1 ratio | ApoB/ApoA1 | 480,311 | 3,377 | -0.1242 | 0.0183 | 0.883 (0.852-0.916) | <0.001 | <0.001 | HR < 1 |
| Cholesterol in Small VLDL | C_S-VLDL | 480,310 | 3,377 | -0.1213 | 0.0179 | 0.886 (0.855-0.917) | <0.001 | <0.001 | HR < 1 |
| Concentration of IDL Particles | IDL-P | 480,310 | 3,377 | -0.1180 | 0.0177 | 0.889 (0.858-0.920) | <0.001 | <0.001 | HR < 1 |
| Phospholipids to Total Lipids in Small HDL percentage | PL/TL_S-HDL% | 480,310 | 3,377 | 0.1185 | 0.0178 | 1.126 (1.087-1.166) | <0.001 | <0.001 | HR > 1 |
| VLDL Cholesterol | VLDL-C | 480,310 | 3,377 | -0.1185 | 0.0179 | 0.888 (0.858-0.920) | <0.001 | <0.001 | HR < 1 |
| Free Cholesterol in IDL | FC_IDL | 480,310 | 3,377 | -0.1183 | 0.0180 | 0.888 (0.858-0.920) | <0.001 | <0.001 | HR < 1 |
| Total Lipids in IDL | TL_IDL | 480,310 | 3,377 | -0.1164 | 0.0181 | 0.890 (0.859-0.922) | <0.001 | <0.001 | HR < 1 |
| Cholesteryl Esters in Very Large VLDL | CE_VL-VLDL | 480,310 | 3,377 | -0.1200 | 0.0186 | 0.887 (0.855-0.920) | <0.001 | <0.001 | HR < 1 |
| Polyunsaturated Fatty Acids | PUFA | 479,703 | 3,372 | -0.1164 | 0.0181 | 0.890 (0.859-0.922) | <0.001 | <0.001 | HR < 1 |
| Cholesteryl Esters in Large VLDL | CE_L-VLDL | 480,310 | 3,377 | -0.1184 | 0.0185 | 0.888 (0.857-0.921) | <0.001 | <0.001 | HR < 1 |
| Omega-6 Fatty Acids | n6FA | 479,703 | 3,372 | -0.1132 | 0.0180 | 0.893 (0.862-0.925) | <0.001 | <0.001 | HR < 1 |
| Linoleic Acid to Total Fatty Acids percentage | LA% | 479,703 | 3,372 | -0.1143 | 0.0183 | 0.892 (0.861-0.925) | <0.001 | <0.001 | HR < 1 |
| Phospholipids in IDL | PL_IDL | 480,310 | 3,377 | -0.1127 | 0.0181 | 0.893 (0.862-0.926) | <0.001 | <0.001 | HR < 1 |
| Cholesteryl Esters in Small VLDL | CE_S-VLDL | 480,310 | 3,377 | -0.1113 | 0.0180 | 0.895 (0.864-0.927) | <0.001 | <0.001 | HR < 1 |
| Acetoacetate | AcAc | 480,297 | 3,377 | 0.0616 | 0.0102 | 1.064 (1.042-1.085) | <0.001 | <0.001 | HR > 1 |
| Cholesteryl Esters to Total Lipids in Medium LDL percentage | CE/TL_M-LDL% | 480,310 | 3,377 | -0.0951 | 0.0157 | 0.909 (0.882-0.938) | <0.001 | <0.001 | HR < 1 |
| Free Cholesterol in VLDL | FC_VLDL | 480,310 | 3,377 | -0.1094 | 0.0182 | 0.896 (0.865-0.929) | <0.001 | <0.001 | HR < 1 |
| Total Lipids in Lipoprotein Particles | TL_LipoP | 480,310 | 3,377 | -0.1063 | 0.0178 | 0.899 (0.868-0.931) | <0.001 | <0.001 | HR < 1 |
| Cholesterol in Large VLDL | C_L-VLDL | 480,310 | 3,377 | -0.1066 | 0.0187 | 0.899 (0.867-0.932) | <0.001 | <0.001 | HR < 1 |
| Triglycerides to Total Lipids in Small LDL percentage | TG/TL_S-LDL% | 480,310 | 3,377 | 0.0825 | 0.0146 | 1.086 (1.055-1.118) | <0.001 | <0.001 | HR > 1 |
| Concentration of VLDL Particles | VLDL-P | 480,310 | 3,377 | -0.1006 | 0.0180 | 0.904 (0.873-0.937) | <0.001 | <0.001 | HR < 1 |
| Total Lipids in Small VLDL | TL_S-VLDL | 480,310 | 3,377 | -0.1009 | 0.0182 | 0.904 (0.872-0.937) | <0.001 | <0.001 | HR < 1 |
| Cholesteryl Esters to Total Lipids in Very Large HDL percentage | CE/TL_VL-HDL% | 480,082 | 3,373 | -0.0847 | 0.0155 | 0.919 (0.891-0.947) | <0.001 | <0.001 | HR < 1 |
| Free Cholesterol to Total Lipids in Very Small VLDL percentage | FC/TL_VS-VLDL% | 480,310 | 3,377 | -0.0867 | 0.0159 | 0.917 (0.889-0.946) | <0.001 | <0.001 | HR < 1 |
| Cholesterol to Total Lipids in Very Small VLDL percentage | C/TL_VS-VLDL% | 480,310 | 3,377 | -0.0959 | 0.0176 | 0.909 (0.878-0.940) | <0.001 | <0.001 | HR < 1 |
| Cholesterol in Very Large VLDL | C_VL-VLDL | 480,310 | 3,377 | -0.0996 | 0.0187 | 0.905 (0.873-0.939) | <0.001 | <0.001 | HR < 1 |
| Cholesteryl Esters to Total Lipids in Very Small VLDL percentage | CE/TL_VS-VLDL% | 480,310 | 3,377 | -0.0940 | 0.0179 | 0.910 (0.879-0.943) | <0.001 | <0.001 | HR < 1 |
| Concentration of Small VLDL Particles | S-VLDL-P | 480,310 | 3,377 | -0.0946 | 0.0183 | 0.910 (0.878-0.943) | <0.001 | <0.001 | HR < 1 |
| Phospholipids in VLDL | PL_VLDL | 480,310 | 3,377 | -0.0945 | 0.0183 | 0.910 (0.878-0.943) | <0.001 | <0.001 | HR < 1 |
| Triglycerides in Medium VLDL | TG_M-VLDL | 480,310 | 3,377 | -0.0962 | 0.0186 | 0.908 (0.876-0.942) | <0.001 | <0.001 | HR < 1 |
| Cholesteryl Esters in Very Small VLDL | CE_VS-VLDL | 480,310 | 3,377 | -0.0931 | 0.0181 | 0.911 (0.879-0.944) | <0.001 | <0.001 | HR < 1 |
| Free Cholesterol to Total Lipids in Small VLDL percentage | FC/TL_S-VLDL% | 480,310 | 3,377 | -0.0941 | 0.0183 | 0.910 (0.878-0.943) | <0.001 | <0.001 | HR < 1 |
| Concentration of Small HDL Particles | S-HDL-P | 480,310 | 3,377 | -0.0920 | 0.0180 | 0.912 (0.880-0.945) | <0.001 | <0.001 | HR < 1 |
| Albumin | ALB | 480,249 | 3,376 | -0.0899 | 0.0178 | 0.914 (0.883-0.947) | <0.001 | <0.001 | HR < 1 |
| Triglycerides to Total Lipids in Very Small VLDL percentage | TG/TL_VS-VLDL% | 480,310 | 3,377 | 0.0864 | 0.0174 | 1.090 (1.054-1.128) | <0.001 | <0.001 | HR > 1 |
| Cholesteryl Esters in Small HDL | CE_S-HDL | 480,310 | 3,377 | -0.0892 | 0.0180 | 0.915 (0.883-0.947) | <0.001 | <0.001 | HR < 1 |
| Phospholipids to Total Lipids in Very Small VLDL percentage | PL/TL_VS-VLDL% | 480,310 | 3,377 | 0.0899 | 0.0182 | 1.094 (1.056-1.134) | <0.001 | <0.001 | HR > 1 |
| Phospholipids to Total Lipids in Small VLDL percentage | PL/TL_S-VLDL% | 480,310 | 3,377 | -0.0906 | 0.0183 | 0.913 (0.881-0.947) | <0.001 | <0.001 | HR < 1 |
| Cholesterol in Very Small VLDL | C_VS-VLDL | 480,310 | 3,377 | -0.0883 | 0.0180 | 0.915 (0.884-0.948) | <0.001 | <0.001 | HR < 1 |
| Free Cholesterol in Large VLDL | FC_L-VLDL | 480,310 | 3,377 | -0.0923 | 0.0189 | 0.912 (0.879-0.946) | <0.001 | <0.001 | HR < 1 |
| Total Lipids in VLDL | TL_VLDL | 480,310 | 3,377 | -0.0887 | 0.0185 | 0.915 (0.883-0.949) | <0.001 | <0.001 | HR < 1 |
| Total Lipids in Large VLDL | TL_L-VLDL | 480,310 | 3,377 | -0.0902 | 0.0188 | 0.914 (0.881-0.948) | <0.001 | <0.001 | HR < 1 |
| Degree of Unsaturation | Unsat | 479,704 | 3,372 | -0.0894 | 0.0188 | 0.914 (0.881-0.949) | <0.001 | <0.001 | HR < 1 |
| Average Diameter for HDL Particles | HDL-D | 480,310 | 3,377 | 0.0992 | 0.0209 | 1.104 (1.060-1.150) | <0.001 | <0.001 | HR > 1 |
| Cholesterol to Total Lipids in Small HDL percentage | C/TL_S-HDL% | 480,310 | 3,377 | -0.0815 | 0.0172 | 0.922 (0.891-0.953) | <0.001 | <0.001 | HR < 1 |
| Cholesteryl Esters to Total Lipids in Small HDL percentage | CE/TL_S-HDL% | 480,310 | 3,377 | -0.0802 | 0.0169 | 0.923 (0.893-0.954) | <0.001 | <0.001 | HR < 1 |
| Cholesterol in Small HDL | C_S-HDL | 480,310 | 3,377 | -0.0849 | 0.0181 | 0.919 (0.887-0.952) | <0.001 | <0.001 | HR < 1 |
| Cholesterol to Total Lipids in Very Large HDL percentage | C/TL_VL-HDL% | 480,082 | 3,373 | -0.0851 | 0.0182 | 0.918 (0.886-0.952) | <0.001 | <0.001 | HR < 1 |
| Triglycerides to Total Lipids in Small VLDL percentage | TG/TL_S-VLDL% | 480,310 | 3,377 | 0.0801 | 0.0177 | 1.083 (1.046-1.122) | <0.001 | <0.001 | HR > 1 |
| Valine | Val | 479,951 | 3,367 | -0.0852 | 0.0189 | 0.918 (0.885-0.953) | <0.001 | <0.001 | HR < 1 |
| Triglycerides in Large VLDL | TG_L-VLDL | 480,310 | 3,377 | -0.0834 | 0.0188 | 0.920 (0.887-0.955) | <0.001 | <0.001 | HR < 1 |
| Concentration of Large VLDL Particles | L-VLDL-P | 480,310 | 3,377 | -0.0815 | 0.0188 | 0.922 (0.888-0.956) | <0.001 | <0.001 | HR < 1 |
| Free Cholesterol to Total Lipids in Small LDL percentage | FC/TL_S-LDL% | 480,310 | 3,377 | -0.0712 | 0.0164 | 0.931 (0.902-0.962) | <0.001 | <0.001 | HR < 1 |
| Glucose | GLU | 479,389 | 3,372 | 0.0610 | 0.0141 | 1.063 (1.034-1.093) | <0.001 | <0.001 | HR > 1 |
| Omega-6 Fatty Acids to Omega-3 Fatty Acids ratio | n6/n3 | 479,674 | 3,372 | 0.0497 | 0.0116 | 1.051 (1.027-1.075) | <0.001 | <0.001 | HR > 1 |
| Cholesterol to Total Lipids in Small VLDL percentage | C/TL_S-VLDL% | 480,310 | 3,377 | -0.0741 | 0.0175 | 0.929 (0.897-0.961) | <0.001 | <0.001 | HR < 1 |
| Phospholipids in Large VLDL | PL_L-VLDL | 480,310 | 3,377 | -0.0790 | 0.0189 | 0.924 (0.891-0.959) | <0.001 | <0.001 | HR < 1 |
| Total Phospholipids in Lipoprotein Particles | PL_LipoP | 480,310 | 3,377 | -0.0776 | 0.0186 | 0.925 (0.892-0.960) | <0.001 | <0.001 | HR < 1 |
| Free Cholesterol in Very Small VLDL | FC_VS-VLDL | 480,310 | 3,377 | -0.0742 | 0.0178 | 0.928 (0.897-0.962) | <0.001 | <0.001 | HR < 1 |
| Average Diameter for VLDL Particles | VLDL-D | 480,310 | 3,377 | -0.0785 | 0.0193 | 0.924 (0.890-0.960) | <0.001 | <0.001 | HR < 1 |
| Histidine | His | 479,734 | 3,372 | -0.0761 | 0.0188 | 0.927 (0.893-0.961) | <0.001 | <0.001 | HR < 1 |
| Polyunsaturated Fatty Acids to Total Fatty Acids percentage | PUFA% | 479,703 | 3,372 | -0.0738 | 0.0183 | 0.929 (0.896-0.963) | <0.001 | <0.001 | HR < 1 |
| Phospholipids in Large HDL | PL_L-HDL | 480,310 | 3,377 | 0.0844 | 0.0211 | 1.088 (1.044-1.134) | <0.001 | <0.001 | HR > 1 |
| Free Cholesterol in Very Large VLDL | FC_VL-VLDL | 480,310 | 3,377 | -0.0746 | 0.0187 | 0.928 (0.895-0.963) | <0.001 | <0.001 | HR < 1 |
| Concentration of Very Small VLDL Particles | VS-VLDL-P | 480,310 | 3,377 | -0.0708 | 0.0178 | 0.932 (0.900-0.965) | <0.001 | <0.001 | HR < 1 |
| Phospholipids in Very Large HDL | PL_VL-HDL | 480,310 | 3,377 | 0.0809 | 0.0203 | 1.084 (1.042-1.128) | <0.001 | <0.001 | HR > 1 |
| Total Concentration of Branched-Chain Amino Acids (Leucine + Isoleucine + Valine) | BCAA | 479,929 | 3,367 | -0.0749 | 0.0189 | 0.928 (0.894-0.963) | <0.001 | <0.001 | HR < 1 |
| Glu-Lac | Glu-Lac | 479,065 | 3,367 | 0.0543 | 0.0138 | 1.056 (1.028-1.085) | <0.001 | <0.001 | HR > 1 |
| Cholesteryl Esters to Total Lipids in Small LDL percentage | CE/TL_S-LDL% | 480,310 | 3,377 | -0.0665 | 0.0172 | 0.936 (0.905-0.968) | <0.001 | <0.001 | HR < 1 |
| Omega-3 Fatty Acids | n3FA | 479,703 | 3,372 | -0.0706 | 0.0186 | 0.932 (0.898-0.966) | <0.001 | <0.001 | HR < 1 |
| Phospholipids to Total Lipids in Small LDL percentage | PL/TL_S-LDL% | 480,310 | 3,377 | 0.0654 | 0.0174 | 1.068 (1.032-1.105) | <0.001 | <0.001 | HR > 1 |
| Leucine | Leu | 480,271 | 3,377 | -0.0698 | 0.0188 | 0.933 (0.899-0.968) | <0.001 | <0.001 | HR < 1 |
| Docosahexaenoic Acid | DHA | 479,703 | 3,372 | -0.0710 | 0.0192 | 0.931 (0.897-0.967) | <0.001 | <0.001 | HR < 1 |
| Free Cholesterol to Total Lipids in Large LDL percentage | FC/TL_L-LDL% | 480,310 | 3,377 | -0.0625 | 0.0170 | 0.939 (0.909-0.971) | <0.001 | <0.001 | HR < 1 |
| Total Lipids in Large HDL | TL_L-HDL | 480,310 | 3,377 | 0.0773 | 0.0212 | 1.080 (1.036-1.126) | <0.001 | <0.001 | HR > 1 |
| Phospholipids in Very Large VLDL | PL_VL-VLDL | 480,310 | 3,377 | -0.0673 | 0.0188 | 0.935 (0.901-0.970) | <0.001 | <0.001 | HR < 1 |
| Free Cholesterol to Total Lipids in Medium VLDL percentage | FC/TL_M-VLDL% | 480,310 | 3,377 | -0.0638 | 0.0178 | 0.938 (0.906-0.972) | <0.001 | <0.001 | HR < 1 |
| Phospholipids to Total Lipids in IDL percentage | PL/TL_IDL% | 480,310 | 3,377 | 0.0615 | 0.0173 | 1.063 (1.028-1.100) | <0.001 | <0.001 | HR > 1 |
| Free Cholesterol to Total Lipids in IDL percentage | FC/TL_IDL% | 480,310 | 3,377 | -0.0582 | 0.0165 | 0.943 (0.913-0.975) | <0.001 | <0.001 | HR < 1 |
| Total Lipids in Very Small VLDL | TL_VS-VLDL | 480,310 | 3,377 | -0.0624 | 0.0178 | 0.939 (0.907-0.973) | <0.001 | <0.001 | HR < 1 |
| Total Lipids in Very Large VLDL | TL_VL-VLDL | 480,310 | 3,377 | -0.0653 | 0.0188 | 0.937 (0.903-0.972) | <0.001 | <0.001 | HR < 1 |
| Phospholipids to Total Lipids in Medium VLDL percentage | PL/TL_M-VLDL% | 480,310 | 3,377 | -0.0605 | 0.0175 | 0.941 (0.910-0.974) | <0.001 | 0.001 | HR < 1 |
| Free Cholesterol in Large HDL | FC_L-HDL | 480,310 | 3,377 | 0.0711 | 0.0212 | 1.074 (1.030-1.119) | <0.001 | 0.001 | HR > 1 |
| Monounsaturated Fatty Acids to Total Fatty Acids percentage | MUFA% | 479,703 | 3,372 | 0.0636 | 0.0190 | 1.066 (1.027-1.106) | <0.001 | 0.001 | HR > 1 |
| Sphingomyelins | SM | 479,697 | 3,372 | -0.0628 | 0.0189 | 0.939 (0.905-0.975) | <0.001 | 0.002 | HR < 1 |
| Polyunsaturated Fatty Acids to Monounsaturated Fatty Acids ratio | PUFA/MUFA | 479,702 | 3,372 | -0.0650 | 0.0196 | 0.937 (0.902-0.974) | <0.001 | 0.002 | HR < 1 |
| Concentration of Very Large VLDL Particles | VL-VLDL-P | 480,310 | 3,377 | -0.0617 | 0.0187 | 0.940 (0.906-0.975) | <0.001 | 0.002 | HR < 1 |
| Total Lipids in Very Large HDL | TL_VL-HDL | 480,310 | 3,377 | 0.0671 | 0.0205 | 1.069 (1.027-1.113) | 0.001 | 0.002 | HR > 1 |
| Triglycerides to Total Lipids in Medium VLDL percentage | TG/TL_M-VLDL% | 480,310 | 3,377 | 0.0597 | 0.0182 | 1.061 (1.024-1.100) | 0.001 | 0.002 | HR > 1 |
| Triglycerides in VLDL | TG_VLDL | 480,310 | 3,377 | -0.0607 | 0.0186 | 0.941 (0.907-0.976) | 0.001 | 0.002 | HR < 1 |
| Cholesteryl Esters to Total Lipids in Small VLDL percentage | CE/TL_S-VLDL% | 480,310 | 3,377 | -0.0551 | 0.0171 | 0.946 (0.915-0.979) | 0.001 | 0.002 | HR < 1 |
| Free Cholesterol in Small HDL | FC_S-HDL | 480,310 | 3,377 | -0.0584 | 0.0183 | 0.943 (0.910-0.978) | 0.001 | 0.002 | HR < 1 |
| Total Fatty Acids | TFA | 479,704 | 3,372 | -0.0565 | 0.0178 | 0.945 (0.913-0.979) | 0.001 | 0.003 | HR < 1 |
| Triglycerides in Large HDL | TG_L-HDL | 480,310 | 3,377 | 0.0553 | 0.0174 | 1.057 (1.021-1.094) | 0.001 | 0.003 | HR > 1 |
| Concentration of Large HDL Particles | L-HDL-P | 480,310 | 3,377 | 0.0677 | 0.0213 | 1.070 (1.026-1.116) | 0.001 | 0.003 | HR > 1 |
| Cholesterol to Total Lipids in Medium VLDL percentage | C/TL_M-VLDL% | 480,310 | 3,377 | -0.0582 | 0.0184 | 0.943 (0.910-0.978) | 0.002 | 0.003 | HR < 1 |
| Saturated Fatty Acids to Total Fatty Acids percentage | SFA% | 479,703 | 3,372 | 0.0546 | 0.0173 | 1.056 (1.021-1.093) | 0.002 | 0.003 | HR > 1 |
| 3-Hydroxybutyrate | 3OHB | 470,798 | 3,261 | 0.0453 | 0.0145 | 1.046 (1.017-1.077) | 0.002 | 0.003 | HR > 1 |
| Cholesterol in Large HDL | C_L-HDL | 480,310 | 3,377 | 0.0659 | 0.0213 | 1.068 (1.024-1.114) | 0.002 | 0.003 | HR > 1 |
| Total Lipids in Small HDL | TL_S-HDL | 480,310 | 3,377 | -0.0548 | 0.0182 | 0.947 (0.914-0.981) | 0.003 | 0.004 | HR < 1 |
| Total Cholines | TCho | 479,704 | 3,372 | -0.0572 | 0.0190 | 0.944 (0.910-0.980) | 0.003 | 0.004 | HR < 1 |
| Cholesteryl Esters in Large HDL | CE_L-HDL | 480,310 | 3,377 | 0.0641 | 0.0213 | 1.066 (1.023-1.112) | 0.003 | 0.004 | HR > 1 |
| Omega-6 Fatty Acids to Total Fatty Acids percentage | n6% | 479,703 | 3,372 | -0.0542 | 0.0180 | 0.947 (0.914-0.981) | 0.003 | 0.004 | HR < 1 |
| Cholesteryl Esters to Total Lipids in Medium VLDL percentage | CE/TL_M-VLDL% | 480,310 | 3,377 | -0.0554 | 0.0186 | 0.946 (0.912-0.981) | 0.003 | 0.005 | HR < 1 |
| Citrate | CIT | 480,277 | 3,377 | -0.0521 | 0.0180 | 0.949 (0.916-0.983) | 0.004 | 0.006 | HR < 1 |
| Phospholipids in Very Small VLDL | PL_VS-VLDL | 480,310 | 3,377 | -0.0498 | 0.0178 | 0.951 (0.919-0.985) | 0.005 | 0.008 | HR < 1 |
| Triglycerides in Small VLDL | TG_S-VLDL | 480,310 | 3,377 | -0.0508 | 0.0183 | 0.950 (0.917-0.985) | 0.006 | 0.009 | HR < 1 |
| Phospholipids to Total Lipids in Very Large HDL percentage | PL/TL_VL-HDL% | 480,082 | 3,373 | 0.0523 | 0.0191 | 1.054 (1.015-1.094) | 0.006 | 0.010 | HR > 1 |
| Total Concentration of Lipoprotein Particles | Total-P | 480,310 | 3,377 | -0.0531 | 0.0195 | 0.948 (0.913-0.985) | 0.006 | 0.010 | HR < 1 |
| Triglycerides in Very Large VLDL | TG_VL-VLDL | 480,310 | 3,377 | -0.0508 | 0.0187 | 0.950 (0.916-0.986) | 0.007 | 0.010 | HR < 1 |
| Phospholipids to Total Lipids in Very Large VLDL percentage | PL/TL_VL-VLDL% | 476,091 | 3,347 | -0.0473 | 0.0178 | 0.954 (0.921-0.988) | 0.008 | 0.012 | HR < 1 |
| Total Triglycerides | TG | 480,311 | 3,377 | -0.0483 | 0.0183 | 0.953 (0.919-0.988) | 0.008 | 0.013 | HR < 1 |
| Omega-3 Fatty Acids to Total Fatty Acids percentage | n3% | 479,703 | 3,372 | -0.0486 | 0.0185 | 0.953 (0.919-0.988) | 0.009 | 0.013 | HR < 1 |
| Glutamine | Gln | 478,405 | 3,352 | -0.0458 | 0.0176 | 0.955 (0.923-0.989) | 0.009 | 0.014 | HR < 1 |
| Glycoprotein Acetyls | GlycA | 480,312 | 3,377 | 0.0467 | 0.0182 | 1.048 (1.011-1.086) | 0.010 | 0.015 | HR > 1 |
| Phosphatidylcholines | PC | 479,704 | 3,372 | -0.0483 | 0.0191 | 0.953 (0.918-0.989) | 0.012 | 0.017 | HR < 1 |
| Free Cholesterol to Total Lipids in Medium LDL percentage | FC/TL_M-LDL% | 480,310 | 3,377 | -0.0442 | 0.0179 | 0.957 (0.924-0.991) | 0.013 | 0.020 | HR < 1 |
| Free Cholesterol in Very Large HDL | FC_VL-HDL | 480,310 | 3,377 | 0.0483 | 0.0196 | 1.049 (1.010-1.091) | 0.014 | 0.020 | HR > 1 |
| Phospholipids in HDL | PL_HDL | 480,310 | 3,377 | 0.0494 | 0.0203 | 1.051 (1.010-1.093) | 0.015 | 0.022 | HR > 1 |
| Concentration of Very Large HDL Particles | VL-HDL-P | 480,310 | 3,377 | 0.0497 | 0.0205 | 1.051 (1.009-1.094) | 0.016 | 0.022 | HR > 1 |
| Isoleucine | Ile | 480,250 | 3,377 | -0.0435 | 0.0183 | 0.957 (0.924-0.992) | 0.017 | 0.024 | HR < 1 |
| Phospholipids to Total Lipids in Large HDL percentage | PL/TL_L-HDL% | 480,310 | 3,377 | 0.0417 | 0.0175 | 1.043 (1.007-1.079) | 0.017 | 0.025 | HR > 1 |
| Phosphoglycerides | PG | 479,704 | 3,372 | -0.0444 | 0.0188 | 0.957 (0.922-0.993) | 0.018 | 0.026 | HR < 1 |
| Cholesteryl Esters in Chylomicrons and Extremely Large VLDL | CE_CM+XL-VLDL | 480,310 | 3,377 | -0.0432 | 0.0185 | 0.958 (0.924-0.993) | 0.019 | 0.027 | HR < 1 |
| Cholesterol in Very Large HDL | C_VL-HDL | 480,310 | 3,377 | 0.0479 | 0.0207 | 1.049 (1.007-1.092) | 0.021 | 0.029 | HR > 1 |
| Cholesteryl Esters in Very Large HDL | CE_VL-HDL | 480,310 | 3,377 | 0.0469 | 0.0209 | 1.048 (1.006-1.092) | 0.025 | 0.035 | HR > 1 |
| Phospholipids in Medium HDL | PL_M-HDL | 480,310 | 3,377 | 0.0409 | 0.0193 | 1.042 (1.003-1.082) | 0.034 | 0.047 | HR > 1 |

**Supplementary Table S17. Shared metabolites passing FDR < 0.05 in both association analyses**

Shared metabolites met FDR < 0.05 in both association analyses. PPI-metabolite coefficients are expressed in SD units, and metabolite-epilepsy HRs correspond to a 1-SD higher metabolite level. Concordant direction indicates a positive product of the two standardised association coefficients. Rows are ordered by the metabolite-epilepsy FDR.

| **Metabolite** | **Abbreviation** | **β PPI→metabolite (SD)** | **FDR** | **HR metabolite→epilepsy per SD (95% CI)** | **FDR** | **Direction** |
| --- | --- | --- | --- | --- | --- | --- |
| Cholesterol to Total Lipids in Medium LDL percentage | C/TL_M-LDL% | -0.1661 | <0.001 | 0.916 (0.900-0.931) | <0.001 | Concordant |
| Triglycerides to Total Lipids in Medium LDL percentage | TG/TL_M-LDL% | 0.1751 | <0.001 | 1.103 (1.081-1.125) | <0.001 | Concordant |
| Free Cholesterol in Small LDL | FC_S-LDL | -0.1441 | <0.001 | 0.855 (0.827-0.885) | <0.001 | Concordant |
| Cholesterol to Total Lipids in Small LDL percentage | C/TL_S-LDL% | -0.1398 | <0.001 | 0.897 (0.875-0.918) | <0.001 | Concordant |
| Free Cholesterol in Medium LDL | FC_M-LDL | -0.1377 | <0.001 | 0.856 (0.827-0.886) | <0.001 | Concordant |
| Triglycerides to Total Lipids in Large LDL percentage | TG/TL_L-LDL% | 0.1960 | <0.001 | 1.102 (1.078-1.125) | <0.001 | Concordant |
| Acetate | Acet | 0.0099 | 0.044 | 1.068 (1.052-1.083) | <0.001 | Concordant |
| Cholesterol in Medium LDL | C_M-LDL | -0.0922 | <0.001 | 0.857 (0.828-0.887) | <0.001 | Concordant |
| Cholesterol to Total Lipids in Large LDL percentage | C/TL_L-LDL% | -0.1932 | <0.001 | 0.924 (0.908-0.941) | <0.001 | Concordant |
| Cholesterol in Small LDL | C_S-LDL | -0.1052 | <0.001 | 0.858 (0.829-0.888) | <0.001 | Concordant |
| Clinical LDL Cholesterol | LDL-C_clin | -0.1459 | <0.001 | 0.861 (0.832-0.892) | <0.001 | Concordant |
| LDL Cholesterol | LDL-C | -0.1314 | <0.001 | 0.861 (0.832-0.891) | <0.001 | Concordant |
| Cholesteryl Esters in Medium LDL | CE_M-LDL | -0.0727 | <0.001 | 0.860 (0.831-0.891) | <0.001 | Concordant |
| Cholesteryl Esters to Total Lipids in Large LDL percentage | CE/TL_L-LDL% | -0.0688 | <0.001 | 0.927 (0.911-0.944) | <0.001 | Concordant |
| Cholesteryl Esters in LDL | CE_LDL | -0.1174 | <0.001 | 0.862 (0.832-0.892) | <0.001 | Concordant |
| Phospholipids in Medium LDL | PL_M-LDL | -0.0870 | <0.001 | 0.862 (0.833-0.892) | <0.001 | Concordant |
| Total Lipids in Medium LDL | TL_M-LDL | -0.0833 | <0.001 | 0.862 (0.833-0.892) | <0.001 | Concordant |
| Phospholipids in LDL | PL_LDL | -0.1255 | <0.001 | 0.864 (0.834-0.894) | <0.001 | Concordant |
| Cholesterol in Medium VLDL | C_M-VLDL | -0.1214 | <0.001 | 0.863 (0.833-0.893) | <0.001 | Concordant |
| Total Lipids in Small LDL | TL_S-LDL | -0.0959 | <0.001 | 0.864 (0.834-0.894) | <0.001 | Concordant |
| Free Cholesterol in LDL | FC_LDL | -0.1636 | <0.001 | 0.864 (0.834-0.894) | <0.001 | Concordant |
| Cholesteryl Esters in Small LDL | CE_S-LDL | -0.0859 | <0.001 | 0.864 (0.835-0.895) | <0.001 | Concordant |
| Cholesteryl Esters in Large LDL | CE_L-LDL | -0.1370 | <0.001 | 0.864 (0.835-0.895) | <0.001 | Concordant |
| Cholesterol in Large LDL | C_L-LDL | -0.1483 | <0.001 | 0.865 (0.835-0.896) | <0.001 | Concordant |
| Total Lipids in LDL | TL_LDL | -0.1213 | <0.001 | 0.866 (0.837-0.896) | <0.001 | Concordant |
| Cholesteryl Esters in Medium VLDL | CE_M-VLDL | -0.1544 | <0.001 | 0.866 (0.836-0.897) | <0.001 | Concordant |
| Phospholipids in Large LDL | PL_L-LDL | -0.1445 | <0.001 | 0.866 (0.837-0.897) | <0.001 | Concordant |
| Concentration of Medium LDL Particles | M-LDL-P | -0.0847 | <0.001 | 0.866 (0.836-0.897) | <0.001 | Concordant |
| Concentration of LDL Particles | LDL-P | -0.1053 | <0.001 | 0.867 (0.837-0.897) | <0.001 | Concordant |
| Total Cholesterol Minus HDL-C | non-HDL-C | -0.1256 | <0.001 | 0.867 (0.838-0.898) | <0.001 | Concordant |
| Phospholipids in Small LDL | PL_S-LDL | -0.1099 | <0.001 | 0.869 (0.840-0.899) | <0.001 | Concordant |
| Free Cholesterol in Medium VLDL | FC_M-VLDL | -0.0695 | <0.001 | 0.867 (0.837-0.898) | <0.001 | Concordant |
| Concentration of Small LDL Particles | S-LDL-P | -0.0870 | <0.001 | 0.869 (0.839-0.900) | <0.001 | Concordant |
| Total Lipids in Large LDL | TL_L-LDL | -0.1392 | <0.001 | 0.870 (0.840-0.900) | <0.001 | Concordant |
| Concentration of Large LDL Particles | L-LDL-P | -0.1136 | <0.001 | 0.871 (0.841-0.901) | <0.001 | Concordant |
| Free Cholesterol in Large LDL | FC_L-LDL | -0.1748 | <0.001 | 0.870 (0.840-0.901) | <0.001 | Concordant |
| Apolipoprotein B | ApoB | -0.1039 | <0.001 | 0.871 (0.842-0.902) | <0.001 | Concordant |
| Acetone | Acetn | -0.0680 | <0.001 | 1.074 (1.054-1.093) | <0.001 | Discordant |
| Free Cholesterol in Small VLDL | FC_S-VLDL | -0.0695 | <0.001 | 0.873 (0.843-0.904) | <0.001 | Concordant |
| Concentration of Medium VLDL Particles | M-VLDL-P | -0.0376 | <0.001 | 0.872 (0.841-0.903) | <0.001 | Concordant |
| Phospholipids in Medium VLDL | PL_M-VLDL | -0.0463 | <0.001 | 0.873 (0.843-0.904) | <0.001 | Concordant |
| Remnant Cholesterol (Non-HDL, Non-LDL -Cholesterol) | Remnant-C | -0.1148 | <0.001 | 0.879 (0.849-0.910) | <0.001 | Concordant |
| Total Lipids in Medium VLDL | TL_M-VLDL | -0.0102 | 0.033 | 0.876 (0.846-0.908) | <0.001 | Concordant |
| Total Free Cholesterol | TFC | -0.1477 | <0.001 | 0.879 (0.848-0.910) | <0.001 | Concordant |
| Triglycerides to Total Lipids in IDL percentage | TG/TL_IDL% | 0.2180 | <0.001 | 1.120 (1.085-1.155) | <0.001 | Concordant |
| Cholesterol to Total Lipids in IDL percentage | C/TL_IDL% | -0.2083 | <0.001 | 0.891 (0.863-0.920) | <0.001 | Concordant |
| Cholesteryl Esters to Total Lipids in IDL percentage | CE/TL_IDL% | -0.1932 | <0.001 | 0.889 (0.860-0.918) | <0.001 | Concordant |
| Total Cholesterol | TC | -0.1634 | <0.001 | 0.880 (0.849-0.911) | <0.001 | Concordant |
| Total Esterified Cholesterol | EC | -0.1682 | <0.001 | 0.881 (0.850-0.913) | <0.001 | Concordant |
| Cholesteryl Esters in IDL | CE_IDL | -0.1796 | <0.001 | 0.882 (0.851-0.914) | <0.001 | Concordant |
| Phospholipids in Small VLDL | PL_S-VLDL | -0.0298 | <0.001 | 0.883 (0.853-0.915) | <0.001 | Concordant |
| Cholesterol in IDL | C_IDL | -0.1806 | <0.001 | 0.883 (0.852-0.915) | <0.001 | Concordant |
| Cholesteryl Esters in VLDL | CE_VLDL | -0.0633 | <0.001 | 0.885 (0.854-0.916) | <0.001 | Concordant |
| Linoleic Acid | LA | -0.1297 | <0.001 | 0.886 (0.855-0.917) | <0.001 | Concordant |
| Apolipoprotein B to Apolipoprotein A1 ratio | ApoB/ApoA1 | -0.0247 | <0.001 | 0.883 (0.852-0.916) | <0.001 | Concordant |
| Cholesterol in Small VLDL | C_S-VLDL | -0.0410 | <0.001 | 0.886 (0.855-0.917) | <0.001 | Concordant |
| Concentration of IDL Particles | IDL-P | -0.1397 | <0.001 | 0.889 (0.858-0.920) | <0.001 | Concordant |
| Phospholipids to Total Lipids in Small HDL percentage | PL/TL_S-HDL% | 0.0358 | <0.001 | 1.126 (1.087-1.166) | <0.001 | Concordant |
| VLDL Cholesterol | VLDL-C | -0.0366 | <0.001 | 0.888 (0.858-0.920) | <0.001 | Concordant |
| Free Cholesterol in IDL | FC_IDL | -0.1790 | <0.001 | 0.888 (0.858-0.920) | <0.001 | Concordant |
| Total Lipids in IDL | TL_IDL | -0.1654 | <0.001 | 0.890 (0.859-0.922) | <0.001 | Concordant |
| Cholesteryl Esters in Very Large VLDL | CE_VL-VLDL | 0.0202 | <0.001 | 0.887 (0.855-0.920) | <0.001 | Discordant |
| Polyunsaturated Fatty Acids | PUFA | -0.1080 | <0.001 | 0.890 (0.859-0.922) | <0.001 | Concordant |
| Cholesteryl Esters in Large VLDL | CE_L-VLDL | 0.0193 | <0.001 | 0.888 (0.857-0.921) | <0.001 | Discordant |
| Omega-6 Fatty Acids | n6FA | -0.1191 | <0.001 | 0.893 (0.862-0.925) | <0.001 | Concordant |
| Linoleic Acid to Total Fatty Acids percentage | LA% | -0.1936 | <0.001 | 0.892 (0.861-0.925) | <0.001 | Concordant |
| Phospholipids in IDL | PL_IDL | -0.1621 | <0.001 | 0.893 (0.862-0.926) | <0.001 | Concordant |
| Cholesteryl Esters in Small VLDL | CE_S-VLDL | -0.0243 | <0.001 | 0.895 (0.864-0.927) | <0.001 | Concordant |
| Acetoacetate | AcAc | -0.0509 | <0.001 | 1.064 (1.042-1.085) | <0.001 | Discordant |
| Cholesteryl Esters to Total Lipids in Medium LDL percentage | CE/TL_M-LDL% | 0.0171 | <0.001 | 0.909 (0.882-0.938) | <0.001 | Discordant |
| Total Lipids in Lipoprotein Particles | TL_LipoP | -0.0936 | <0.001 | 0.899 (0.868-0.931) | <0.001 | Concordant |
| Cholesterol in Large VLDL | C_L-VLDL | 0.0451 | <0.001 | 0.899 (0.867-0.932) | <0.001 | Discordant |
| Triglycerides to Total Lipids in Small LDL percentage | TG/TL_S-LDL% | 0.1670 | <0.001 | 1.086 (1.055-1.118) | <0.001 | Concordant |
| Total Lipids in Small VLDL | TL_S-VLDL | 0.0216 | <0.001 | 0.904 (0.872-0.937) | <0.001 | Discordant |
| Cholesteryl Esters to Total Lipids in Very Large HDL percentage | CE/TL_VL-HDL% | -0.0909 | <0.001 | 0.919 (0.891-0.947) | <0.001 | Concordant |
| Free Cholesterol to Total Lipids in Very Small VLDL percentage | FC/TL_VS-VLDL% | -0.1325 | <0.001 | 0.917 (0.889-0.946) | <0.001 | Concordant |
| Cholesterol to Total Lipids in Very Small VLDL percentage | C/TL_VS-VLDL% | -0.2179 | <0.001 | 0.909 (0.878-0.940) | <0.001 | Concordant |
| Cholesterol in Very Large VLDL | C_VL-VLDL | 0.0440 | <0.001 | 0.905 (0.873-0.939) | <0.001 | Discordant |
| Cholesteryl Esters to Total Lipids in Very Small VLDL percentage | CE/TL_VS-VLDL% | -0.2219 | <0.001 | 0.910 (0.879-0.943) | <0.001 | Concordant |
| Concentration of Small VLDL Particles | S-VLDL-P | 0.0327 | <0.001 | 0.910 (0.878-0.943) | <0.001 | Discordant |
| Phospholipids in VLDL | PL_VLDL | 0.0229 | <0.001 | 0.910 (0.878-0.943) | <0.001 | Discordant |
| Triglycerides in Medium VLDL | TG_M-VLDL | 0.0633 | <0.001 | 0.908 (0.876-0.942) | <0.001 | Discordant |
| Cholesteryl Esters in Very Small VLDL | CE_VS-VLDL | -0.1411 | <0.001 | 0.911 (0.879-0.944) | <0.001 | Concordant |
| Free Cholesterol to Total Lipids in Small VLDL percentage | FC/TL_S-VLDL% | -0.1967 | <0.001 | 0.910 (0.878-0.943) | <0.001 | Concordant |
| Concentration of Small HDL Particles | S-HDL-P | -0.0220 | <0.001 | 0.912 (0.880-0.945) | <0.001 | Concordant |
| Albumin | ALB | -0.0845 | <0.001 | 0.914 (0.883-0.947) | <0.001 | Concordant |
| Triglycerides to Total Lipids in Very Small VLDL percentage | TG/TL_VS-VLDL% | 0.2089 | <0.001 | 1.090 (1.054-1.128) | <0.001 | Concordant |
| Cholesteryl Esters in Small HDL | CE_S-HDL | -0.0270 | <0.001 | 0.915 (0.883-0.947) | <0.001 | Concordant |
| Phospholipids to Total Lipids in Very Small VLDL percentage | PL/TL_VS-VLDL% | 0.1604 | <0.001 | 1.094 (1.056-1.134) | <0.001 | Concordant |
| Phospholipids to Total Lipids in Small VLDL percentage | PL/TL_S-VLDL% | -0.1931 | <0.001 | 0.913 (0.881-0.947) | <0.001 | Concordant |
| Cholesterol in Very Small VLDL | C_VS-VLDL | -0.1221 | <0.001 | 0.915 (0.884-0.948) | <0.001 | Concordant |
| Free Cholesterol in Large VLDL | FC_L-VLDL | 0.0678 | <0.001 | 0.912 (0.879-0.946) | <0.001 | Discordant |
| Total Lipids in VLDL | TL_VLDL | 0.0446 | <0.001 | 0.915 (0.883-0.949) | <0.001 | Discordant |
| Total Lipids in Large VLDL | TL_L-VLDL | 0.0707 | <0.001 | 0.914 (0.881-0.948) | <0.001 | Discordant |
| Average Diameter for HDL Particles | HDL-D | -0.1418 | <0.001 | 1.104 (1.060-1.150) | <0.001 | Discordant |
| Degree of Unsaturation | Unsat | -0.1444 | <0.001 | 0.914 (0.881-0.949) | <0.001 | Concordant |
| Cholesterol to Total Lipids in Small HDL percentage | C/TL_S-HDL% | -0.1232 | <0.001 | 0.922 (0.891-0.953) | <0.001 | Concordant |
| Cholesteryl Esters to Total Lipids in Small HDL percentage | CE/TL_S-HDL% | -0.0828 | <0.001 | 0.923 (0.893-0.954) | <0.001 | Concordant |
| Cholesterol in Small HDL | C_S-HDL | -0.0319 | <0.001 | 0.919 (0.887-0.952) | <0.001 | Concordant |
| Cholesterol to Total Lipids in Very Large HDL percentage | C/TL_VL-HDL% | 0.0324 | <0.001 | 0.918 (0.886-0.952) | <0.001 | Discordant |
| Triglycerides to Total Lipids in Small VLDL percentage | TG/TL_S-VLDL% | 0.1764 | <0.001 | 1.083 (1.046-1.122) | <0.001 | Concordant |
| Valine | Val | -0.1031 | <0.001 | 0.918 (0.885-0.953) | <0.001 | Concordant |
| Triglycerides in Large VLDL | TG_L-VLDL | 0.0776 | <0.001 | 0.920 (0.887-0.955) | <0.001 | Discordant |
| Concentration of Large VLDL Particles | L-VLDL-P | 0.0737 | <0.001 | 0.922 (0.888-0.956) | <0.001 | Discordant |
| Free Cholesterol to Total Lipids in Small LDL percentage | FC/TL_S-LDL% | -0.1368 | <0.001 | 0.931 (0.902-0.962) | <0.001 | Concordant |
| Glucose | GLU | 0.0985 | <0.001 | 1.063 (1.034-1.093) | <0.001 | Concordant |
| Omega-6 Fatty Acids to Omega-3 Fatty Acids ratio | n6/n3 | -0.0155 | 0.001 | 1.051 (1.027-1.075) | <0.001 | Discordant |
| Cholesterol to Total Lipids in Small VLDL percentage | C/TL_S-VLDL% | -0.1647 | <0.001 | 0.929 (0.897-0.961) | <0.001 | Concordant |
| Phospholipids in Large VLDL | PL_L-VLDL | 0.0797 | <0.001 | 0.924 (0.891-0.959) | <0.001 | Discordant |
| Total Phospholipids in Lipoprotein Particles | PL_LipoP | -0.1162 | <0.001 | 0.925 (0.892-0.960) | <0.001 | Concordant |
| Free Cholesterol in Very Small VLDL | FC_VS-VLDL | -0.0719 | <0.001 | 0.928 (0.897-0.962) | <0.001 | Concordant |
| Average Diameter for VLDL Particles | VLDL-D | 0.0960 | <0.001 | 0.924 (0.890-0.960) | <0.001 | Discordant |
| Polyunsaturated Fatty Acids to Total Fatty Acids percentage | PUFA% | -0.1567 | <0.001 | 0.929 (0.896-0.963) | <0.001 | Concordant |
| Phospholipids in Large HDL | PL_L-HDL | -0.1363 | <0.001 | 1.088 (1.044-1.134) | <0.001 | Discordant |
| Phospholipids in Very Large HDL | PL_VL-HDL | -0.1522 | <0.001 | 1.084 (1.042-1.128) | <0.001 | Discordant |
| Free Cholesterol in Very Large VLDL | FC_VL-VLDL | 0.0668 | <0.001 | 0.928 (0.895-0.963) | <0.001 | Discordant |
| Concentration of Very Small VLDL Particles | VS-VLDL-P | -0.0630 | <0.001 | 0.932 (0.900-0.965) | <0.001 | Concordant |
| Total Concentration of Branched-Chain Amino Acids (Leucine + Isoleucine + Valine) | BCAA | -0.0791 | <0.001 | 0.928 (0.894-0.963) | <0.001 | Concordant |
| Glucose-lactate | Glu-Lac | 0.1072 | <0.001 | 1.056 (1.028-1.085) | <0.001 | Concordant |
| Omega-3 Fatty Acids | n3FA | -0.0251 | <0.001 | 0.932 (0.898-0.966) | <0.001 | Concordant |
| Phospholipids to Total Lipids in Small LDL percentage | PL/TL_S-LDL% | -0.0172 | <0.001 | 1.068 (1.032-1.105) | <0.001 | Discordant |
| Leucine | Leu | -0.0512 | <0.001 | 0.933 (0.899-0.968) | <0.001 | Concordant |
| Docosahexaenoic Acid | DHA | -0.0595 | <0.001 | 0.931 (0.897-0.967) | <0.001 | Concordant |
| Free Cholesterol to Total Lipids in Large LDL percentage | FC/TL_L-LDL% | -0.2038 | <0.001 | 0.939 (0.909-0.971) | <0.001 | Concordant |
| Total Lipids in Large HDL | TL_L-HDL | -0.1477 | <0.001 | 1.080 (1.036-1.126) | <0.001 | Discordant |
| Phospholipids in Very Large VLDL | PL_VL-VLDL | 0.0767 | <0.001 | 0.935 (0.901-0.970) | <0.001 | Discordant |
| Free Cholesterol to Total Lipids in Medium VLDL percentage | FC/TL_M-VLDL% | -0.1836 | <0.001 | 0.938 (0.906-0.972) | <0.001 | Concordant |
| Phospholipids to Total Lipids in IDL percentage | PL/TL_IDL% | 0.0751 | <0.001 | 1.063 (1.028-1.100) | <0.001 | Concordant |
| Free Cholesterol to Total Lipids in IDL percentage | FC/TL_IDL% | -0.1329 | <0.001 | 0.943 (0.913-0.975) | <0.001 | Concordant |
| Total Lipids in Very Small VLDL | TL_VS-VLDL | -0.0559 | <0.001 | 0.939 (0.907-0.973) | <0.001 | Concordant |
| Total Lipids in Very Large VLDL | TL_VL-VLDL | 0.0852 | <0.001 | 0.937 (0.903-0.972) | <0.001 | Discordant |
| Phospholipids to Total Lipids in Medium VLDL percentage | PL/TL_M-VLDL% | -0.1790 | <0.001 | 0.941 (0.910-0.974) | 0.001 | Concordant |
| Free Cholesterol in Large HDL | FC_L-HDL | -0.1559 | <0.001 | 1.074 (1.030-1.119) | 0.001 | Discordant |
| Monounsaturated Fatty Acids to Total Fatty Acids percentage | MUFA% | 0.1681 | <0.001 | 1.066 (1.027-1.106) | 0.001 | Concordant |
| Sphingomyelins | SM | -0.1381 | <0.001 | 0.939 (0.905-0.975) | 0.002 | Concordant |
| Polyunsaturated Fatty Acids to Monounsaturated Fatty Acids ratio | PUFA/MUFA | -0.1582 | <0.001 | 0.937 (0.902-0.974) | 0.002 | Concordant |
| Concentration of Very Large VLDL Particles | VL-VLDL-P | 0.0854 | <0.001 | 0.940 (0.906-0.975) | 0.002 | Discordant |
| Total Lipids in Very Large HDL | TL_VL-HDL | -0.1631 | <0.001 | 1.069 (1.027-1.113) | 0.002 | Discordant |
| Triglycerides to Total Lipids in Medium VLDL percentage | TG/TL_M-VLDL% | 0.1921 | <0.001 | 1.061 (1.024-1.100) | 0.002 | Concordant |
| Triglycerides in VLDL | TG_VLDL | 0.0873 | <0.001 | 0.941 (0.907-0.976) | 0.002 | Discordant |
| Cholesteryl Esters to Total Lipids in Small VLDL percentage | CE/TL_S-VLDL% | -0.1224 | <0.001 | 0.946 (0.915-0.979) | 0.002 | Concordant |
| Free Cholesterol in Small HDL | FC_S-HDL | -0.0414 | <0.001 | 0.943 (0.910-0.978) | 0.002 | Concordant |
| Triglycerides in Large HDL | TG_L-HDL | -0.0126 | 0.008 | 1.057 (1.021-1.094) | 0.003 | Discordant |
| Concentration of Large HDL Particles | L-HDL-P | -0.1558 | <0.001 | 1.070 (1.026-1.116) | 0.003 | Discordant |
| Cholesterol to Total Lipids in Medium VLDL percentage | C/TL_M-VLDL% | -0.1922 | <0.001 | 0.943 (0.910-0.978) | 0.003 | Concordant |
| Saturated Fatty Acids to Total Fatty Acids percentage | SFA% | 0.0724 | <0.001 | 1.056 (1.021-1.093) | 0.003 | Concordant |
| 3-Hydroxybutyrate | 3OHB | -0.0606 | <0.001 | 1.046 (1.017-1.077) | 0.003 | Discordant |
| Cholesterol in Large HDL | C_L-HDL | -0.1599 | <0.001 | 1.068 (1.024-1.114) | 0.003 | Discordant |
| Total Lipids in Small HDL | TL_S-HDL | 0.0136 | 0.004 | 0.947 (0.914-0.981) | 0.004 | Discordant |
| Total Cholines | TCho | -0.1093 | <0.001 | 0.944 (0.910-0.980) | 0.004 | Concordant |
| Cholesteryl Esters in Large HDL | CE_L-HDL | -0.1607 | <0.001 | 1.066 (1.023-1.112) | 0.004 | Discordant |
| Omega-6 Fatty Acids to Total Fatty Acids percentage | n6% | -0.1530 | <0.001 | 0.947 (0.914-0.981) | 0.004 | Concordant |
| Cholesteryl Esters to Total Lipids in Medium VLDL percentage | CE/TL_M-VLDL% | -0.1928 | <0.001 | 0.946 (0.912-0.981) | 0.005 | Concordant |
| Citrate | CIT | -0.0592 | <0.001 | 0.949 (0.916-0.983) | 0.006 | Concordant |
| Phospholipids in Very Small VLDL | PL_VS-VLDL | -0.0360 | <0.001 | 0.951 (0.919-0.985) | 0.008 | Concordant |
| Triglycerides in Small VLDL | TG_S-VLDL | 0.0921 | <0.001 | 0.950 (0.917-0.985) | 0.009 | Discordant |
| Phospholipids to Total Lipids in Very Large HDL percentage | PL/TL_VL-HDL% | -0.1135 | <0.001 | 1.054 (1.015-1.094) | 0.010 | Discordant |
| Total Concentration of Lipoprotein Particles | Total-P | -0.1126 | <0.001 | 0.948 (0.913-0.985) | 0.010 | Concordant |
| Triglycerides in Very Large VLDL | TG_VL-VLDL | 0.0997 | <0.001 | 0.950 (0.916-0.986) | 0.010 | Discordant |
| Phospholipids to Total Lipids in Very Large VLDL percentage | PL/TL_VL-VLDL% | -0.0206 | <0.001 | 0.954 (0.921-0.988) | 0.012 | Concordant |
| Total Triglycerides | TG | 0.0866 | <0.001 | 0.953 (0.919-0.988) | 0.013 | Discordant |
| Omega-3 Fatty Acids to Total Fatty Acids percentage | n3% | -0.0236 | <0.001 | 0.953 (0.919-0.988) | 0.013 | Concordant |
| Glutamine | Gln | 0.0215 | <0.001 | 0.955 (0.923-0.989) | 0.014 | Discordant |
| Glycoprotein Acetyls | GlycA | 0.1703 | <0.001 | 1.048 (1.011-1.086) | 0.015 | Concordant |
| Phosphatidylcholines | PC | -0.0949 | <0.001 | 0.953 (0.918-0.989) | 0.017 | Concordant |
| Free Cholesterol to Total Lipids in Medium LDL percentage | FC/TL_M-LDL% | -0.1586 | <0.001 | 0.957 (0.924-0.991) | 0.020 | Concordant |
| Free Cholesterol in Very Large HDL | FC_VL-HDL | -0.1750 | <0.001 | 1.049 (1.010-1.091) | 0.020 | Discordant |
| Phospholipids in HDL | PL_HDL | -0.0960 | <0.001 | 1.051 (1.010-1.093) | 0.022 | Discordant |
| Concentration of Very Large HDL Particles | VL-HDL-P | -0.1709 | <0.001 | 1.051 (1.009-1.094) | 0.022 | Discordant |
| Isoleucine | Ile | -0.0529 | <0.001 | 0.957 (0.924-0.992) | 0.024 | Concordant |
| Phospholipids to Total Lipids in Large HDL percentage | PL/TL_L-HDL% | 0.2092 | <0.001 | 1.043 (1.007-1.079) | 0.025 | Concordant |
| Phosphoglycerides | PG | -0.0853 | <0.001 | 0.957 (0.922-0.993) | 0.026 | Concordant |
| Cholesteryl Esters in Chylomicrons and Extremely Large VLDL | CE_CM+XL-VLDL | 0.0749 | <0.001 | 0.958 (0.924-0.993) | 0.027 | Discordant |
| Cholesterol in Very Large HDL | C_VL-HDL | -0.1779 | <0.001 | 1.049 (1.007-1.092) | 0.029 | Discordant |
| Cholesteryl Esters in Very Large HDL | CE_VL-HDL | -0.1768 | <0.001 | 1.048 (1.006-1.092) | 0.035 | Discordant |
| Phospholipids in Medium HDL | PL_M-HDL | -0.0492 | <0.001 | 1.042 (1.003-1.082) | 0.047 | Discordant |

**Supplementary Table S18. Mediation analysis for 12 metabolites**

Mediation analyses used a fixed simple random sample of 100,000 participants drawn without replacement from the 480,315 participants with baseline NMR metabolomics, using a prespecified random seed. Metabolite-specific complete-case samples included 99,733-100,000 participants and 741-744 events; missing values were not imputed. The mediator and outcome models used Model 2 covariates. Indirect, direct, and total effects were estimated on the log-hazard scale and exponentiated to HRs. Percentile 95% CIs were obtained from 200 nonparametric bootstrap samples. Because the finite bootstrap distribution provides limited P-value resolution, inference is presented using percentile 95% CIs rather than bootstrap P values.

| **Metabolite** | **N** | **Events** | **Bootstrap replicates** | **Indirect HR (95% CI)** | **Direct HR (95% CI)** | **Total HR (95% CI)** | **Proportion mediated, % (95% CI)** |
| --- | --- | --- | --- | --- | --- | --- | --- |
| Val | 99,921 | 741 | 200 | 1.004 (0.997-1.013) | 1.700 (1.398-1.991) | 1.708 (1.403-1.999) | 0.8 (-0.6-3.1) |
| Glu-Lac | 99,733 | 743 | 200 | 1.005 (0.995-1.012) | 1.713 (1.407-2.062) | 1.720 (1.417-2.084) | 0.8 (-0.9-2.1) |
| GlycA | 99,998 | 744 | 200 | 1.010 (0.998-1.023) | 1.699 (1.380-2.000) | 1.717 (1.383-2.014) | 1.9 (-0.5-4.7) |
| Unsat | 99,867 | 742 | 200 | 1.013 (1.003-1.023) | 1.702 (1.420-2.098) | 1.725 (1.429-2.119) | 2.4 (0.6-5.0) |
| ApoB | 100,000 | 744 | 200 | 1.016 (1.007-1.025) | 1.690 (1.444-2.011) | 1.717 (1.471-2.034) | 2.9 (1.3-5.6) |
| HDL-D | 99,999 | 744 | 200 | 0.982 (0.971-0.994) | 1.748 (1.428-2.058) | 1.717 (1.404-2.032) | -3.3 (-6.3--1.1) |
| CE_VS-VLDL | 99,999 | 744 | 200 | 1.010 (1.000-1.021) | 1.699 (1.433-2.002) | 1.717 (1.458-2.015) | 1.9 (0.1-4.6) |
| FC_IDL | 99,999 | 744 | 200 | 1.020 (1.004-1.036) | 1.684 (1.371-2.007) | 1.717 (1.401-2.068) | 3.6 (0.7-8.4) |
| PL_IDL | 99,999 | 744 | 200 | 1.016 (1.002-1.027) | 1.690 (1.453-2.021) | 1.717 (1.482-2.073) | 2.9 (0.4-5.7) |
| TG_S-VLDL | 99,999 | 744 | 200 | 0.993 (0.985-0.999) | 1.729 (1.447-2.068) | 1.717 (1.430-2.059) | -1.3 (-3.3--0.2) |
| TL_S-LDL | 99,999 | 744 | 200 | 1.016 (1.008-1.025) | 1.690 (1.393-2.053) | 1.717 (1.416-2.084) | 3.0 (1.3-5.4) |
| C_M-VLDL | 99,999 | 744 | 200 | 1.020 (1.009-1.031) | 1.684 (1.392-2.049) | 1.717 (1.429-2.090) | 3.6 (1.6-7.2) |

**Supplementary Table S19. Shared metabolite distribution by biochemical module**

The table summarises the 176 metabolites associated with both PPI use and incident epilepsy at FDR < 0.05 within the ten biochemical modules displayed in Figure 5D. Fractions use the full module size as the denominator. Net direction is based on the mean product of the standardised PPI-metabolite and metabolite-epilepsy coefficients among shared metabolites.

| **Biochemical module** | **Module total (N)** | **Shared metabolites (N)** | **Shared fraction** | **Mean coefficient product** | **Net association direction** |
| --- | --- | --- | --- | --- | --- |
| Amino acids | 11 | 5 | 45.5% | 0.0039 | Concordant |
| Fatty acids | 17 | 13 | 76.5% | 0.0095 | Concordant |
| Apolipoproteins | 3 | 2 | 66.7% | 0.0087 | Concordant |
| LP particle sizes | 3 | 2 | 66.7% | -0.0108 | Discordant |
| Cholesteryl esters | 31 | 23 | 74.2% | 0.0080 | Concordant |
| Free cholesterol | 32 | 21 | 65.6% | 0.0099 | Concordant |
| Phospholipids | 32 | 25 | 78.1% | 0.0047 | Concordant |
| Triglycerides | 33 | 14 | 42.4% | 0.0061 | Concordant |
| Total lipids | 32 | 24 | 75.0% | 0.0074 | Concordant |
| Cholesterol | 22 | 19 | 86.4% | 0.0107 | Concordant |

Abbreviations: FDR, false discovery rate; NMR, nuclear magnetic resonance; PPI, proton pump inhibitor.

**Supplementary Figure Legends**

**Supplementary Figure S1**

Figure S1. Robustness analyses and propensity-score diagnostics. (A) Alternative Cox specifications, landmark analyses, and the G40-only outcome. (B) Medication and neurological-condition sensitivity analyses. For each neurological condition, baseline cases were excluded and follow-up was censored when the condition occurred strictly before epilepsy. The five-condition joint analysis combined stroke, brain/CNS tumour, traumatic brain injury, CNS infection, and dementia. (C) GP prescription-supported epilepsy analyses in 175,614 participants with eligible primary-care follow-up. The primary confirmation definition required an eligible maintenance ASM prescription within 365 days after G40/G41; any subsequent prescription and a 90-day window were secondary definitions. The confirmed event date was the first qualifying prescription. (D) Absolute standardized mean differences before and after PPI–H2RA propensity score matching. The propensity score included age, sex, body mass index, Townsend deprivation index, smoking, alcohol intake, ethnicity, education, and baseline acid-related gastrointestinal disease (ICD-10 K20, K21, K25–K27, and K29). The dashed line marks |SMD| = 0.10.

**Supplementary Figure S2**

Figure S2. Protein–protein interaction network of FDR-significant candidate mediator proteins. Proteins with FDR < 0.05 for the indirect effect were submitted to STRING v12.0. Functional associations with a combined interaction score ≥0.70 were retained. Node size is proportional to the estimated proportion mediated; edge width and opacity reflect the STRING combined score; edge colour denotes community membership after greedy modularity optimization, with grey for cross-community edges. The network is a descriptive summary of connectivity and does not establish causal or biological centrality.

**Supplementary Figure S3**

Figure S3. Standardised plasma levels of 12 module-representative NMR metabolites by PPI use status. Box plots showing standardised (z-scored) metabolite levels in PPI users (n=49,451) versus non-PPI users (n=430,864) at UK Biobank baseline (total N=480,315). Metabolite levels were standardised to z-scores across the full analytic sample. Box: interquartile range; horizontal line: median; whiskers: 1.5×IQR; outliers shown as semi-transparent dots. Significance markers indicate Wilcoxon rank-sum test results: ***P<0.001, **P<0.01, *P<0.05, ns P≥0.05. Panel colours correspond to the five-colour NMR biomarker class scheme used in Figure 5. Abbreviations: ApoB, apolipoprotein B; CE, cholesteryl esters; FC, free cholesterol; GlycA, glycoprotein acetyls; HDL-D, average diameter of HDL particles; PL, phospholipids; PPI, proton pump inhibitor; TG, triglycerides; TL, total lipids; Unsat, degree of unsaturation; Val, valine.

**Supplementary Figure S4**

Figure S4. Spearman correlation matrix of 12 module-representative NMR metabolites. Pairwise Spearman correlation coefficients among the 12 NMR metabolite module representatives in the full metabolomic analytic sample (N=480,315). Colour scale ranges from blue (r=−1.0) to red (r=+1.0). Metabolite label colours correspond to the five-colour NMR biomarker class scheme used in Figure 5. High within-module correlations (e.g., CE_VS-VLDL, FC_IDL, PL_IDL, TL_S-LDL: r=0.80–0.96) and predominantly low between-module correlations support selection of these metabolites as non-redundant module representatives. Abbreviations: ApoB, apolipoprotein B; CE, cholesteryl esters; FC, free cholesterol; GlycA, glycoprotein acetyls; HDL-D, average diameter of HDL particles; PL, phospholipids; TG, triglycerides; TL, total lipids; Unsat, degree of unsaturation; Val, valine.
